# An Experimental Investigation of the Functional Hypothesis and Evolutionary Advantage of Stone-Tipped Spears

**DOI:** 10.1371/journal.pone.0104514

**Published:** 2014-08-27

**Authors:** Jayne Wilkins, Benjamin J. Schoville, Kyle S. Brown

**Affiliations:** 1 Institute of Human Origins, School of Human Evolution and Social Change, Arizona State University, Tempe, Arizona, United States of America; 2 Department of Archaeology, University of Cape Town, Cape Town, South Africa; University of Oxford, United Kingdom

## Abstract

Stone-tipped weapons were a significant innovation for Middle Pleistocene hominins. Hafted hunting technology represents the development of new cognitive and social learning mechanisms within the genus *Homo*, and may have provided a foraging advantage over simpler forms of hunting technology, such as a sharpened wooden spear. However, the nature of this foraging advantage has not been confirmed. Experimental studies and ethnographic reports provide conflicting results regarding the relative importance of the functional, economic, and social roles of hafted hunting technology. The controlled experiment reported here was designed to test the functional hypothesis for stone-tipped weapons using spears and ballistics gelatin. It differs from previous investigations of this type because it includes a quantitative analysis of wound track profiles and focuses specifically on hand-delivered spear technology. Our results do not support the hypothesis that tipped spears penetrate deeper than untipped spears. However, tipped spears create a significantly larger inner wound cavity that widens distally. This inner wound cavity is analogous to the permanent wound cavity in ballistics research, which is considered the key variable affecting the relative ‘stopping power’ or ‘killing power’ of a penetrating weapon. Tipped spears conferred a functional advantage to Middle Pleistocene hominins, potentially affecting the frequency and regularity of hunting success with important implications for human adaptation and life history.

## Introduction

Recent functional studies of Middle and Late Pleistocene stone tools highlight the importance of hafted hunting technology during the evolution of the human lineage [1]–[6]. It is often assumed that weapons with a stone tool hafted to the tip confer a foraging advantage over untipped sharpened wooden weapons. Numerous suggestions about what that advantage is include functional, economic, and social explanations. Few of these suggestions have been experimentally tested, despite the important implications this new innovation had on Middle Pleistocene hominins.

Spear technology dates to at least the early Middle Pleistocene. At Kathu Pan 1, South Africa, an assemblage of ∼500-thousand-year-old stone points exhibit multiple characteristics that indicate their use as spear tips, including but not limited to macrofractures diagnostic of weapon use [7], basal modifications consistent with hafting, and an edge damage distribution that is concentrated at the tip consistent with experimental studies of spear use [1]. A horse scapula recovered from Boxgrove, England, also dated to ∼500 ka, exhibits a semicircular perforation consistent with spear-aided hunting [8]. At Schöningen, Germany four wooden spears were recovered from sediments with exceptional preservation conditions dating to ∼400 ka [9]. These were originally interpreted as throwing spears, but the mode of delivery as either thrusting or throwing spears is unclear [10]–[12]. Middle Stone Age points from the Gademotta Formation, Ethiopia date to >279 ka and show microscopic impact features suggestive of their use as spear tips, perhaps for throwing spears, rather than thrusting spears [13], [14]. Based on use-wear traces that include impact fractures and microscopic linear impact traces, stone spear points were identified in Middle Paleolithic levels at Biache-Saint-Vaast, France dated to ∼250 ka [2]. At Sai Island, Sudan, a single point fragment from the Middle Sangoan unit (∼200–160 ka) exhibits evidence for hafting and impact damage and is argued to be a ‘projectile’ fragment [3].

In modern hunter-gatherers, spears are used in conjunction with other types of weapon systems that often include high velocity projectiles (i.e., atlatl and dart, bow and arrow). For that reason, spears sometimes play a minor role in hunting and warfare [15]. Humans did not always possess high velocity projectiles, however. The earliest evidence for high velocity weapons based on tool form (i.e., microlithic backed blades) comes from the South African Late Pleistocene ∼71 ka [16]. Backed blades dating to 65–60 ka exhibit wear and residue features consistent with hafting and impact [4], [5], [17]–[21]. The small, lightweight, and standardized nature of quartz backed pieces from Sibudu Cave supports the argument that they were used as arrowheads [22]. A small bone point may provide additional support for bow and arrow technology during this time [23]. High velocity projectiles have the advantage of increasing distance between the hunter and prey, which significantly reduces risk of injury and death for the hunter [10], [24]. Before the invention of high velocity projectiles in the Late Pleistocene, humans must have relied solely on close-proximity weapons such as spears for dispatching game, and this type of hunting technology would have been subjected to intensive selective pressure. Even throwing spears only have an effective range of ∼8 meters [24] and would have required hunters to put themselves in dangerous and difficult positions in order to dispatch large game.

Direct evidence for hunting technologies prior to ∼500 ka is lacking, despite evidence that hominins were regularly gaining primary access to meat by at least ∼780 ka [25]. Handaxes and other heavy-duty stone tool types may have been used to deliver deathly blows by throwing [26], [27], or by hand during the Early and Middle Pleistocene. Indirect evidence also suggests that hominins could have constructed sharpened wooden spears, perhaps similar to those recovered at Schöningen [9], through the Early and Middle Pleistocene. Residue and use-wear studies show that Acheulean tools were at least sometimes used for processing plant materials, including wood [28]–[30]. Denticulates and notches have been interpreted as woodworking tools [31] and these kinds of tools are found in Acheulean contexts. Humans have a derived morphology that enables endurance running and exhibited aspects of this morphology by the early Pleistocene [32], and may have used this advantage to chase prey to exhaustion during persistence hunting. Prior to the mid-Middle Pleistocene, hominins were probably employing a combination of hand-held or thrown stone tools, wooden spears and clubs, and endurance running to take down large game.

Hafting a stone tip to a wooden shaft was a significant innovation for Middle Pleistocene hominins and may represent the origin of new cognitive and social capacities within the human lineage. Part of human cognition is the ability to hold in attention multiple tasks and conduct goal-oriented behavior. The concept of ‘working memory’ has been used to highlight this capacity [33]–[36]. Evidence for hafted hunting technology in the Middle Pleistocene may indicate an enrichment of working memory capabilities compared to earlier periods. The manufacture of hafted technologies is one type of behavior that requires working memory because it requires the collection, preparation, and combination of different kinds of resources – wood, stone, and binding material. Another way to approach hominin cognition relevant to technological change is the concept of ‘constructive memory’. Ambrose [37] argues that this human capacity for imagining future scenarios and planning for them is more important than working memory, which is focused on immediate tasks. Hafting represents an advance in hominin constructive memory because it involves the completion of multiple subgoals (shaft manufacture, point manufacture, resin manufacture) and final assembly occurs later. It also represents a substantial amount of prior investment with the ultimate goal of securing game in the future. Boyd and Richerson [38] suggest that hafted spears represent cumulative culture. Hafted spears are the combination of multiple innovations to the lithic point and the shaft. A hafted spear is something unlikely to result solely from individual learning in the course of one individual's lifetime. Rather, social learning mechanisms that pass information through multiple generations are required to explain the regular manufacture of points and their use as armatures on spears.

Hafting stone tips is a costly behavior. It requires more time and effort to collect, prepare, and assemble hafted spears than to prepare a sharpened wooden spear. Stone tips are also prone to breakage and require more protection during transport [15] and they frequently break during use [39], [40], requiring maintenance and/or replacement. With respect to thrusting spears in particular, it could even be disadvantageous to have a stone tip, because its fragility could prohibit multiple thrusts and there are some ethnographic testimonies that support this concern [15]. The haft itself adds an additional element of costly risk; an imperfect haft may fail upon impact and interfere with penetration.

If hafting is costly, *why* was the stone-tipped spear innovation selected for in the Middle Pleistocene? A functional explanation for why the stone-tipped spear innovation was selected for – that stone-tipped spears are more effective hunting weapons than untipped spears – is the most intuitive one, but ethnographic and experimental research so far provides mixed support for this hypothesis. Whether hafted spears provide a functional advantage or not has important implications for evolutionary impact of this innovation.

## Background

Previous studies demonstrate that stone-tipped weapons are effective for dispatching large game. Hand-thrust spears hafted with Clovis point reproductions appeared to be effective when used on an elephant carcass, penetrating the skin, and sometimes penetrating at least 30 cm into the flesh; maximum penetration in this case was limited by the design of the foreshaft [41]. Penetration deeper than 20 cm is considered the lethal depth for large mammals [42]. Clovis reproductions used as atlatl darts also effectively penetrated elephant skin and caused what appeared to be fatal wounds [43]. Hunzicker [44] tested Folsom point reproductions as atlatl dart tips and found that 74% of shots penetrated more than 40 cm into cow ribcages.

Points do not need to be intensively shaped or bifacially-worked to be effective. An experimental comparison of weapons with bifacially-retouched and unretouched stone tips found that bifacially-retouched stone points penetrated slightly deeper into dog carcasses than unretouched stone points for both arrows and spears, but the difference was not significant [40]. Odell and Cowan (1986) did find that unretouched points were broken or lost more often than retouched points, and the difference was significant [40]. Shea et. al [45] found that unretouched and minimally retouched Levallois point replicas delivered as thrusting spears effectively penetrated goat carcass targets beyond 20 cm, and short, broad points were the most durable [45]. However, Levallois points are not durable when used as high velocity arrow tips [46]. Both unretouched and retouched Middle Stone Age point reproductions are also effective spear tips [1], [47].

Reviews of ethnographic and ethnohistorical studies provide equivocal evidence for the advantage of tipping weapons with stone. Ellis [15] cites examples from the literature of hunter-gatherer interviewees stating that stone tips cause more lethal wounds. Multiple explanations are given for lethality of these wounds, including that they are deeper, they are lacerated rather than punctured, and they bleed more. Other studies have mentioned the effect of tip breakage, which causes parts of the stone tip or even the whole tip to remain lodged in the wound cavity and cause more damage [15]. While those studies support a functional explanation for stone-tipped weapons, they are unspecific about exactly *why* they are more effective, and in some cases are based on ‘memory culture’ (i.e., the information was obtained from informants long after stone use had ceased). Ethnographic research also demonstrates that wood alone is the most common material for projectile points among societies that hunt. More than 64% of a sample of 59 ethnographic groups use projectiles made exclusively of wood [48]. There are also numerous hunter-gatherer groups who use only wooden projectile points, and they use them on a variety of prey types [48]. From the ethnographic and ethnohistorical literature alone, it is clear the costs of stone-tipped weaponry do not always outweigh the benefits, and that we do not know with confidence exactly what the benefits are.

There have been a few experimental studies with untipped controls designed to test the relative effectiveness of composite weapons. Guthrie [49] found that, compared to antler and bone-tipped darts propelled with a compound bow at a moose carcass, untipped wood darts exhibited a lower mean penetration depth at ∼14 cm. Different types of antler and bone-tipped darts penetrated to mean depths of between ∼21 and ∼28 cm. However, there are no statistics presented in that study to evaluate whether the difference between bone and antler or wood-tipped points is significant, and these results are not directly relevant to the effectiveness of stone tips.

Petillion et al. [50] present the results of an experiment comparing the performance characteristics of antler points with and without lithic inserts. These weapons were modelled after archaeological examples of Magdelenian dart tips that have a polished antler core with backed bladelets glued into notches that run along the point laterals. The Magdalenian reproductions were hand-propelled with the aid of an atlatl into complete young deer carcasses. Plain antler points exhibited a mean penetration depth of 15.5 cm, while points with lithic inserts penetrated a mean depth of 28.3 cm. The size of the effect is large and suggestive; however, the experimenters used six different weapon designs and did not control for total spear mass, spear velocity, identity of the thrower, or throwing fatigue, which would influence penetration depth. The authors report no significant correlations between these variables and penetration depth [50], but the small sample sizes for these different combinations of variables warrant concern about Type 2 error. Furthermore, these results are not directly relevant to the performance difference between wooden tips and stone tips.

Contrary to expectations, Holmberg [51] found that untipped weapons penetrated deeper into soft targets (straw bales) at lower energies and exhibit a stronger response to increased velocities than stone-tipped weapons. For complex animal targets with bone, skin, and fur, there is no significant difference in performance between weapon tips of different stone raw material or form [51]. Based on this evidence, Holmberg suggested that differences in tip type may have more to do with stylistic choices and local identity than functional performance [51]. However, there is significant difference in the damage area (determined by multiplying point width by penetration depth) between untipped spears and stone-tipped spears for all target types [51].

Waguespack et al. [48] compared the penetration depths of untipped and stone-tipped arrows and found that stone-tipped weapons penetrated significantly deeper than untipped weapons into ballistic gelatin targets. However, Waguespack et al. [48] conclude that an exclusively functional explanation for tipping weapons with stone seems unlikely, because the difference in mean penetration depth was small (∼2 cm) and both weapon types penetrated to >20 cm. They suggest that economic or social advantages may provide a better explanation for why many ethnographic groups tip their arrows with stone points.

In an experiment designed to look at how different weapon tip morphologies influence wound characteristics, Anderson [52] found that untipped weapons penetrated deeper into ballistic gelatin than many of the tipped weapon types, but that some tipped designs (i.e., Cumberland points) did penetrate substantially deeper (>10 cm) than the untipped control. This finding suggests that some stone tips can substantially increase weapon effectiveness, contrary to the findings of Waguespack et al. [48], and that point form does influence penetration ability, contrary to the findings of Holmberg [51]. Anderson [52] also looked at the width of the wound track, and found that tipped weapons create wider wounds, on average. However, the differences in mean wound depth and width between the different weapon types were not subjected to statistical analysis by Anderson [52], and it is not possible to evaluate whether the observed patterns are significant with the published data. Furthermore, the tips used for the experiment were plastic replicas of stone points, and it is unknown to what extent the use of this material may have influenced the experiment outcome.

Salem and Churchill [53] recently conducted an experiment comparing three types of arrows: symmetrical tipped, asymmetrical tipped, and untipped arrows. They shot gelatin targets and found that wooden arrows penetrated slightly but significantly deeper than stone-tipped arrows. Using point tip cross-sectional area (TCSA) and penetration depth they also calculated the ‘volume of tissue disrupted’. Despite resulting in lower penetration depths, tipped arrows disrupted a significantly higher volume of tissue compared to untipped arrows based on this calculation. The results of Salem and Churchill [53] support a functional explanation for tipping projectile weapons with stone.

Wound ballistics research uses gelatin targets to investigate wound track profiles, which demonstrate how different types of weapons damage flesh [54], [55]. There are two parts of the ballistics wound track; the *permanent cavity* and the *temporary cavity*. The permanent cavity represents the track of the bullet, where the bullet contacted the flesh or gelatin and displaced it through the mechanism of crushing. Bullets designed for increased penetration, such as full-metal jacket rifle bullets, create long, narrow permanent cavities. Bullets known for having increased “stopping power” or “killing power”, such as fragmentation bullets, are designed to expand, slow down, and then fragment in order to create the biggest permanent cavity possible. The temporary cavity, which extends beyond the permanent cavity, is created by dispersed energy due to impact that displaces the flesh or gelatin, causing it to stretch. The type of damage experienced by this stretching is a kind of ‘blunt trauma’, which often manifests physically as a bruise. Because elastic tissues like muscle, bowel walls, and lungs, are fairly resistant to damage from stretching, the size of the temporary cavity is not seen as a key contributor to the lethality of the wound [54].

We present the result of a controlled experiment designed to test the functional hypothesis for stone-tipped weapons using spear replications and ballistics gelatin. The study differs from previous investigations of this type because (1) it includes a quantitative analysis of wound track profiles, expanding the types of variables used to assess weapon performance, (2) it statistically assesses the observed differences in these variables, and (3) it focuses specifically on hand-delivered spear technology, which is the main weapon-delivery system relevant to understanding human technological evolution during the Middle Pleistocene of Africa and Eurasia.

## Materials and Methods

Two standardized sets of spears (tipped and untipped), a calibrated crossbow that could deliver a consistent draw force, and ballistic gelatin targets were constructed to carry out the experiments.

### Spears

The experimental spears were modeled after the published reports and illustrations of the Schöningen spears [9]. Each of the 10 experimental spears was manufactured the same way and from the same materials, until the final stage in which lithic points were hafted onto 5 of the spears. The spears were constructed from 1 5/16 inch (3.33 cm) diameter poplar dowels. This diameter was chosen because mean maximum diameter of the three Schöningen spears is 1.48 inches, or 3.77 cm [9]. The Schöningen spears were manufactured from spruce, but poplar was chosen as a suitable material for our spearing experiments because it as a soft wood with a Janka hardness of ∼2.0, and in those respects, is similar to spruce.

**Figure 1:**
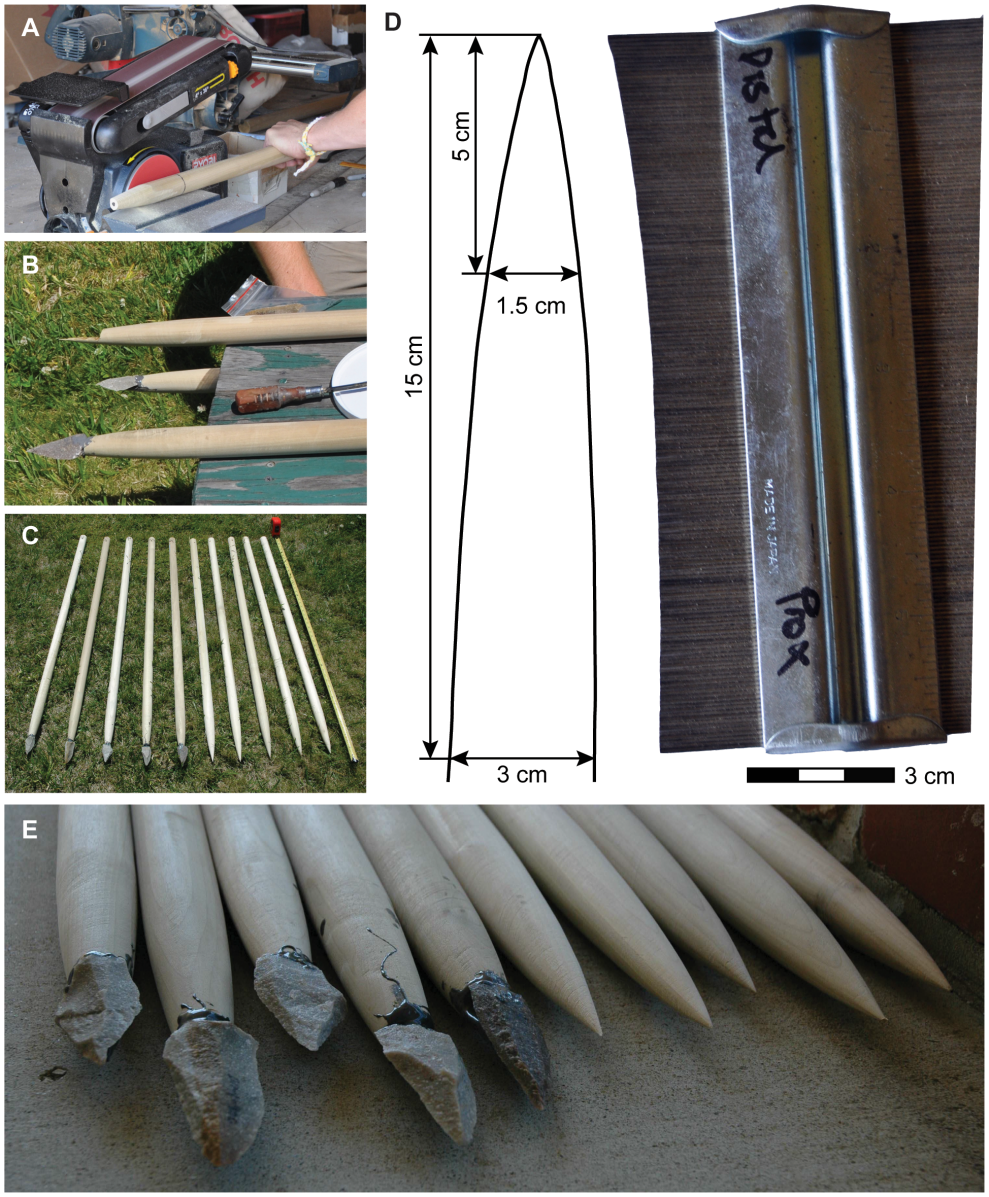
Manufacturing the experimental spears. A. Using disc sander to shape distal end of spear. B. L-notch used for hafting stone tools to half of the spears. C. Two sets of spears were manufactured, 5 tipped, 5 untipped. D. The shape of the distal end was constant between all 10 spears; a contour gauge was used to ensure consistency. E. Commercial epoxy was used as the binding agent to attach the quartzite stone tips.

The tip of each spear was sanded to a point using a 100 grit disc sander (Figure 1A). The shape of the tip was modeled after Spear II from Schöningen [9]. To maintain consistency between the spears, only the last 30 cm of the tips were sanded. The tips were shaped to the following specifications; a diameter of 3 cm at 15 cm from the tip and diameter of 1.5 cm at 5 cm from the tip (Figure 1D). The convexity of the spear tip was checked and made consistent using a contour gauge (Figure 1D). A hole was drilled through the dowel near the base for drawing the spear back with the calibrated crossbow.

A stone point was hafted to half of the experimental spears (Figure 1B, C, and E). These points were knapped by KSB on quartzite collected from the South coast of South Africa. Four of the points were manufactured on quartzite collected at Cape St. Blaize (lat −34.18565167°, long 22.15974667°) and one on quartzite collected near Fransmanshoek (lat −34.30364°, long 21.931205°). No permission was required for the small amount of stone collected at Cape St. Blaize. Permission for collection at Fransmanshoek was granted by the Fransmanshoek Conservancy. The mean technological length and tip cross sectional area (TCSA) for the experimental points (Table S1 in File S1) are similar to mean values for points recovered from MSA and MP archaeological contexts [1], [10], [56], [57].

The points were hafted onto the tips of five of the shaped spears by creating a tapered L-notch at the tip of the spear using an angle grinder (Figure 1B). The length of each L-notch was 2/3 the length of the hafted point. A commercial epoxy (JB Weld brand) was used to bond the points. An artificial binding agent was chosen in order to keep the haft strength as consistent as possible so that variations in the effectiveness of the haft did not influence the experimental results.

Because mass influences armature velocity [58], it was important that mass did not differ significantly between the untipped and tipped spears in our experiment. We ensured this by taking the mass of each completed spear and conducting a t-test on the two group means. The lithic tips did not significantly alter the mass of the complete spear. The most variability in mass was introduced by the internal characteristics of the wooden dowels themselves. One spear (13-U2) originally had a very high mass, so some material was removed from the shaft using an angle grinder to make the mass of the spear consistent with the others. Summary statistics for the complete spears used in the experiment are summarized in Table S2 in File S1. An unpaired t-test shows that the mean masses between the two groups (untipped mean = 585 g, tipped mean = 556 g) are not significantly different (t = 1.447, df = 8, *p* = 0.186).

### Calibrated crossbow

**Figure 2:**
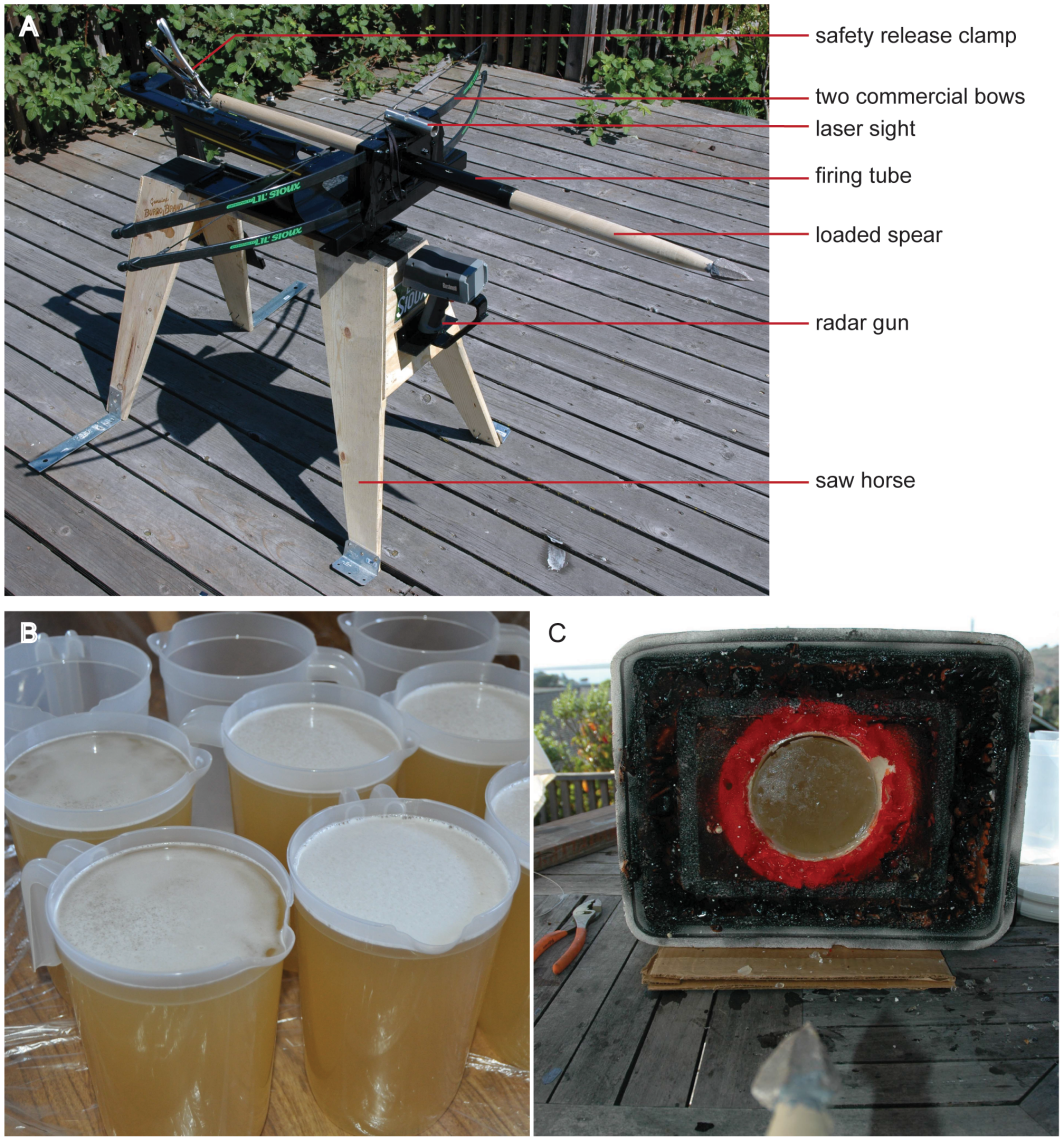
The calibrated crossbow and gelatin targets. A. The calibrated crossbow consists of two commercial bows mounted crosswise. A digital scale was used to draw the bow with a consistent 20 kg draw force. B. Fifty gelatin targets were manufactured using published ballistic standards. Plastic pictures were used as the molds. C. During shooting, the molds were secured in place with a foam target.

A calibrated crossbow was constructed [45] so that each shot could be delivered with a consistent draw force that simulates thrusting spears. Two commercial bows (Lil' Sioux Jr. brand) were mounted crosswise onto a welded metal plate with pivoting vertical and horizontal angle adjustments. The metal plate was then attached to a locking track-way that allowed forward and backward adjustment. The crossbow assembly was anchored to a saw horse bolted to a wooden deck for safety (Figure 2A). A firing tube and laser pointer, also mounted onto the saw horse, helped direct the spears at the target. For each shot, the spear was aligned towards the center of the gelatin target using the laser pointer, and the horizontal track-way adjusted so that when the bows were drawn, the tip of the spear was 43 cm from the target. This distance allowed for accurate shot placement into the center of the target with negligible external effects (e.g., wind). This method was accurate, and no spears missed the center of the gelatin target. A digital spring scale was used to draw with a force of 20 kg. The velocity generated (8.9–9.4 m/s) is between estimates from knife stabbing at 5.8 m/s [59] and throwing spears at 17–27 m/s [60] where thrusting spear velocity might reasonably be expected. For these experiments, it is less important to replicate exact thrusting spear parameters that will vary based on the weight of the spear, strength of the hunter and other variables, but essential that each spear was subjected to the same draw force and traveled the same distance before hitting the gelatin target.

### Gelatin targets

Gelatin preparation instructions followed those of Jussila [61], a standardized preparation method that produces homogenous gelatin blocks of good quality. The gelatin (250A bloom Traileze brand) was purchased from an equine supply store. Each batch was made using 2.0 lbs. (907.2 g) of gelatin powder first mixed with 4 (3.79 L) quarts of cool tap water (65°F) using an electric paint mixer. Once thoroughly mixed, 4 quarts of warm water (70–75°F) was slowly added to the mixture and stirred for approximately 7–10 minutes until the liquid was clear and any large lumps had dissolved. Any remaining small lumps were removed with a strainer. The liquid was poured into 2.5 quart cylindrical plastic molds with a depth of 21.3 cm and a maximum diameter of 14.0 cm (Figure 2B). If any foam formed on the surface when it was poured into the mold, the foam was scooped off. The mold was covered in plastic wrap and left to cool in ice tubs and in a refrigerator at approximately 4°C for 20–24 hours.

The molded gelatin was secured within a styrofoam target for the experiments (Figure 2C). The target was designed so in the event that the spear penetrated past the end of the gelatin mold into the styrofoam backing, a penetration depth reading could still be taken.

### Shooting experiments

Each spear was shot into five gelatin targets. Untipped and tipped spears were used alternatively in the following sequence: 13-T1, 13-U1, 13-T2, 13-U2, 13-T3, 13-U3…etc. The two groups were used alternatively in this manner so that any variables related to the time of day (i.e. temperature) or wear on the equipment were spread evenly between the two groups. The velocity of each shot was measured using a Bushnell Speedster III radar gun. Penetration depth was recorded by marking the shaft of the spear while it was still penetrating the gelatin and then measuring from that mark to the tip once the spear was extracted. Withdraw force was measured while the spear was being extracted using a digital hanging scale. Sometimes extracting the spear caused gelatin to be pulled out from the wound track, and this material, if present, was weighed following each shot.

### Photographing and analyzing the gelatin targets

**Figure 3:**
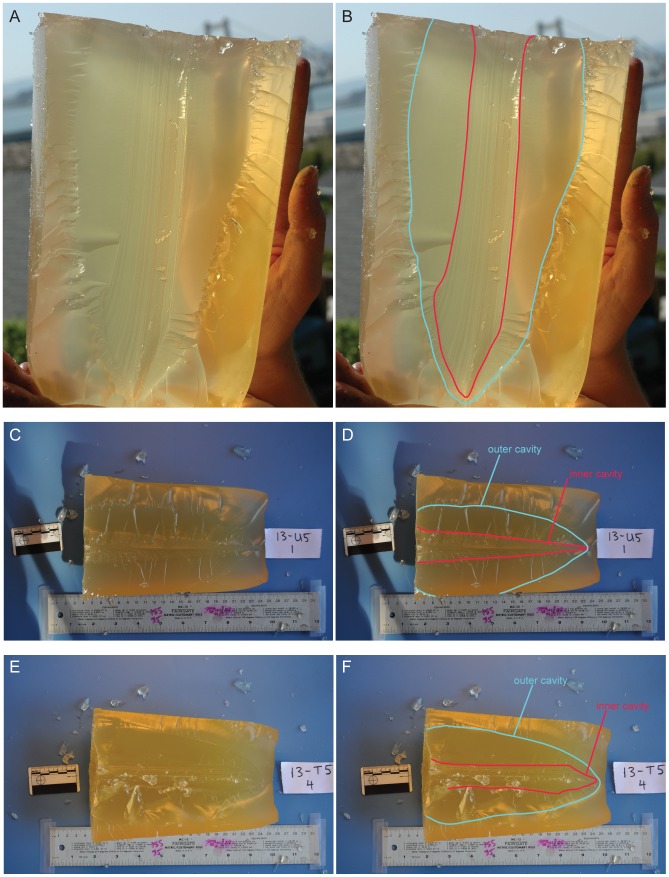
Wound track profiles in gelatin targets after being shot. A. Tipped spear gelatin half showing wound track details. B. Same gelatin in A with traced outlines of wound track features. C. Untipped gelatin half, spear 13-U5, shot 1. D. Same gelatin in C with traced outlines of wound track features. The inner cavity is represented by the red line and the outer cavity is represented by the blue line. E. Tipped gelatin half, spear 13-T5, shot 4. F. Same gelatin in E with traced outlines of wound track features.

After being shot, the gelatin targets were easily pulled apart to expose the wound track (Figure 3). Generally, the gelatin split into two halves, and each of these sections were photographed with a scale (Figure 3C–F). The scale was used to rectify the photographs in ArcMap 10.1. The polygon tool in ArcMap 10.1 was used to outline the wound track and the ‘calculate geometry’ function was used to calculate area and perimeter of the polygons (Figure S1 in File S1).

Each wound track consisted of two parts, what we are calling the ‘inner’ and ‘outer’ cavities (Figure 3D, E), which are analogous to the ‘permanent’ and ‘temporary’ cavities in bullet ballistics [54]. In our experiments, the outer cavities sometimes extended past the limits of the gelatin mold. In these cases, we were unable to trace the outer cavity outlines. Future experiments will use larger gelatin targets to avoid this issue. In order to maximize our analyzable sample size from these experiments, each wound track was further divided longitudinally into an upper and lower half (Figure S1 in File S1), so that we could still acquire data if only one of these halves was complete. For this reason, the absolute values presented for area and perimeter represent one-quarter of each wound track feature.

The shape of the resulting one-quarter inner and outer cavity was analyzed using geometric morphometrics following the thin plane spline protocols. Each GIS polygon was converted to a curve within TPSDig2 [62]. Each curve was then systematically converted into 30 approximately equidistant landmarks using the curve resampling method in TPSDig2. The first landmark (the most proximal wound entry point in the gelatin) and the last landmark (the distal most extent of the wound) are considered homologous landmarks (Type 1) across wound tracks, and the 28 landmarks between these are treated as sliding semi-landmarks. A Generalized Procrustes analysis was performed in TPSRelw [62] using thin-plate spline methods. This removes differences in photo orientation and size of the wound tracks, leaving only shape variability. A relative warps, or principal components, analysis on the shape variables was performed to evaluate the factors underlying shape variability in the tipped and untipped wound tracks. The resulting shape variables were analyzed using non-parametric multivariate analysis of variance to compare mean shapes.

## Results

**Figure 4:**
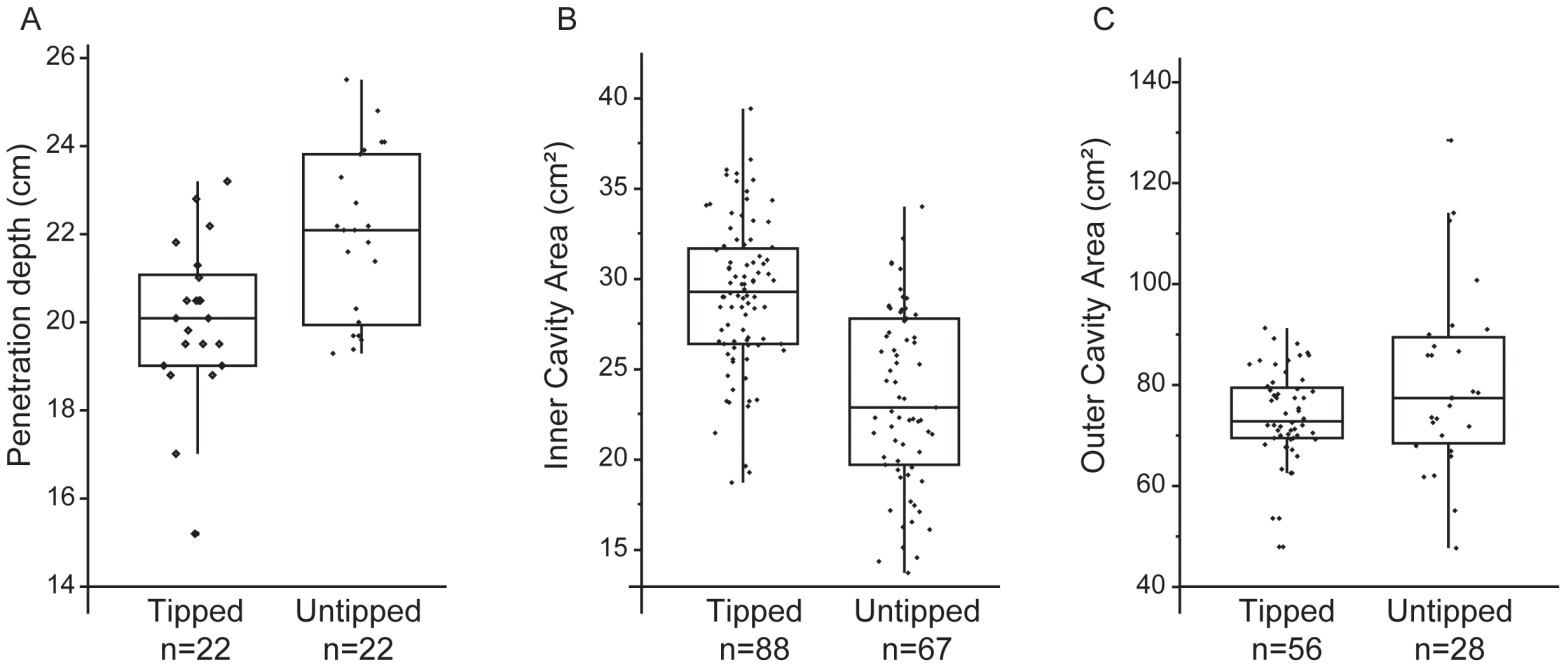
Box plot comparison of tipped and untipped performance characteristics. A. Penetration depth (cm). B. Inner cavity area (cm^2^). C. Outer cavity area (cm^2^). Each of these variables is significantly different between tipped and untipped spears based on t-tests (see text).

Figure 4 presents a comparison between tipped and untipped spears for penetration depth and wound track size. Additional tables are available in File S1 of the online Supplementary Information. Raw data, including those from two shots which were excluded from analyses because of equipment failure, are available in Appendix S1 of File S1. Wound track images, shapefiles, and tps files are available in File S2.

### Velocity

We measured velocity using a radar gun (Table S3 in File S1) to ensure that slight variations in mass between the spears due to random variation in the characteristics of the wooden dowels did not affect the velocity at which they were contacting the target. We found no significant difference between the tipped and untipped spears with respect to velocity (*t* = 1.7385, df = 21, *p* = 0.0968).

### Penetration depth

Untipped spears had a mean penetration depth greater than tipped spears (Figure 4A, Table S4 in File S1) and this difference is significant (*t* = 3.5078, df = 42, *p* = 0.001). Tipped spears had a mean penetration depth of 20.0 cm and untipped spears had a mean penetration depth of 22.0 cm. Both of these penetration depths are consistent with that recommended for hunting large game. Our results do not support the hypothesis that adding a stone tip to the end of a thrusting spear improves its penetration ability.

### Extraction

We looked at two other variables related to the effect of extracting the spears and these data showed no significant difference between the two groups. One of these variables was pull-out force (Table S5 in File S1) – we used a scale to measure the maximum kilograms draw force it took to extract the spear from the gelatin. It has been suggested that adding a stone essentially creates barbs along the spear laterals that could prohibit multiple thrusts and result in a disadvantage [15], or could beneficially cause additional damage as it is extracted. We expected the tipped spears to demonstrate greater pull-out forces on average than untipped spears, but there was no significant difference (*t* = 0.1418, df = 40, *p* = 0.888).

Sometimes, but quite rarely, some gelatin detritus would unintentionally be extracted from the wound when the spear was pulled out. We took the mass of this detritus after each extraction (Table S6 in File S1). There is no significant difference between the amount of detritus extracted for tipped and untipped spears (*t* = 1.5572, df = 43, *p* = 0.127). A higher frequency of tipped spears resulted in some extracted detritus. In other words, the number of non-zero values is higher for tipped spears than untipped spears, but the difference is not significant (Fisher's exact test, *p* = 0.135).

### Wound track size

The results of the wound track size analysis are presented in Figure 4B and 4C and Table S7 in File S1. The mean area of the inner cavity quarter is significantly larger for the tipped spears than the untipped spears (*t* = 8.0020, df = 153, p<0.001, Figure 4B). Tipped spears create inner cavities that are 24.8% larger than those of untipped spears. The mean area of the outer wound track quarter is significantly larger for the untipped spears than the tipped spears (*t* = 2.1005, df = 82, *p* = 0.039, Figure 4B). There are no significant differences between the mean inner perimeter (*t* = 0.4143, df = 153, *p* = 0.453) or the mean outer perimeter (*t* = 0.5119, df = 82, *p* = 0.610) of the wound track quarters. To summarize, the wound track area differs significantly between tipped and untipped spears. The tipped spears created significantly larger inner wound tracks, which are associated with incapacitating tissue damage. Untipped spears create significantly larger outer cavities, which are generally considered analogous to the area of stretched and bruised tissue surrounding a wound.

### Wound track shape

**Figure 5:**
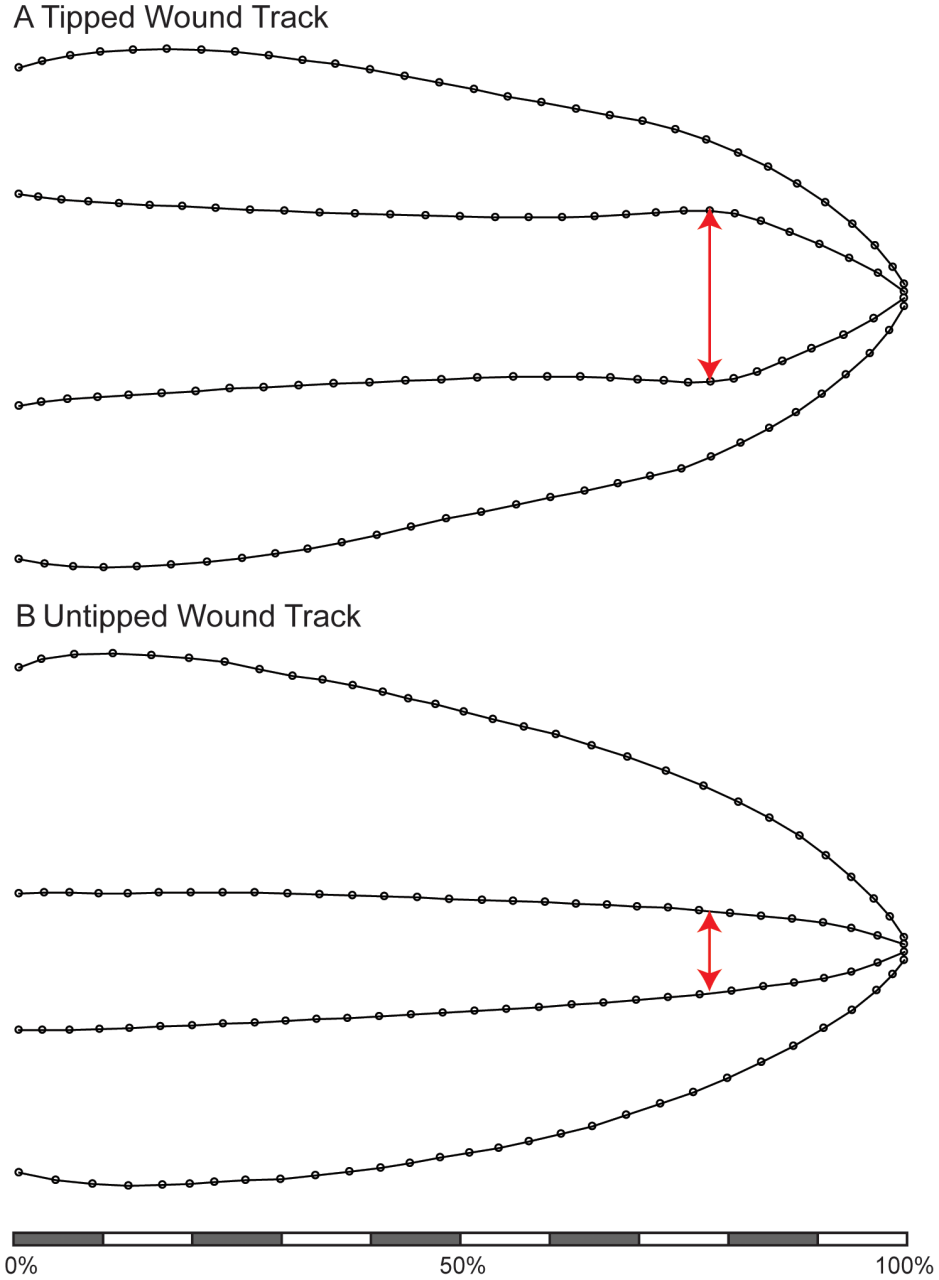
Consensus shape of gelatin wound track profiles based on geometric morphometrics for A) tipped and B) untipped spears. Scale represents relative percentage of total wound track length. Red arrow highlights location at about 80% of wound length where there is a widening of the inner cavity in tipped spears. That same relative location is narrow in tipped spears.

**Figure 6:**
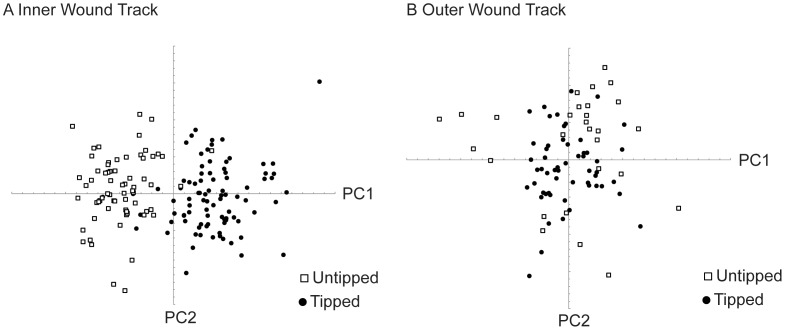
Scatter plots of the first two principal components from the geometric morphometric relative warps analysis of the tipped and untipped wound track shape. A) The tipped inner wound track shape is significantly different from the untipped inner wound track shape (PC1 explains 35% and PC2 explains 27% of variance). B) The outer wound track shapes are not significantly different between tipped and untipped spears (PC1 explains 52% and PC2 explains 23% of variance).

The mean shape of the inner and outer cavities for tipped and untipped spears are shown in Figure 5. The inner cavity exhibits a widening near the distal part of the wound track for tipped spears, at about 80% of the wound track length (Figure 5A). That same location is much narrower for untipped spears (Figure 5B). The difference in mean shape for the inner cavity is significant (*F* = 49.5, *p*<0.001, Figure 6A). The mean shape of the outer cavities are visually similar for tipped and untipped spears (Figure 5A–B), and the mean shapes are not significantly different (*F* = 2.085, *p* = 0.093, Figure 6B).

## Discussion

Our controlled experiment compared the performance characteristics of ten spears that were nearly identical in every respect except that half of them had a quartzite point hafted to their tips. The masses of the two sets of spears did not differ significantly. They were propelled at standardized ballistic gelatin targets using a calibrated crossbow and with a consistent draw force. The velocity of the spears as they hit the targets was not significantly different between the two groups. The tipped spears did not penetrate deeper than the untipped spears, but they did create larger inner wound cavities that were distally wide.

It has been suggested that stone-tipped armatures are more effective for dispatching game because they increase penetration depth. Our results do not support this hypothesis. In contrast, the untipped sample showed a significantly greater mean penetration depth than the tipped samples. The difference between the means is small, only 2 cm, but the pattern is robust; tipped spears are responsible for the 6 smallest penetration depth observations, and untipped spears for the 7 largest penetration depth observations. Based on these results, if penetration depth was the primary goal of a hunter using a thrusting spear, it may be more advantageous to use an untipped spear than a tipped spear.

Our results contrast those of Waguespack et al. [48], who found that, on average, stone-tipped arrows penetrated ∼2 cm deeper than untipped arrows. They found this relationship to be true for both types of targets that they used (one uncovered gelatin target and one covered in hide). Besides the fact that the weapon delivery systems differed between our experiments, there are a few other potential explanations for these contrasting results. First, differences in the stone point characteristics may explain the conflicting results. Waguespack et al. [48] used small bifacially-worked arrowheads and some experimental work has suggested that bifacial points penetrate deeper than unretouched stone points [40]. Other factors such as shape, size, and tip angle also seem to effect penetration ability [52]. Second, because they were interested specifically in high velocity projectile technologies, Waguespack et al. [48] used a 60 lbs. (27.2 kg) draw force, which is greater than the draw force used in our thrusting spear experiment. More work is required to determine how different velocities and different weapon delivery systems influence weapon performance. Third, the tipped arrows in the Waguespack et al. experiment have a higher mass than the untipped arrows [48]. They found no correlation between mass and penetration depth, so they argue that it is unlikely that the difference in mass explains the difference in penetration depth. However, for spears our results do show a weak but significant positive correlation between mass and penetration depth (Spearman's rank correlation coefficient, *ρ* = 0.315, *p* = 0.033).

The penetration results of this experiment are consistent with those of Holmberg [51], who found that untipped weapons penetrated deeper into soft targets (i.e. hay bales). However, Holmberg [51]) used different kinds of targets in his set of experiments, and ‘complex’ targets that consisted of various types of prey parts yielded different results. The results reported here are inconsistent with the those reported by Petillion et al [50], who reported that antler arrow points with stone inserts penetrated significantly deeper into complete deer carcasses than antler arrow points without stone inserts. The difference could be related to the kind of target used. Gelatin is an imperfect medium for assessing penetration depth of arrows and spears because it interacts differently with arrows and spears than it does with bullets [63], and lacks proxies for the fur, hide, fat, and bone that characterizes natural animal targets. Nonetheless, gelatin provides a standardized and consistent target that limits the number of factors affecting weapon performance, in contrast to more natural targets that show higher degrees of intra and inter-target variability.

Future work will utilize complex targets that are as standardized as the homogenous gelatin targets used in this experiment, by incorporating synthetic bone insertions and hide coverings. There is reason to doubt that hide will exert a great effect; in the Waguespack et al. study [48], a caribou hide covering was used in half of the experimental shots, and the hide equally affected both the tipped and the untipped weapons with respect to penetration depth.

The benefit of using homogenous gelatin targets is the ability to conduct a wound track profile analysis. Based on our wound track profile analysis, tipped spears create a significantly larger inner wound track than untipped spears. This inner wound track is analogous to the permanent cavity of bullet wounds, because it represents the debilitating tissue damage caused by direct contact with the penetrating object. Bullets that create expansive permanent cavities, such as hollow point and soft point bullets are known for having increased ‘stopping power’ or ‘killing power’ over bullets designed to maximize penetration. Other researchers have used proxies of wound track size to address the effectiveness of tipped vs. untipped weapons, and our results are consistent with theirs. Holmberg [51] determined ‘damage area’ by multiplying point width by penetration depth, and found that tipped weapons showed significantly higher values than untipped weapons. Salem and Churchill [53] found the same relationship by calculating damage volume as the product of penetration depth and tip cross-sectional area. Anderson [52] measured the width of the wound track, and found that tipped weapons create wider wounds, on average, but statistical results are not presented in that study. Our result is based on direct analyses of wound track profiles and provides robust quantitative and statistical support to the hypothesis that a stone-tipped spear provides a functional advantage over an untipped spear because it creates a larger wound.

The outer wound track is slightly larger for untipped spears compared to tipped spears. In ballistics research, the outer wound track is generally associated with bruising and less debilitating tissue damage [54]. An increased area of bruising for untipped spears may be due to the fact that untipped spears displace flesh primarily through pressure as opposed to cutting. Modern hunters are advised to remove bruised tissue when inspecting meat cuts, because bruised meat has a strong, gamey flavor [64]. For that reason, the smaller size of the outer wound track for tipped spears may actually provide a slight, but additional functional advantage.

Tipped spears create wounds with inner cavities that are a different shape than those created by untipped spears. Tipped inner cavities exhibit a widening at about 80% of the wound track length, at depths that are more likely to result in fatal damage to major organs and blood vessels.

Variables related to extraction showed no significant difference between tipped and untipped spears. There was no significant difference in the draw force required to extract the weapon from the gelatin and there was no difference in the mean amount of gelatin detritus that resulted from the extraction. Our results do not support the assertion that tipped spears are more difficult to retrieve and cause more damage during their extraction than untipped spears. This potential characteristic of tipped weapons can be considered both disadvantageous [15] and advantageous. Trauma surgeons are warned that barbed arrows cause more complications than arrows with target points because of risk of extensive damage when retrieved [65]. We hypothesize that a more complicated target, and especially a target that continued to move after being shot, would influence extraction in a way that our experiment was not designed to test.

We provide evidence that the evolutionary advantage of tipping a spear with stone has a functional explanation. A larger wound track translates to more tissue damage, an increased probability of hitting the heart, lungs, and/or major blood vessels, and an increased probability of incapacitating prey. Thus, the stone-tipped spear innovation may have significantly impacted the evolution of human life history and cooperative behavior. Fastening spears with stone tips is a strategy for reducing the risk of an unsuccessful hunt and providing more reliable access to meat. Because one strike with a stone-tipped spear has a higher probability of success, stone-tipped spears reduce the need for prolonged proximity to dangerous prey compared to untipped spears. Regular use of this new technology could have reduced adult mortality, increased average adult lifespan, increased daily return rates of large, high-quality food packages, and decreased daily nutritional variance. These effects may have changed the amount and regularity of resources adults can contribute to dependents, with important implications for human life history. An increased juvenile period, higher female fertility, and pair-bonded cooperative breeding all may be explained in part by higher rates and reduced variability in successful resource capture among hunter-gatherers [66], [67]. Stone-tipped spears may have also influenced the nature of inter and intra-group interaction; other humans may have at least sometimes been the target of these weapons. Computer simulations suggest that weapon use may be linked to human cooperation [68]. Agents with extra-somatic weapons are more likely to cooperate with each other than agents without extra-somatic weapons, in part because of the increased risk of lethality when agents choose to defect [68].

New archaeological evidence for stone-tipped spears in the mid-Middle Pleistocene [1], [2], [13] indicate an early chronology for a technology-dependent hunting adaptation with hafted tools. The first appearance of this adaptation by at least ∼500 ka at Kathu Pan 1 [1] predates the genetic divergence of the human and Neanderthal lineages at ∼400 ka [69], and is consistent with parallel archaeological evidence for hafted hunting technology used by the human lineage in Africa through the Middle Stone Age [3], [4], [10], [70]–[73] and the Neanderthal lineage in Eurasia through the Middle Paleolithic [2], [6], [10], [56], [74]. Currently, there are few other localities in Africa reliably dated to between ∼500 and 280 ka [75]. Like Kathu Pan 1, the assemblages between 509 ka and 284 ka at the Kapthurin sites contain artifacts typical of the Middle Stone Age, including points [76]–[78]. At Gademotta, sediments dated to >280 ka also have points interpreted as weapon tips [13]. The points from Gademotta have microscopic features that suggest they experienced impact at high velocities and may have been used as throwing spears. One might expect hominins to have used stone-tipped thrusting spears before stone-tipped throwing spears, which may require special engineering to optimize their aerodynamics.

Both humans and Neanderthals inherited the cognitive and psychological structures that enabled the construction and use of hafted spear technology, and hafted spear technology may have similarly influenced daily foraging returns, life history, and cooperation in the two lineages. However, only the human lineage took hafted hunting technology to the next level with the invention and spread of high velocity, long-distance projectile weapons [4], [5], [10], [22], [79]. The impetus for this kind of technological ratcheting during the Late Pleistocene for humans only is not well understood. The challenge remains to explain why the human lineage in Africa experienced such a radically different trajectory compared to the Neanderthal lineage in Eurasia with respect to weapon technology, despite starting with similar cognitive, social, and technological adaptations. Human niche widening in African refugia during Late Pleistocene arid periods is one potential explanation [79]. Population size may have also played a role; large population sizes are required to successfully transmit complex technological information through multiple generations [80]. Furthermore, only the human lineage developed an elaborate and exaggerated dependence on symbolism and prosocial behaviors [67], with roots for these behaviors expressed archaeologically in Africa during the Late Pleistocene [81]–[86]. Inter-species differences in these respects could explain why one species invented high velocity, long-distance projectile weapons and the other did not.

## Supporting Information

File S1 **Supplementary tables, figures, and appendices.**
(DOCX)

File S2 **Wound track images, shapefiles, and tps files.**
(ZIP)
