# Supplementary material for: An Experimental Investigation of the Functional Hypothesis and Evolutionary Advantage of Stone-Tipped Spears
Source: PLoS One. 2014 Aug 27;9(8):e104514. doi: 10.1371/journal.pone.0104514 (PMC4146534; doi:10.1371/journal.pone.0104514)
Supplement: File S1 — Supplementary tables, figures, and appendices. (DOCX) [file pone.0104514.s001.docx]

**Supplementary Information for: An Experimental Investigation of the Functional Hypothesis and Evolutionary Advantage of Stone-Tipped Spears**

Jayne Wilkins^1*^, Benjamin J. Schoville^1^, Kyle S. Brown^2^

^1^Institute of Human Origins, School of Human Evolution and Social Change, Arizona State University, Tempe AZ, U.S.A.

^2^Department of Archaeology, University of Cape Town, Cape Town, South Africa

*corresponding author ([jayne.wilkins@asu.edu](mailto:jayne.wilkins@asu.edu))

**Table S1.** Summary statistics of quartzite points used to tip experimental spears.

| **Spear Number** | **Technological Length (mm)** | **Max Width (mm)** | **Max Thickness (mm)** | **Mass (g)** | **TCSA** | **Material** |
| --- | --- | --- | --- | --- | --- | --- |
| 13-T1 | 69 | 35 | 13 | 23 | 225 | Quartzite (Fransmanshoek) |
| 13-T2 | 68 | 32 | 12 | 23 | 190 | Quartzite (Cape St. Blaize) |
| 13-T3 | 61 | 36 | 8 | 17 | 142 | Quartzite (Cape St. Blaize) |
| 13-T4 | 81 | 33 | 15 | 31 | 244 | Quartzite (Cape St. Blaize) |
| 13-T5 | 66 | 34 | 15 | 26 | 256 | Quartzite (Cape St. Blaize) |
| **Mean** | **69** | **34** | **13** | **24** | **211** |  |
| **Min** | **61** | **32** | **8** | **17** | **142** |  |
| **Max** | **81** | **36** | **15** | **31** | **256** |  |
| **SD** | **7** | **2** | **3** | **5** | **46** |  |

**Table S2.** Summary statistics for complete experimental spears.

| **Spear Number** | **Spear Type** | **Mass (g)** | **Length (cm)** |
| --- | --- | --- | --- |
| 13-U1 | Untipped | 630 | 120.7 |
| 13-U2 | Untipped | 619 | 121.3 |
| 13-U3 | Untipped | 526 | 121.4 |
| 13-U4 | Untipped | 565 | 121.6 |
| 13-U5 | Untipped | 583 | 121.0 |
| 13-T1 | Tipped | 551 | 123.8 |
| 13-T2 | Tipped | 556 | 124.1 |
| 13-T3 | Tipped | 547 | 123.2 |
| 13-T4 | Tipped | 578 | 124.1 |
| 13-T5 | Tipped | 549 | 122.6 |
| **Mean** |  | **570** | **122.4** |
| **Min** |  | **526** | **120.7** |
| **Max** |  | **630** | **124.1** |
| **SD** |  | **33** | **1.3** |

**Table S3.** Comparison of velocity (m/s) between tipped and untipped thrusting spear replicates. The radar gun failed to record velocity for a number of shots, which is why the N values are much lower than the number of shots taken.

|  | **Mean** | **95% CI** | **Min** | **Max** | **SD** | **N** |
| --- | --- | --- | --- | --- | --- | --- |
| Tipped | 9.4 | 9.0 - 9.8 | 8.3 | 10.3 | 0.71 | 13 |
| Untipped | 8.9 | 8.5 - 9.4 | 7.8 | 9.7 | 0.62 | 10 |

**Table S4.** Comparison of penetration depth (cm) between stone-tipped and untipped thrusting spear replicates into ballistics gelatin.

|  | **Mean** | **95% CI** | **Min** | **Max** | **SD** | **N** |
| --- | --- | --- | --- | --- | --- | --- |
| Tipped | 20.0 | 19.2 to 20.8 | 15.2 | 23.2 | 1.8 | 22 |
| Untipped | 22.0 | 21.1 to 22.8 | 19.3 | 25.5 | 1.9 | 22 |

**Table S5.** Comparison of the pull-out force (kg) required to extract stone-tipped and untipped thrusting spear replicates from ballistics gelatin.

|  | **Mean** | **95% CI** | **Min** | **Max** | **SD** | **N** |
| --- | --- | --- | --- | --- | --- | --- |
| Tipped | 6.5 | 5.974 to 7.054 | 4.3 | 8.5 | 1.2 | 21 |
| Untipped | 6.4 | 5.629 to 7.266 | 3.8 | 11.1 | 1.8 | 21 |

**Table S6.** Comparison of gelatin detritus (g) removed from target when stone-tipped and untipped thrusting spear replicates were extracted from ballistics gelatin.

|  | **Mean** | **95% CI** | **Min** | **Max** | **SD** | **N** | **N (non-zero values)** |
| --- | --- | --- | --- | --- | --- | --- | --- |
| Tipped | 0.3 | -0.034 to 0.607 | 0.0 | 2.7 | 0.7 | 22 | 6 |
| Untipped | 0.0 | -0.038 to 0.125 | 0.0 | 0.9 | 0.2 | 23 | 2 |

**Table S7.** Comparison of gelatin wound track cavity quarter (a) area and (b) perimeter between tipped and untipped thrusting spear replicates.

| **(a)** | **Inner Quarter Area (cm^2^)** | | | | | | **Outer Quarter Area (cm^2^)** | | | | | |
| --- | --- | --- | --- | --- | --- | --- | --- | --- | --- | --- | --- | --- |
|  | **Mean** | **95% CI** | **Min** | **Max** | **SD** | **N** | **Mean** | **95% CI** | **Min** | **Max** | **SD** | **N** |
| Tipped | 29.2 | 28.3 to 30.0 | 18.7 | 39.4 | 4.1 | 88 | 74.4 | 72.1 to 76.6 | 47.9 | 91.3 | 8.4 | 56 |
| Untipped | 23.4 | 22.2 to 24.6 | 13.7 | 34.0 | 4.9 | 67 | 80.4 | 73.4 to 87.4 | 47.7 | 128.5 | 18.0 | 28 |

| **(b)** | **Inner Quarter Perimeter (cm)** | | | | | | **Outer Quarter Perimeter (cm)** | | | | | |
| --- | --- | --- | --- | --- | --- | --- | --- | --- | --- | --- | --- | --- |
|  | **Mean** | **95% CI** | **Min** | **Max** | **SD** | **N** | **Mean** | **95% CI** | **Min** | **Max** | **SD** | **N** |
| Tipped | 42.2 | 41.6 to 42.8 | 35.1 | 47.3 | 2.9 | 88 | 45.6 | 45.0 to 46.3 | 38.0 | 50.2 | 2.5 | 56 |
| Untipped | 42.0 | 41.4 to 42.6 | 34.7 | 46.0 | 2.6 | 67 | 46.0 | 44.3 to 47.8 | 36.2 | 53.6 | 4.6 | 28 |


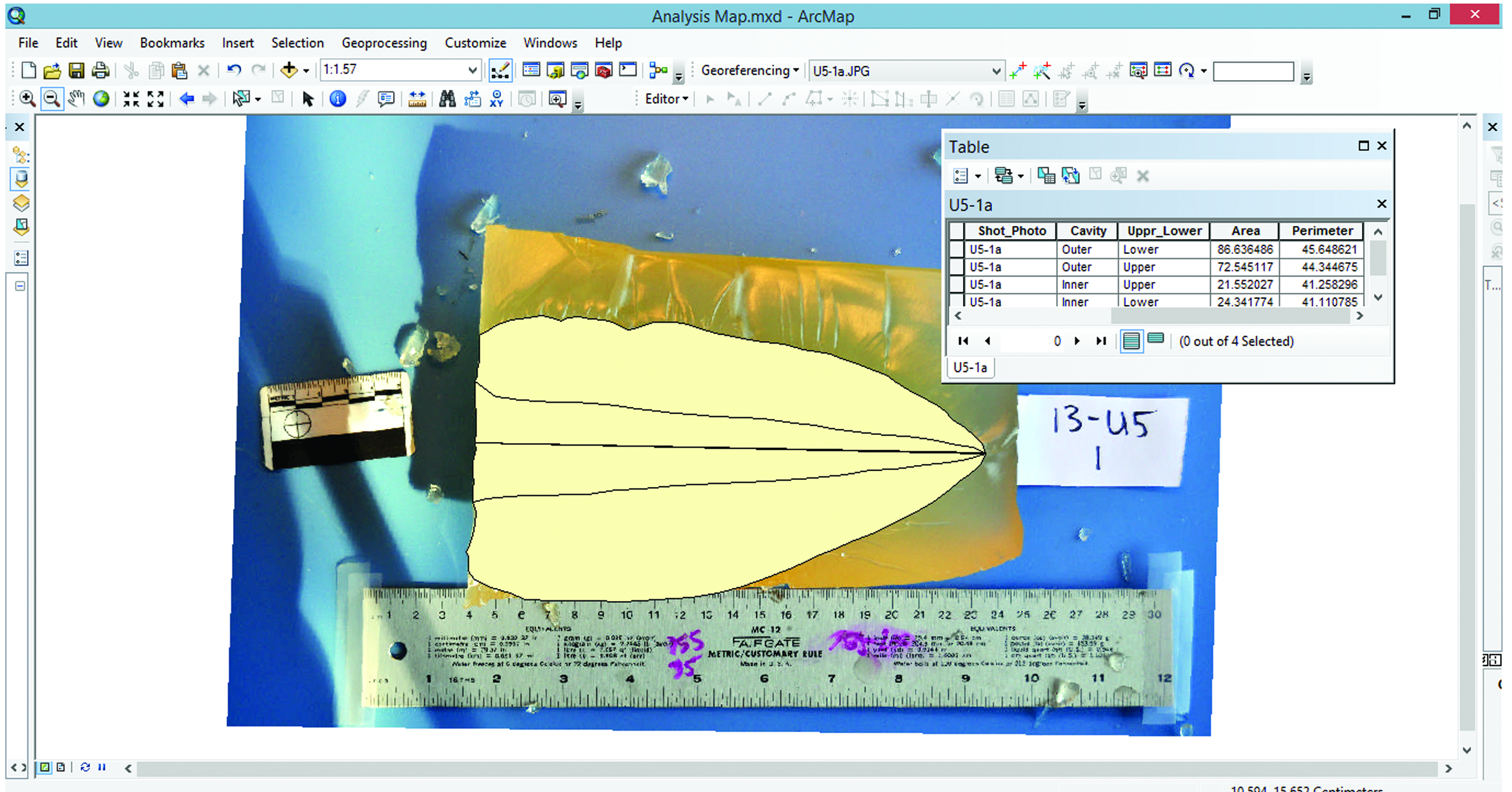


**Figure S1.** Screenshot of ArcGIS shapefiles used to calculate wound track area and perimeter.

**Appendix S1***Shot Data (including two exclusions due to equipment failure)*

| Shot No. | Spear Type | Spear No. | Spear Shot No. | Velocity (km/hr) | Velocity (m/s) | Penetration Depth (cm) | Withdraw Force (kg) | Mass of Detritus (g) | Comments |
| --- | --- | --- | --- | --- | --- | --- | --- | --- | --- |
| 1 | Tipped | T1 | 1 | 31 | 8.6 | 21.0 | nr^*^ | 2.1 |  |
| 2 | Tipped | T1 | 2 | 36 | 10.0 | 20.5 | 6.5 | 0.0 |  |
| 3 | Tipped | T1 | 3 | 31 | 8.6 | 20.5 | 6.5 | 0.0 |  |
| 4 | Tipped | T1 | 4 | 33 | 9.2 | 19.0 | 8.5 | 0.1 |  |
| 5 | Tipped | T1 | 5 | 35 | 9.7 | 21.8 | 6.8 | 2.7 |  |
| 6 | Untipped | U1 | 1 | nr | - | 21.4 | 6.7 | 0.0 |  |
| 7 | Untipped | U1 | 2 | 35 | 9.7 | 22.2 | 6.9 | 0.9 |  |
| 8 | Untipped | U1 | 3 | nr | - | 25.5 | 5.1 | 0.0 |  |
| 9 | Untipped | U1 | 4 | nr | - | 22.1 | 6.1 | 0.0 |  |
| 10 | Untipped | U1 | 5 | 33 | 9.2 | nr | nr | 0.0 |  |
| 11 | Tipped | T2 | 1 | nr | - | 19.0 | 4.4 | 0.2 |  |
| 12 | Tipped | T2 | 2 | nr | - | 20.1 | 5.8 | 0.0 |  |
| 13 | Tipped | T2 | 3 | 36 | 10.0 | 20.1 | 4.5 | 0.0 |  |
| 14 | Tipped | T2 | 4 | nr | - | 19.5 | 6.1 | 0.0 |  |
| 15 | Tipped | T2 | 5 | 31 | 8.6 | 19.5 | 7.1 | 1.0 |  |
| 16 | Untipped | U2 | 1 | 30 | 8.3 | 20.0 | 6.4 | 0.1 |  |
| 17 | Untipped | U2 | 2 | 33 | 9.2 | 22.2 | 5.4 | 0.0 |  |
| 18 | Untipped | U2 | 3 | nr | - | 21.8 | 6.7 | 0.0 |  |
| 19 | Untipped | U2 | 4 | nr | - | 23.9 | 7.7 | 0.0 |  |
| 20 | Untipped | U2 | 5 | 33 | 9.2 | 24.1 | 5.4 | 0.0 |  |
| 21 | Tipped | T3 | 1 | 37 | 10.3 | 15.2 | 4.3 | 0.0 |  |
| 22 | Tipped | T3 | 2 | 37 | 10.3 | 22.8 | 7.1 | 0.0 |  |
| 23^+^ | Tipped | T3 | 3 | 37 | 10.3 | 20.2 | 5.8 | 0.3 | equipment failure, excluded |
| 24 | Tipped | T3 | 4 | na^**^ | - | na | na | na | no shot because tip broke |
| 25 | Tipped | T3 | 5 | na | - | na | na | na | no shot because tip broke |
| 26 | Untipped | U3 | 1 | 28 | 7.8 | 19.6 | 4.5 | 0.0 |  |
| 27 | Untipped | U3 | 2 | 34 | 9.4 | 23.3 | 4.6 | 0.0 |  |
| 28 | Untipped | U3 | 3 | nr | - | 22.7 | 6.6 | 0.0 |  |
| 29 | Untipped | U3 | 4 | nr | - | 19.3 | 3.8 | 0.0 |  |
| 30 | Untipped | U3 | 5 | nr | - | 20.3 | 4.2 | 0.0 |  |
| 31 | Tipped | T4 | 1 | nr | - | 18.8 | 7.8 | 0.0 |  |
| 32 | Tipped | T4 | 2 | nr | - | 22.2 | 7.6 | 0.0 |  |
| 33 | Tipped | T4 | 3 | nr | - | 20.5 | 8.3 | 0.0 |  |
| 34 | Tipped | T4 | 4 | 30 | 8.3 | 21.3 | 5.7 | 0.0 |  |
| 35 | Tipped | T4 | 5 | 36 | 10.0 | 23.2 | 7.4 | 0.0 |  |
| 36 | Untipped | U4 | 1 | 31 | 8.6 | 22.1 | 7.3 | 0.0 |  |
| 37 | Untipped | U4 | 2 | nr | - | 24.1 | 6.7 | 0.0 |  |
| 38 | Untipped | U4 | 3 | nr | - | 23.8 | 6.8 | 0.0 |  |
| 39 | Untipped | U4 | 4 | 34 | 9.4 | 21.6 | nr | 0.0 |  |
| 40 | Untipped | U4 | 5 | nr | - | 24.8 | 6 | 0.0 |  |
| 41 | Tipped | T5 | 1 | 34 | 9.4 | 18.8 | 5.8 | 0.2 |  |
| 42 | Tipped | T5 | 2 | nr | - | 19.8 | 7.4 | 0.0 |  |
| 43 | Tipped | T5 | 3 | nr | - | 20.5 | 6.9 | 0.0 |  |
| 44 | Tipped | T5 | 4 | 33 | 9.2 | 19.5 | 5.9 | 0.0 |  |
| 45 | Tipped | T5 | 5 | nr | - | 17.0 | 6.4 | 0.0 |  |
| 46 | Untipped | U5 | 1 | nr | - | 19.7 | 11.1 | 0.0 |  |
| 47 | Untipped | U5 | 2 | nr | - | 19.4 | 10.5 | 0.0 |  |
| 48 | Untipped | U5 | 3 | 30 | 8.3 | 19.7 | 6.9 | 0.0 |  |
| 49^+^ | Untipped | U5 | 4 | 23 | 6.4 | 16.1 | 3.8 | 0.0 | equipment failure, excluded |
| 50 | Untipped | U5 | 5 | na | - | na | na | na | no shot because of equipment failure |

^*^nr=not recorded, ^**^na=not applicable, ^+^shot excluded from analyses

**Appendix S2**

Wound Track Data

| **Spear Type** | **Shot_Photo** | **Cavity** | **Upper_Lower** | **Area** | **Perimeter** |
| --- | --- | --- | --- | --- | --- |
| Tipped | T1-4b | Inner | Upper | 25.52 | 39.44 |
| Tipped | T1-4b | Inner | Lower | 29.41 | 39.95 |
| Tipped | T1-4b | Outer | Upper | 69.22 | 43.10 |
| Tipped | T1-4b | Outer | Lower | 70.35 | 44.36 |
| Tipped | T1-4a | Outer | Upper | 72.06 | 43.97 |
| Tipped | T1-4a | Outer | Lower | 72.03 | 44.24 |
| Tipped | T1-4a | Inner | Lower | 29.00 | 39.22 |
| Tipped | T1-4a | Inner | Upper | 29.80 | 39.85 |
| Tipped | T1-3b | Inner | Upper | 26.38 | 41.80 |
| Tipped | T1-3b | Inner | Lower | 31.30 | 42.40 |
| Tipped | T1-3b | Outer | Lower | 79.76 | 46.68 |
| Tipped | T1-3a | Inner | Upper | 30.93 | 42.32 |
| Tipped | T1-3a | Inner | Lower | 26.34 | 41.98 |
| Tipped | T1-3a | Outer | Upper | 77.32 | 46.39 |
| Tipped | T1-2b | Inner | Lower | 27.16 | 40.93 |
| Tipped | T1-2b | Inner | Upper | 25.54 | 40.33 |
| Tipped | T1-2b | Outer | Lower | 70.05 | 44.53 |
| Tipped | T1-2a | Outer | Upper | 62.53 | 42.47 |
| Tipped | T1-2a | Inner | Lower | 30.35 | 39.10 |
| Tipped | T1-2a | Inner | Upper | 29.18 | 39.12 |
| Tipped | T1-1b | Outer | Upper | 91.29 | 47.90 |
| Tipped | T1-1b | Inner | Upper | 25.38 | 42.27 |
| Tipped | T1-1b | Outer | Lower | 77.39 | 46.93 |
| Tipped | T1-1b | Inner | Lower | 34.07 | 42.50 |
| Tipped | T1-1a | Outer | Upper | 71.95 | 45.47 |
| Tipped | T1-1a | Inner | Upper | 33.68 | 42.54 |
| Tipped | T1-1a | Outer | Lower | 89.20 | 47.19 |
| Tipped | T1-1a | Inner | Lower | 28.41 | 42.59 |
| Tipped | T1-5a | Outer | Upper | 77.32 | 49.61 |
| Tipped | T1-5a | Inner | Upper | 30.90 | 45.05 |
| Tipped | T1-5a | Inner | Lower | 35.82 | 44.53 |
| Tipped | T1-5b | Inner | Upper | 31.72 | 46.25 |
| Tipped | T1-5b | Inner | Lower | 32.82 | 46.74 |
| Tipped | T1-5b | Outer | Lower | 84.18 | 50.19 |
| Tipped | T2-1a | Inner | Upper | 32.19 | 41.51 |
| Tipped | T2-1a | Inner | Lower | 26.60 | 41.82 |
| Tipped | T2-1a | Outer | Upper | 84.05 | 45.96 |
| Tipped | T2-1a | Outer | Lower | 68.13 | 46.43 |
| Tipped | T2-1b | Inner | Upper | 29.69 | 41.10 |
| Tipped | T2-1b | Inner | Lower | 27.15 | 41.10 |
| Tipped | T2-1b | Outer | Upper | 69.39 | 44.06 |
| Tipped | T2-1b | Outer | Lower | 71.77 | 44.49 |
| Tipped | T2-2a | Inner | Lower | 28.64 | 43.33 |
| Tipped | T2-2a | Outer | Lower | 69.30 | 47.12 |
| Tipped | T2-2a | Outer | Upper | 77.91 | 47.08 |
| Tipped | T2-2a | Inner | Upper | 29.05 | 43.35 |
| Tipped | T2-2b | Inner | Upper | 29.69 | 41.61 |
| Tipped | T2-2b | Inner | Lower | 26.18 | 41.51 |
| Tipped | T2-2b | Outer | Upper | 71.33 | 45.30 |
| Tipped | T2-2b | Outer | Lower | 71.04 | 44.67 |
| Tipped | T2-3a | Inner | Upper | 29.08 | 41.91 |
| Tipped | T2-3a | Inner | Lower | 30.13 | 42.12 |
| Tipped | T2-3b | Inner | Upper | 30.64 | 42.43 |
| Tipped | T2-3b | Inner | Lower | 26.52 | 42.55 |
| Tipped | T2-3b | Outer | Lower | 67.75 | 45.41 |
| Tipped | T2-4a | Inner | Upper | 28.97 | 40.82 |
| Tipped | T2-4a | Inner | Lower | 30.80 | 40.73 |
| Tipped | T2-4a | Outer | Upper | 67.65 | 44.27 |
| Tipped | T2-4b | Inner | Upper | 29.81 | 41.73 |
| Tipped | T2-4b | Inner | Lower | 26.31 | 42.36 |
| Tipped | T2-4b | Outer | Lower | 72.48 | 46.26 |
| Tipped | T2-5a | Inner | Upper | 31.27 | 41.95 |
| Tipped | T2-5a | Inner | Lower | 35.44 | 41.75 |
| Tipped | T2-5a | Outer | Upper | 63.26 | 44.11 |
| Tipped | T2-5a | Outer | Lower | 84.98 | 46.43 |
| Tipped | T2-5b | Inner | Upper | 27.46 | 41.05 |
| Tipped | T2-5b | Inner | Lower | 28.41 | 41.41 |
| Tipped | T2-5b | Outer | Upper | 70.98 | 44.80 |
| Tipped | T2-5b | Outer | Lower | 62.55 | 43.81 |
| Tipped | T3-1a | Inner | Lower | 28.95 | 35.91 |
| Tipped | T3-1a | Inner | Upper | 19.26 | 35.52 |
| Tipped | T3-1a | Outer | Upper | 47.95 | 38.00 |
| Tipped | T3-1b | Inner | Upper | 28.35 | 38.47 |
| Tipped | T3-1b | Inner | Lower | 26.73 | 39.03 |
| Tipped | T3-1b | Outer | Lower | 53.49 | 40.84 |
| Tipped | T3-3a | Inner | Upper | 29.88 | 44.82 |
| Tipped | T2-3a | Inner | Lower | 35.51 | 44.66 |
| Tipped | T3-3b | Inner | Upper | 33.13 | 44.52 |
| Tipped | T3-3b | Inner | Lower | 33.55 | 44.71 |
| Tipped | T4-1a | Inner | Upper | 25.84 | 40.40 |
| Tipped | T4-1a | Inner | Lower | 31.59 | 41.10 |
| Tipped | T4-1a | Outer | Lower | 78.09 | 45.33 |
| Tipped | T4-1b | Inner | Upper | 33.24 | 40.14 |
| Tipped | T4-1b | Inner | Lower | 23.27 | 39.61 |
| Tipped | T4-1b | Outer | Upper | 74.96 | 43.63 |
| Tipped | T4-2a | Inner | Upper | 28.46 | 46.11 |
| Tipped | T4-2a | Inner | Upper | 36.06 | 45.23 |
| Tipped | T4-2b | Inner | Upper | 39.42 | 46.81 |
| Tipped | T4-2b | Inner | Lower | 30.27 | 47.30 |
| Tipped | T4-3a | Inner | Upper | 31.79 | 45.93 |
| Tipped | T4-3a | Inner | Lower | 34.34 | 45.88 |
| Tipped | T4-3a | Outer | Lower | 86.43 | 49.52 |
| Tipped | t4-3a | Outer | Upper | 65.81 | 48.41 |
| Tipped | T4-3b | Inner | Upper | 31.06 | 45.14 |
| Tipped | T4-3b | Inner | Lower | 34.82 | 45.73 |
| Tipped | T4-3b | Outer | Upper | 81.05 | 48.31 |
| Tipped | T4-3b | Outer | Lower | 73.21 | 48.99 |
| Tipped | T4-4a | Outer | Upper | 69.97 | 48.36 |
| Tipped | T4-4a | Outer | Lower | 80.60 | 49.70 |
| Tipped | T4-4a | Inner | Upper | 31.93 | 46.68 |
| Tipped | T4-4a | Inner | Lower | 28.40 | 45.52 |
| Tipped | T4-4b | Outer | Upper | 85.94 | 50.15 |
| Tipped | T4-4b | Outer | Lower | 70.41 | 49.47 |
| Tipped | T4-4b | Inner | Upper | 30.79 | 46.47 |
| Tipped | T4-4b | Inner | Lower | 34.15 | 46.88 |
| Tipped | T4-5a | Inner | Upper | 27.98 | 45.53 |
| Tipped | T4-5a | Inner | Lower | 34.40 | 45.30 |
| Tipped | T4-5b | Inner | Upper | 36.62 | 46.72 |
| Tipped | T4-5b | Inner | Lower | 35.75 | 46.68 |
| Tipped | T5-1a | Inner | Upper | 30.12 | 40.12 |
| Tipped | T5-1a | Inner | Lower | 19.62 | 39.41 |
| Tipped | T5-1a | Outer | Lower | 69.43 | 45.59 |
| Tipped | T5-1b | Inner | Upper | 22.91 | 42.80 |
| Tipped | T5-1b | Inner | Lower | 26.38 | 42.79 |
| Tipped | T5-1b | Outer | Upper | 75.49 | 46.67 |
| Tipped | T5-2a | Inner | Upper | 29.90 | 40.28 |
| Tipped | T5-2a | Inner | Lower | 24.65 | 40.05 |
| Tipped | T5-2a | Outer | Upper | 67.23 | 43.58 |
| Tipped | T5-2a | Outer | Lower | 79.16 | 46.21 |
| Tipped | T5-2b | Inner | Upper | 23.19 | 40.41 |
| Tipped | T5-2b | Inner | Lower | 29.89 | 40.77 |
| Tipped | T5-2b | Outer | Upper | 69.89 | 43.41 |
| Tipped | T5-2b | Outer | Lower | 70.43 | 44.48 |
| Tipped | T5-3a | Inner | Upper | 32.15 | 44.77 |
| Tipped | T5-3a | Inner | Lower | 26.68 | 44.62 |
| Tipped | T5-3a | Outer | Lower | 74.39 | 48.29 |
| Tipped | T5-3b | Inner | Upper | 28.97 | 45.19 |
| Tipped | T5-3b | Inner | Lower | 30.55 | 45.30 |
| Tipped | T5-3b | Outer | Upper | 76.91 | 48.64 |
| Tipped | T5-4a | Inner | Upper | 24.48 | 39.87 |
| Tipped | T5-4a | Inner | Lower | 23.16 | 40.14 |
| Tipped | T5-4a | Outer | Upper | 84.90 | 45.25 |
| Tipped | T5-4a | Outer | Lower | 88.10 | 45.33 |
| Tipped | T5-4b | Inner | Upper | 23.19 | 39.93 |
| Tipped | T5-4b | Inner | Upper | 26.57 | 40.40 |
| Tipped | T5-4b | Outer | Lower | 85.95 | 45.55 |
| Tipped | T5-5a | Inner | Upper | 21.47 | 35.09 |
| Tipped | T5-5a | Inner | Lower | 23.82 | 35.80 |
| Tipped | T5-5a | Outer | Upper | 82.50 | 41.28 |
| Tipped | T5-5a | Outer | Lower | 79.08 | 43.27 |
| Tipped | T5-5b | Inner | Upper | 26.07 | 36.44 |
| Tipped | T5-5b | Inner | Lower | 18.72 | 36.01 |
| Tipped | T5-5b | Outer | Upper | 77.35 | 42.68 |
| Tipped | T5-5b | Outer | Lower | 78.62 | 41.61 |
| Untipped | U1-1a | Inner | Upper | 20.14 | 41.31 |
| Untipped | U1-1a | Inner | Lower | 28.42 | 41.78 |
| Untipped | U1-1b | Inner | Lower | 22.12 | 40.95 |
| Untipped | U1-1b | Inner | Upper | 28.97 | 41.34 |
| Untipped | U1-2a | Inner | Upper | 19.01 | 45.70 |
| Untipped | U1-2a | Inner | Lower | 22.33 | 45.86 |
| Untipped | U1-21 | Outer | Upper | 89.90 | 53.14 |
| Untipped | U1-2b | Inner | Upper | 16.49 | 45.59 |
| Untipped | U1-2b | Inner | Lower | 26.74 | 45.99 |
| Untipped | U1-2b | Outer | Lower | 100.64 | 53.55 |
| Untipped | U1-4a | Inner | Lower | 22.24 | 43.66 |
| Untipped | U1-4a | Inner | Upper | 28.32 | 44.02 |
| Untipped | U1-4a | Outer | Lower | 78.71 | 47.90 |
| Untipped | U1-4a | Outer | Upper | 91.74 | 49.78 |
| Untipped | U1-4a | Inner | Upper | 26.01 | 44.68 |
| Untipped | U1-4a | Inner | Lower | 26.83 | 45.36 |
| Untipped | U1-5a | Inner | Lower | 20.84 | 44.20 |
| Untipped | U1-5a | Inner | Upper | 28.34 | 44.29 |
| Untipped | U1-5b | Inner | Lower | 22.18 | 43.76 |
| Untipped | U1-5b | Inner | Upper | 22.31 | 43.61 |
| Untipped | U2-1a | Outer | Upper | 75.86 | 46.31 |
| Untipped | U2-1a | Inner | Lower | 28.27 | 43.11 |
| Untipped | U2-1a | Inner | Upper | 21.36 | 42.66 |
| Untipped | U2-1a | Outer | Lower | 73.44 | 46.38 |
| Untipped | U2-1b | Outer | Upper | 65.99 | 44.81 |
| Untipped | U2-1b | Outer | Lower | 67.82 | 45.07 |
| Untipped | U2-1b | Inner | Lower | 17.62 | 41.35 |
| Untipped | U2-1b | Inner | Upper | 25.77 | 41.51 |
| Untipped | U2-2a | Inner | Lower | 27.68 | 43.50 |
| Untipped | U2-2a | Inner | Upper | 25.24 | 43.32 |
| Untipped | U2-2b | Inner | Upper | 30.83 | 43.48 |
| Untipped | U2-2b | Inner | Upper | 28.35 | 43.69 |
| Untipped | U2-3a | Inner | Upper | 23.37 | 41.87 |
| Untipped | U2-3a | Inner | Lower | 34.00 | 42.13 |
| Untipped | U2-3a | Outer | Upper | 77.44 | 47.33 |
| Untipped | U2-3b | Inner | Upper | 28.04 | 40.87 |
| Untipped | U2-3b | Inner | Lower | 17.18 | 40.76 |
| Untipped | U2-3b | Outer | Lower | 77.38 | 47.20 |
| Untipped | U2-4a | Inner | Lower | 32.28 | 43.95 |
| Untipped | U2-4a | Inner | Upper | 26.57 | 43.71 |
| Untipped | U2-4b | Inner | Upper | 29.39 | 42.60 |
| Untipped | U2-4b | Inner | Lower | 28.53 | 42.69 |
| Untipped | U3-1c | Inner | Upper | 18.75 | 39.56 |
| Untipped | U3-1c | Inner | Lower | 13.68 | 39.64 |
| Untipped | U3-1c | Outer | Upper | 47.70 | 42.81 |
| Untipped | U3-1c | Outer | Lower | 73.58 | 48.77 |
| Untipped | U3-3d | Inner | Upper | 19.89 | 40.33 |
| Untipped | U3-3d | Inner | Lower | 16.25 | 40.23 |
| Untipped | U3-4a | Inner | Lower | 14.30 | 40.36 |
| Untipped | U3-4a | Outer | Lower | 112.56 | 50.63 |
| Untipped | U3-4b | Outer | Upper | 128.50 | 52.10 |
| Untipped | U3-4b | Inner | Lower | 19.73 | 39.65 |
| Untipped | U3-4b | Inner | Upper | 17.07 | 39.76 |
| Untipped | U3-5c | Inner | Upper | 19.11 | 42.13 |
| Untipped | U3-5c | Inner | Lower | 23.45 | 42.73 |
| Untipped | U3-5c | Outer | Upper | 114.20 | 50.97 |
| Untipped | U3-5c | Outer | Lower | 90.98 | 47.37 |
| Untipped | U4-1a | Inner | Upper | 28.18 | 45.83 |
| Untipped | U4-1a | Inner | Lower | 27.77 | 45.84 |
| Untipped | U4-1b | Inner | Upper | 26.43 | 45.23 |
| Untipped | U4-1b | Inner | Lower | 27.06 | 44.89 |
| Untipped | U4-3b | Inner | Upper | 28.96 | 43.94 |
| Untipped | U4-3b | Inner | Lower | 24.88 | 43.91 |
| Untipped | U4-5a | Inner | Upper | 22.17 | 43.31 |
| Untipped | U4-5a | Inner | Lower | 30.88 | 43.99 |
| Untipped | U4-5b | Inner | Upper | 30.52 | 43.33 |
| Untipped | U4-5b | Inner | Lower | 25.97 | 43.28 |
| Untipped | U5-1a | Inner | Upper | 21.55 | 41.26 |
| Untipped | U5-1a | Inner | Lower | 24.34 | 41.11 |
| Untipped | U5-1a | Outer | Lower | 86.64 | 45.65 |
| Untipped | U5-1a | Outer | Upper | 72.55 | 44.34 |
| Untipped | U5-1b | Inner | Upper | 22.68 | 40.70 |
| Untipped | U5-1b | Inner | Lower | 25.36 | 41.05 |
| Untipped | U5-1b | Outer | Upper | 85.93 | 45.78 |
| Untipped | U5-1b | Outer | Lower | 78.44 | 44.95 |
| Untipped | U5-2a | Inner | Upper | 17.43 | 39.41 |
| Untipped | U5-2a | Inner | Lower | 24.26 | 40.34 |
| Untipped | U5-2a | Outer | Upper | 69.93 | 43.30 |
| Untipped | U5-2b | Inner | Upper | 22.86 | 40.06 |
| Untipped | U5-2b | Inner | Lower | 20.38 | 39.60 |
| Untipped | U5-2b | Outer | Lower | 71.81 | 44.03 |
| Untipped | U5-3a | Inner | Upper | 19.57 | 38.41 |
| Untipped | U5-3a | Inner | Lower | 21.44 | 38.69 |
| Untipped | U5-3a | Outer | Upper | 85.85 | 48.55 |
| Untipped | U5-3b | Inner | Upper | 21.79 | 39.03 |
| Untipped | U5-3b | Inner | Upper | 21.01 | 39.48 |
| Untipped | U5-3b | Outer | Lower | 87.75 | 48.90 |
| Untipped | U5-4a | Inner | Upper | 16.06 | 36.53 |
| Untipped | U5-4a | Inner | Lower | 19.43 | 36.97 |
| Untipped | U5-4a | Outer | Upper | 66.96 | 38.05 |
| Untipped | U5-4a | Outer | Lower | 61.93 | 38.31 |
| Untipped | U5-4b | Inner | Upper | 14.54 | 35.01 |
| Untipped | U5-4b | Inner | Lower | 15.11 | 34.73 |
| Untipped | U5-4b | Outer | Upper | 55.00 | 36.25 |
| Untipped | U5-4b | Outer | Lower | 61.81 | 36.76 |
