# Supplementary figures and images for: An Experimental Investigation of the Functional Hypothesis and Evolutionary Advantage of Stone-Tipped Spears (part 2 of 2)
Source: PLoS One. 2014 Aug 27;9(8):e104514. doi: 10.1371/journal.pone.0104514 (PMC4146534; doi:10.1371/journal.pone.0104514)

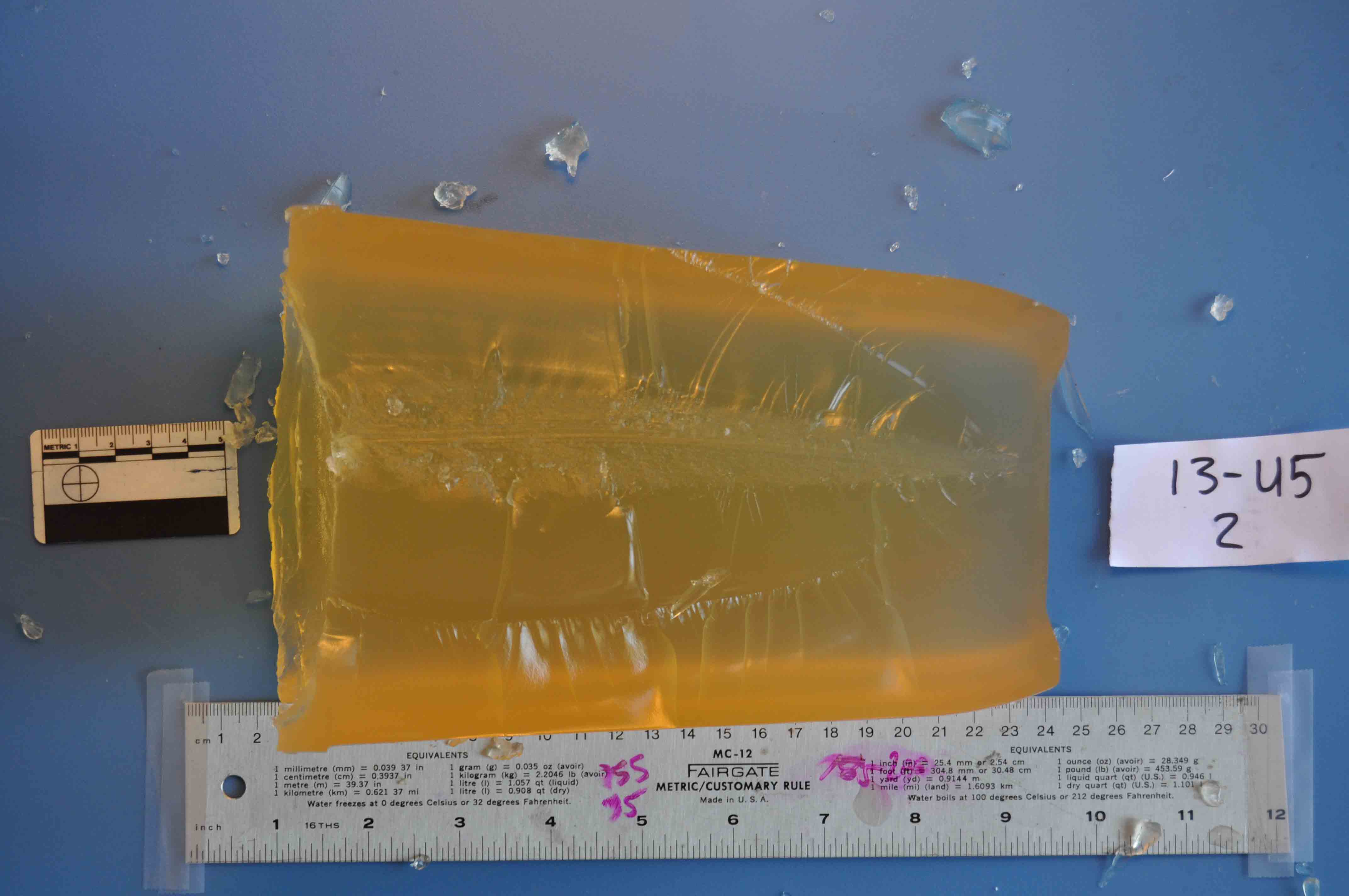

Supplement: File S2 — Wound track images, shapefiles, and tps files. (ZIP) [file pone.0104514.s002.zip › File S2/JPEGS/U5-2b.jpg]

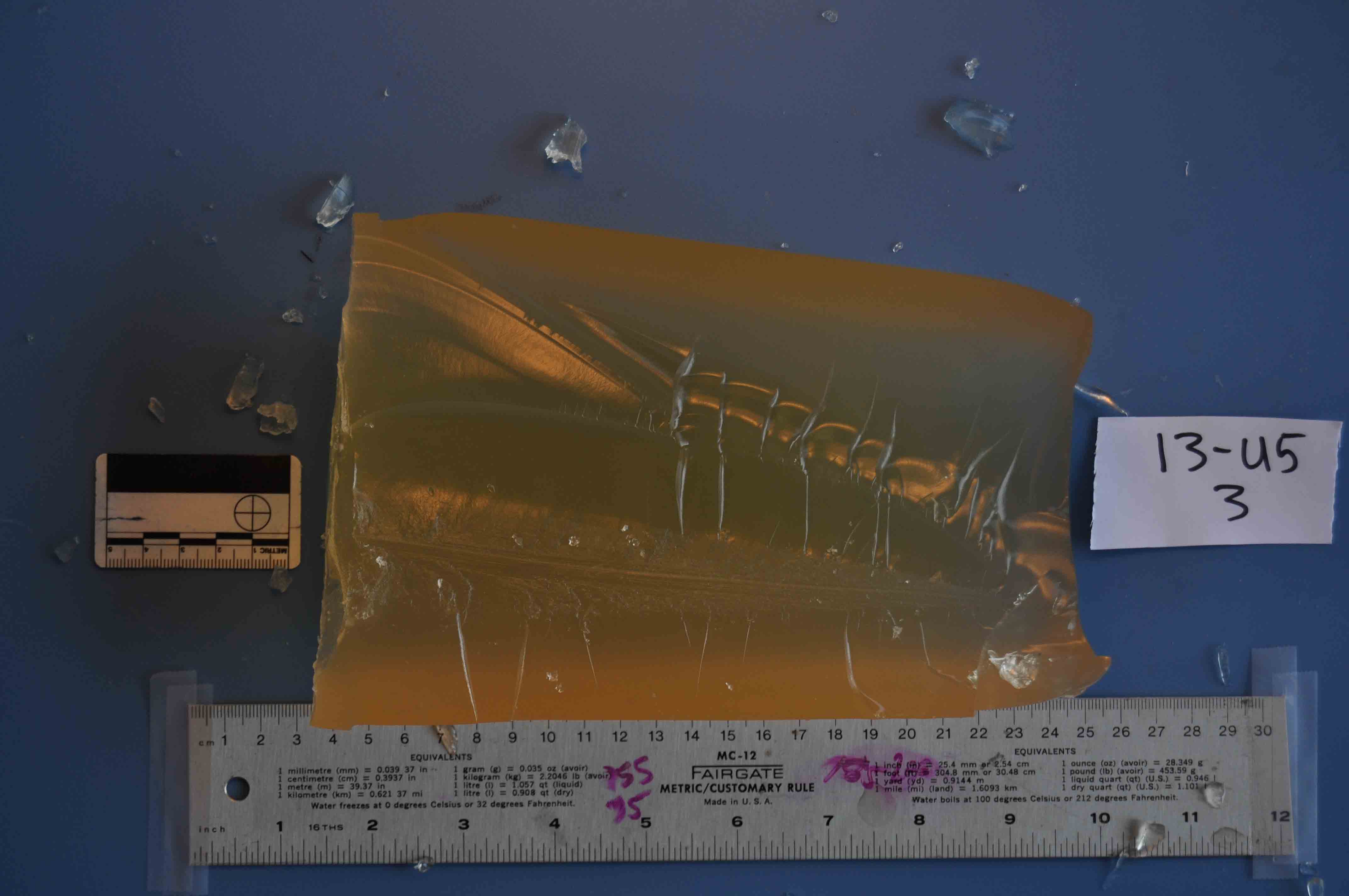

Supplement: File S2 — Wound track images, shapefiles, and tps files. (ZIP) [file pone.0104514.s002.zip › File S2/JPEGS/U5-3a.jpg]

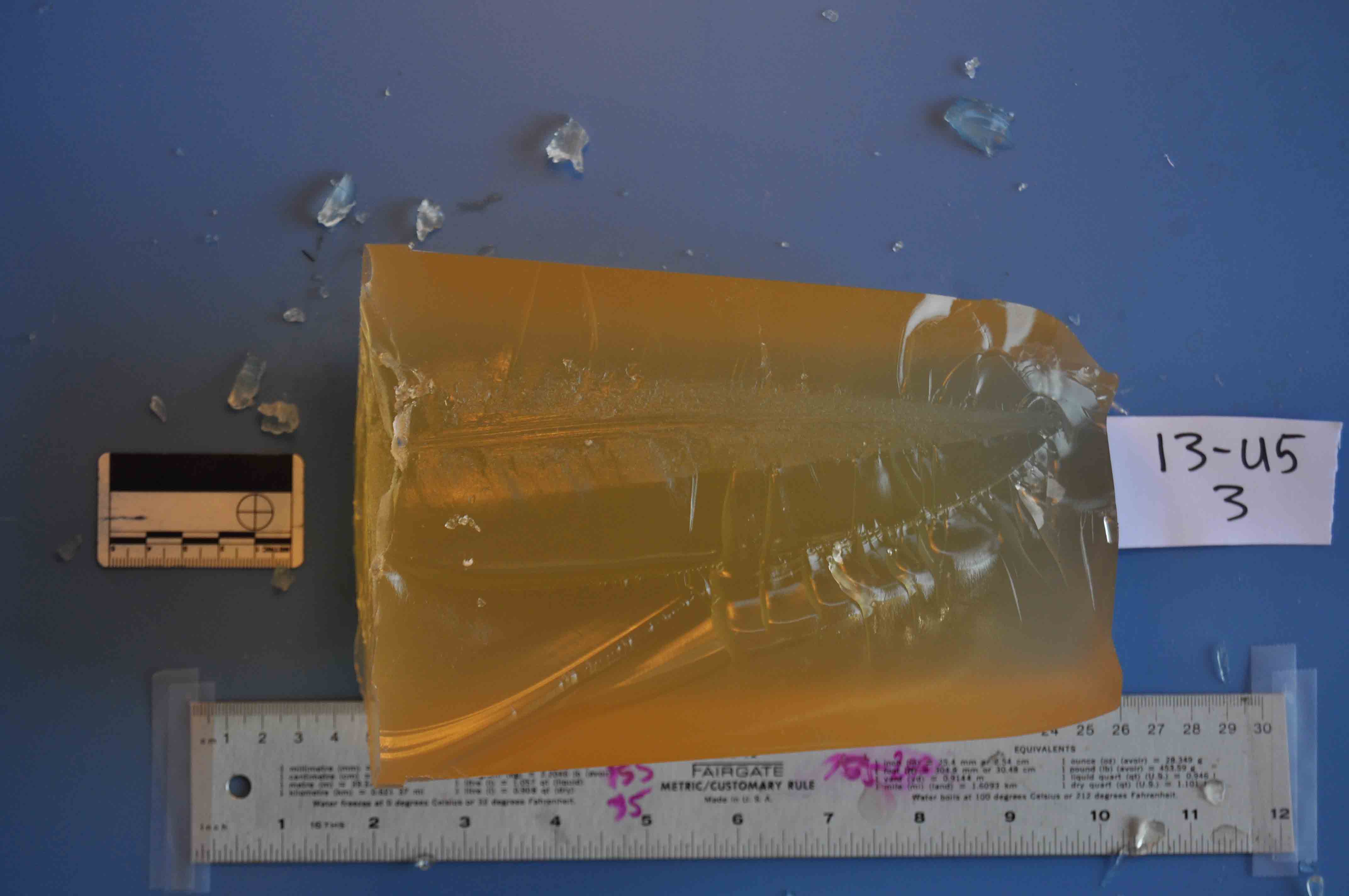

Supplement: File S2 — Wound track images, shapefiles, and tps files. (ZIP) [file pone.0104514.s002.zip › File S2/JPEGS/U5-3b.jpg]

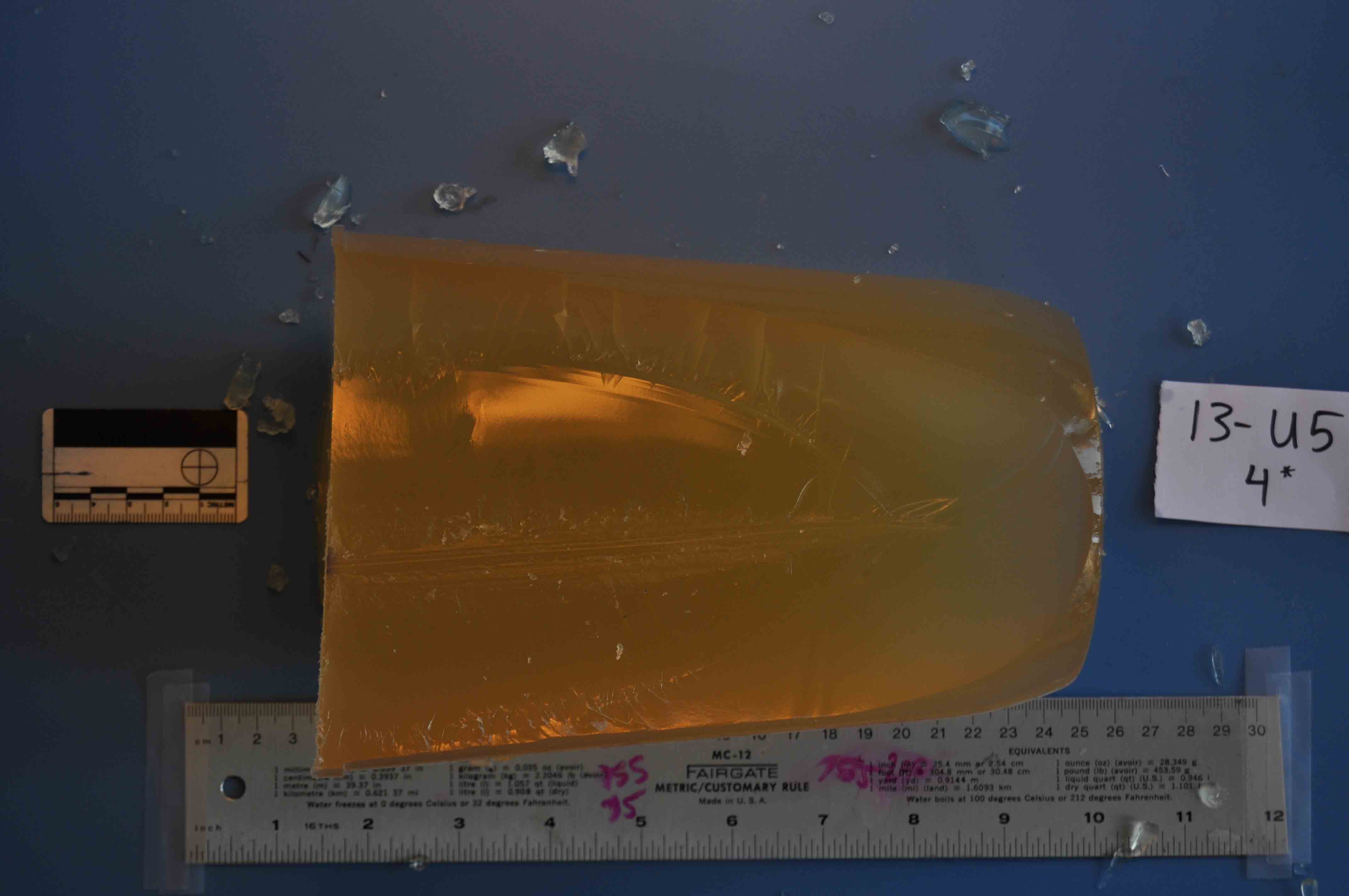

Supplement: File S2 — Wound track images, shapefiles, and tps files. (ZIP) [file pone.0104514.s002.zip › File S2/JPEGS/U5-4a.jpg]

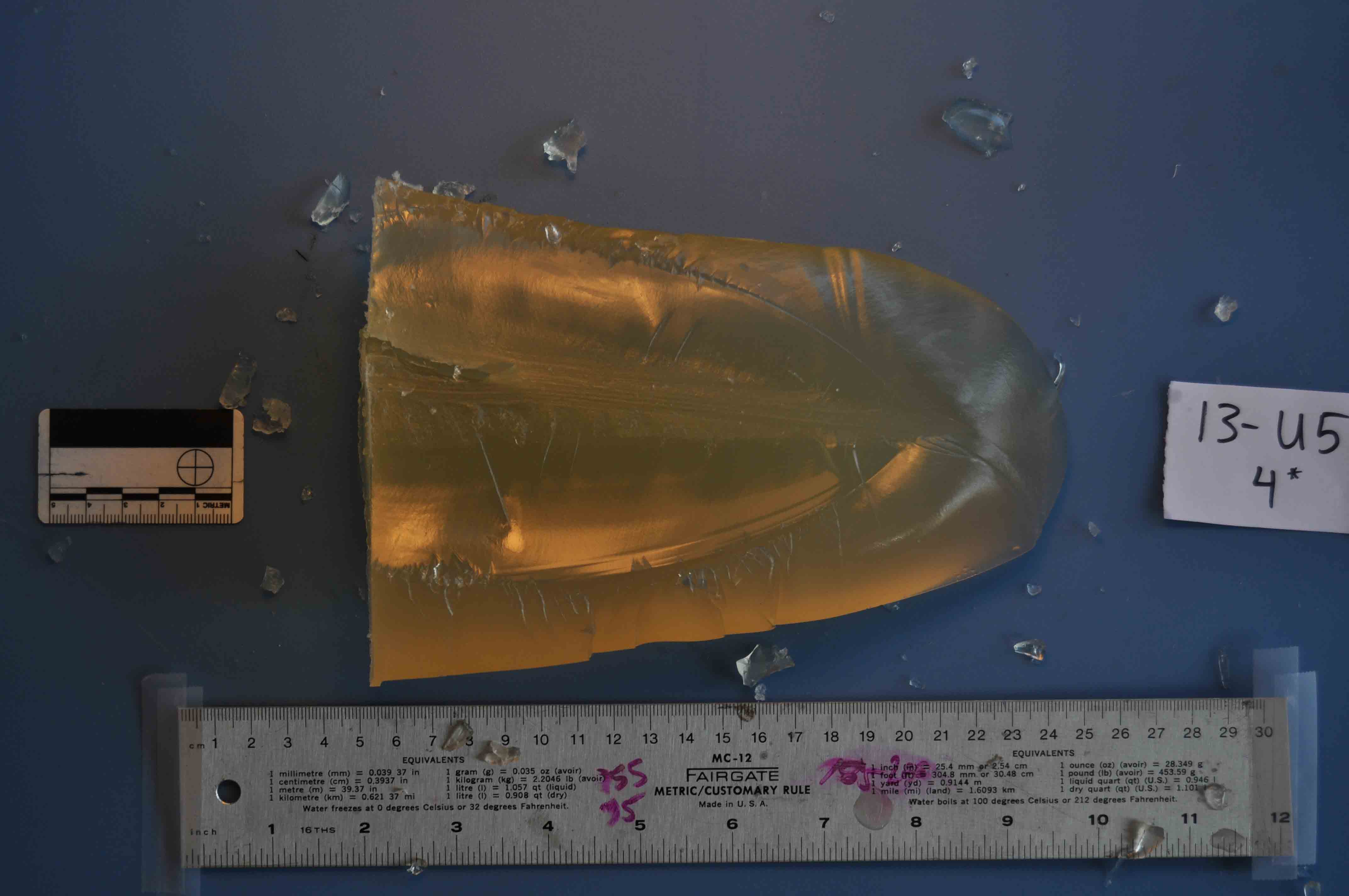

Supplement: File S2 — Wound track images, shapefiles, and tps files. (ZIP) [file pone.0104514.s002.zip › File S2/JPEGS/U5-4b.jpg]
