# Supplementary figures and images for: An Experimental Investigation of the Functional Hypothesis and Evolutionary Advantage of Stone-Tipped Spears (part 1 of 2)
Source: PLoS One. 2014 Aug 27;9(8):e104514. doi: 10.1371/journal.pone.0104514 (PMC4146534; doi:10.1371/journal.pone.0104514)

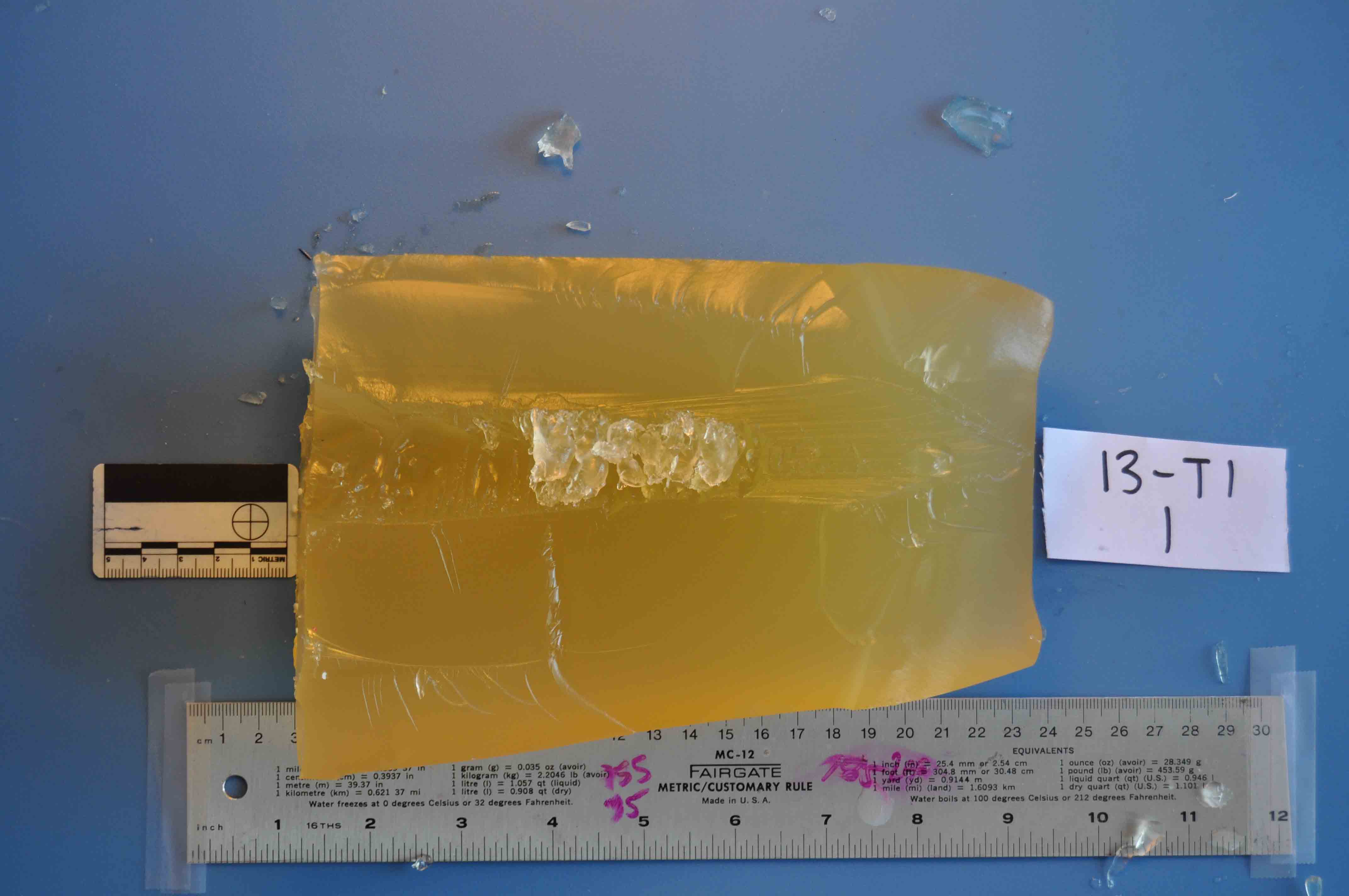

Supplement: File S2 — Wound track images, shapefiles, and tps files. (ZIP) [file pone.0104514.s002.zip › File S2/JPEGS/T1-1a.jpg]

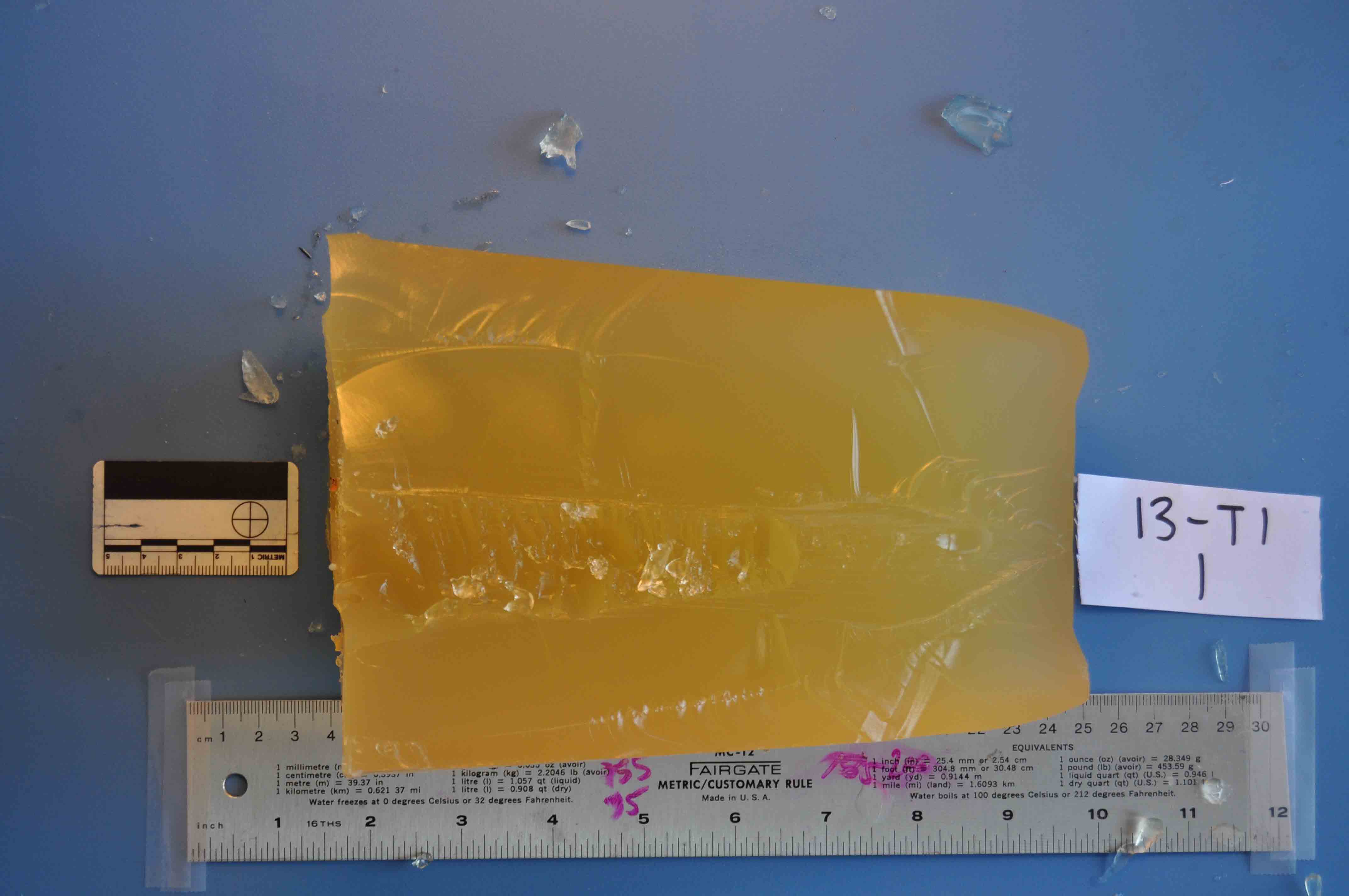

Supplement: File S2 — Wound track images, shapefiles, and tps files. (ZIP) [file pone.0104514.s002.zip › File S2/JPEGS/T1-1b.jpg]

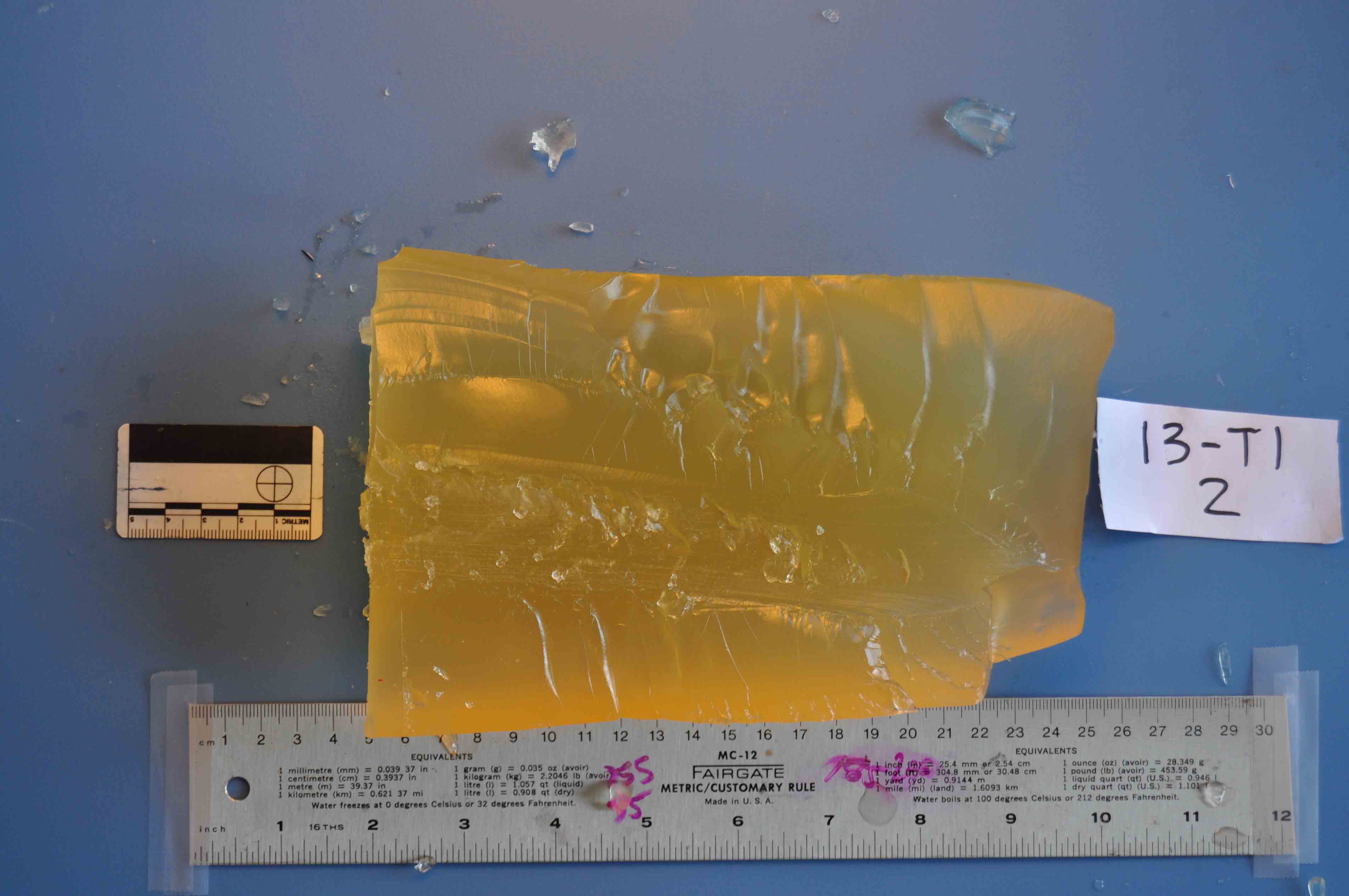

Supplement: File S2 — Wound track images, shapefiles, and tps files. (ZIP) [file pone.0104514.s002.zip › File S2/JPEGS/T1-2a.jpg]

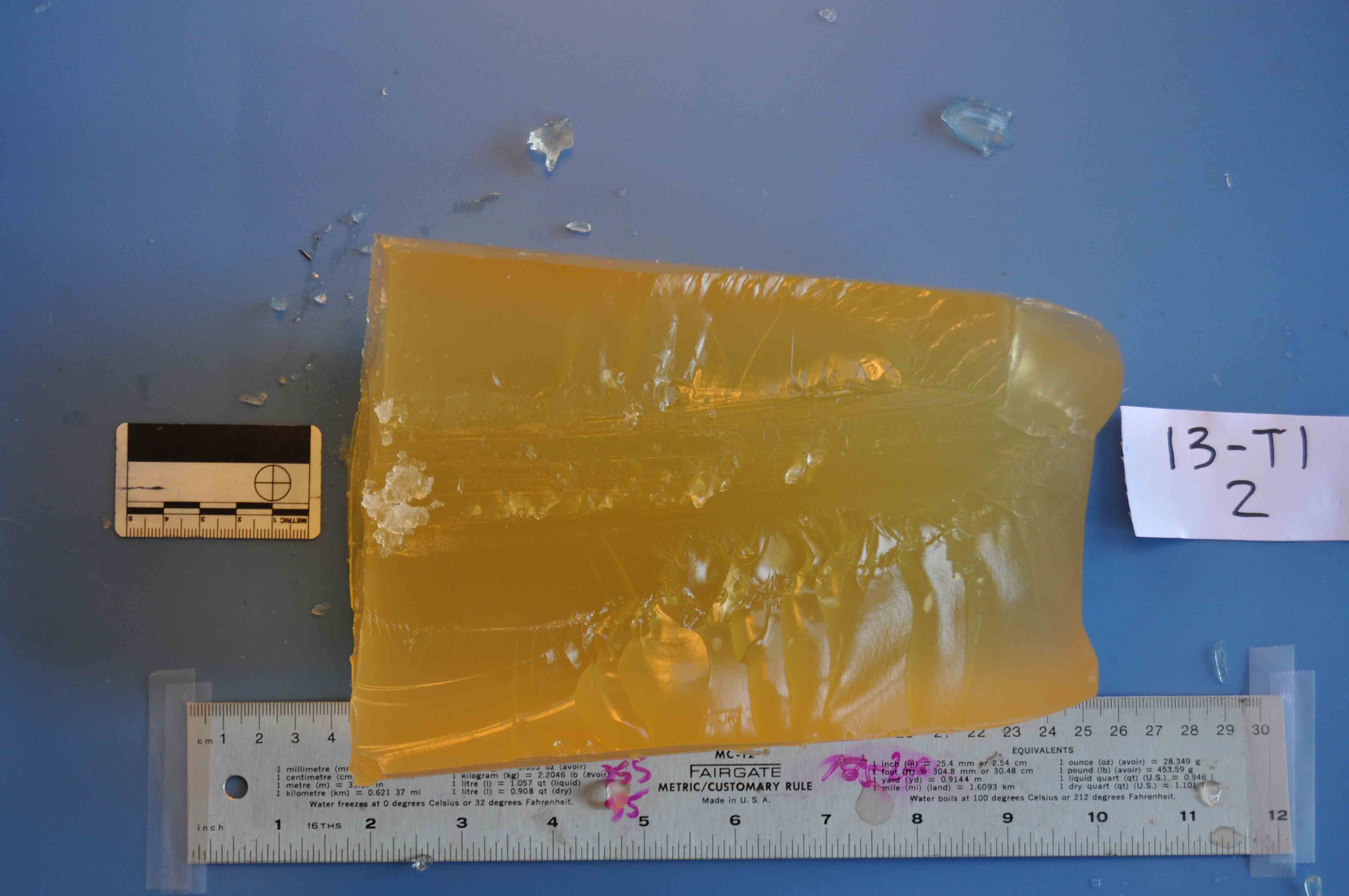

Supplement: File S2 — Wound track images, shapefiles, and tps files. (ZIP) [file pone.0104514.s002.zip › File S2/JPEGS/T1-2b.jpg]

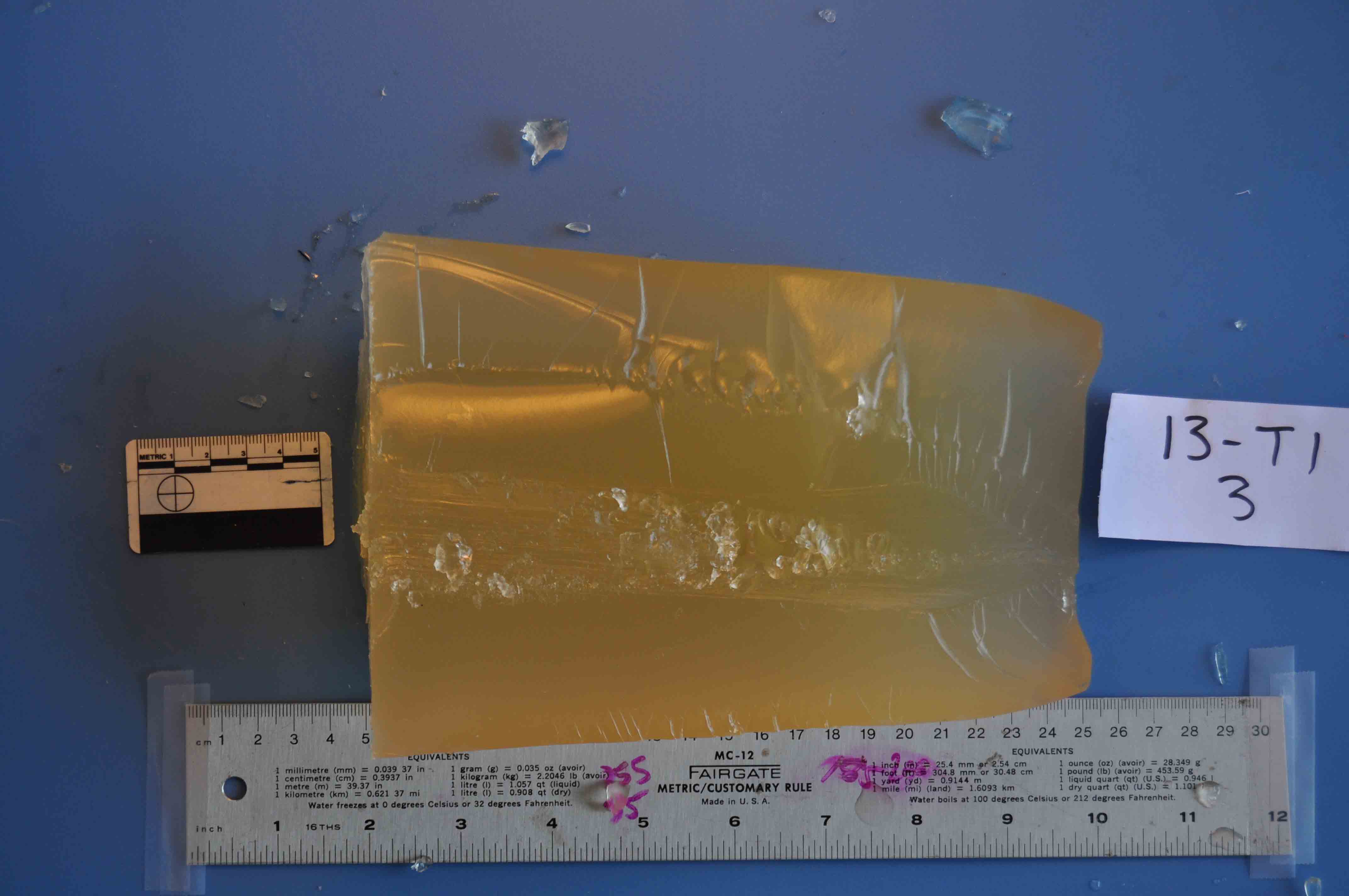

Supplement: File S2 — Wound track images, shapefiles, and tps files. (ZIP) [file pone.0104514.s002.zip › File S2/JPEGS/T1-3a.jpg]

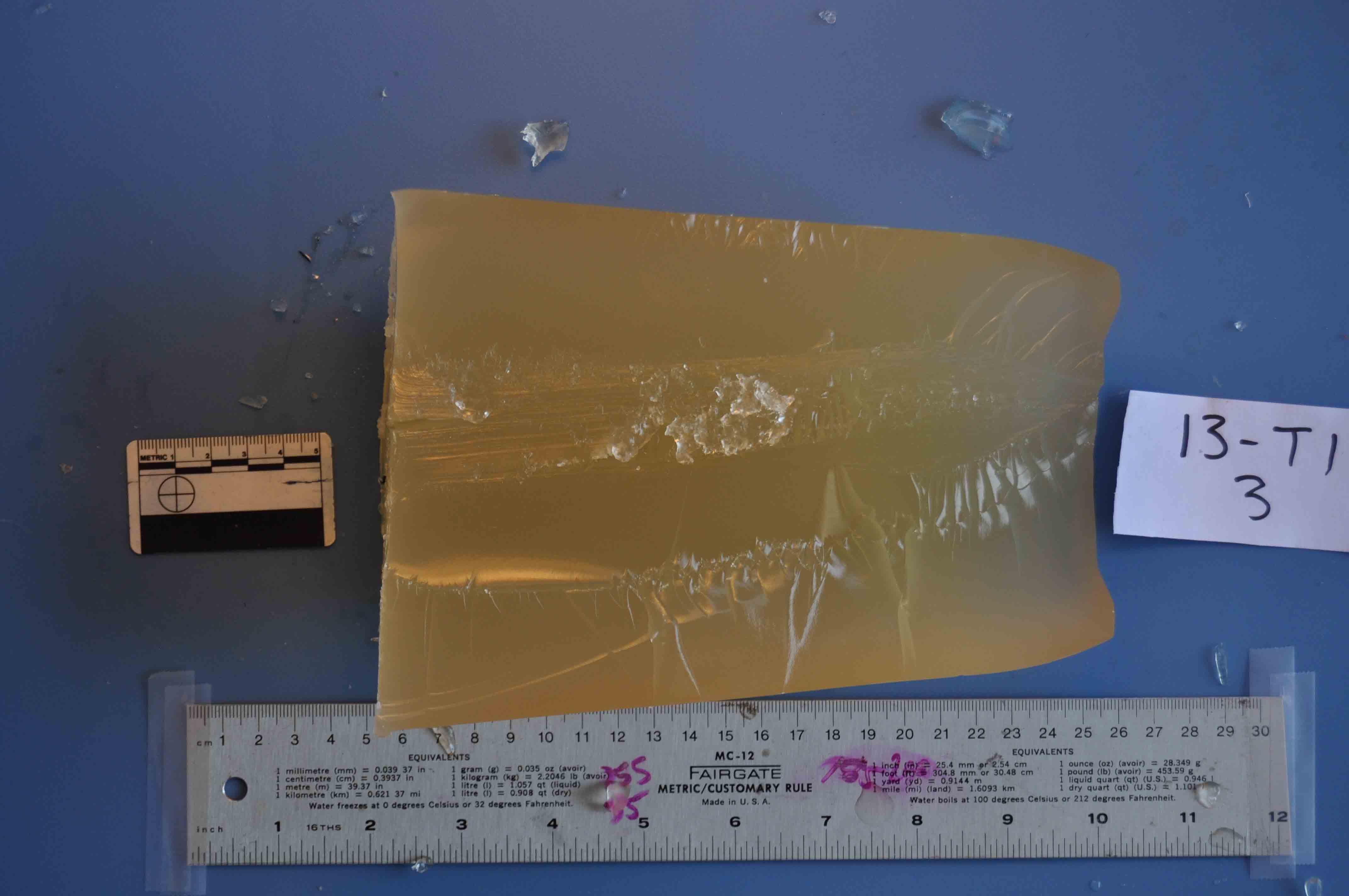

Supplement: File S2 — Wound track images, shapefiles, and tps files. (ZIP) [file pone.0104514.s002.zip › File S2/JPEGS/T1-3b.jpg]

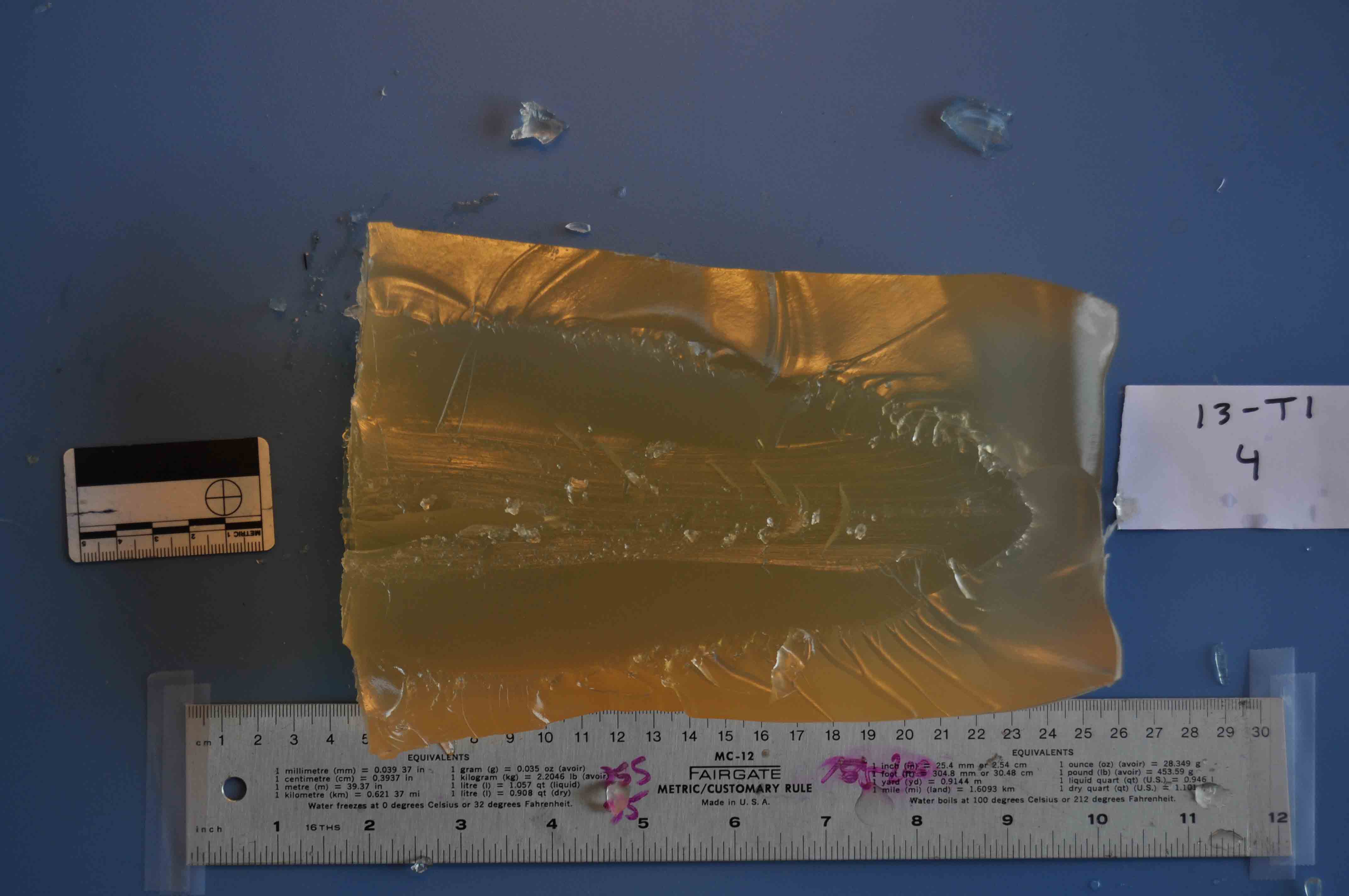

Supplement: File S2 — Wound track images, shapefiles, and tps files. (ZIP) [file pone.0104514.s002.zip › File S2/JPEGS/T1-4a.jpg]

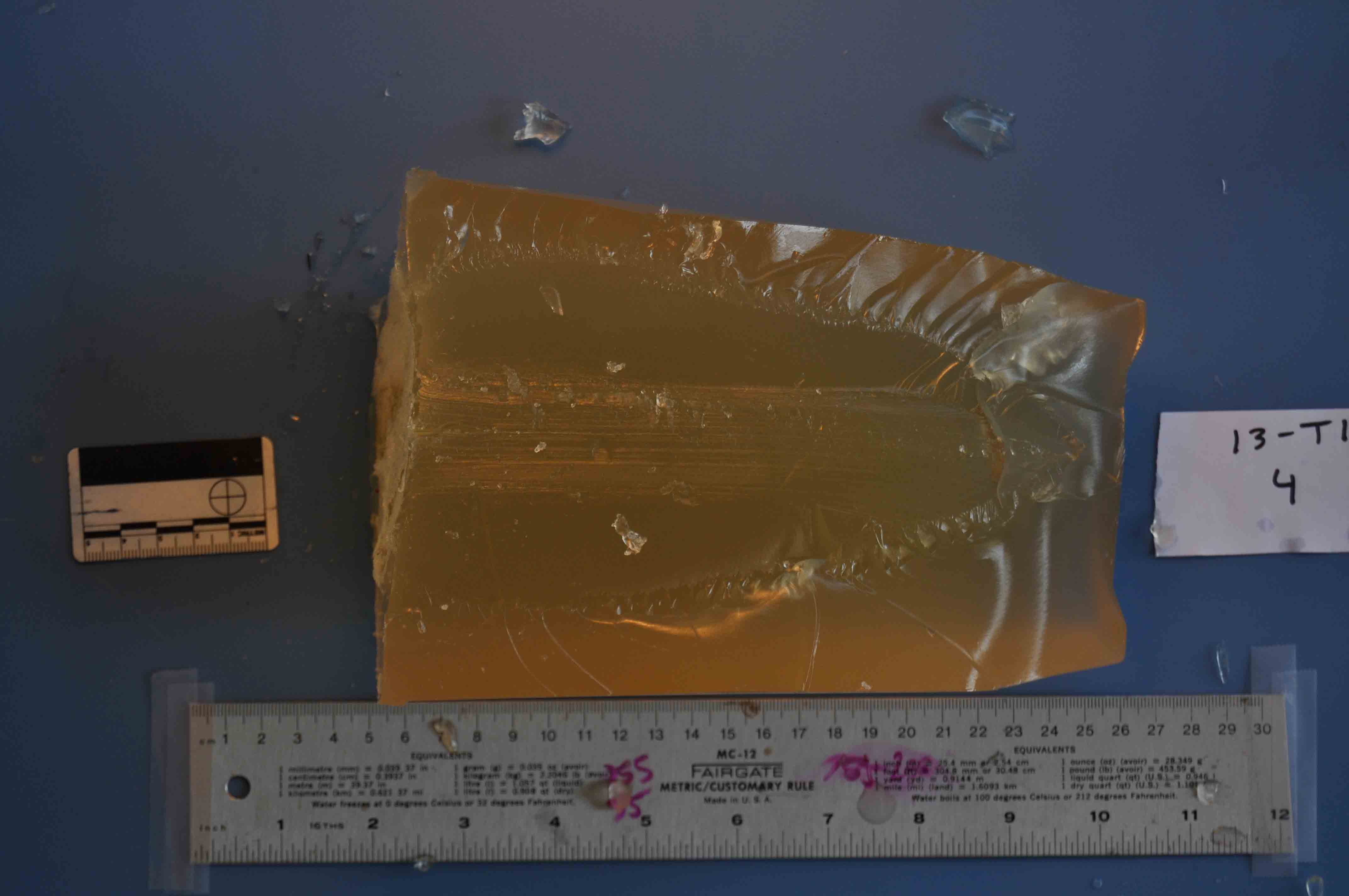

Supplement: File S2 — Wound track images, shapefiles, and tps files. (ZIP) [file pone.0104514.s002.zip › File S2/JPEGS/T1-4b.jpg]

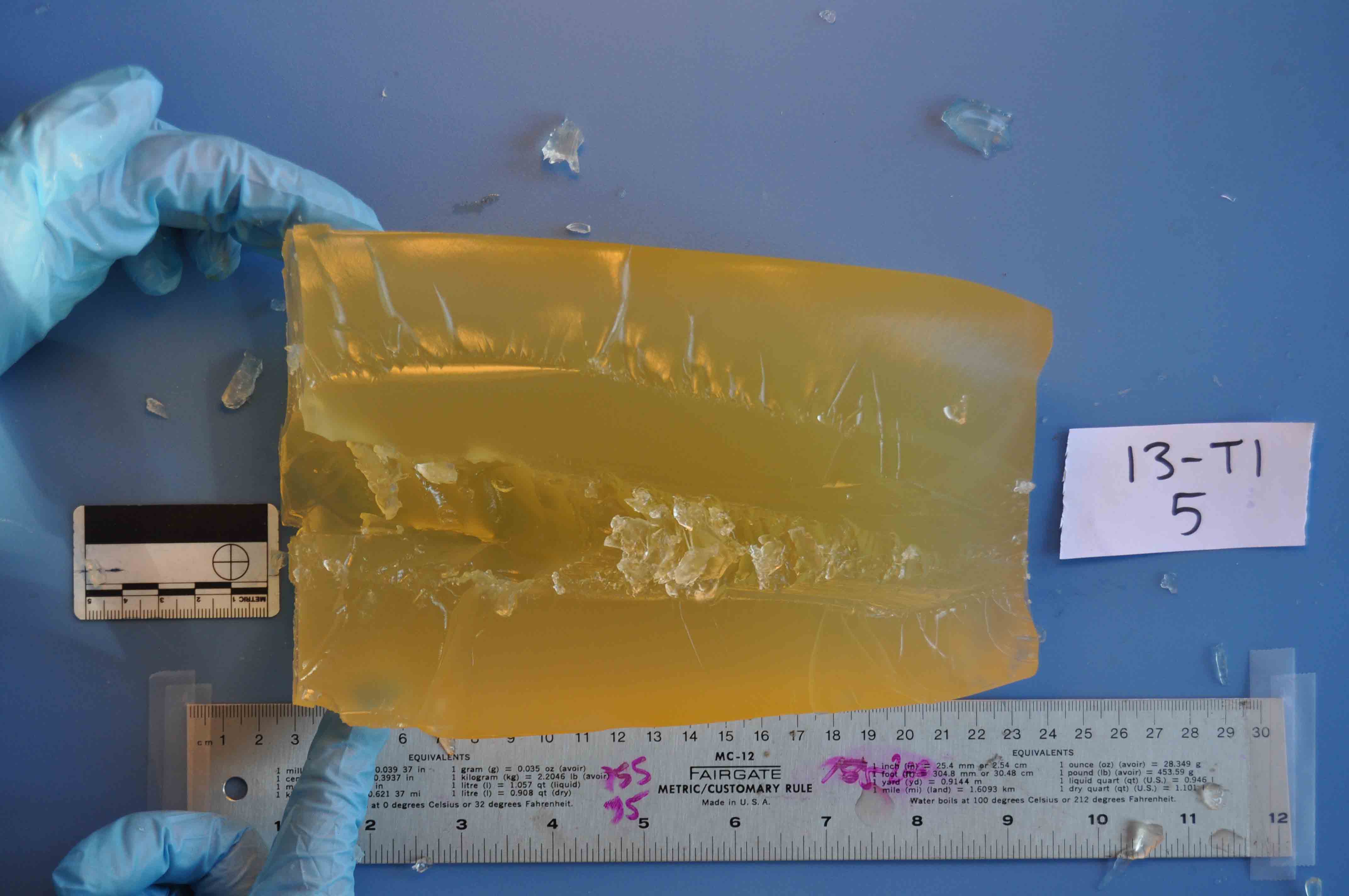

Supplement: File S2 — Wound track images, shapefiles, and tps files. (ZIP) [file pone.0104514.s002.zip › File S2/JPEGS/T1-5a.jpg]

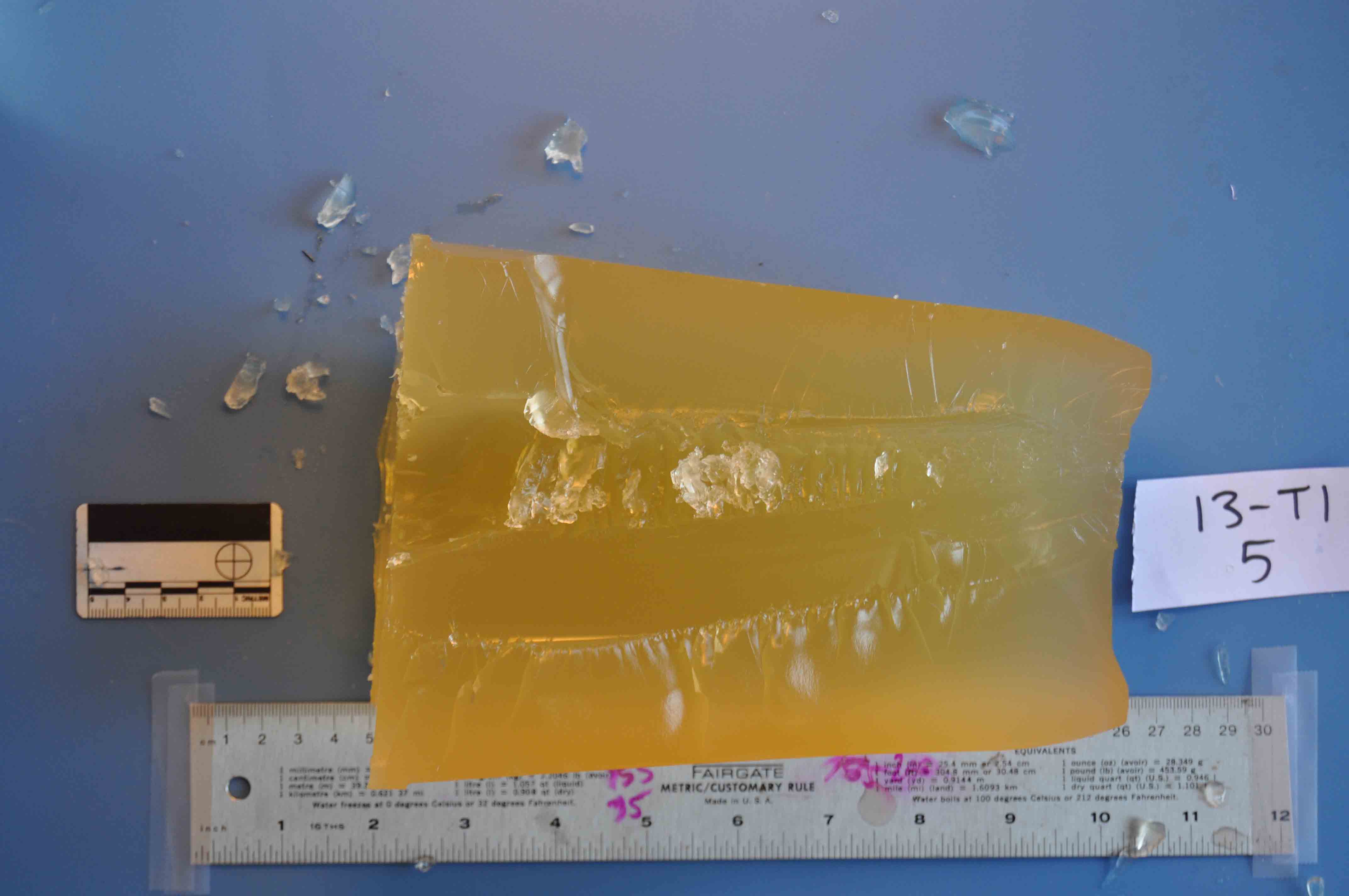

Supplement: File S2 — Wound track images, shapefiles, and tps files. (ZIP) [file pone.0104514.s002.zip › File S2/JPEGS/T1-5b.jpg]

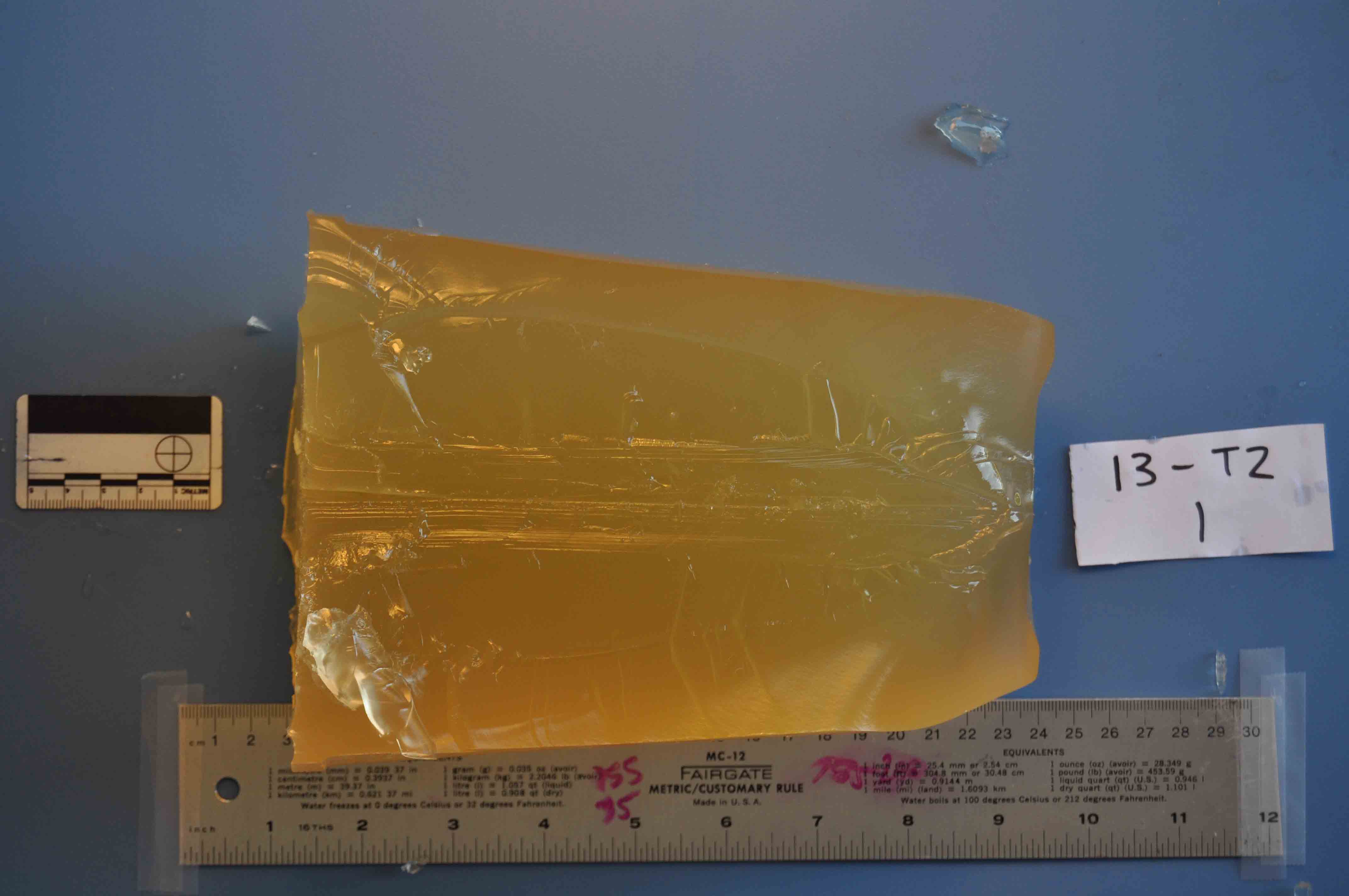

Supplement: File S2 — Wound track images, shapefiles, and tps files. (ZIP) [file pone.0104514.s002.zip › File S2/JPEGS/T2-1a.jpg]

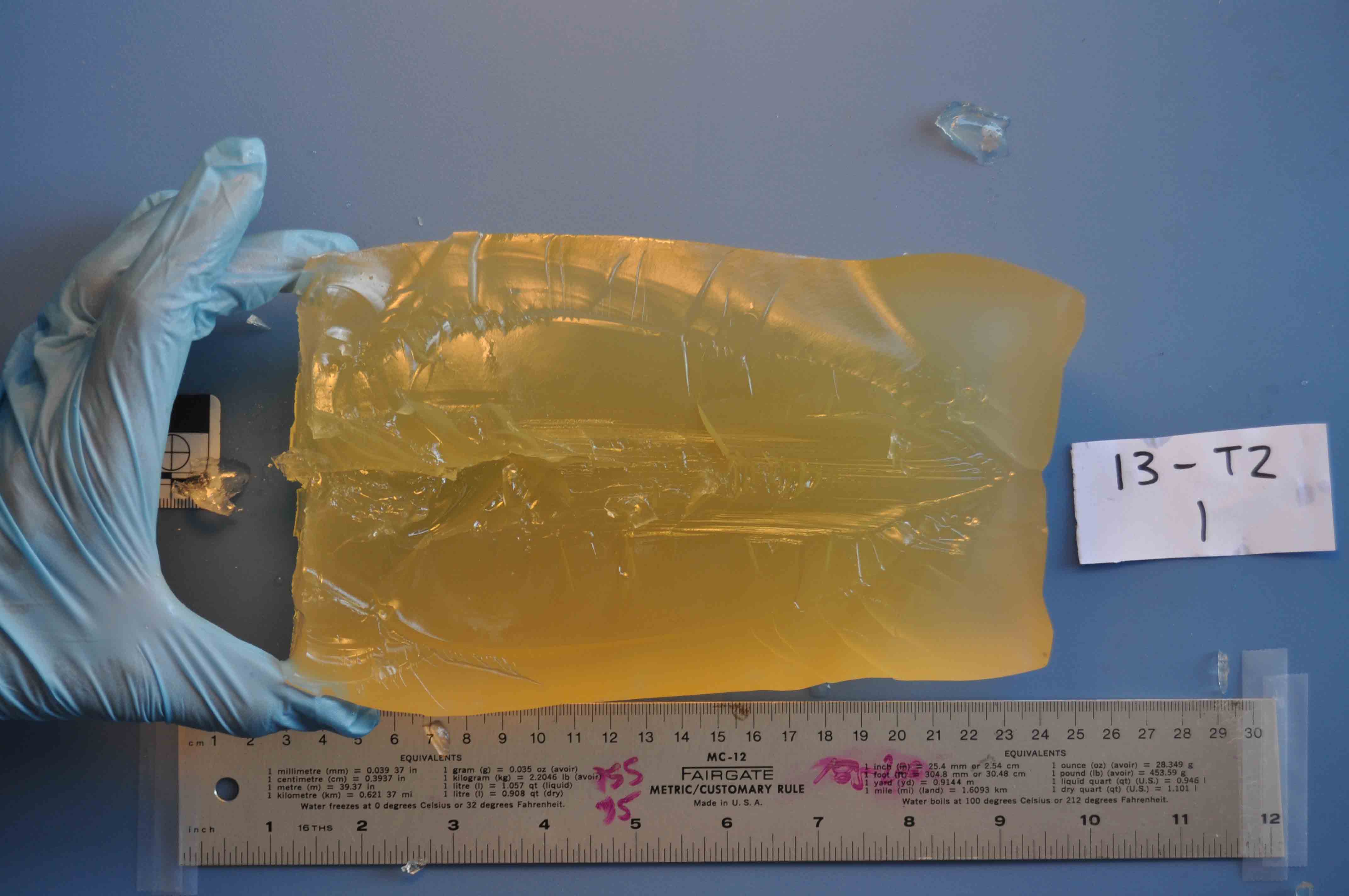

Supplement: File S2 — Wound track images, shapefiles, and tps files. (ZIP) [file pone.0104514.s002.zip › File S2/JPEGS/T2-1b.jpg]

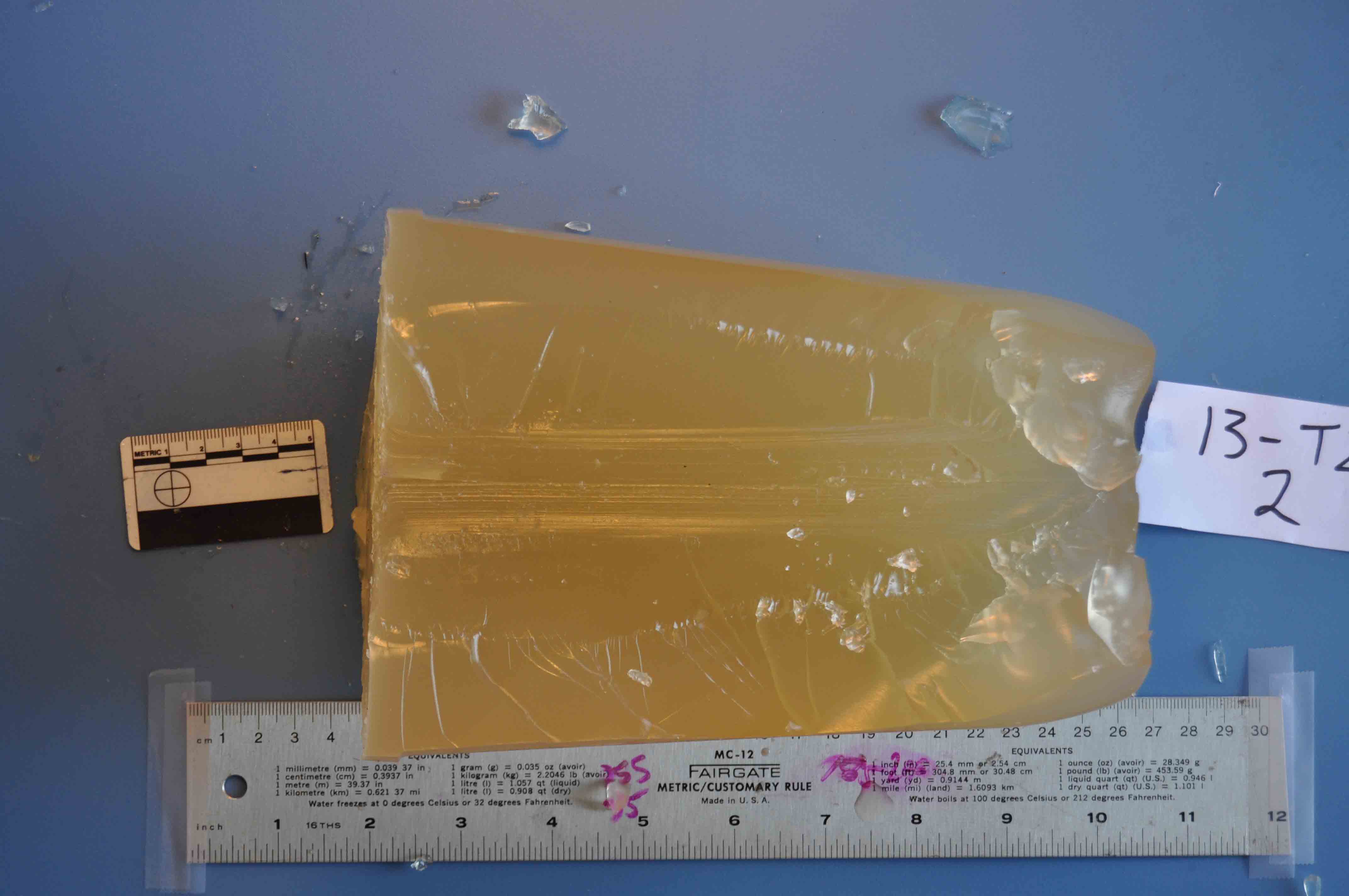

Supplement: File S2 — Wound track images, shapefiles, and tps files. (ZIP) [file pone.0104514.s002.zip › File S2/JPEGS/T2-2a.jpg]

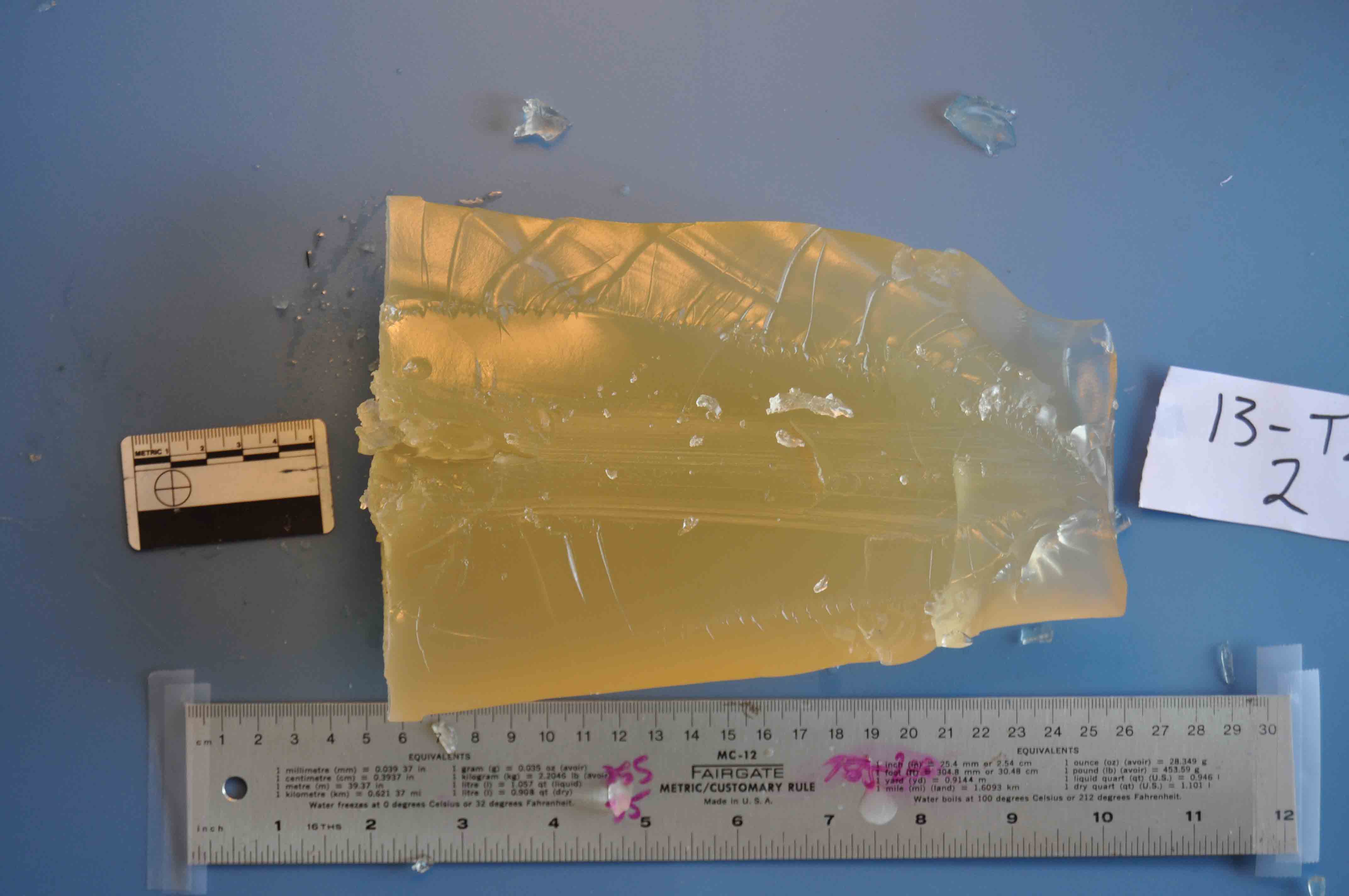

Supplement: File S2 — Wound track images, shapefiles, and tps files. (ZIP) [file pone.0104514.s002.zip › File S2/JPEGS/T2-2b.jpg]

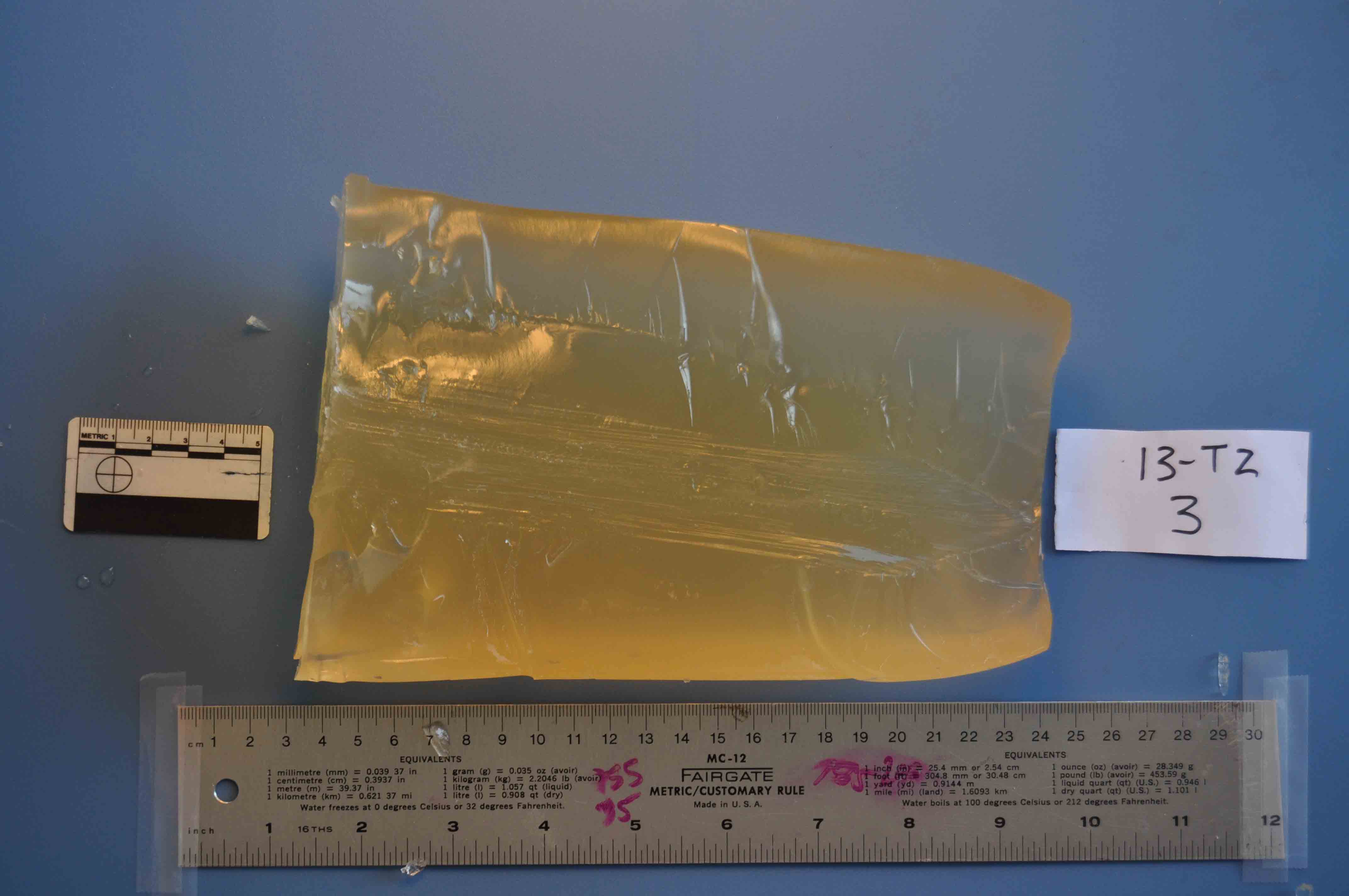

Supplement: File S2 — Wound track images, shapefiles, and tps files. (ZIP) [file pone.0104514.s002.zip › File S2/JPEGS/T2-3a.jpg]

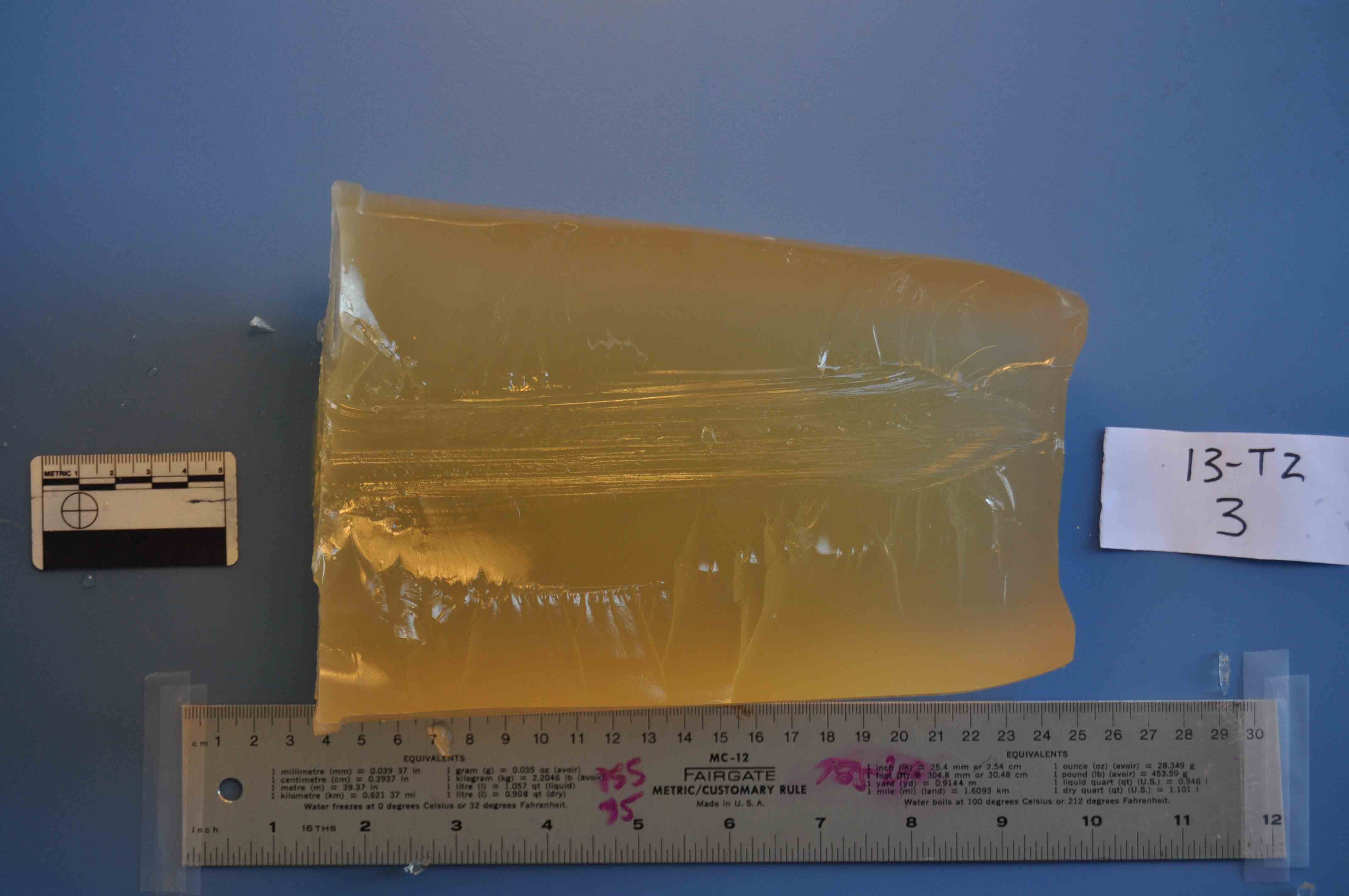

Supplement: File S2 — Wound track images, shapefiles, and tps files. (ZIP) [file pone.0104514.s002.zip › File S2/JPEGS/T2-3b.jpg]

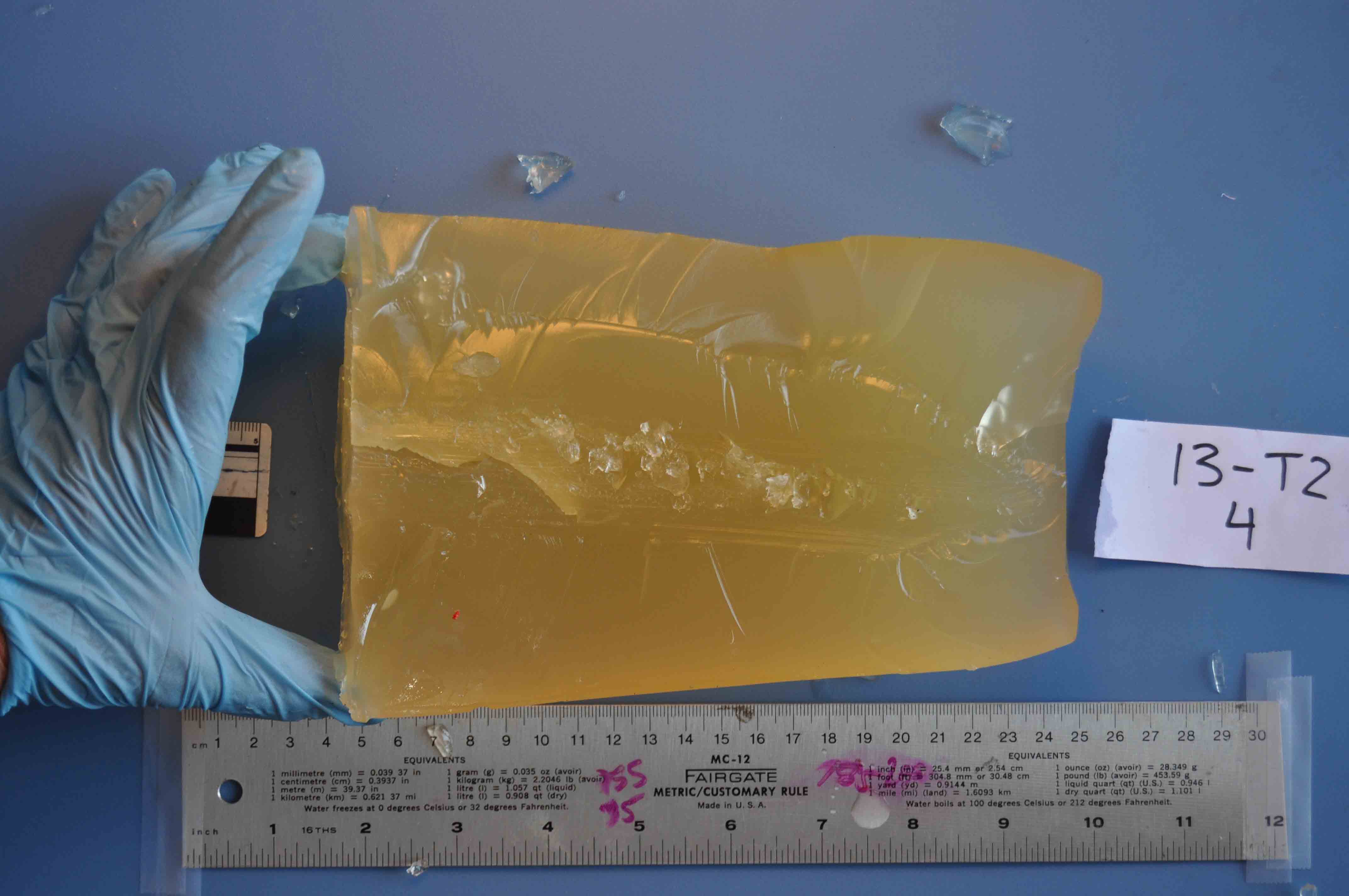

Supplement: File S2 — Wound track images, shapefiles, and tps files. (ZIP) [file pone.0104514.s002.zip › File S2/JPEGS/T2-4a.jpg]

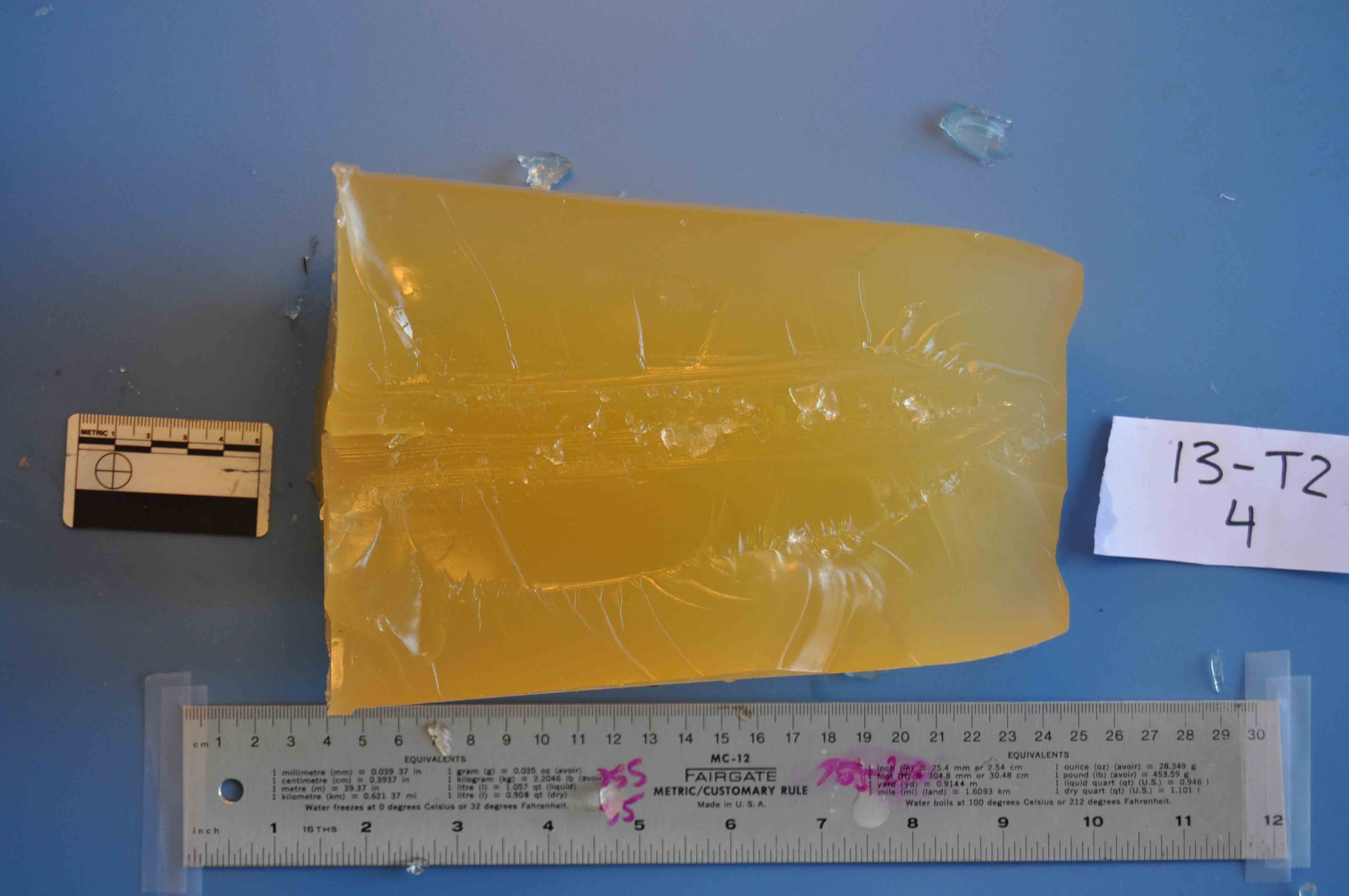

Supplement: File S2 — Wound track images, shapefiles, and tps files. (ZIP) [file pone.0104514.s002.zip › File S2/JPEGS/T2-4b.jpg]

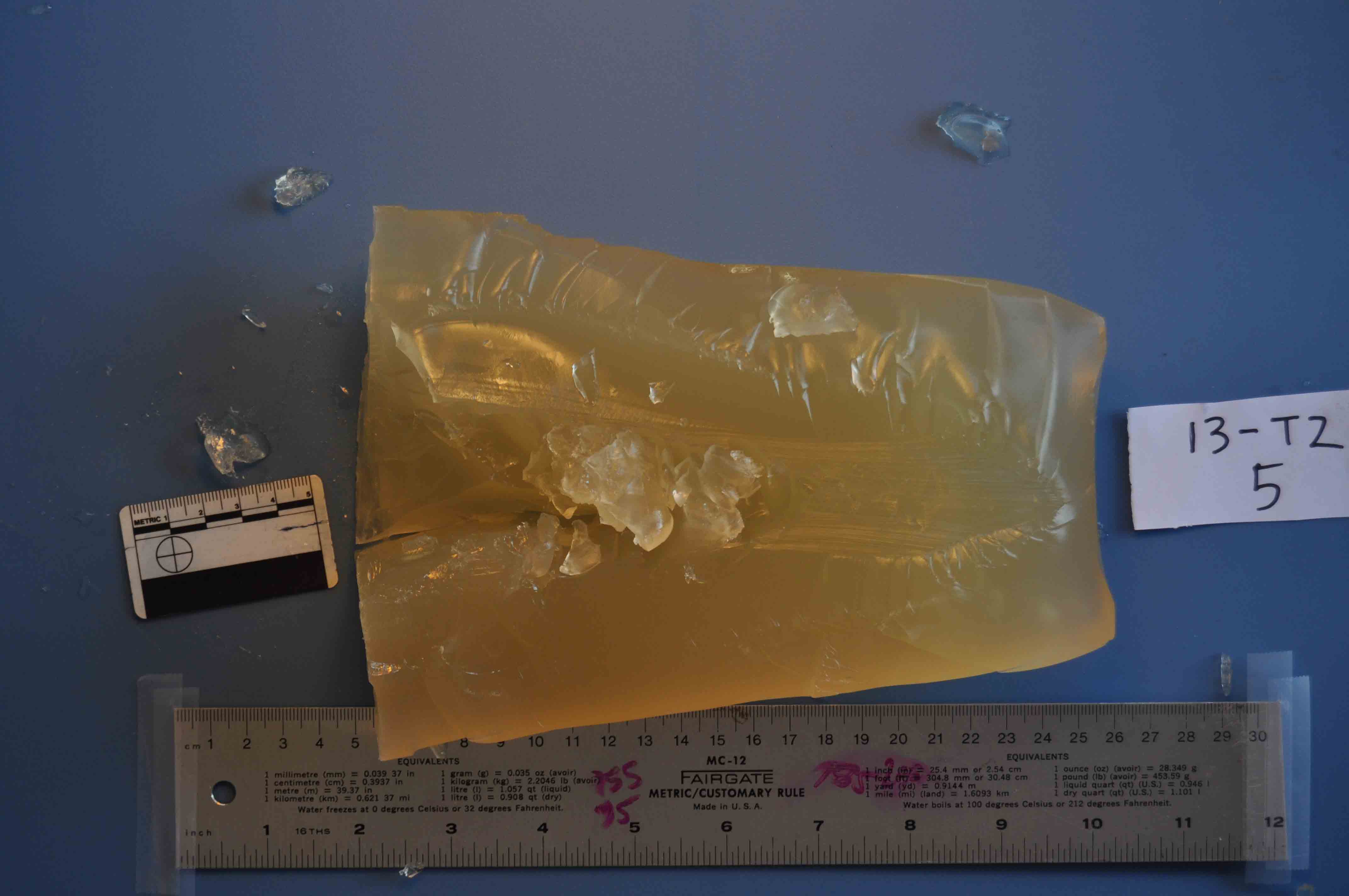

Supplement: File S2 — Wound track images, shapefiles, and tps files. (ZIP) [file pone.0104514.s002.zip › File S2/JPEGS/T2-5a.jpg]

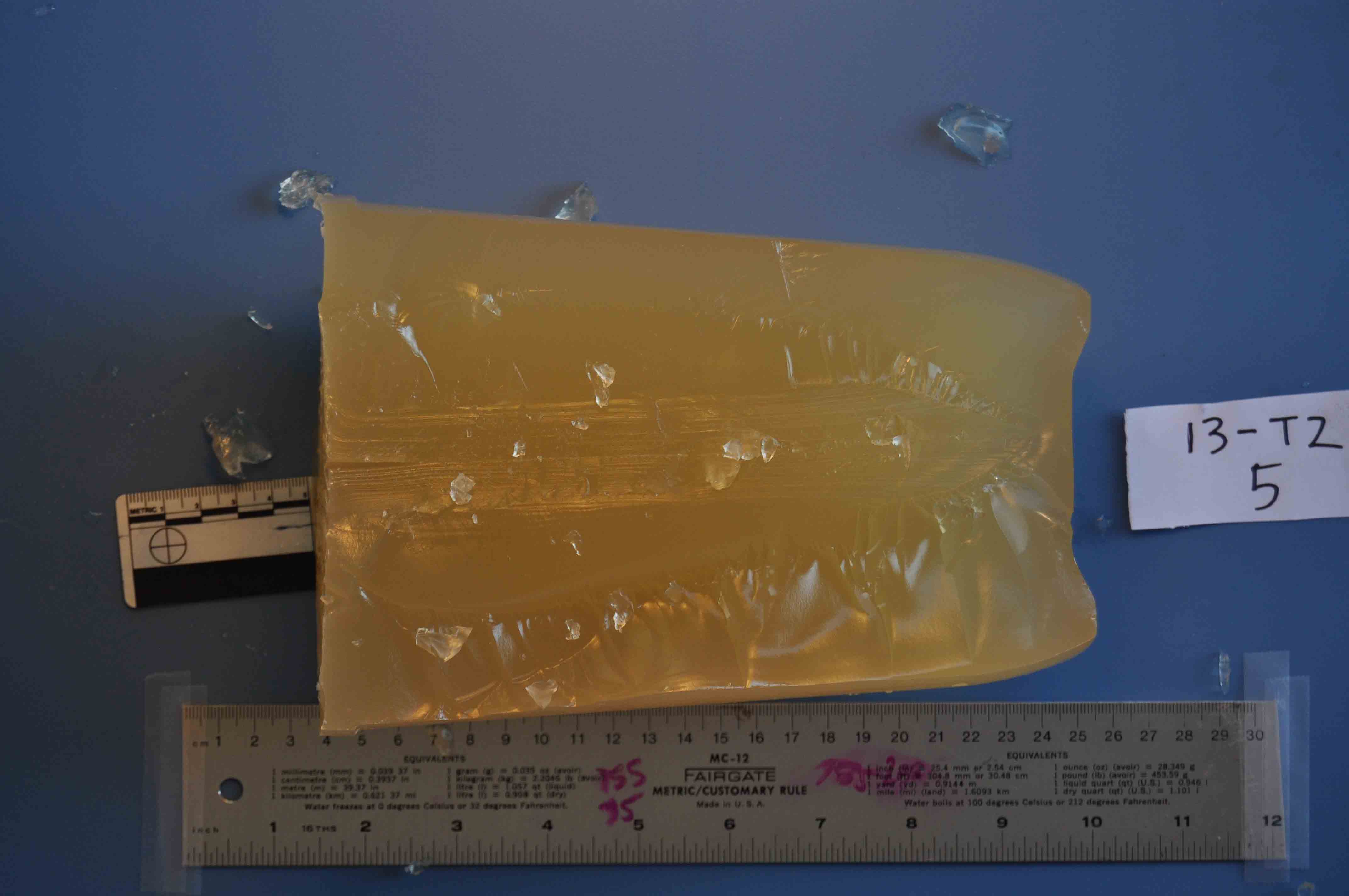

Supplement: File S2 — Wound track images, shapefiles, and tps files. (ZIP) [file pone.0104514.s002.zip › File S2/JPEGS/T2-5b.jpg]

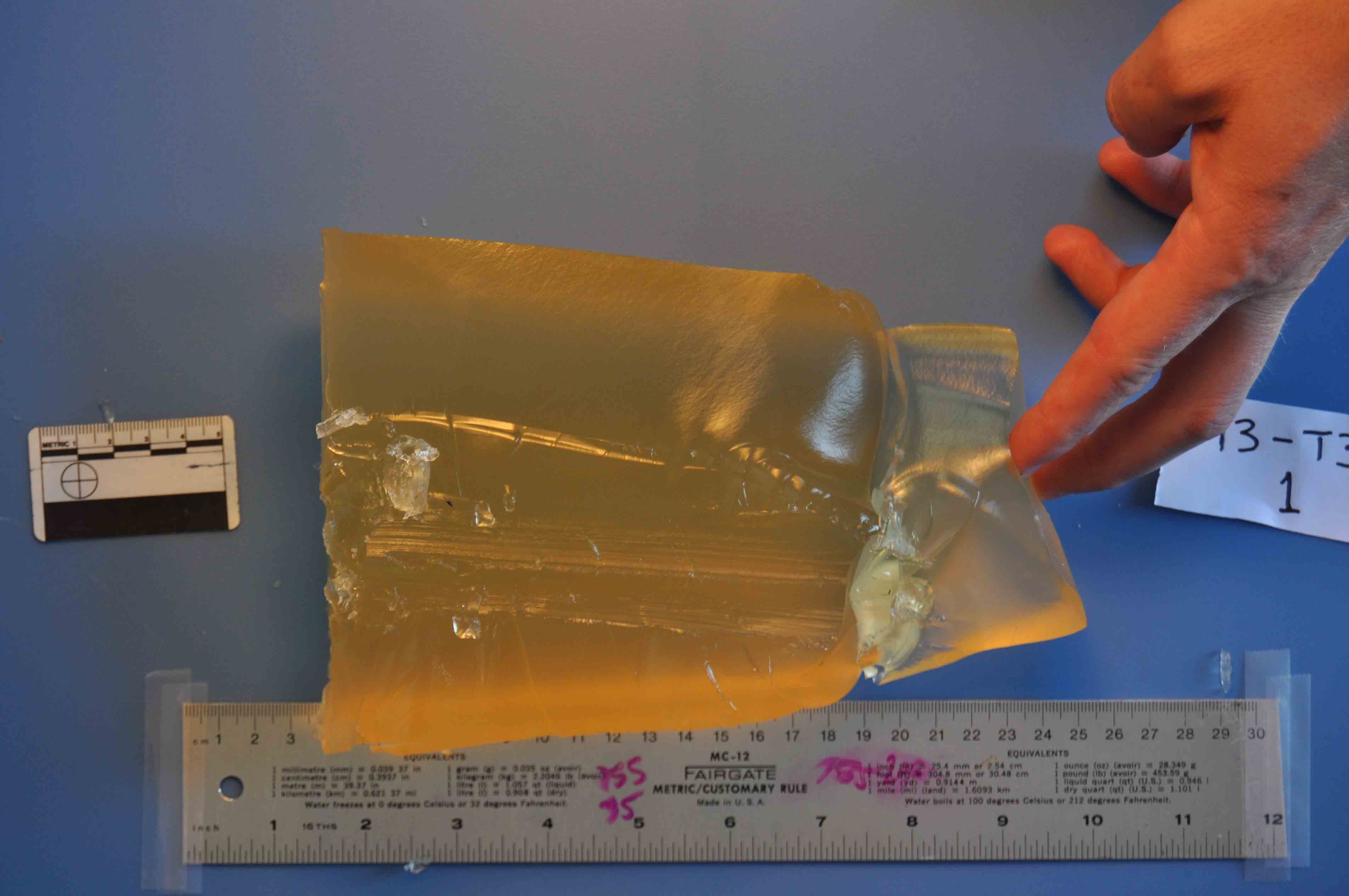

Supplement: File S2 — Wound track images, shapefiles, and tps files. (ZIP) [file pone.0104514.s002.zip › File S2/JPEGS/T3-1a.jpg]

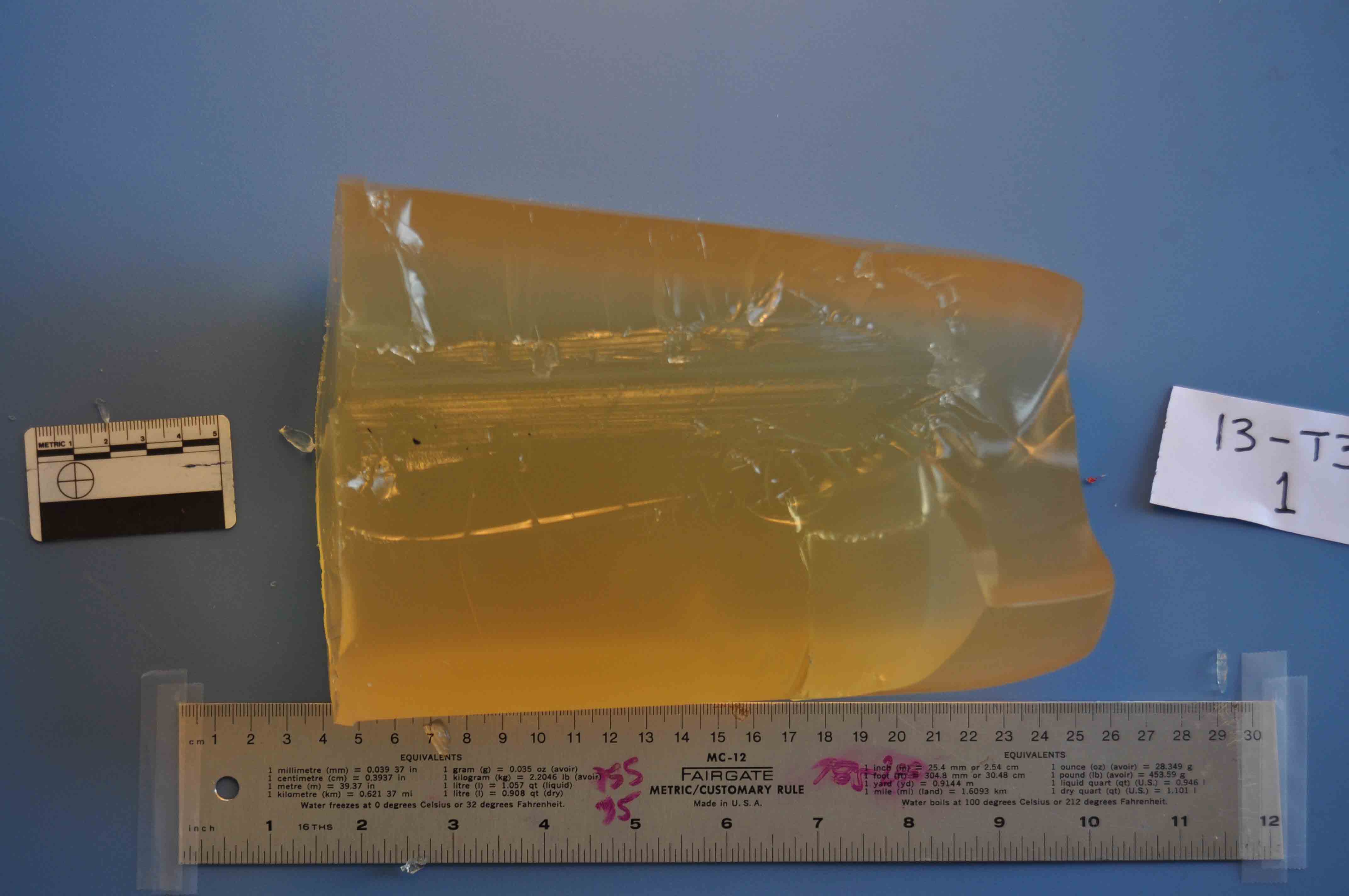

Supplement: File S2 — Wound track images, shapefiles, and tps files. (ZIP) [file pone.0104514.s002.zip › File S2/JPEGS/T3-1b.jpg]

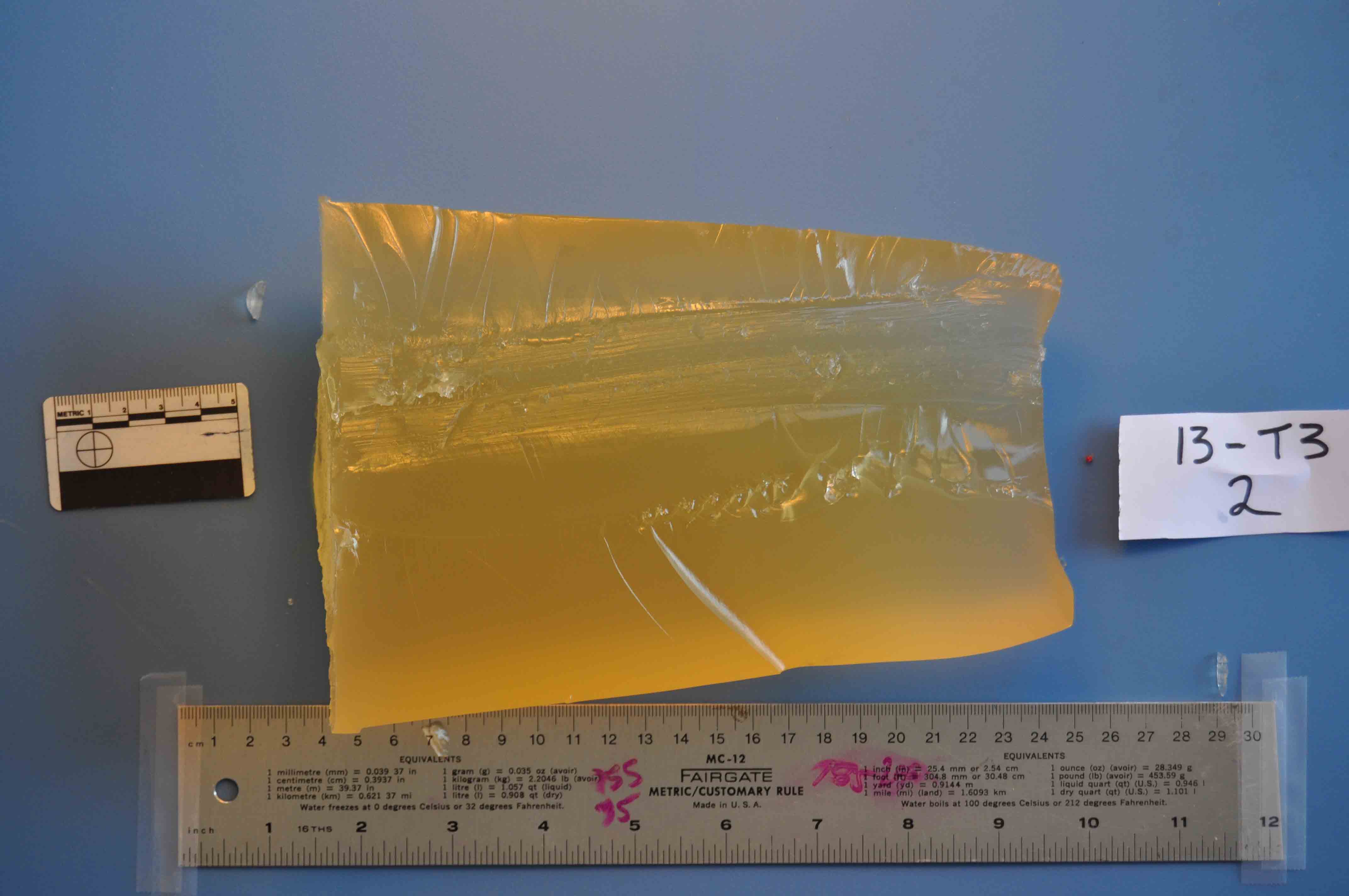

Supplement: File S2 — Wound track images, shapefiles, and tps files. (ZIP) [file pone.0104514.s002.zip › File S2/JPEGS/T3-2a.jpg]

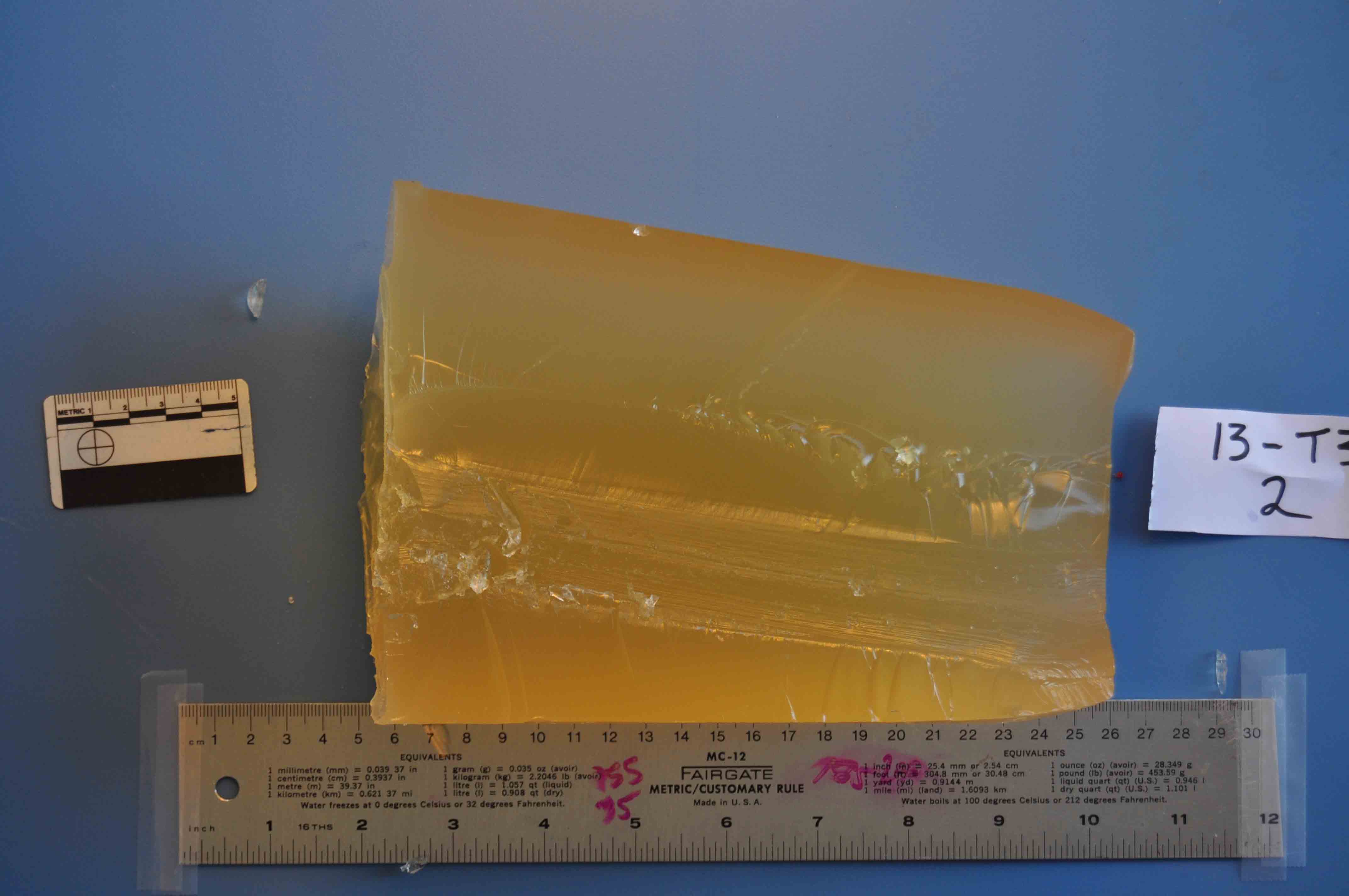

Supplement: File S2 — Wound track images, shapefiles, and tps files. (ZIP) [file pone.0104514.s002.zip › File S2/JPEGS/T3-2b.jpg]

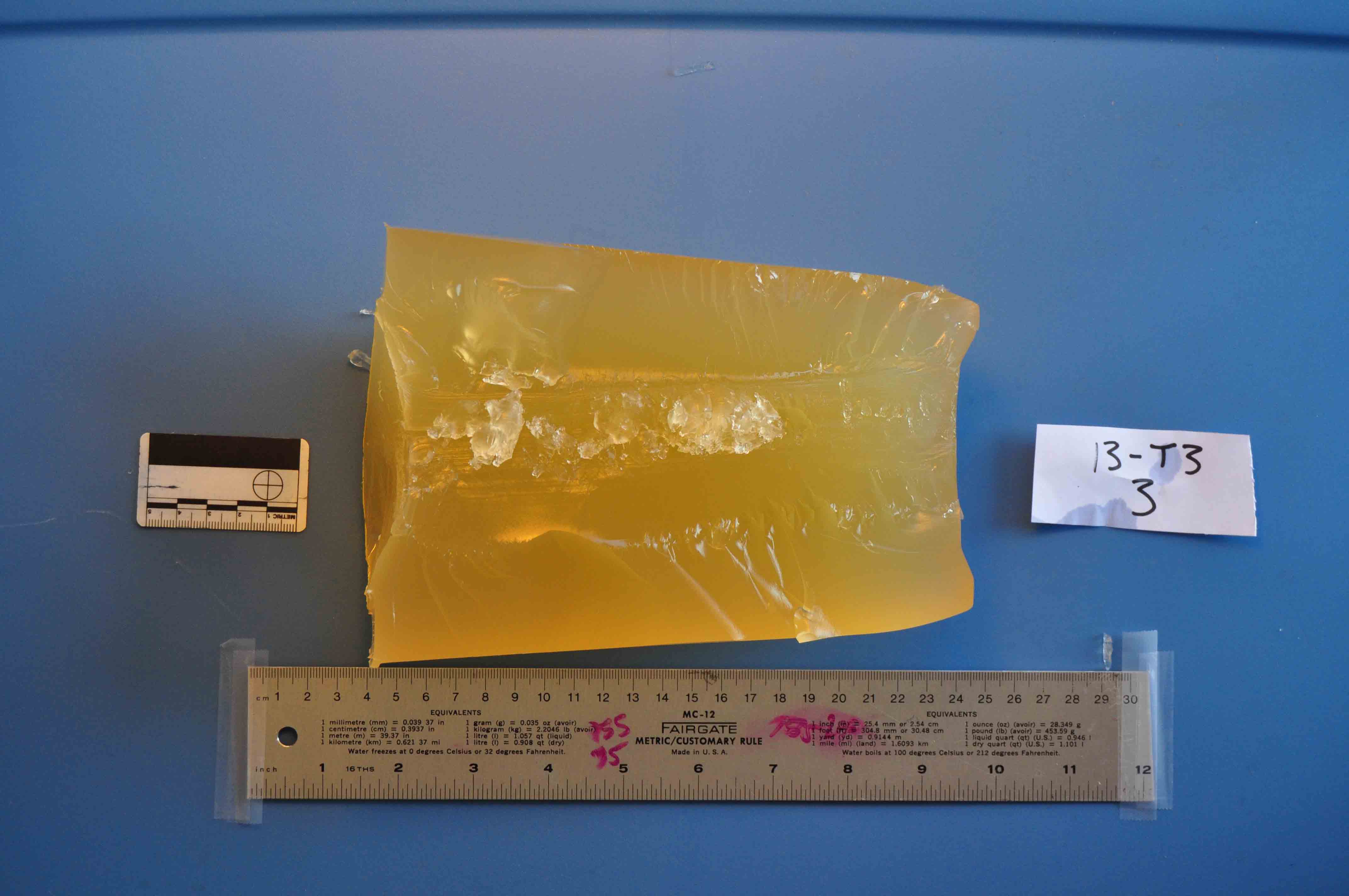

Supplement: File S2 — Wound track images, shapefiles, and tps files. (ZIP) [file pone.0104514.s002.zip › File S2/JPEGS/T3-3a.jpg]

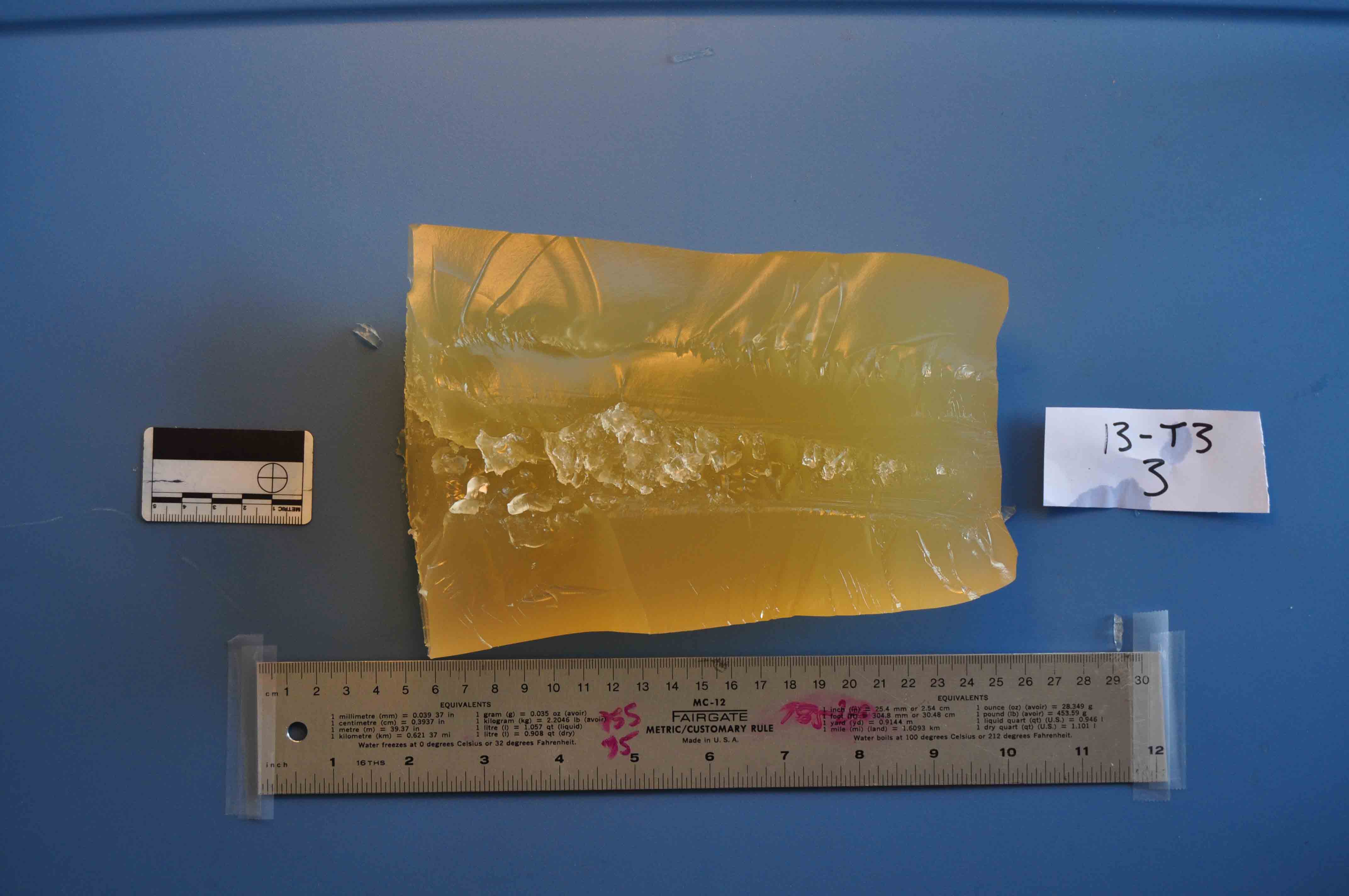

Supplement: File S2 — Wound track images, shapefiles, and tps files. (ZIP) [file pone.0104514.s002.zip › File S2/JPEGS/T3-3b.jpg]

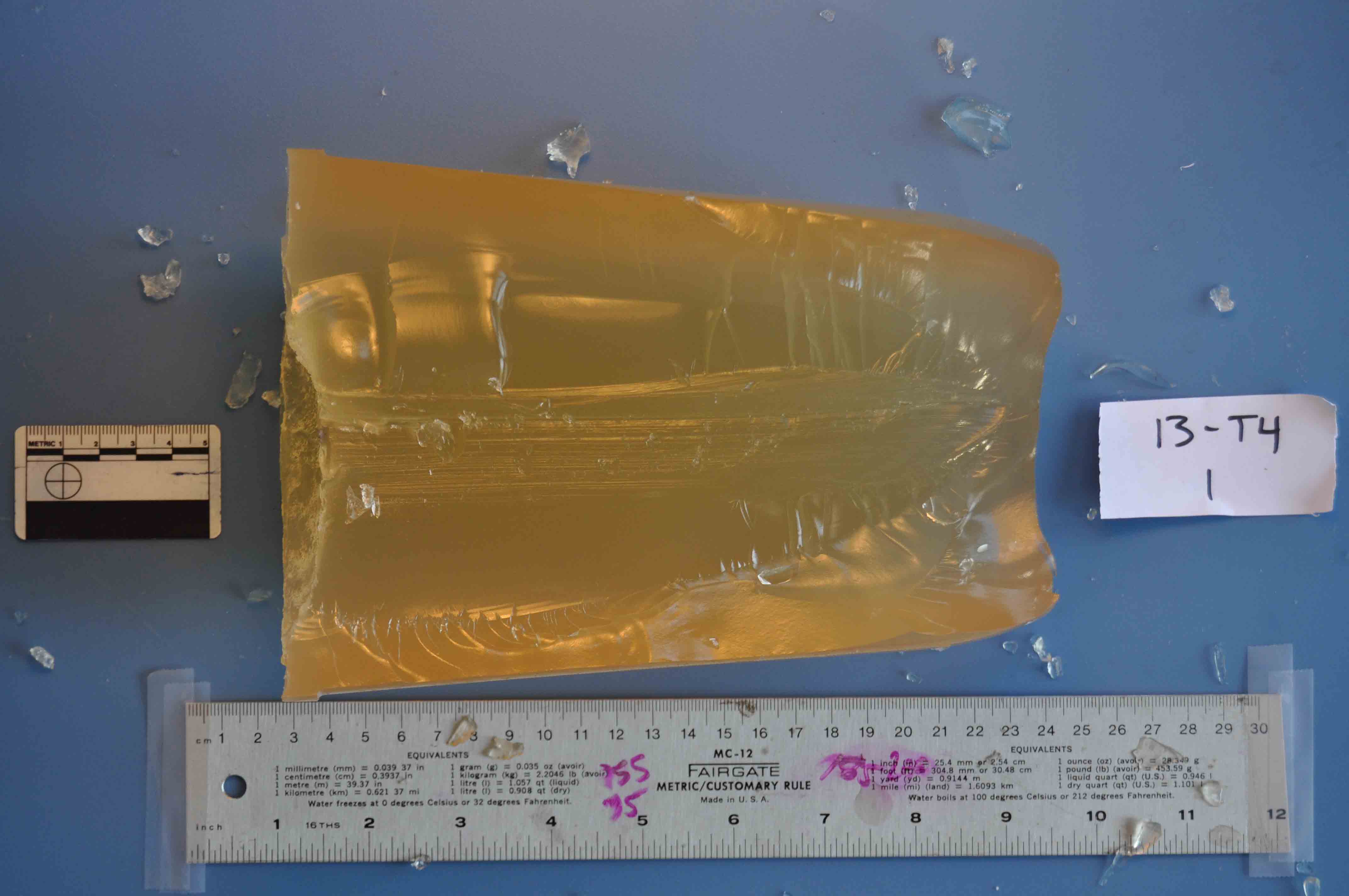

Supplement: File S2 — Wound track images, shapefiles, and tps files. (ZIP) [file pone.0104514.s002.zip › File S2/JPEGS/T4-1a.jpg]

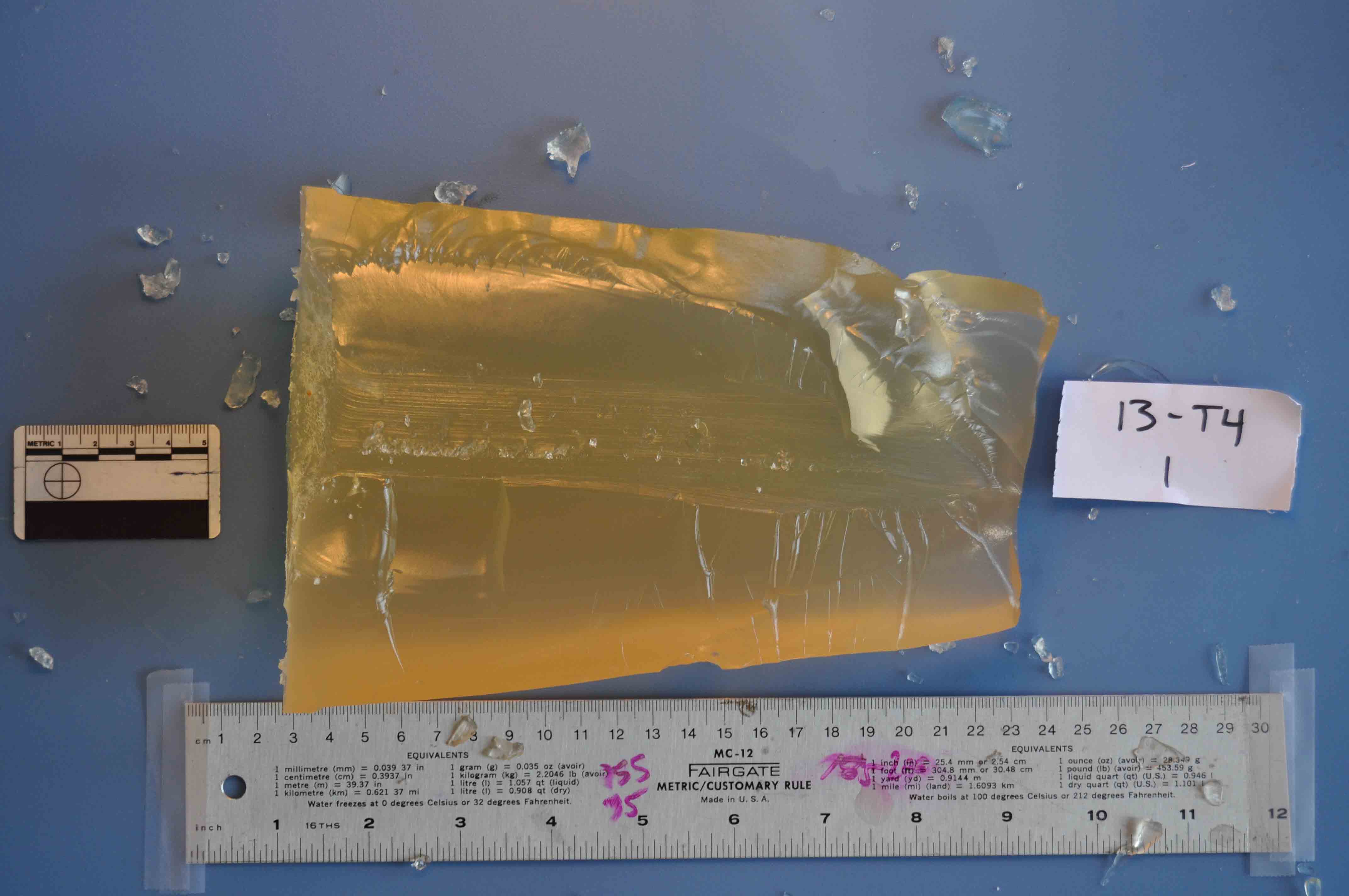

Supplement: File S2 — Wound track images, shapefiles, and tps files. (ZIP) [file pone.0104514.s002.zip › File S2/JPEGS/T4-1b.jpg]

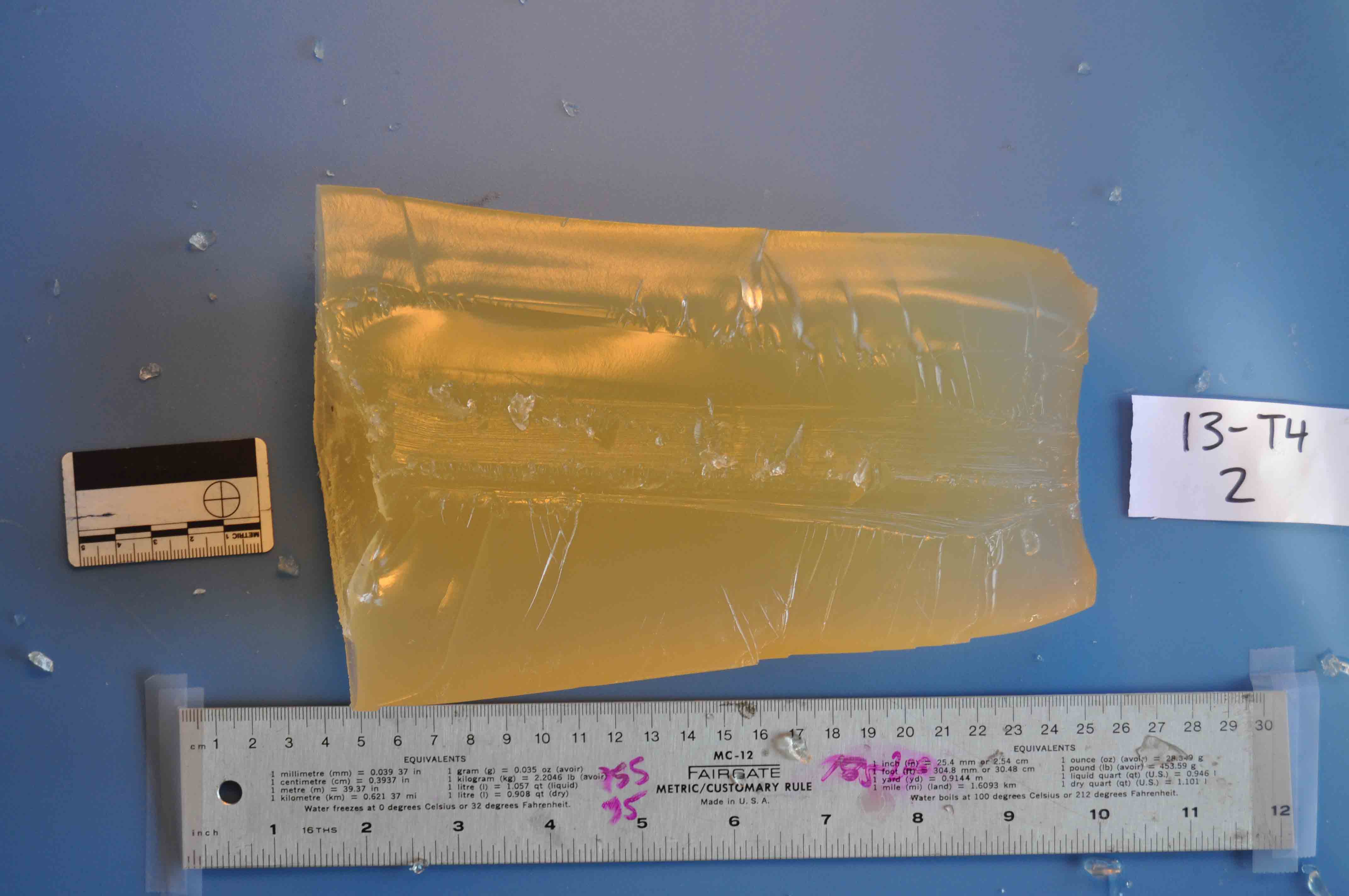

Supplement: File S2 — Wound track images, shapefiles, and tps files. (ZIP) [file pone.0104514.s002.zip › File S2/JPEGS/T4-2a.jpg]

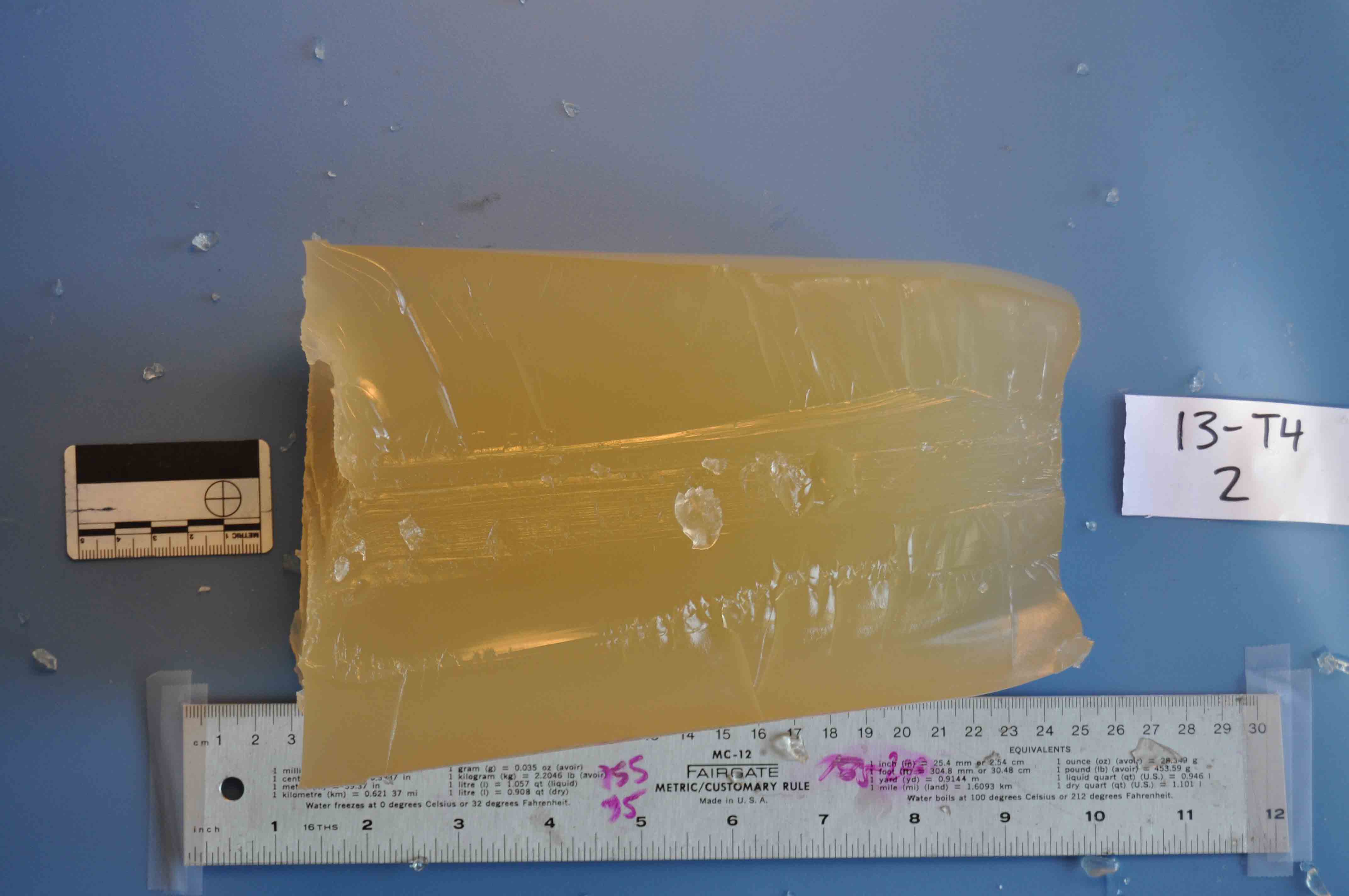

Supplement: File S2 — Wound track images, shapefiles, and tps files. (ZIP) [file pone.0104514.s002.zip › File S2/JPEGS/T4-2b.jpg]

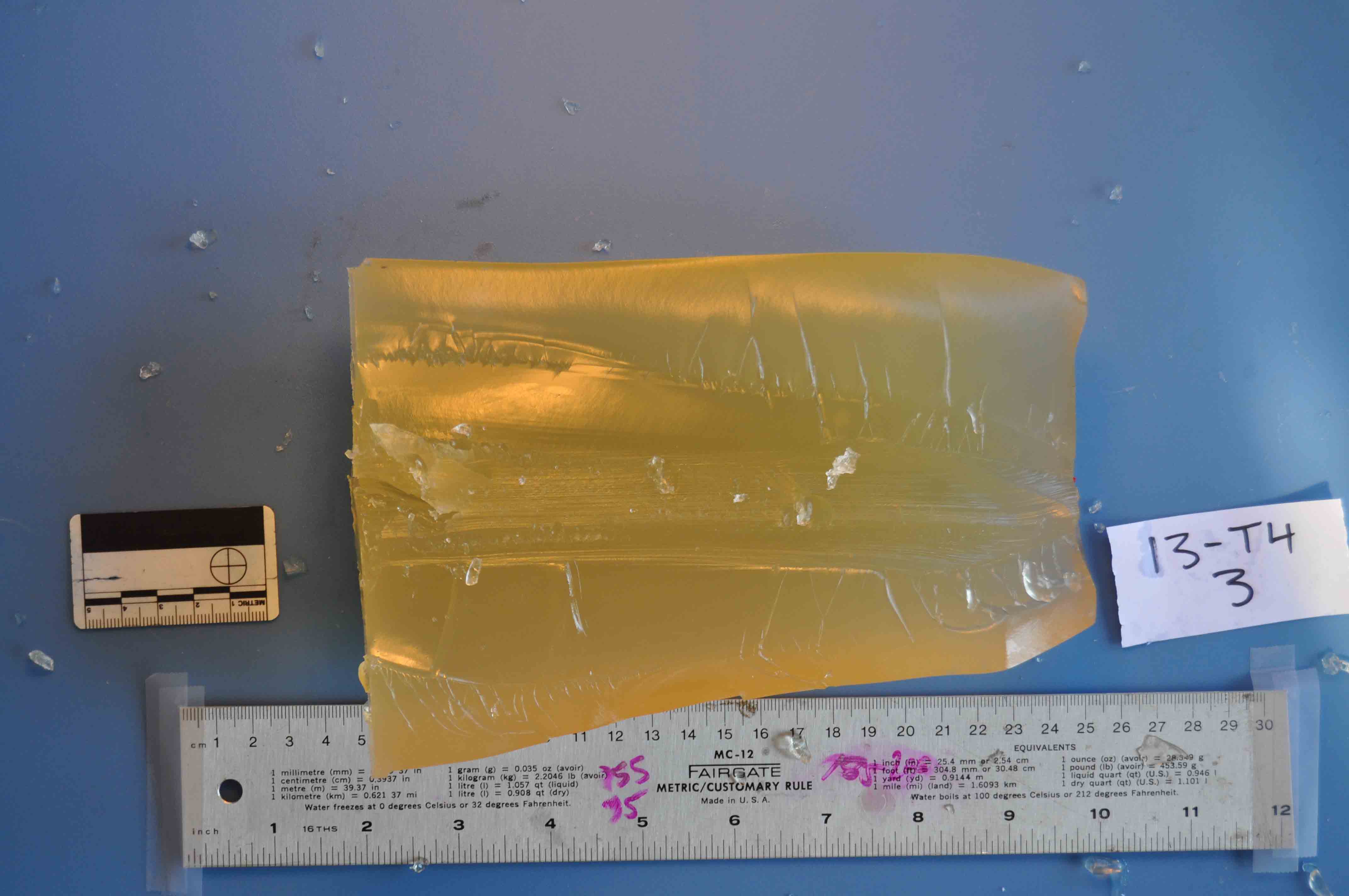

Supplement: File S2 — Wound track images, shapefiles, and tps files. (ZIP) [file pone.0104514.s002.zip › File S2/JPEGS/T4-3a.jpg]

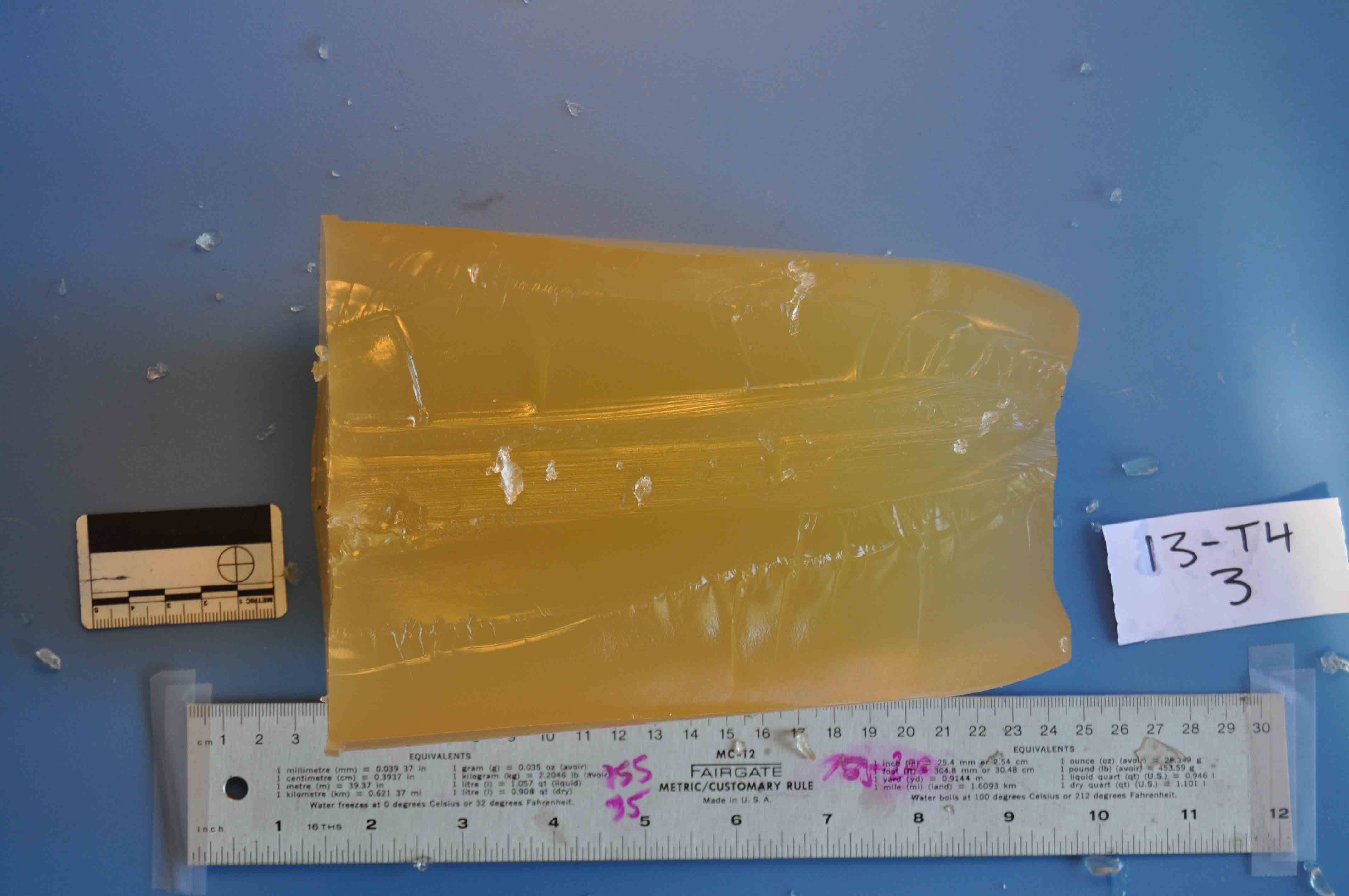

Supplement: File S2 — Wound track images, shapefiles, and tps files. (ZIP) [file pone.0104514.s002.zip › File S2/JPEGS/T4-3b.jpg]

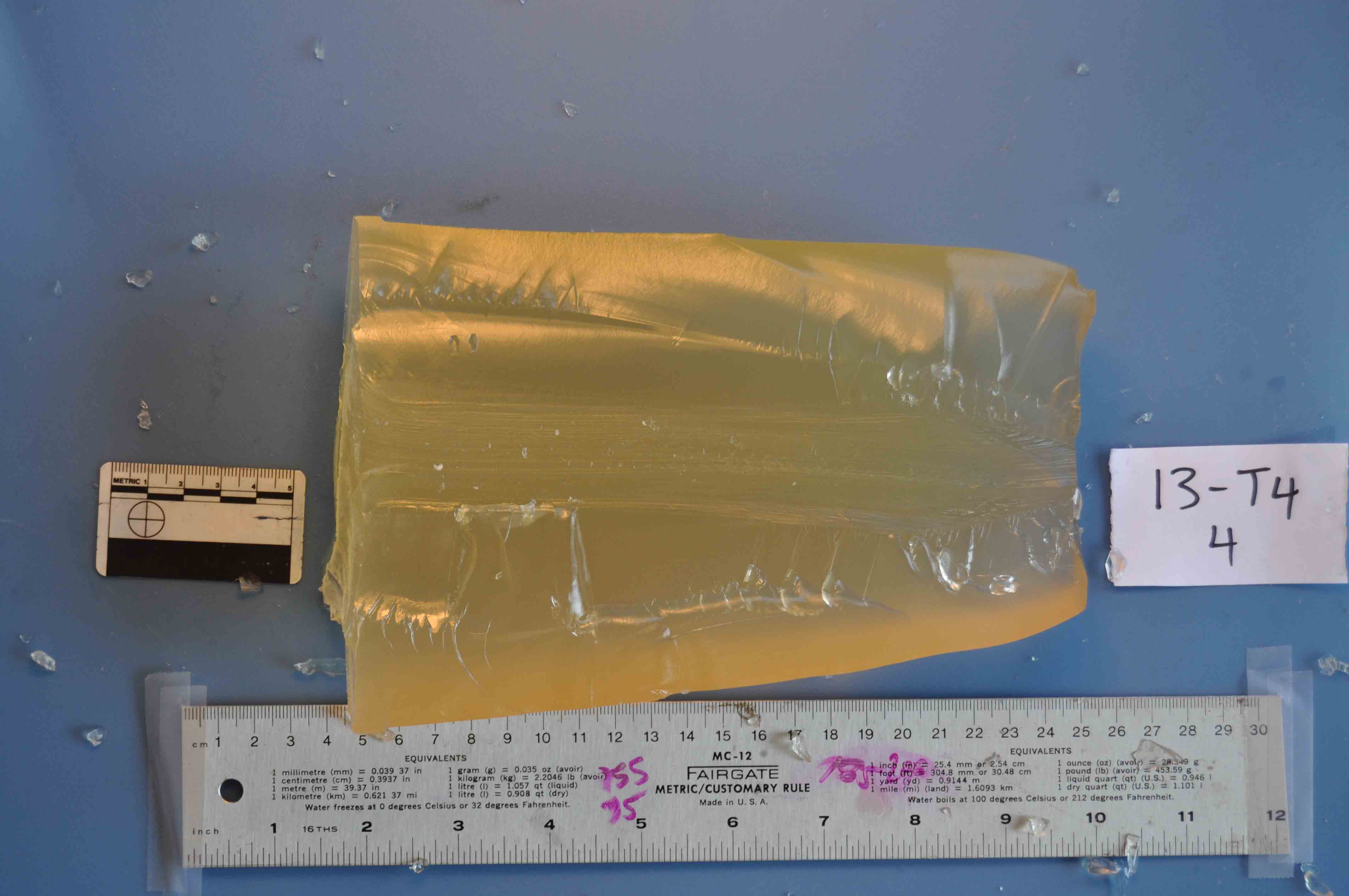

Supplement: File S2 — Wound track images, shapefiles, and tps files. (ZIP) [file pone.0104514.s002.zip › File S2/JPEGS/T4-4a.jpg]

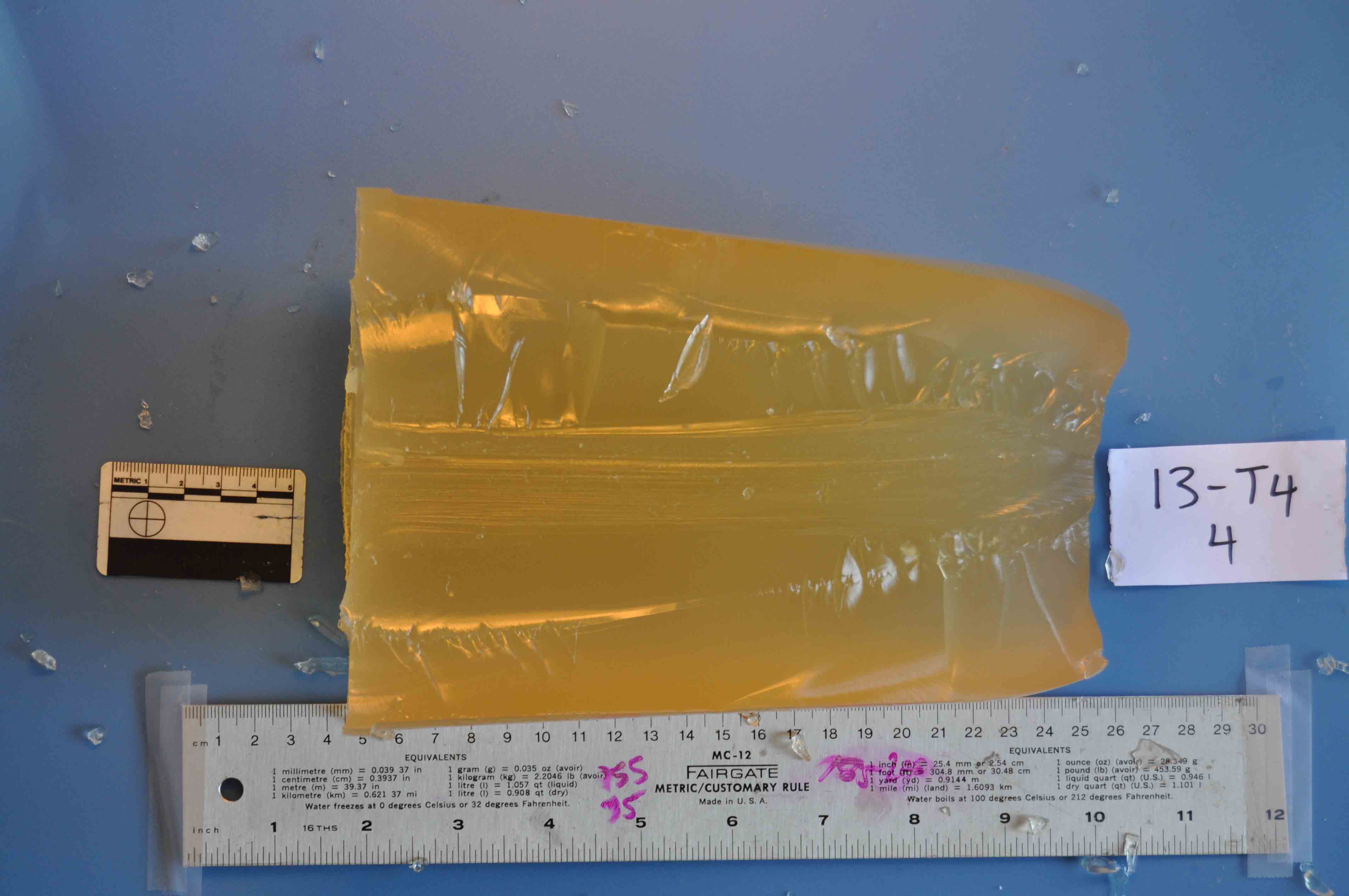

Supplement: File S2 — Wound track images, shapefiles, and tps files. (ZIP) [file pone.0104514.s002.zip › File S2/JPEGS/T4-4b.jpg]

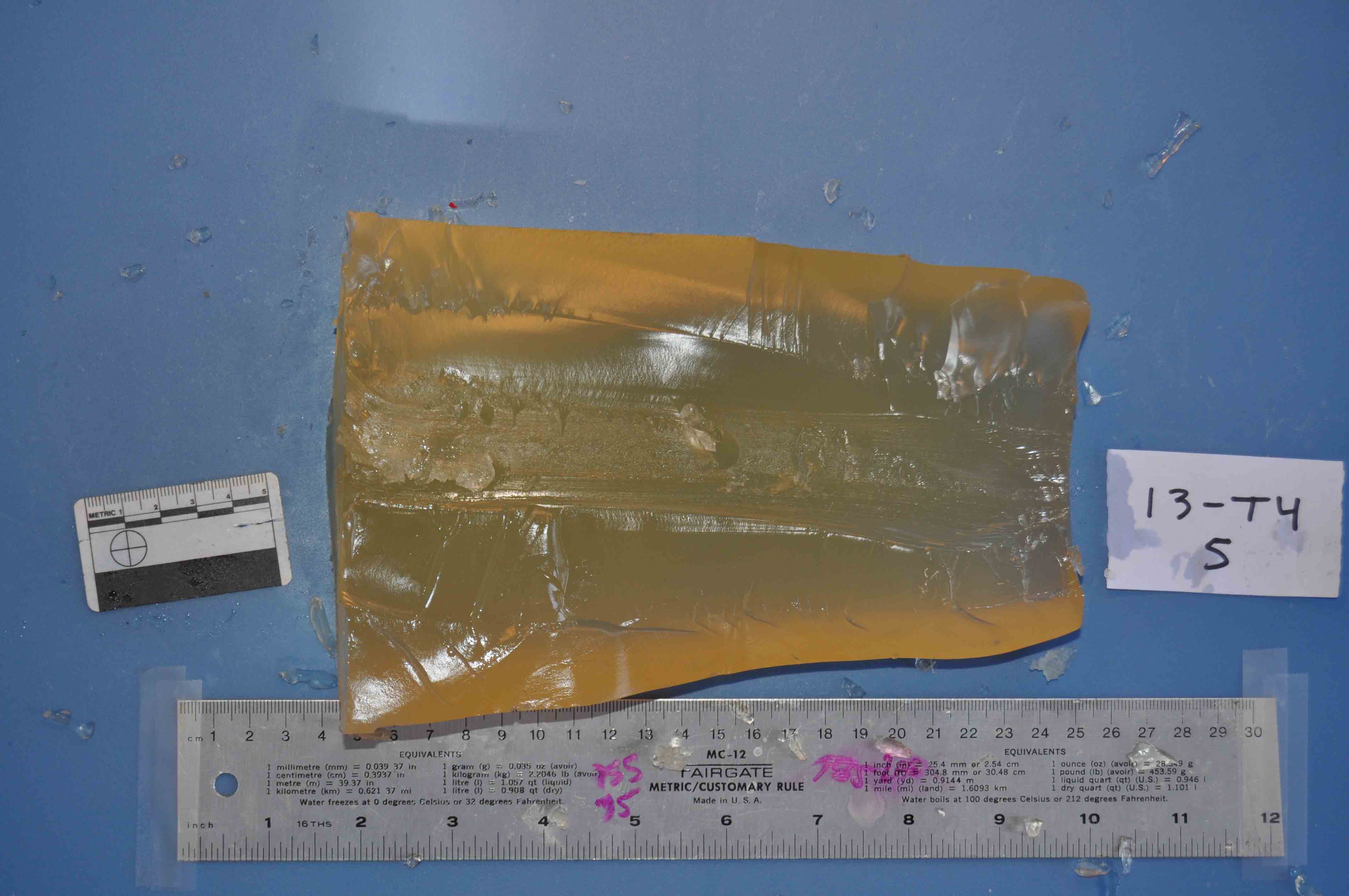

Supplement: File S2 — Wound track images, shapefiles, and tps files. (ZIP) [file pone.0104514.s002.zip › File S2/JPEGS/T4-5a.jpg]

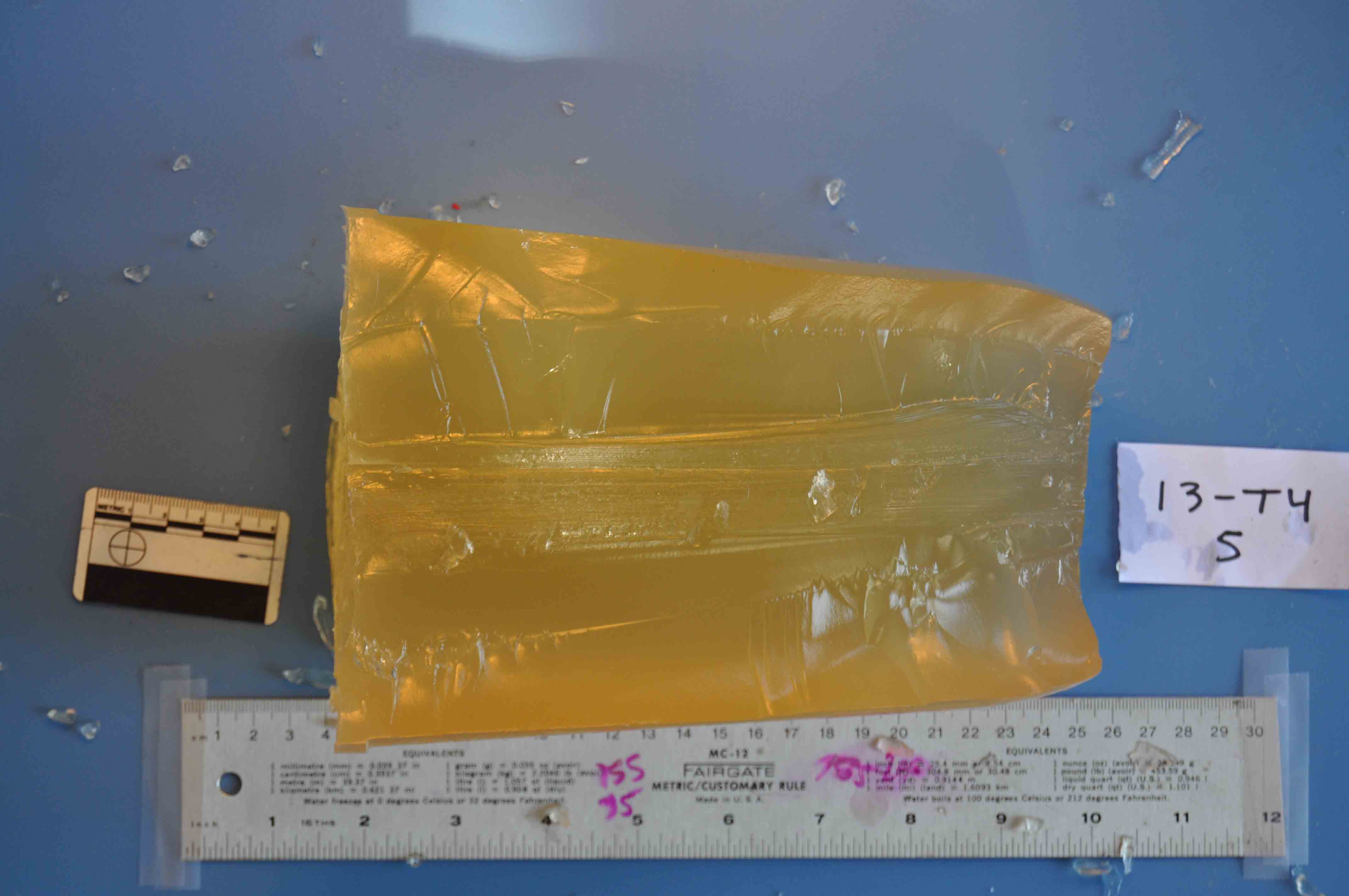

Supplement: File S2 — Wound track images, shapefiles, and tps files. (ZIP) [file pone.0104514.s002.zip › File S2/JPEGS/T4-5b.jpg]

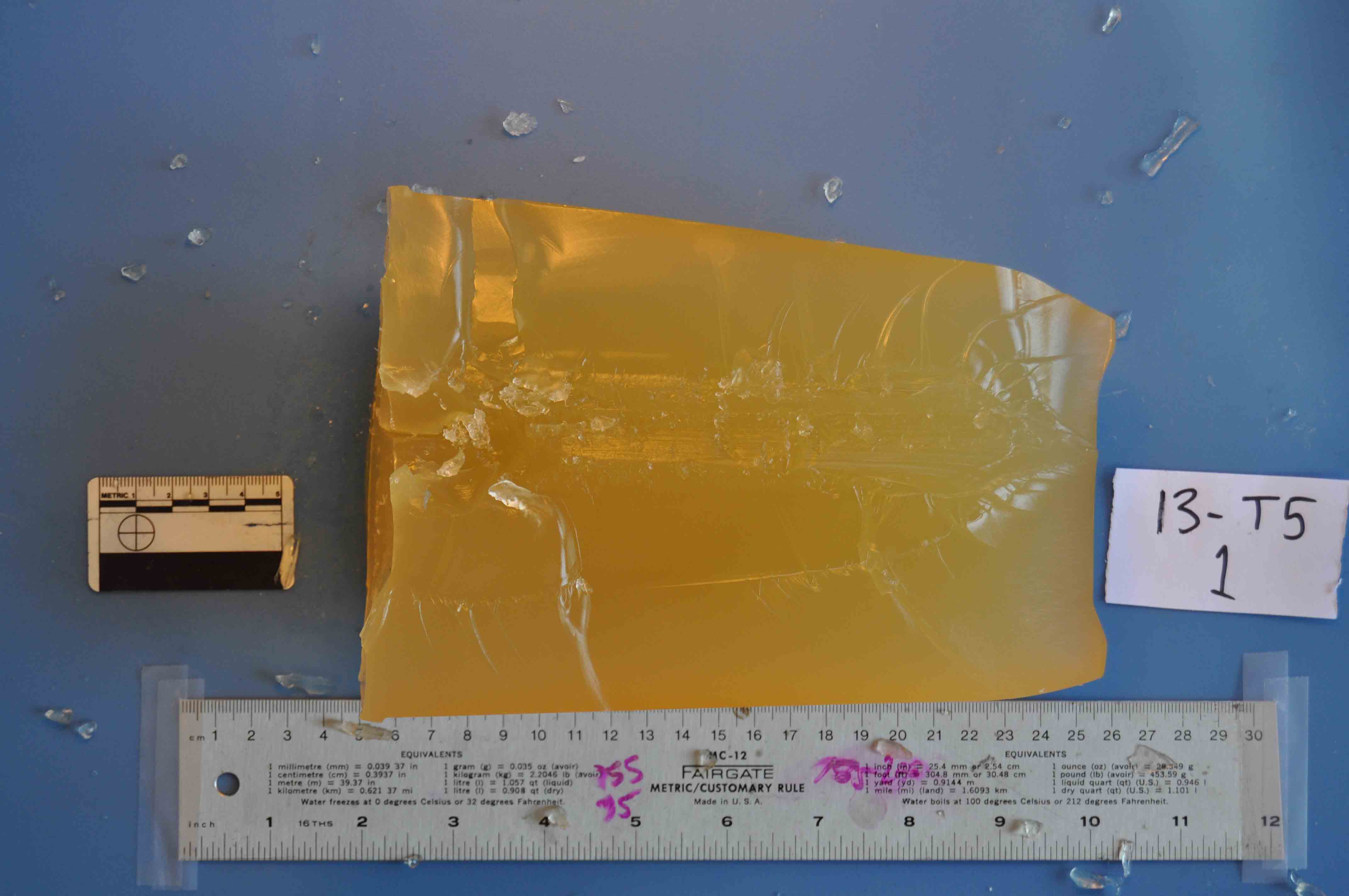

Supplement: File S2 — Wound track images, shapefiles, and tps files. (ZIP) [file pone.0104514.s002.zip › File S2/JPEGS/T5-1a.jpg]

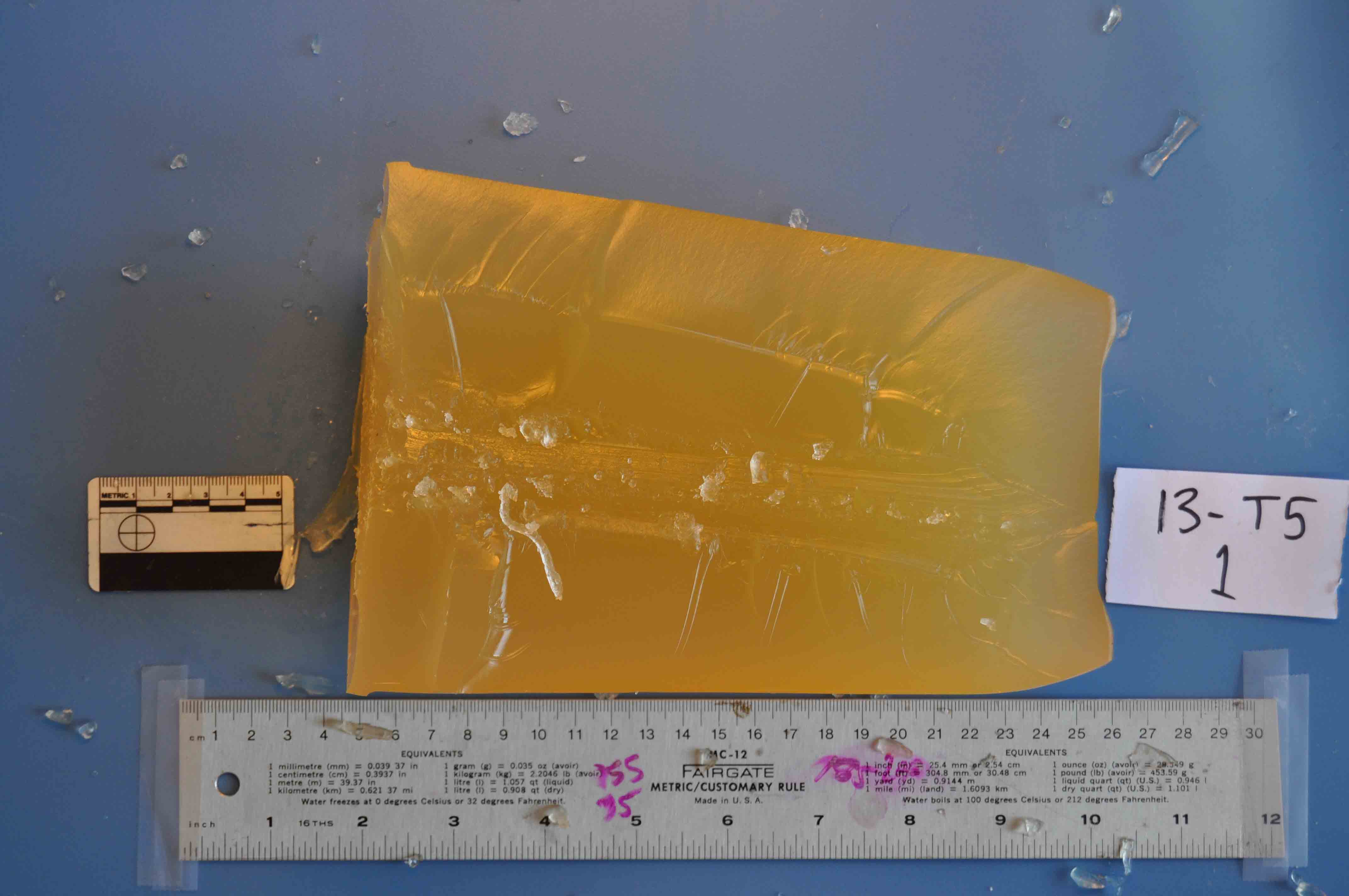

Supplement: File S2 — Wound track images, shapefiles, and tps files. (ZIP) [file pone.0104514.s002.zip › File S2/JPEGS/T5-1b.jpg]

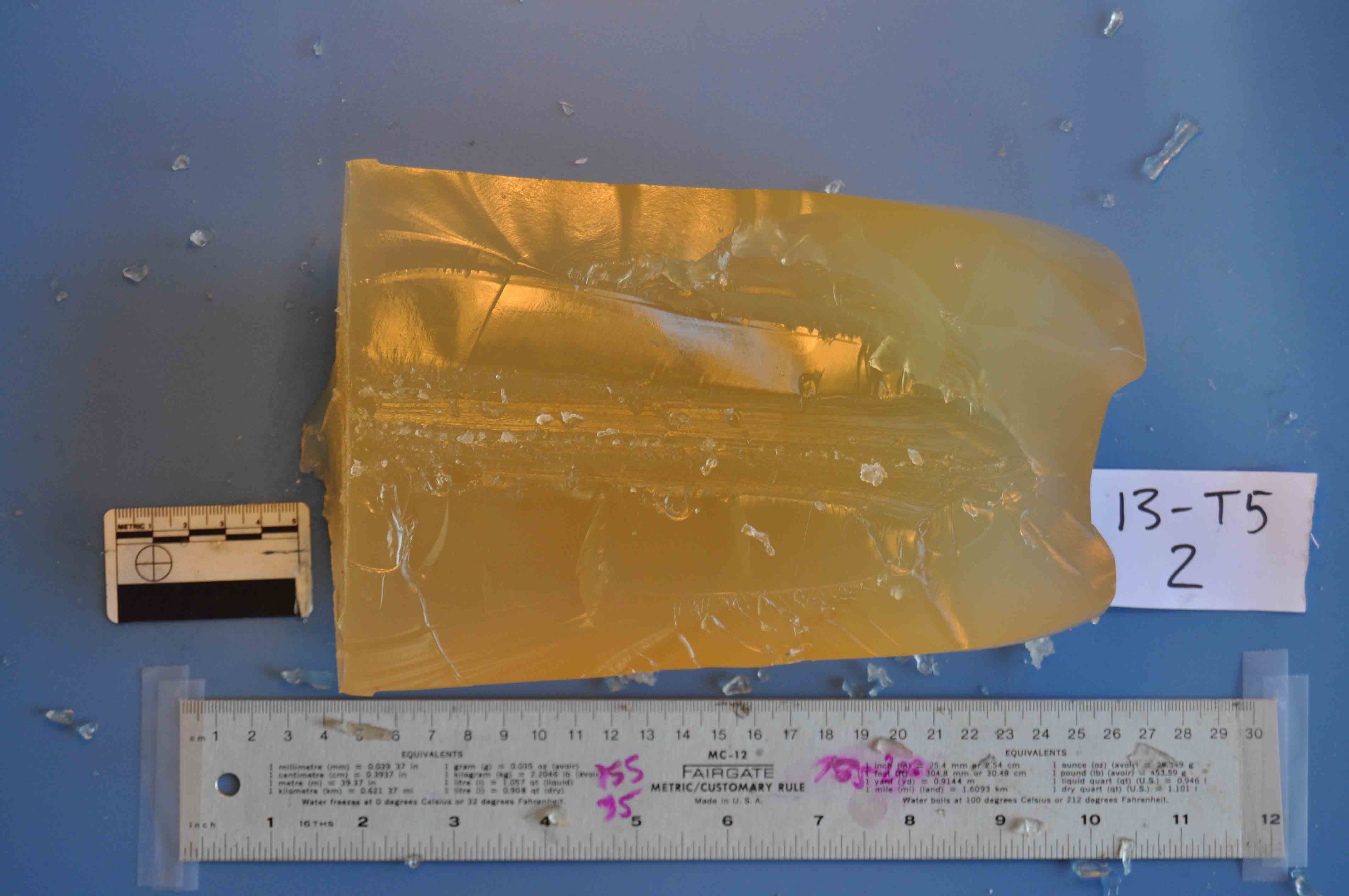

Supplement: File S2 — Wound track images, shapefiles, and tps files. (ZIP) [file pone.0104514.s002.zip › File S2/JPEGS/T5-2a.jpg]

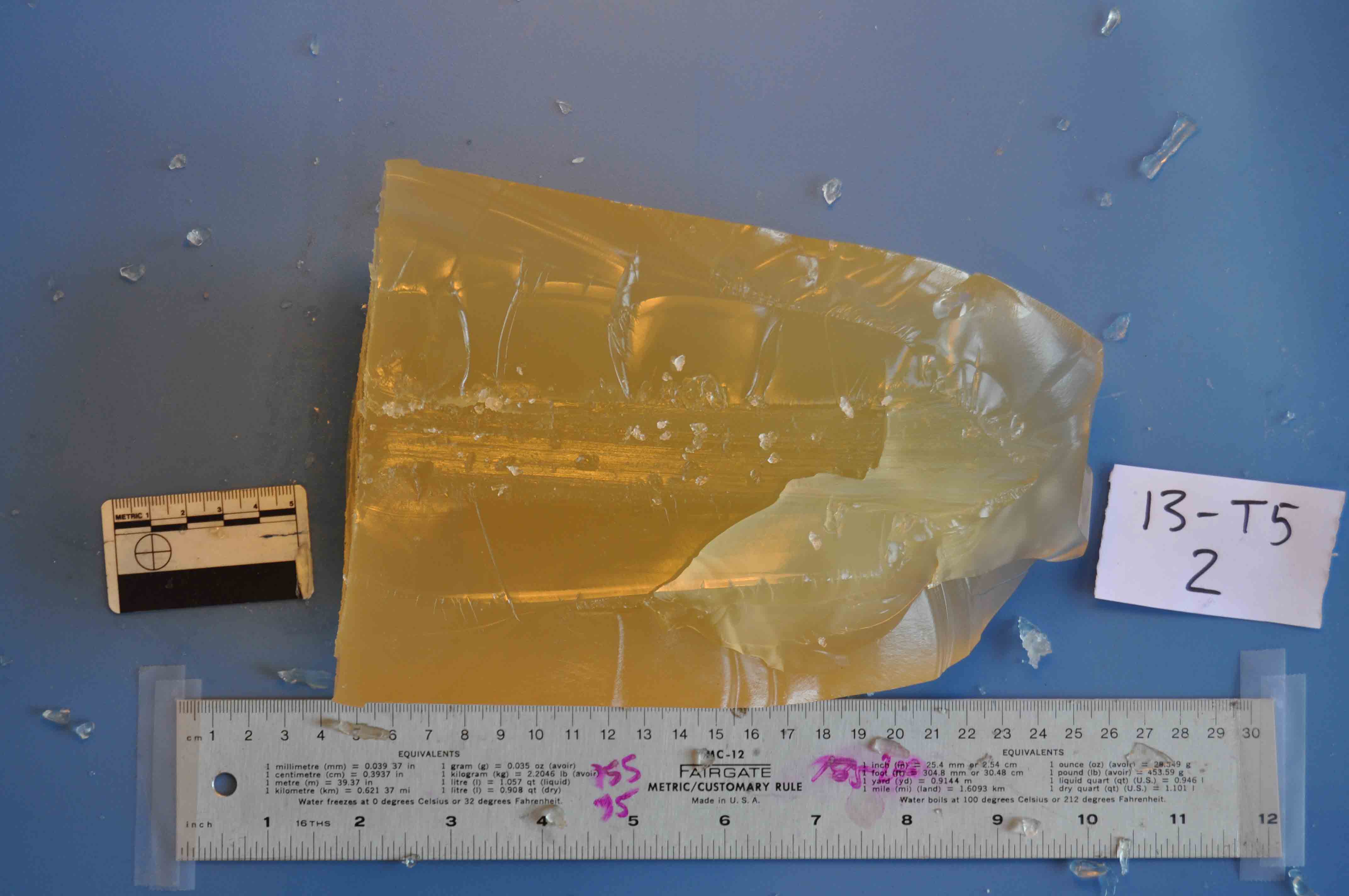

Supplement: File S2 — Wound track images, shapefiles, and tps files. (ZIP) [file pone.0104514.s002.zip › File S2/JPEGS/T5-2b.jpg]

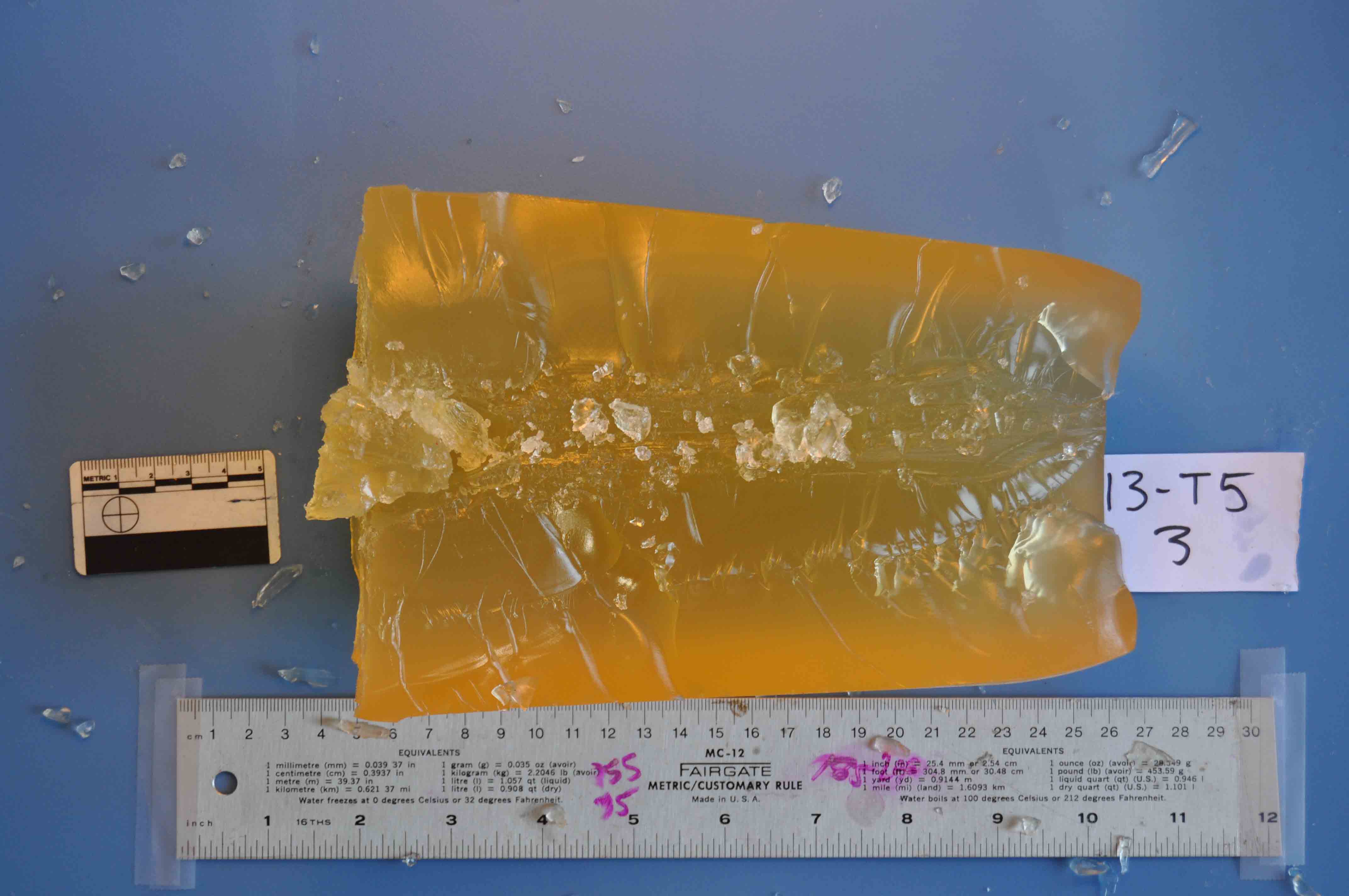

Supplement: File S2 — Wound track images, shapefiles, and tps files. (ZIP) [file pone.0104514.s002.zip › File S2/JPEGS/T5-3a.jpg]

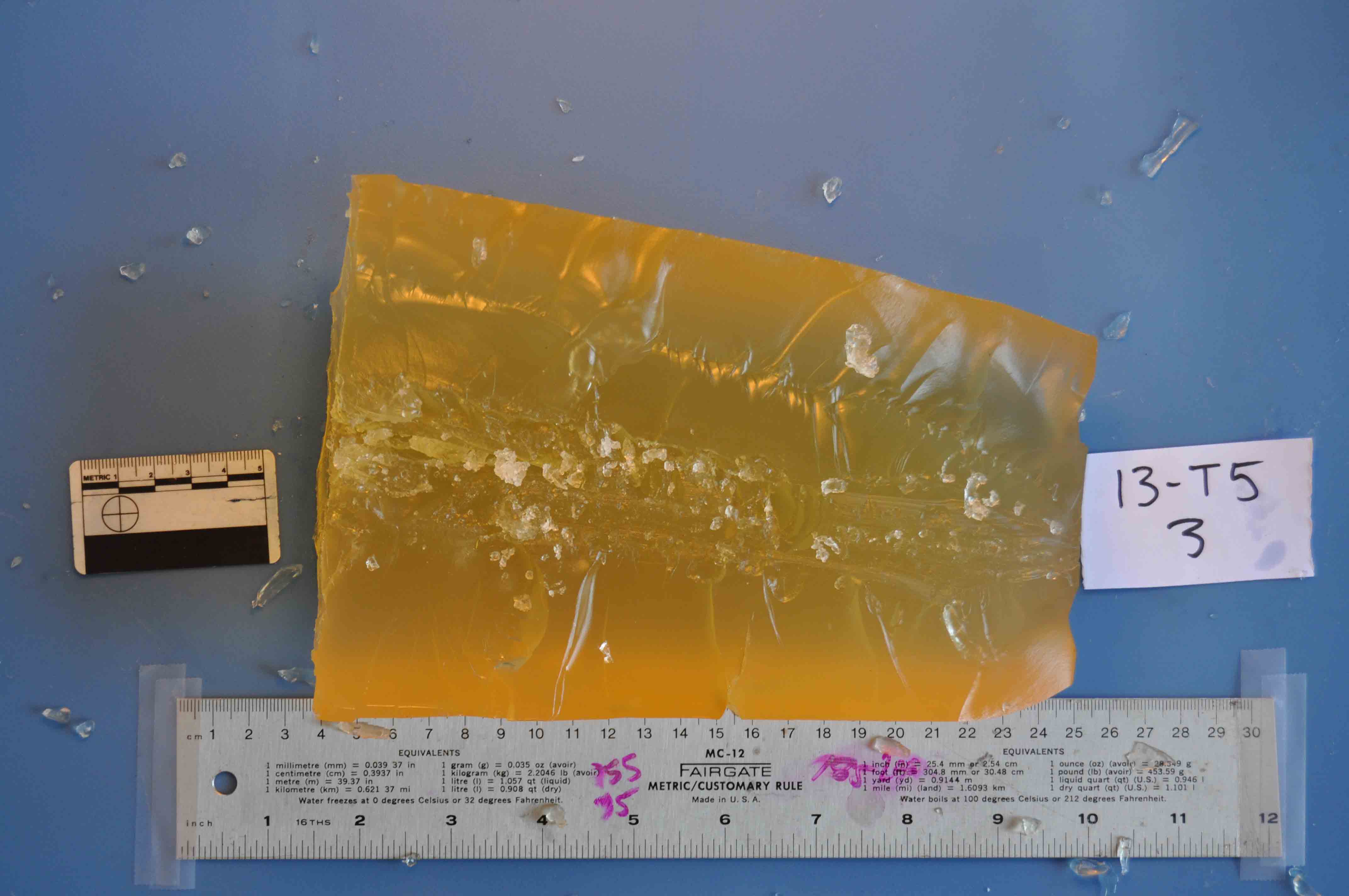

Supplement: File S2 — Wound track images, shapefiles, and tps files. (ZIP) [file pone.0104514.s002.zip › File S2/JPEGS/T5-3b.jpg]

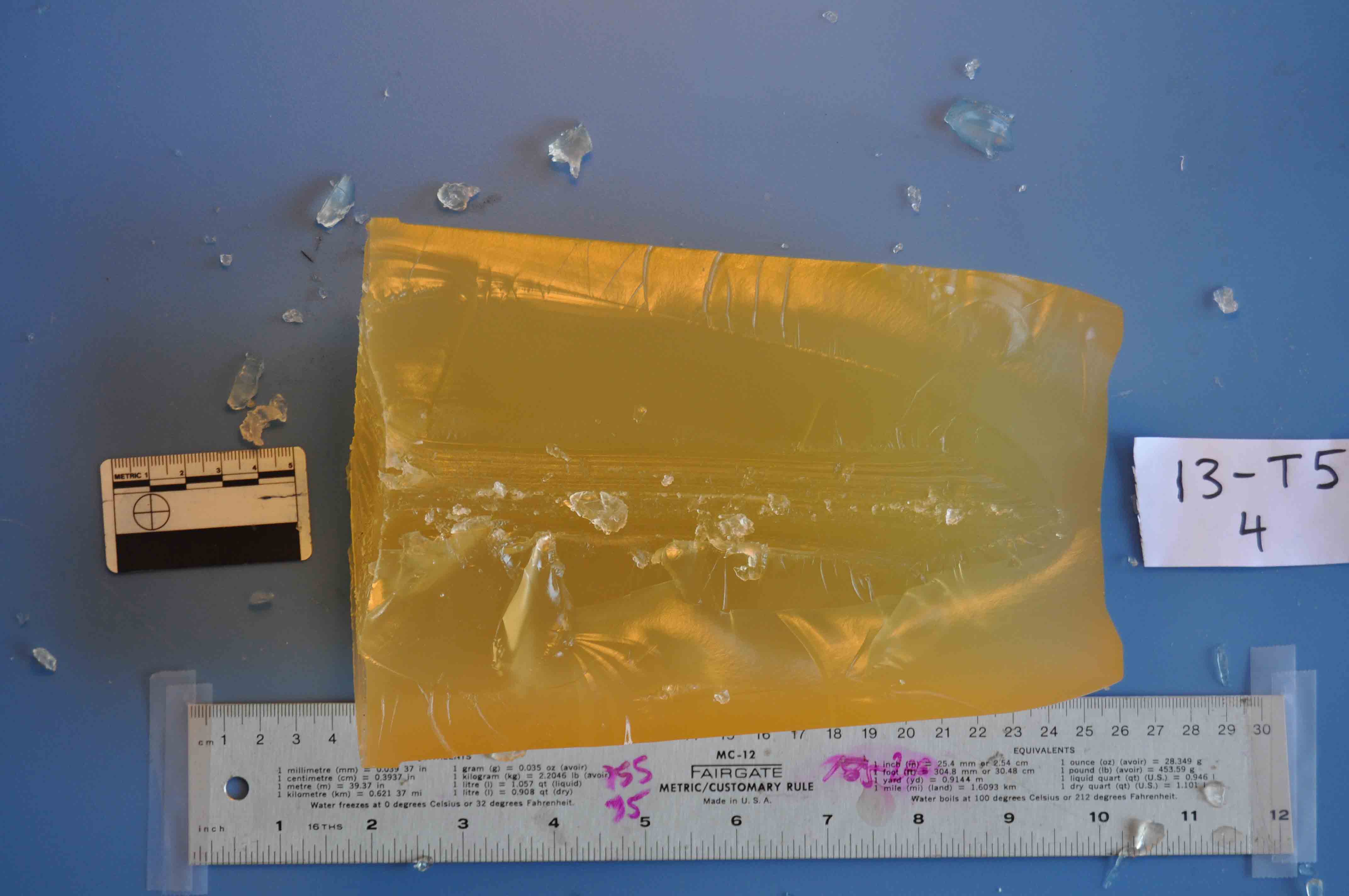

Supplement: File S2 — Wound track images, shapefiles, and tps files. (ZIP) [file pone.0104514.s002.zip › File S2/JPEGS/T5-4a.jpg]

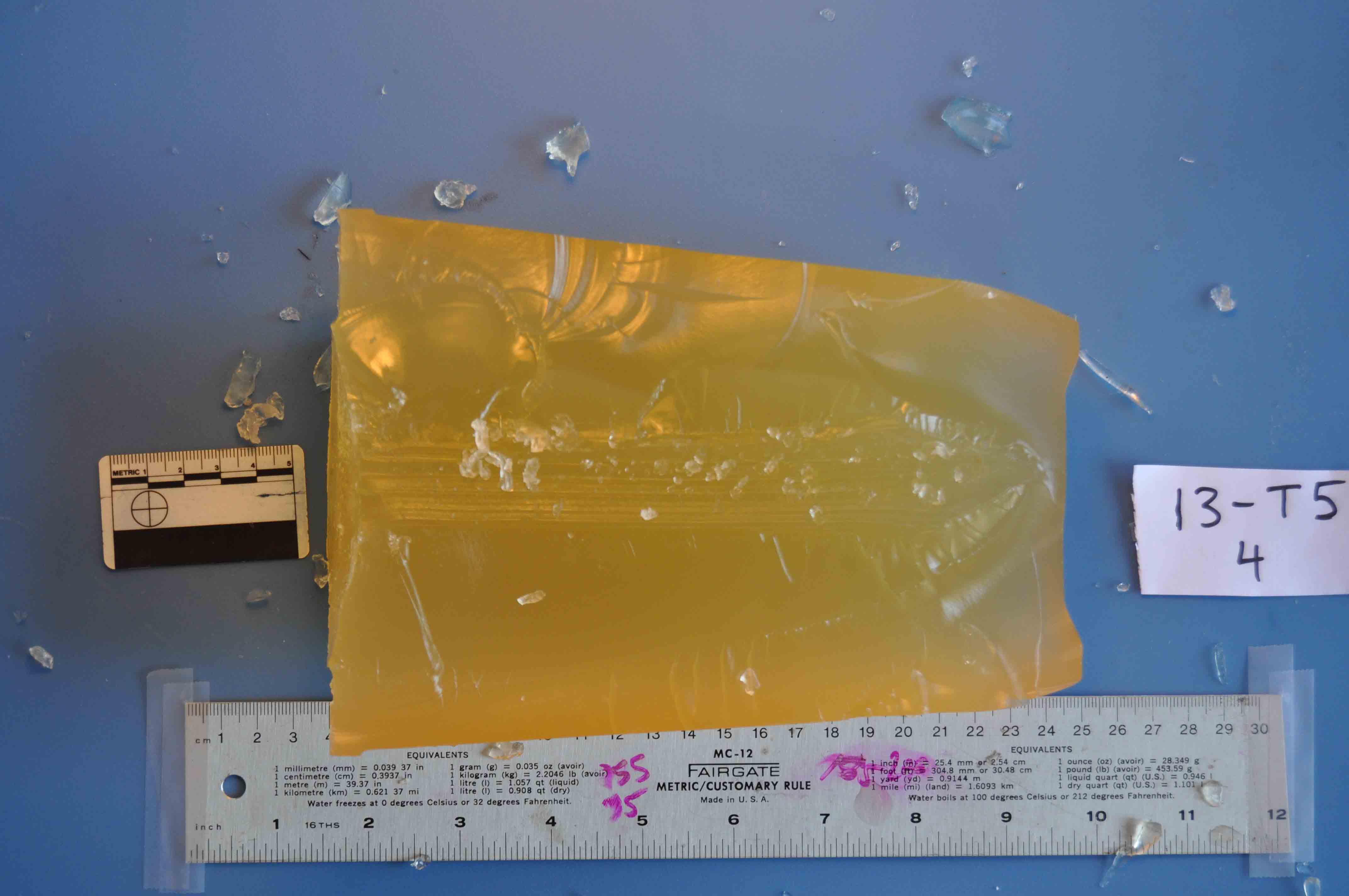

Supplement: File S2 — Wound track images, shapefiles, and tps files. (ZIP) [file pone.0104514.s002.zip › File S2/JPEGS/T5-4b.jpg]

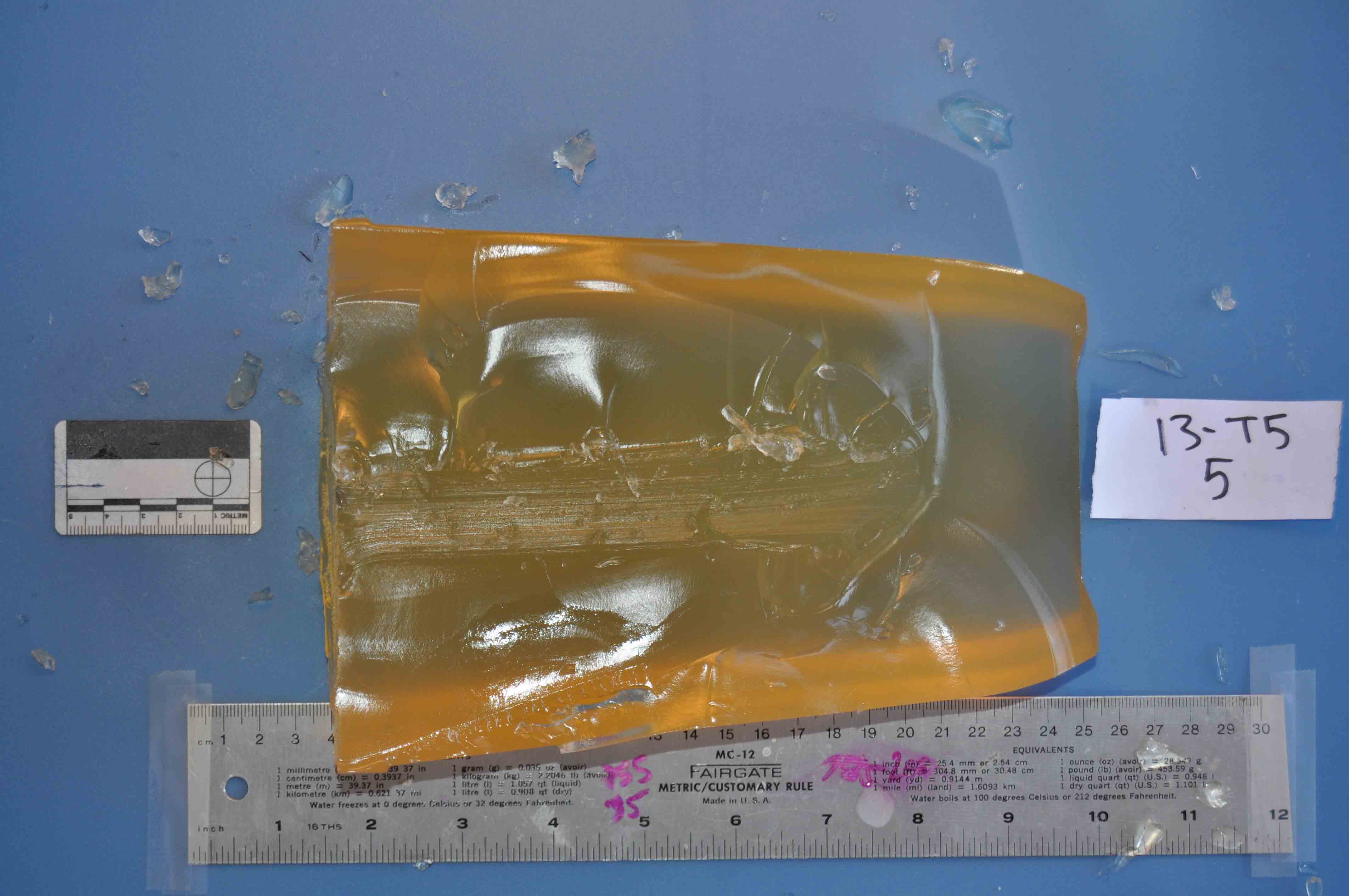

Supplement: File S2 — Wound track images, shapefiles, and tps files. (ZIP) [file pone.0104514.s002.zip › File S2/JPEGS/T5-5a.jpg]

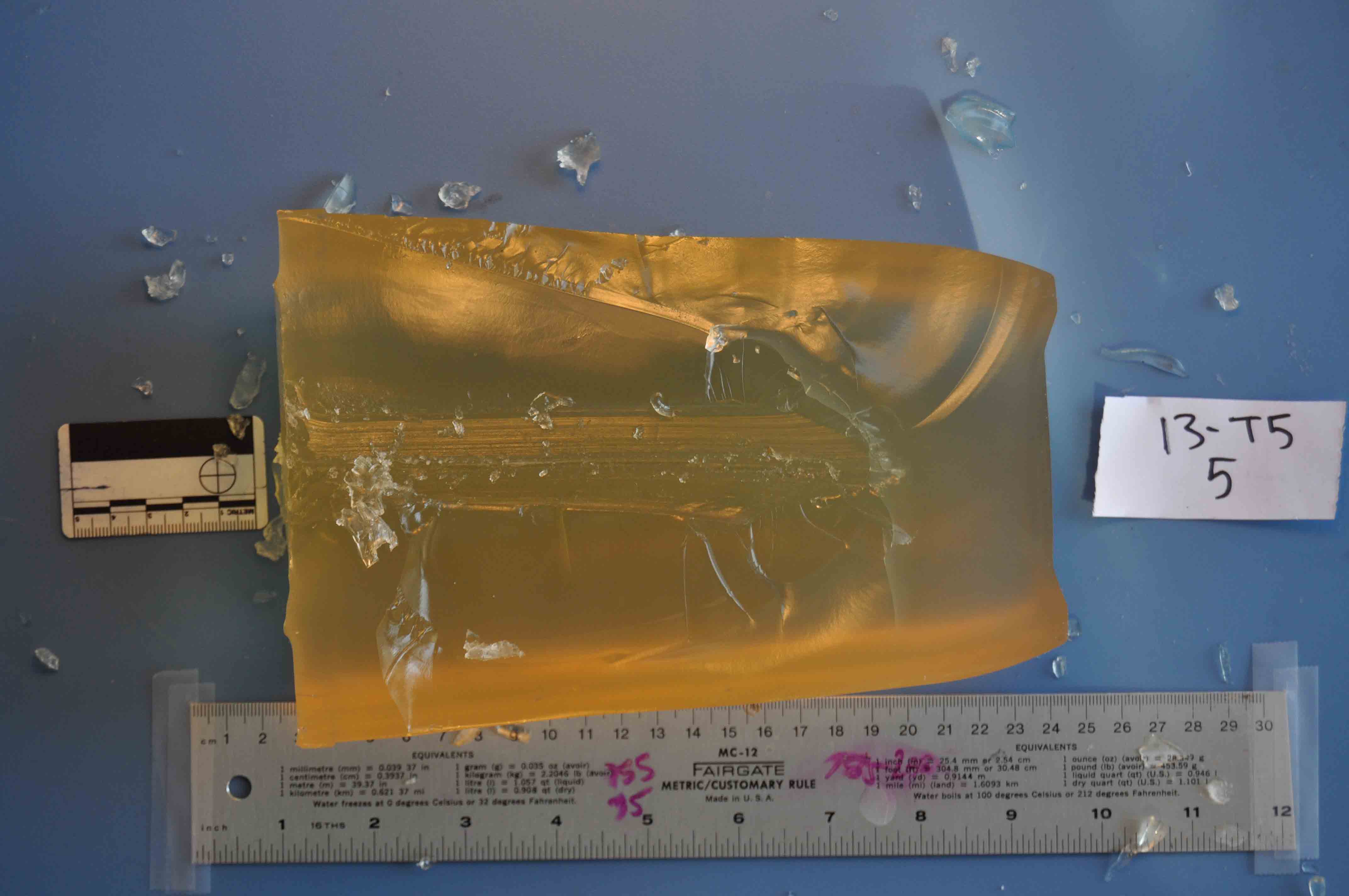

Supplement: File S2 — Wound track images, shapefiles, and tps files. (ZIP) [file pone.0104514.s002.zip › File S2/JPEGS/T5-5b.jpg]

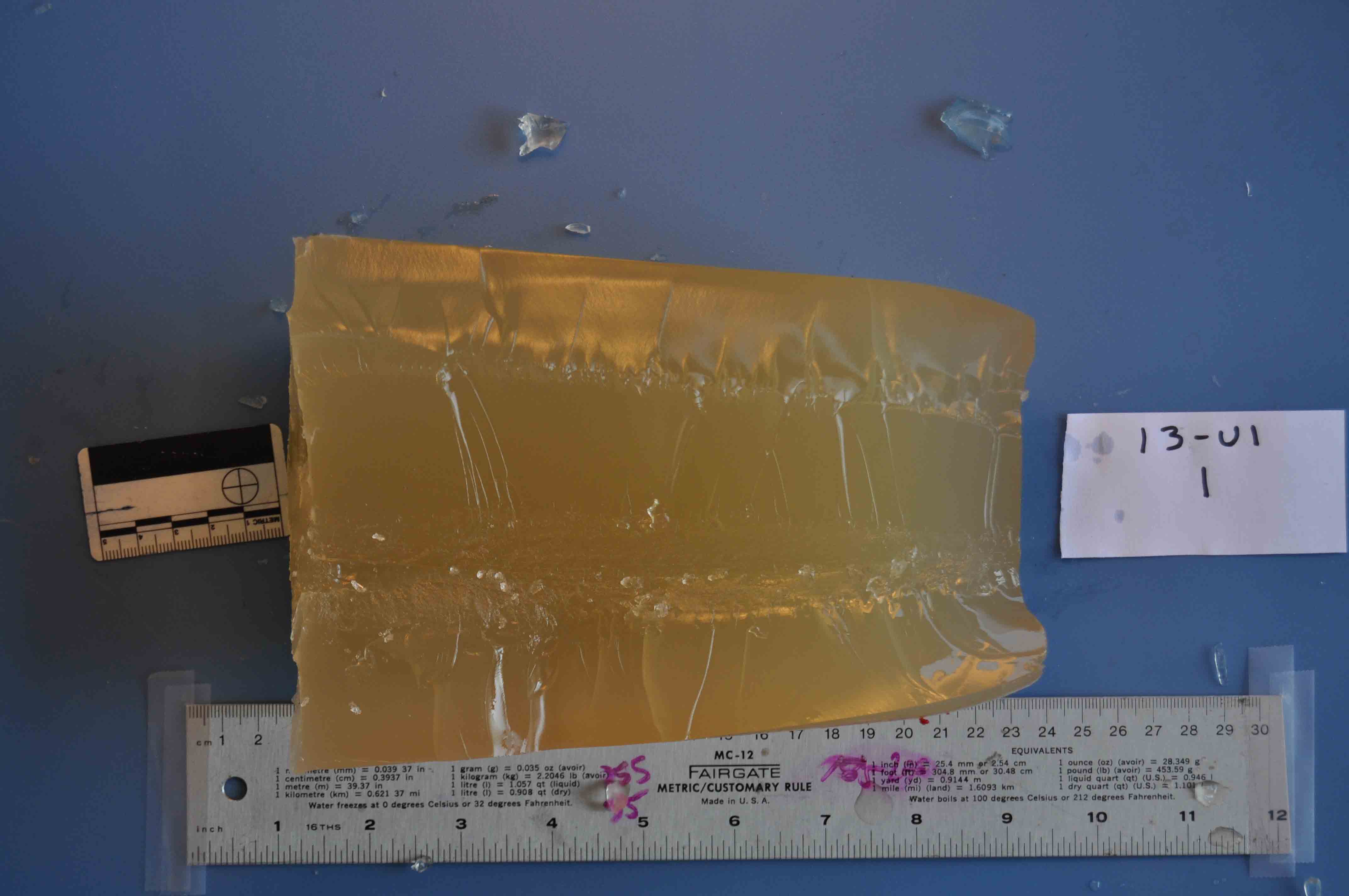

Supplement: File S2 — Wound track images, shapefiles, and tps files. (ZIP) [file pone.0104514.s002.zip › File S2/JPEGS/U1-1a.jpg]

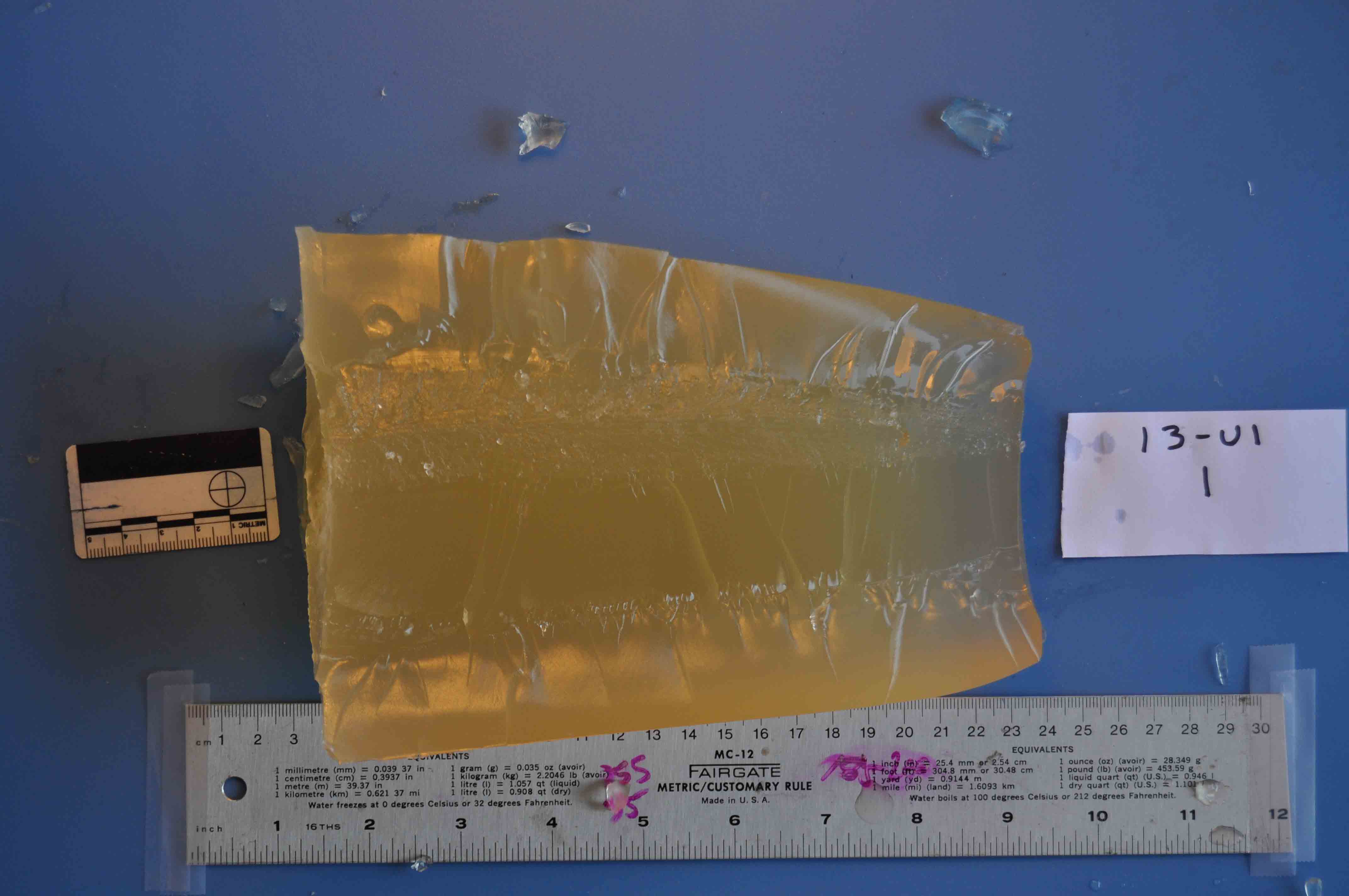

Supplement: File S2 — Wound track images, shapefiles, and tps files. (ZIP) [file pone.0104514.s002.zip › File S2/JPEGS/U1-1b.jpg]

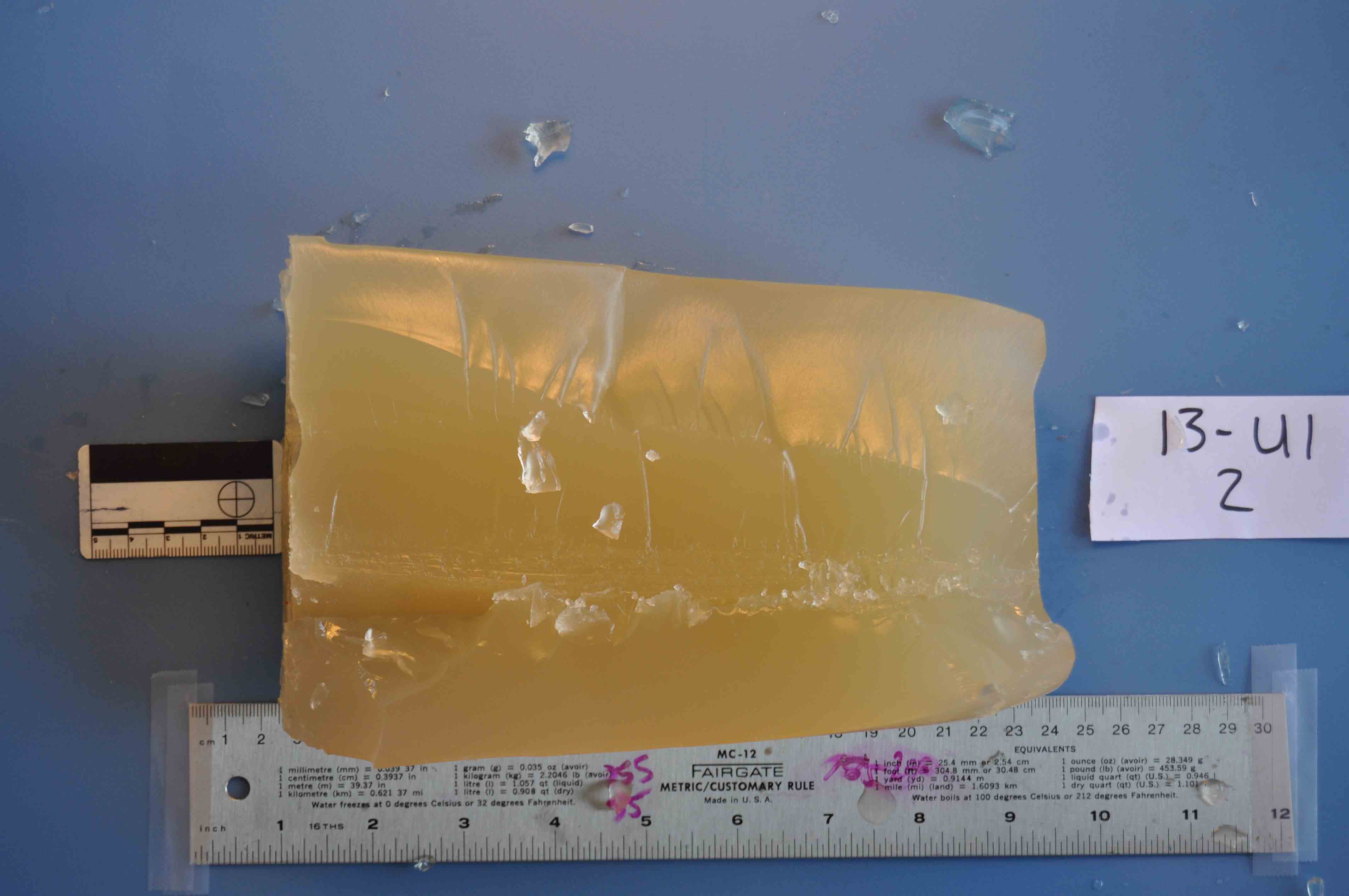

Supplement: File S2 — Wound track images, shapefiles, and tps files. (ZIP) [file pone.0104514.s002.zip › File S2/JPEGS/U1-2a.jpg]

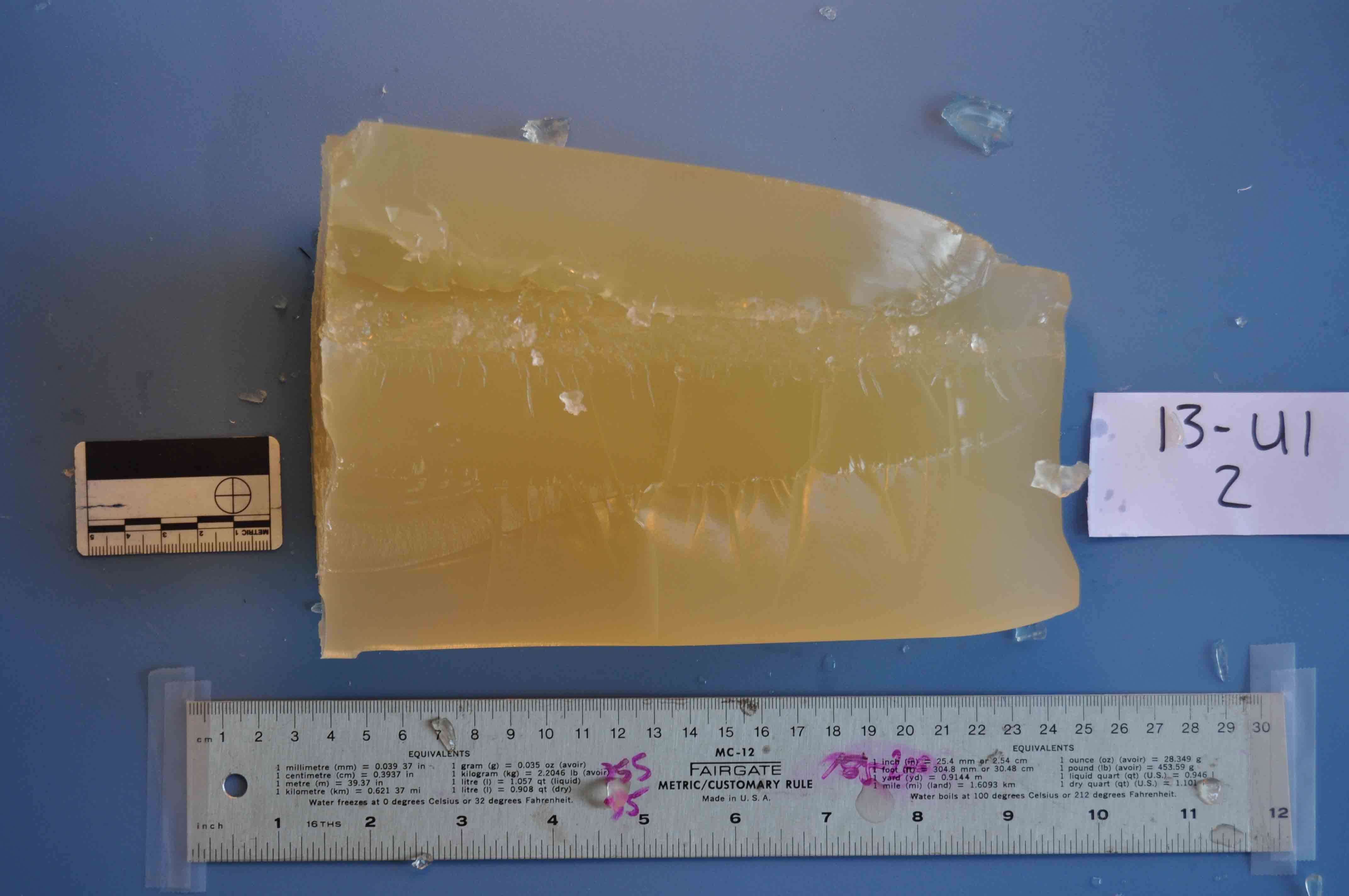

Supplement: File S2 — Wound track images, shapefiles, and tps files. (ZIP) [file pone.0104514.s002.zip › File S2/JPEGS/U1-2b.jpg]

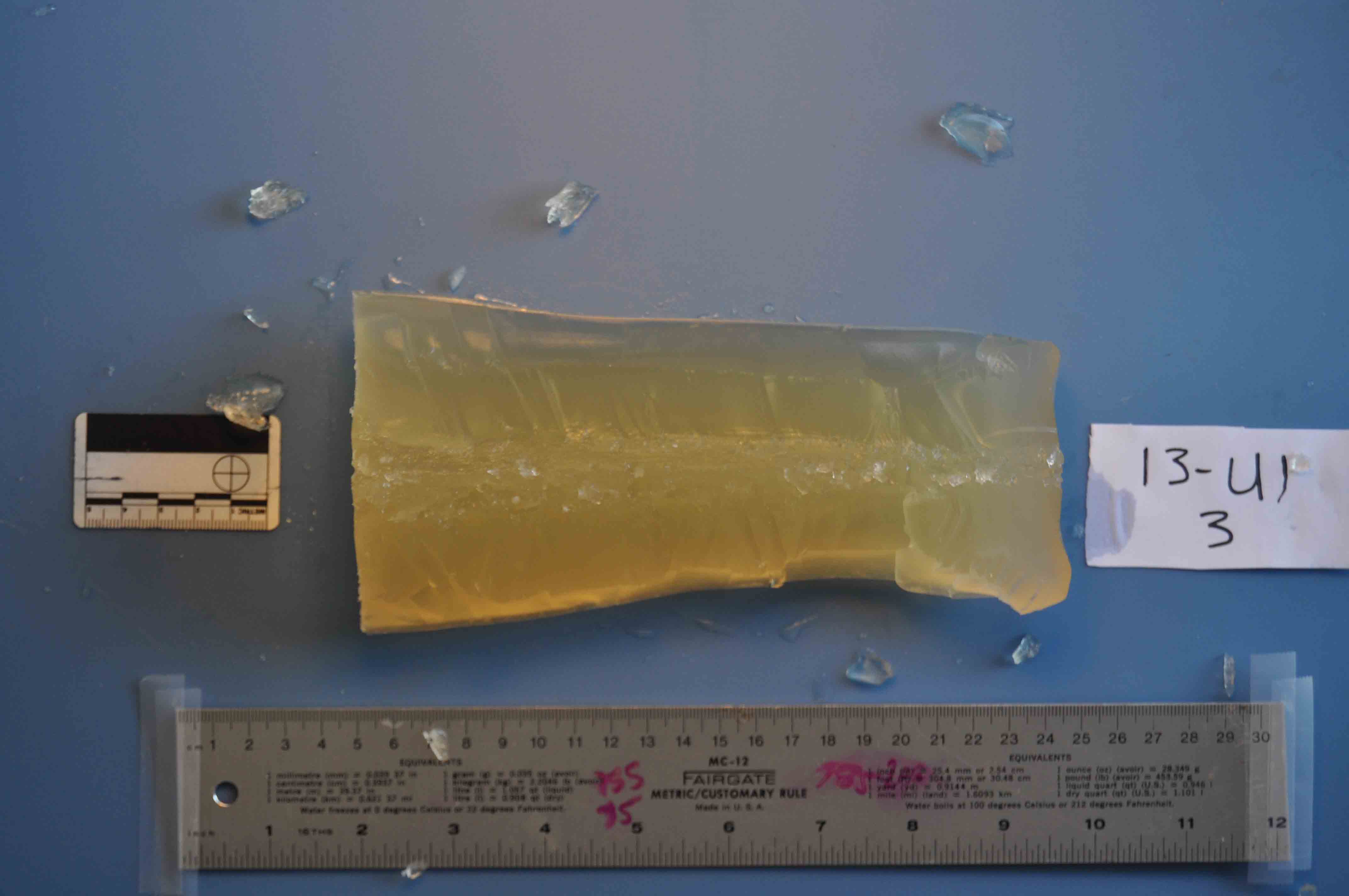

Supplement: File S2 — Wound track images, shapefiles, and tps files. (ZIP) [file pone.0104514.s002.zip › File S2/JPEGS/U1-3a.jpg]

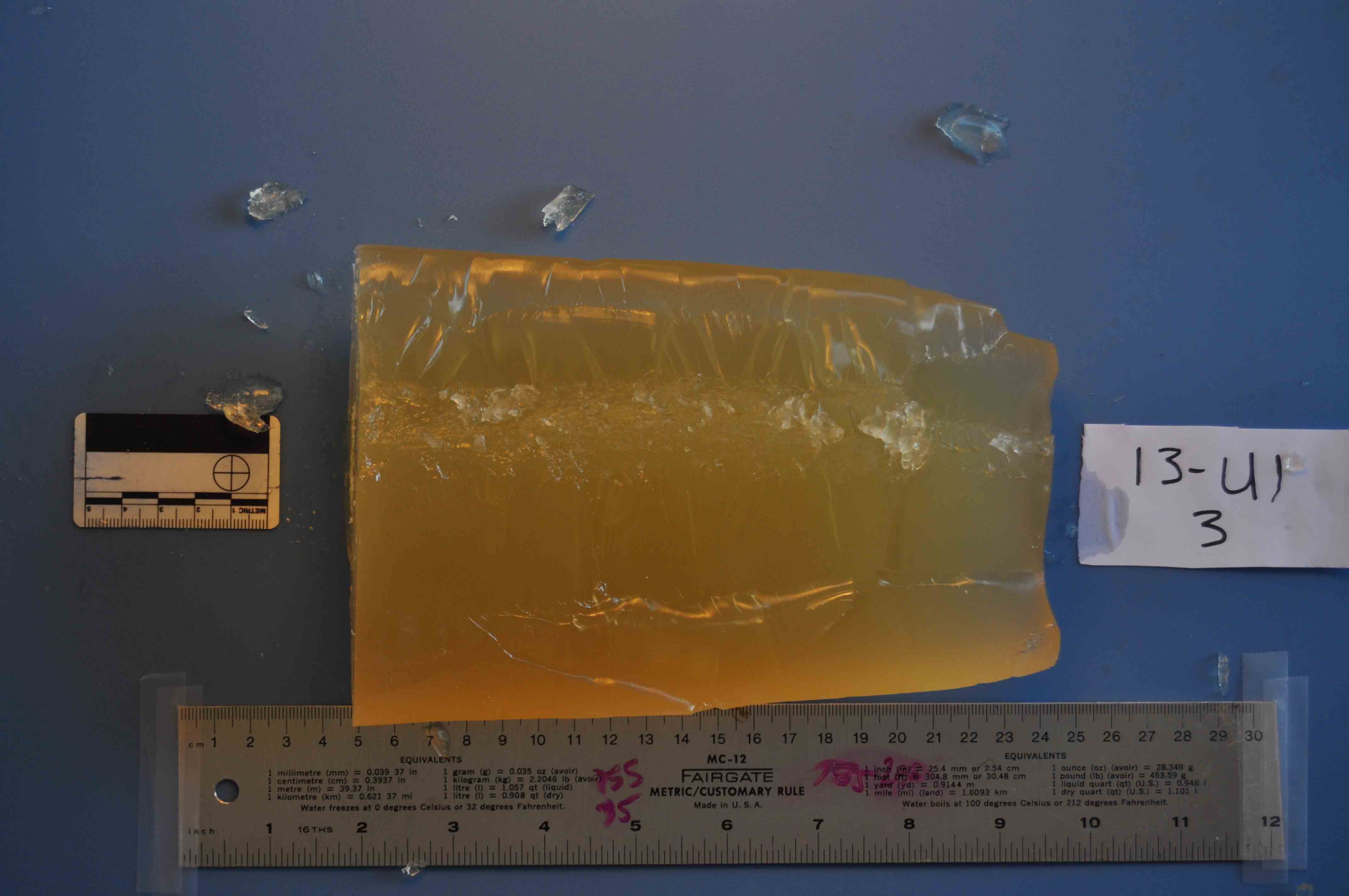

Supplement: File S2 — Wound track images, shapefiles, and tps files. (ZIP) [file pone.0104514.s002.zip › File S2/JPEGS/U1-3b.jpg]

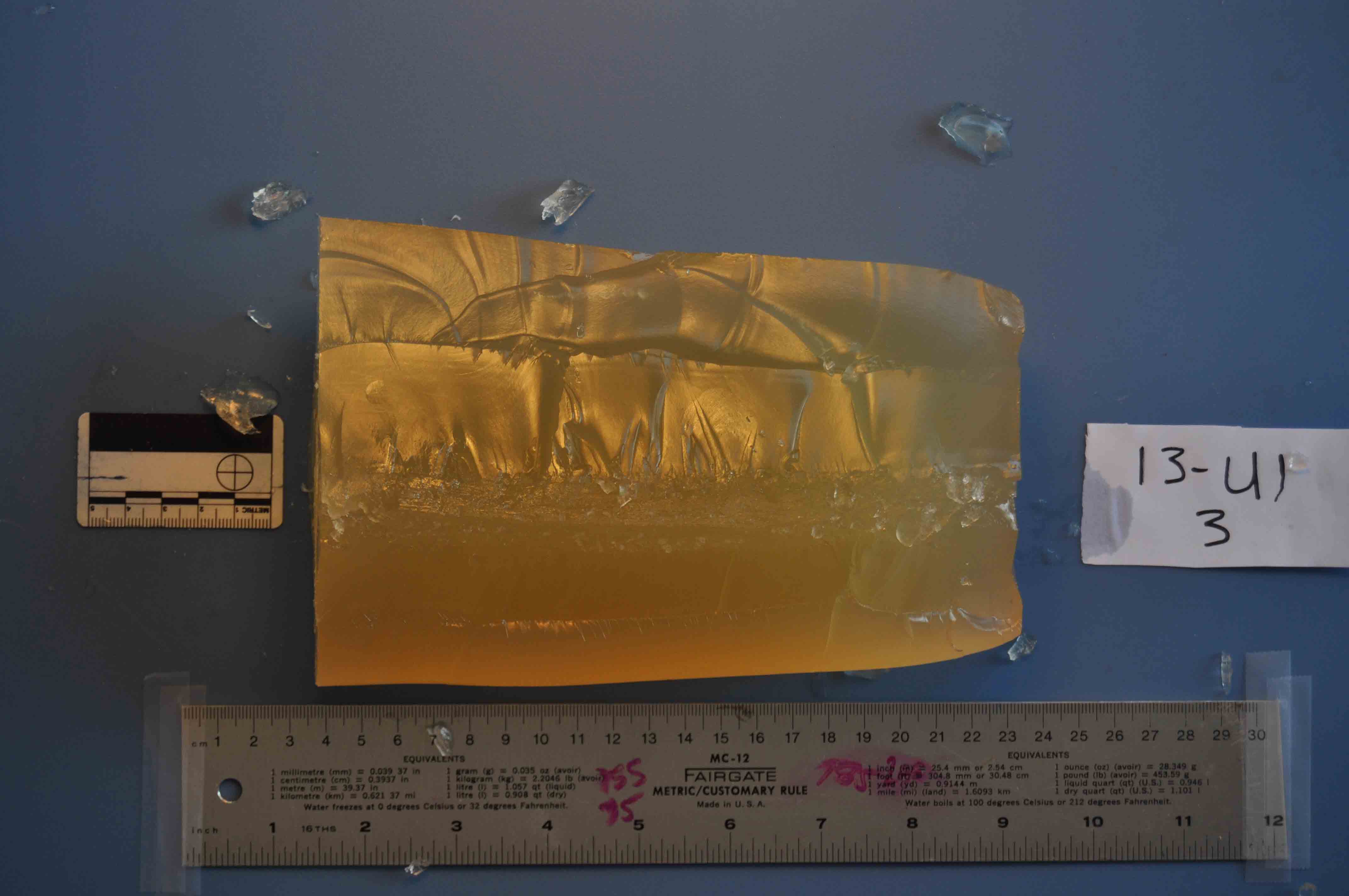

Supplement: File S2 — Wound track images, shapefiles, and tps files. (ZIP) [file pone.0104514.s002.zip › File S2/JPEGS/U1-3c.jpg]

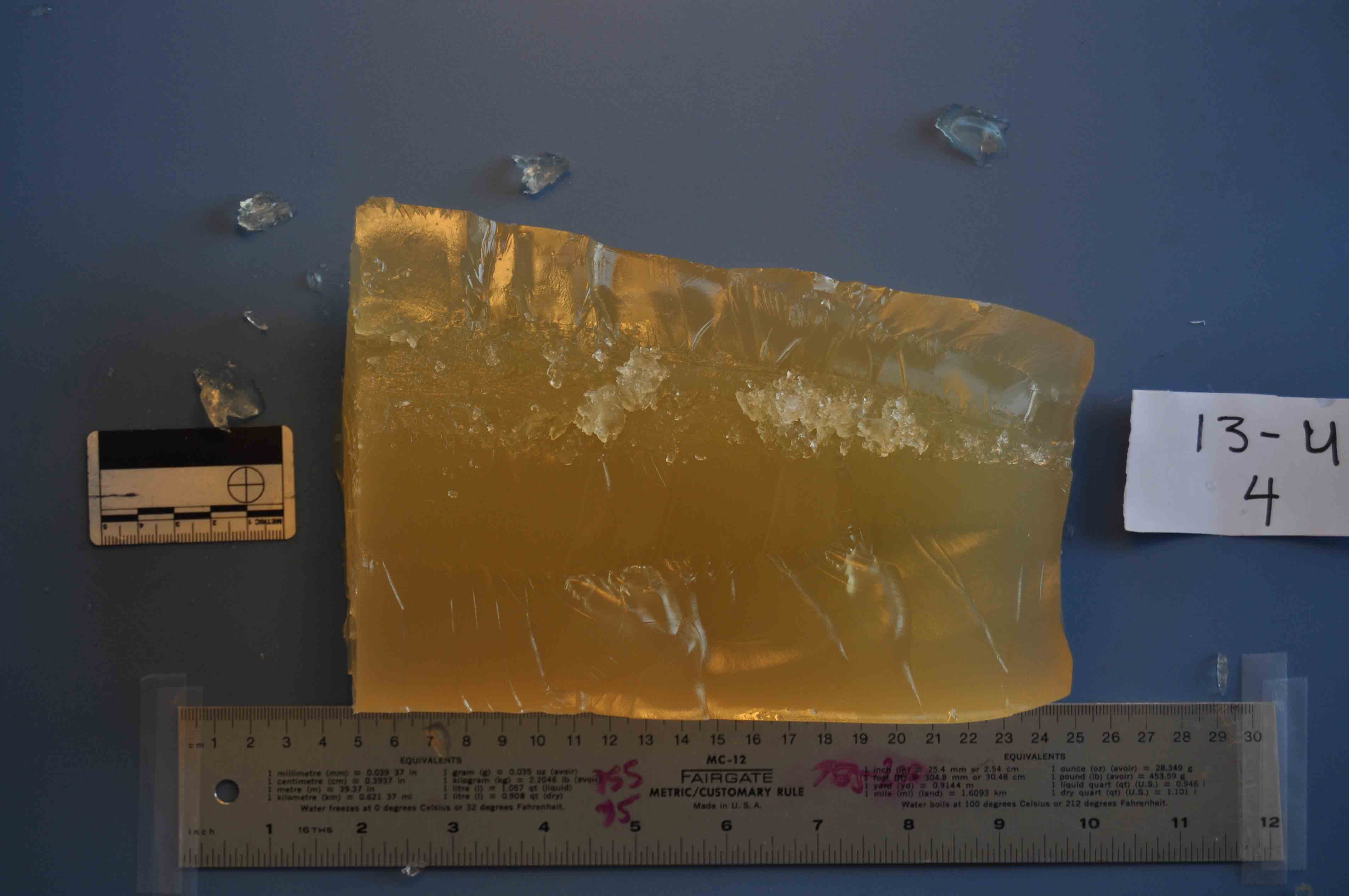

Supplement: File S2 — Wound track images, shapefiles, and tps files. (ZIP) [file pone.0104514.s002.zip › File S2/JPEGS/U1-4a.jpg]

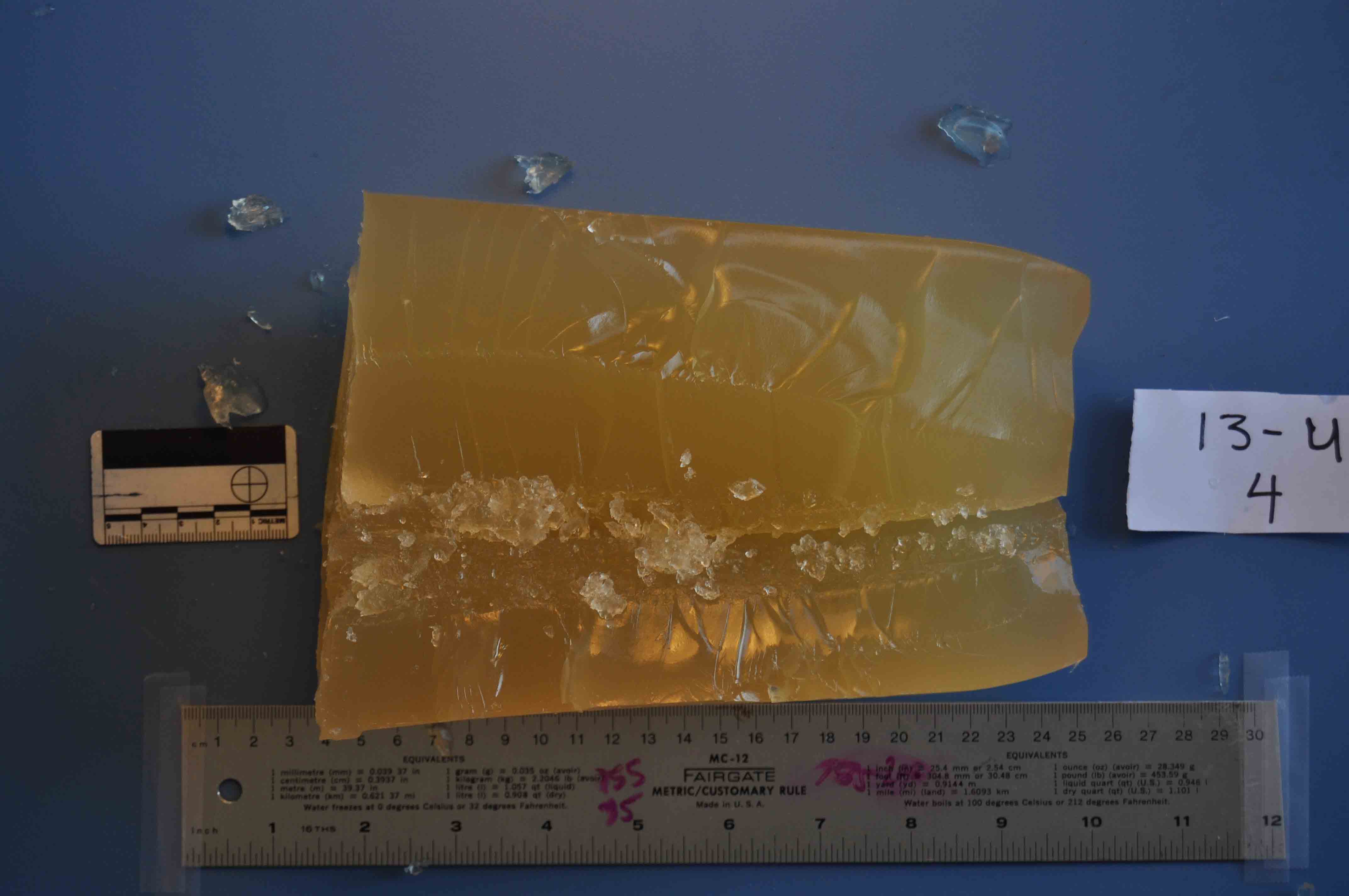

Supplement: File S2 — Wound track images, shapefiles, and tps files. (ZIP) [file pone.0104514.s002.zip › File S2/JPEGS/U1-4b.jpg]

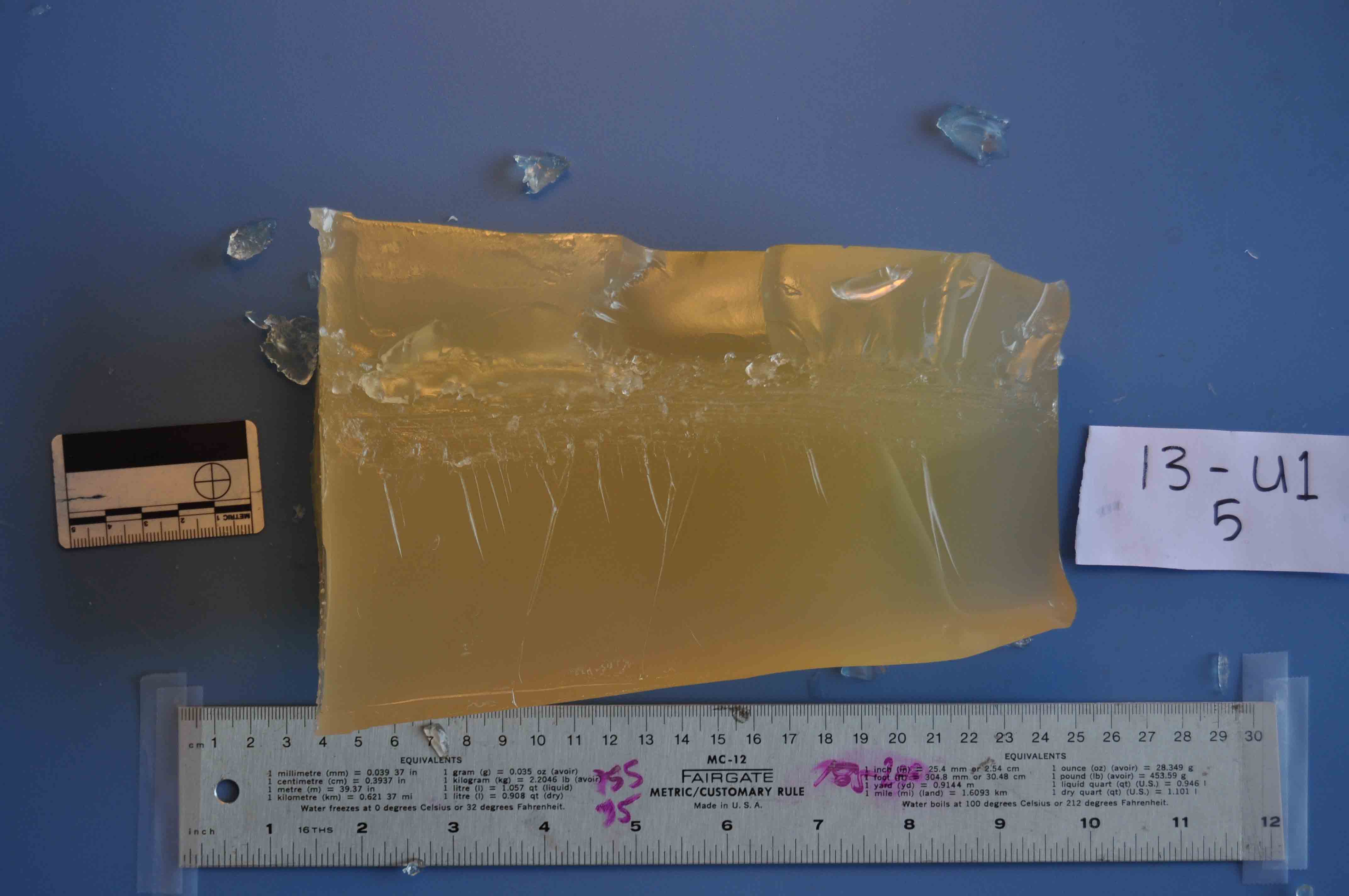

Supplement: File S2 — Wound track images, shapefiles, and tps files. (ZIP) [file pone.0104514.s002.zip › File S2/JPEGS/U1-5a.jpg]

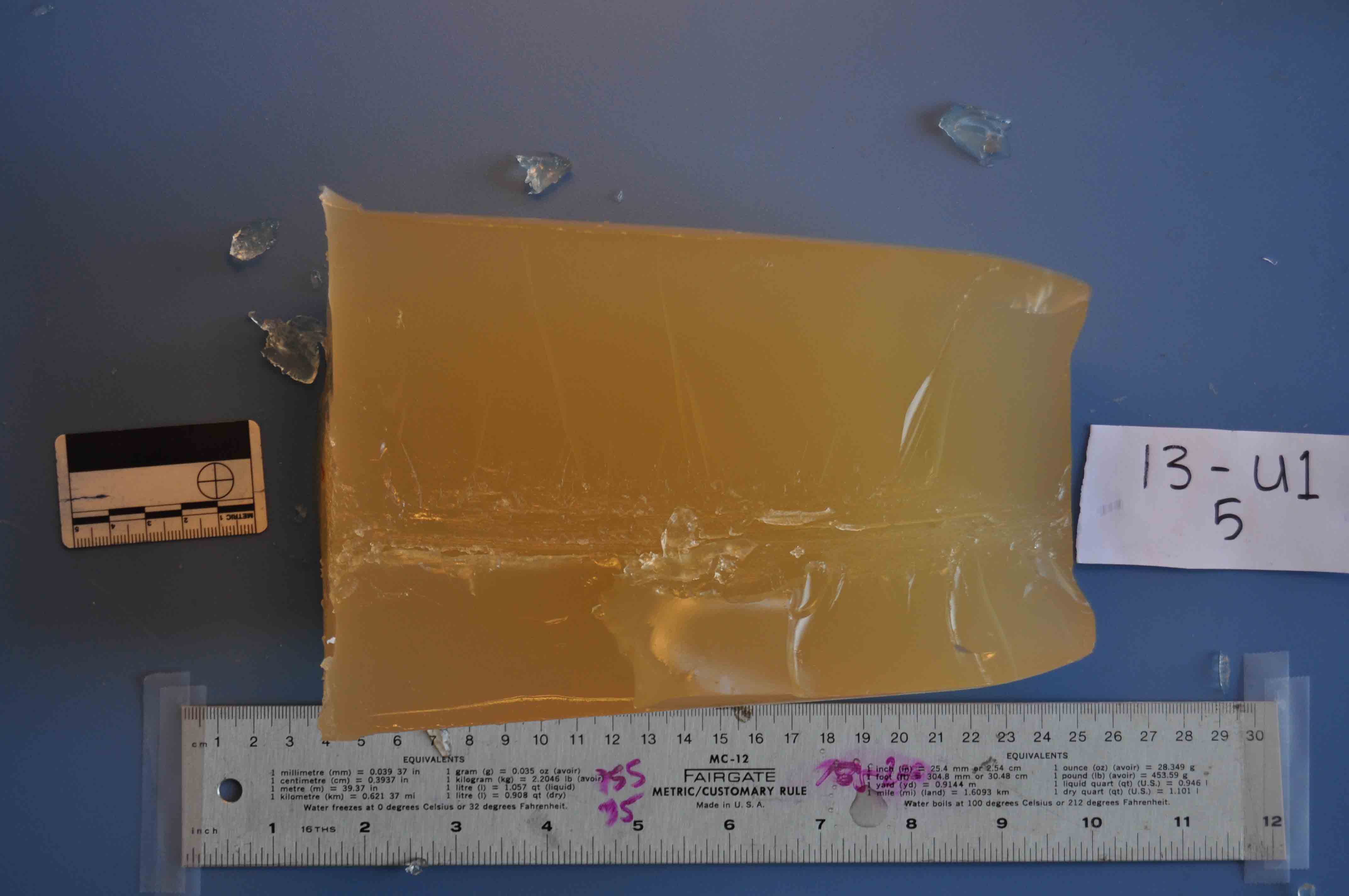

Supplement: File S2 — Wound track images, shapefiles, and tps files. (ZIP) [file pone.0104514.s002.zip › File S2/JPEGS/U1-5b.jpg]

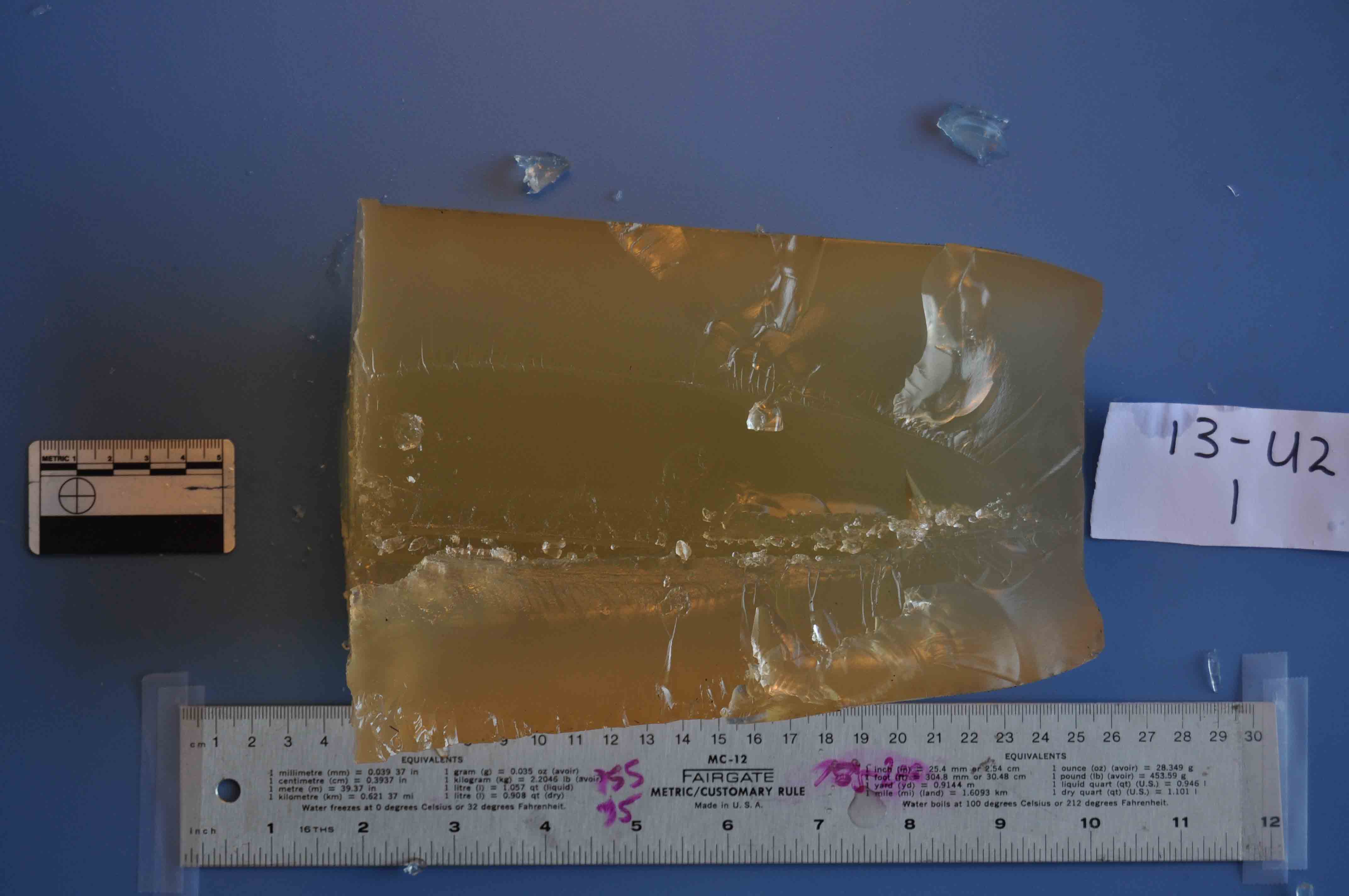

Supplement: File S2 — Wound track images, shapefiles, and tps files. (ZIP) [file pone.0104514.s002.zip › File S2/JPEGS/U2-1a.jpg]

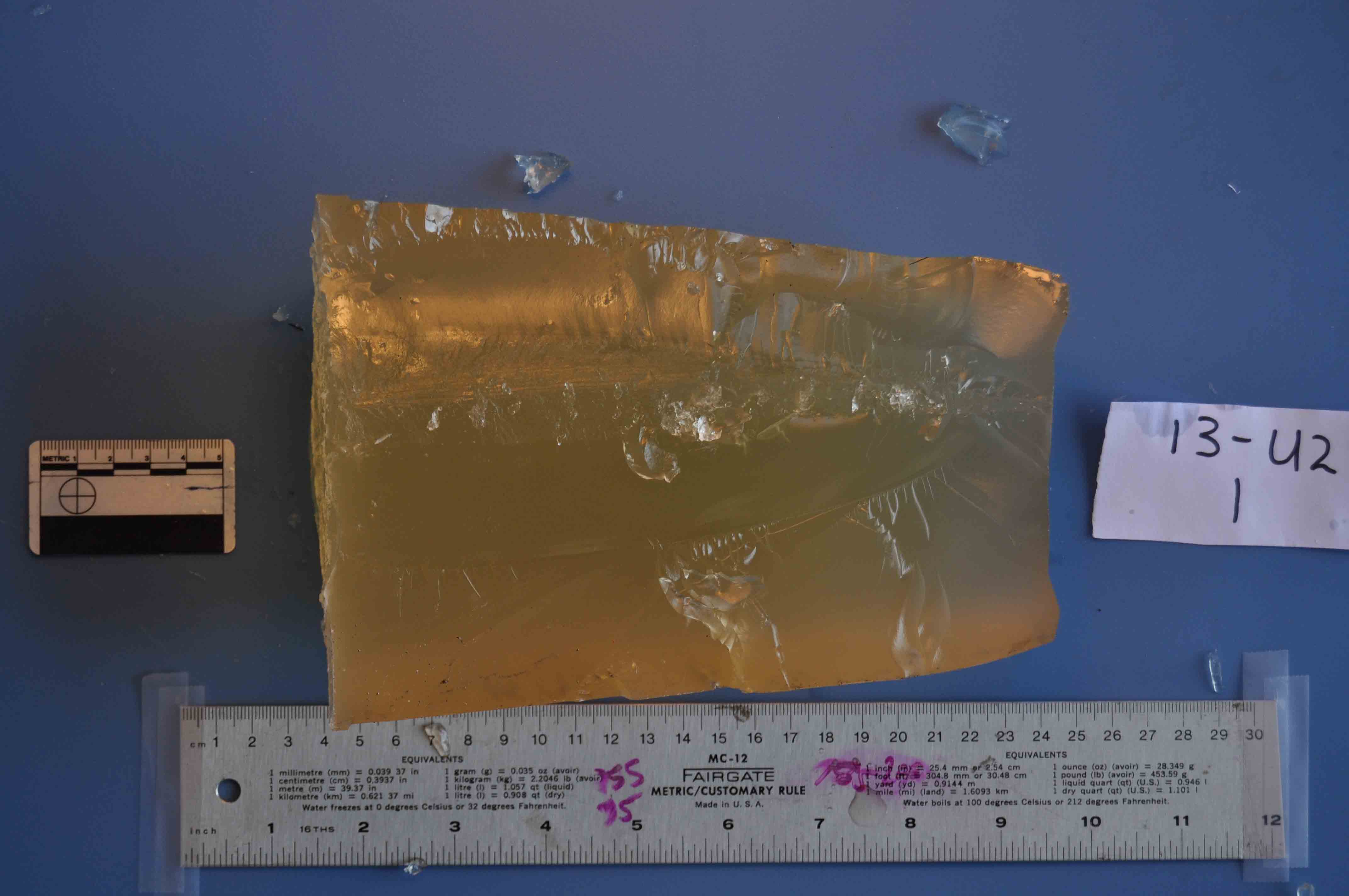

Supplement: File S2 — Wound track images, shapefiles, and tps files. (ZIP) [file pone.0104514.s002.zip › File S2/JPEGS/U2-1b.jpg]

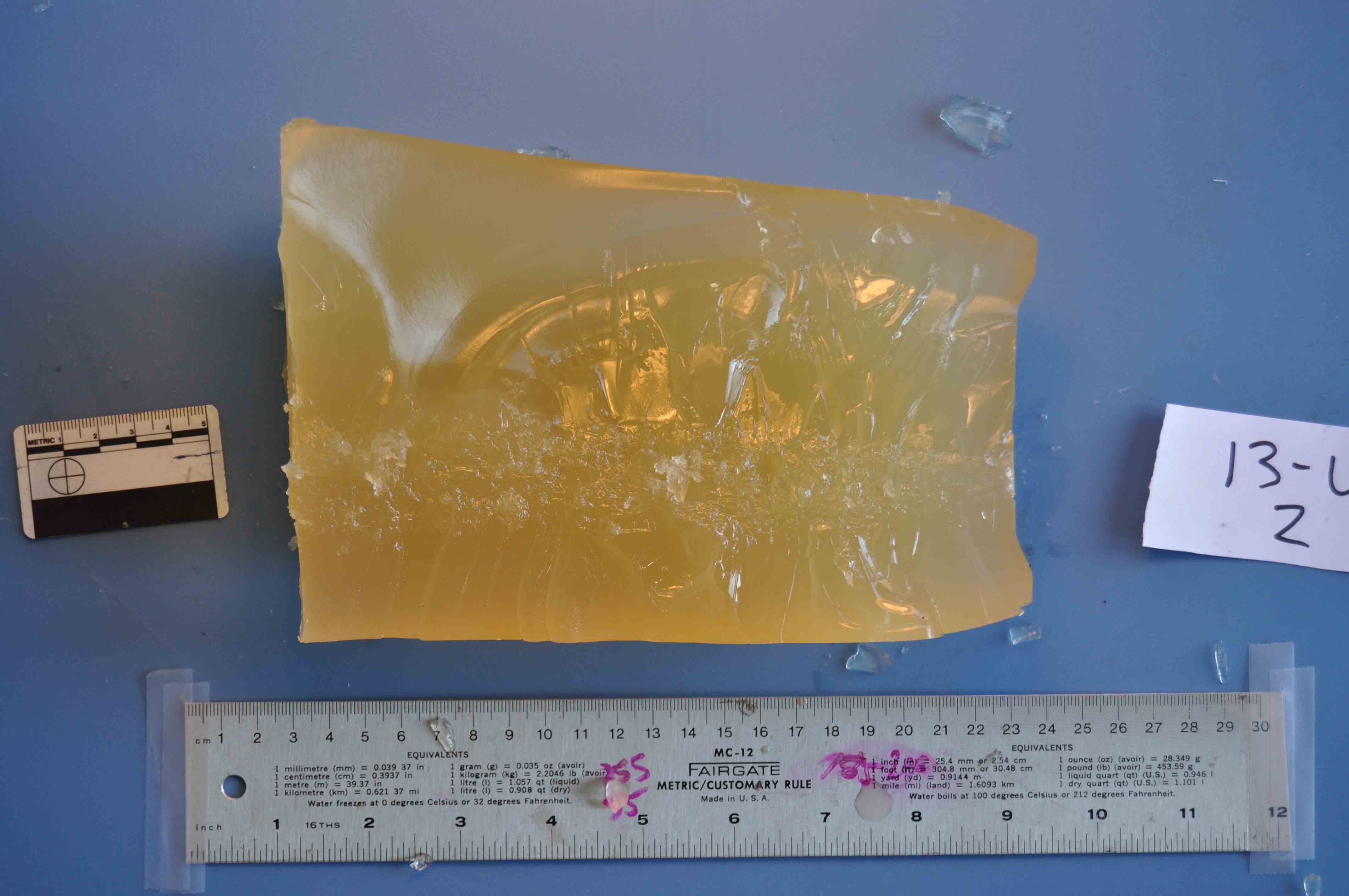

Supplement: File S2 — Wound track images, shapefiles, and tps files. (ZIP) [file pone.0104514.s002.zip › File S2/JPEGS/U2-2a.jpg]

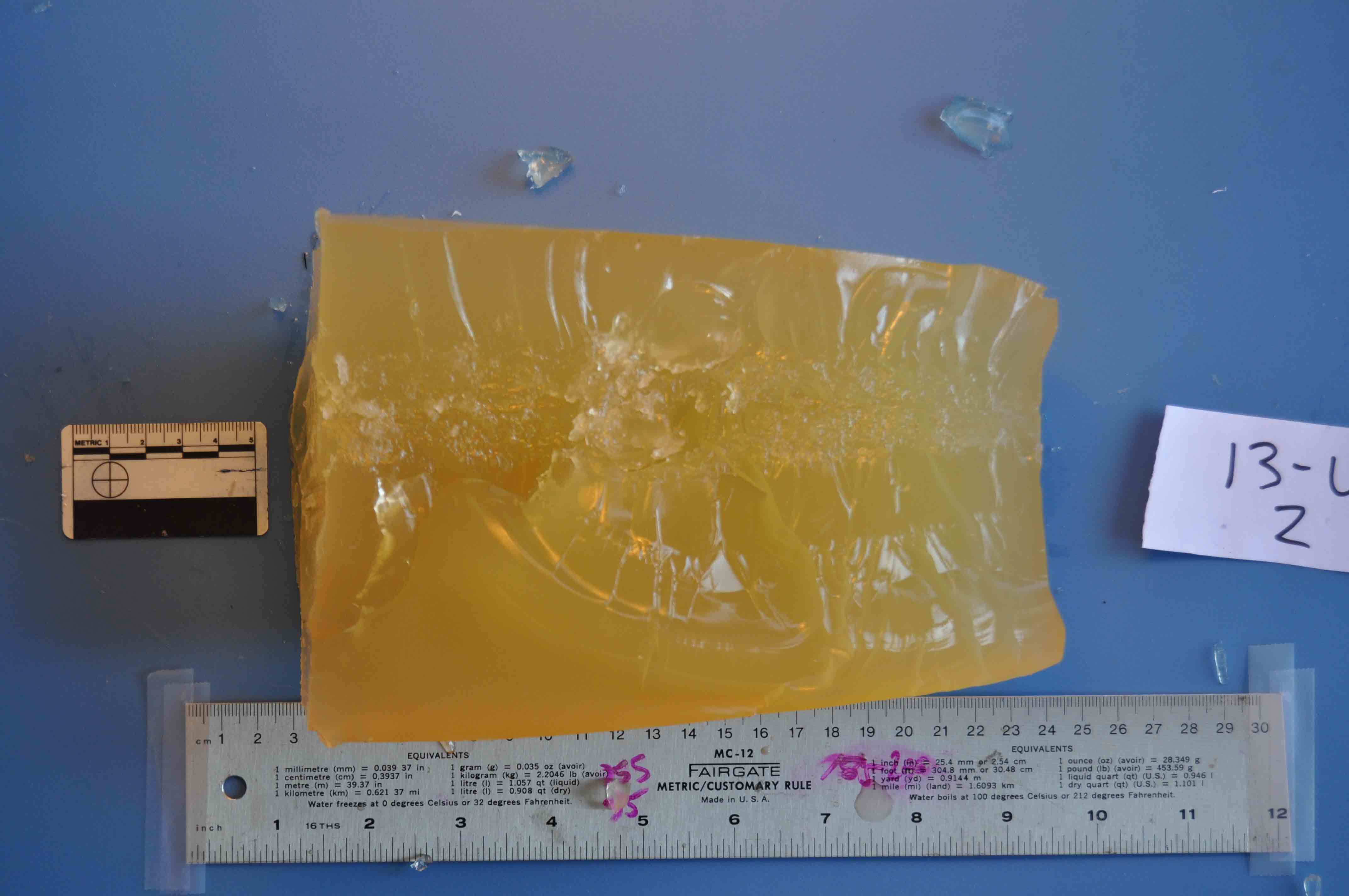

Supplement: File S2 — Wound track images, shapefiles, and tps files. (ZIP) [file pone.0104514.s002.zip › File S2/JPEGS/U2-2b.jpg]

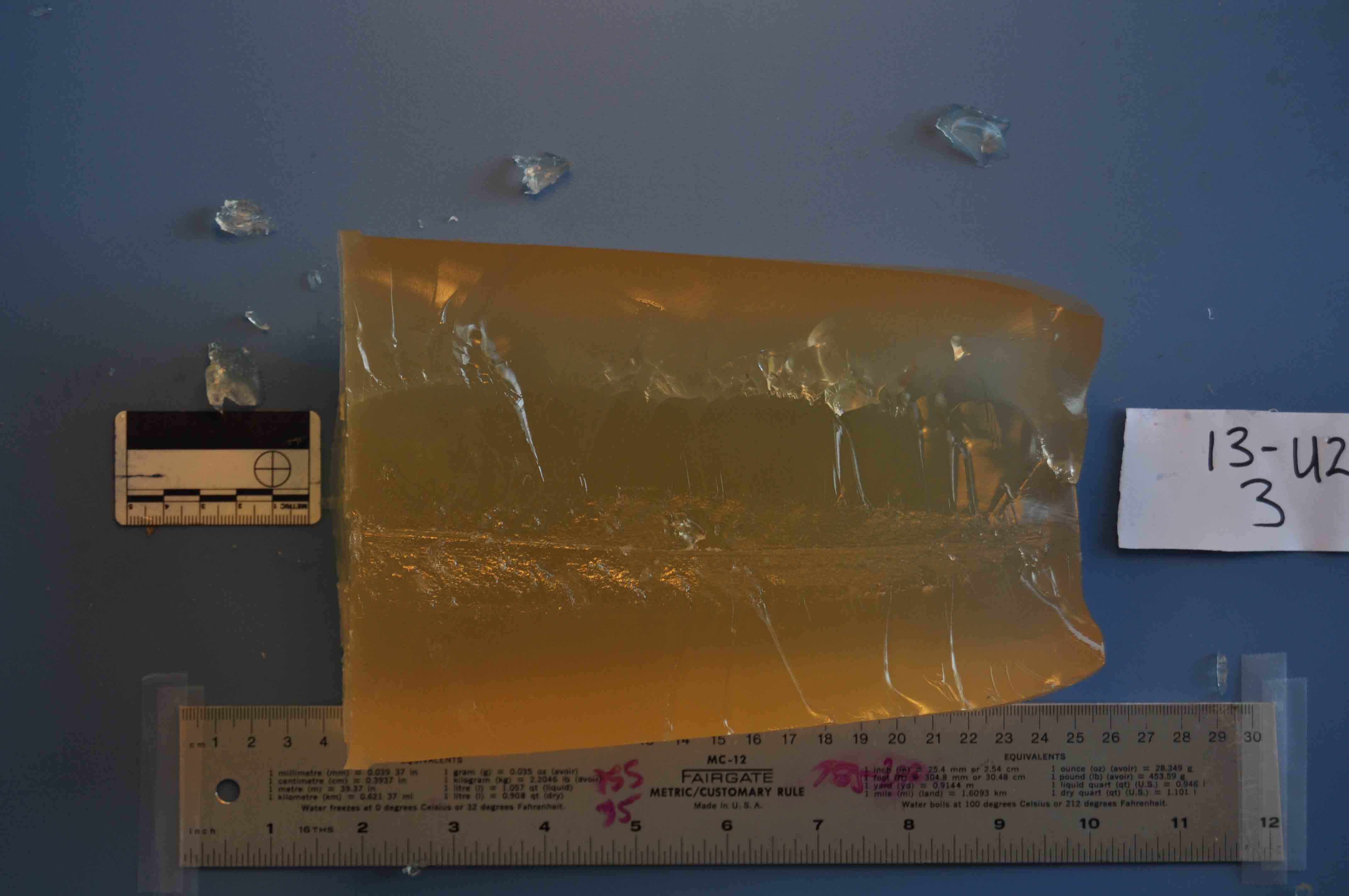

Supplement: File S2 — Wound track images, shapefiles, and tps files. (ZIP) [file pone.0104514.s002.zip › File S2/JPEGS/U2-3a.jpg]

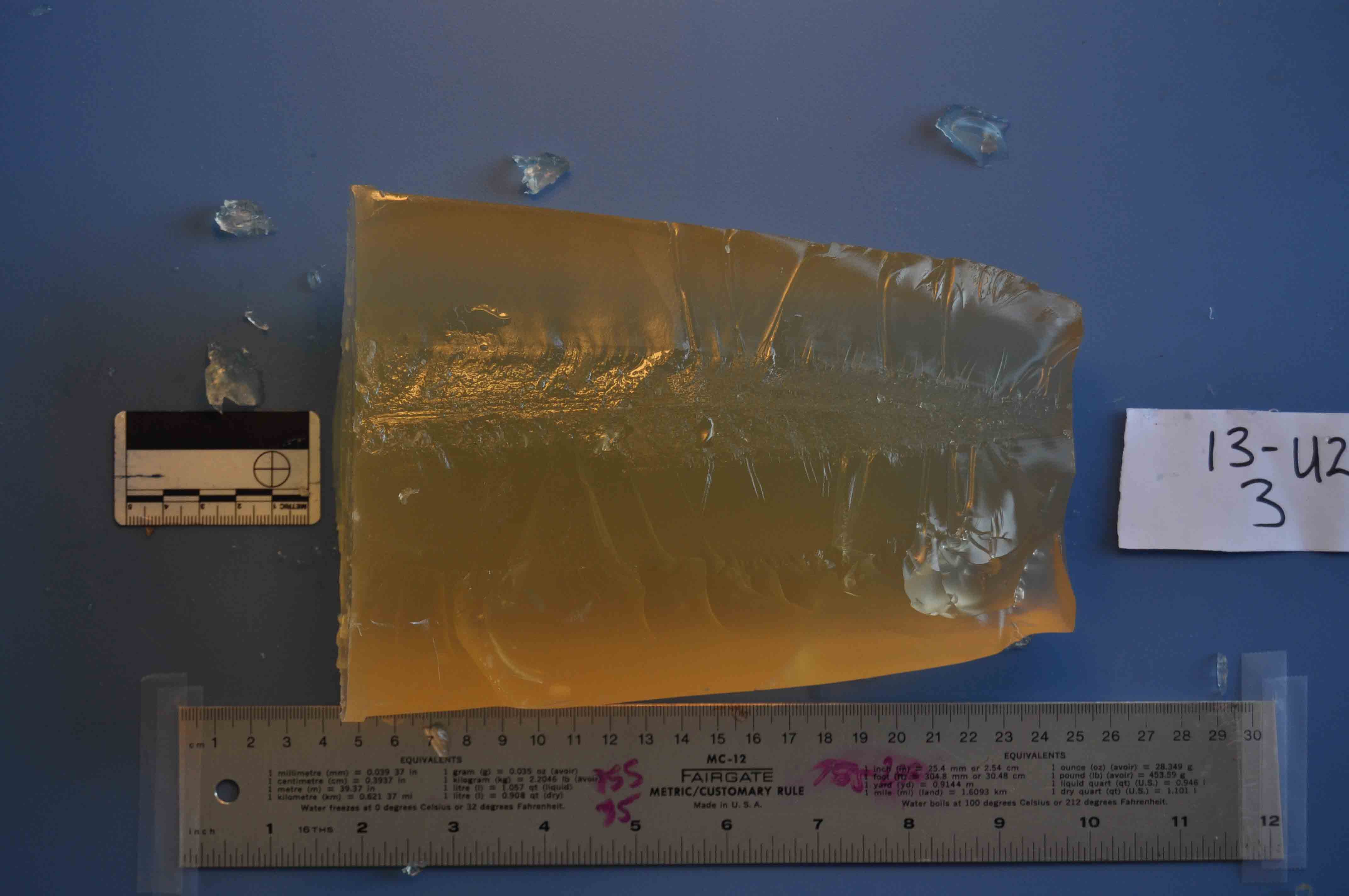

Supplement: File S2 — Wound track images, shapefiles, and tps files. (ZIP) [file pone.0104514.s002.zip › File S2/JPEGS/U2-3b.jpg]

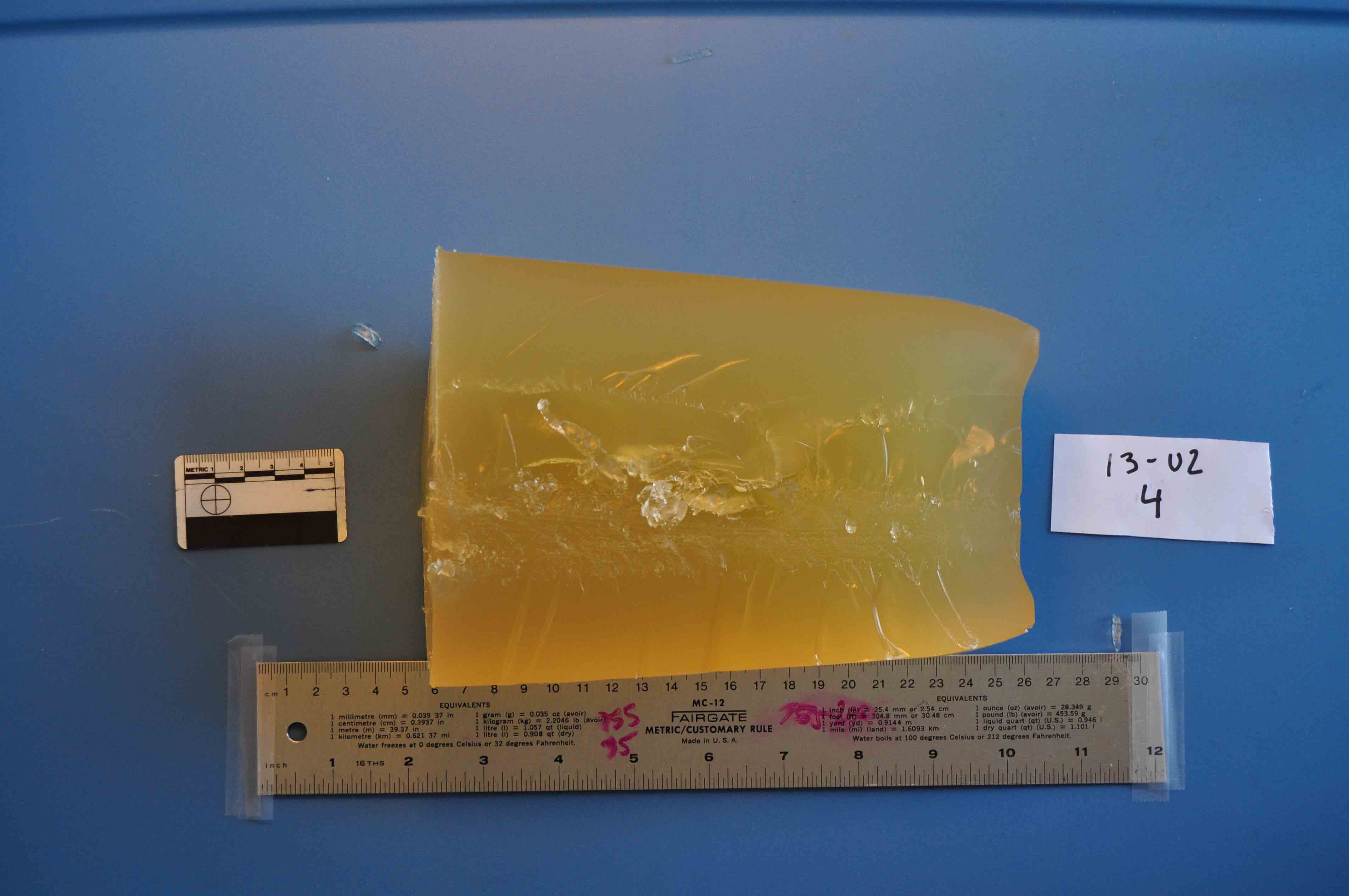

Supplement: File S2 — Wound track images, shapefiles, and tps files. (ZIP) [file pone.0104514.s002.zip › File S2/JPEGS/U2-4a.jpg]

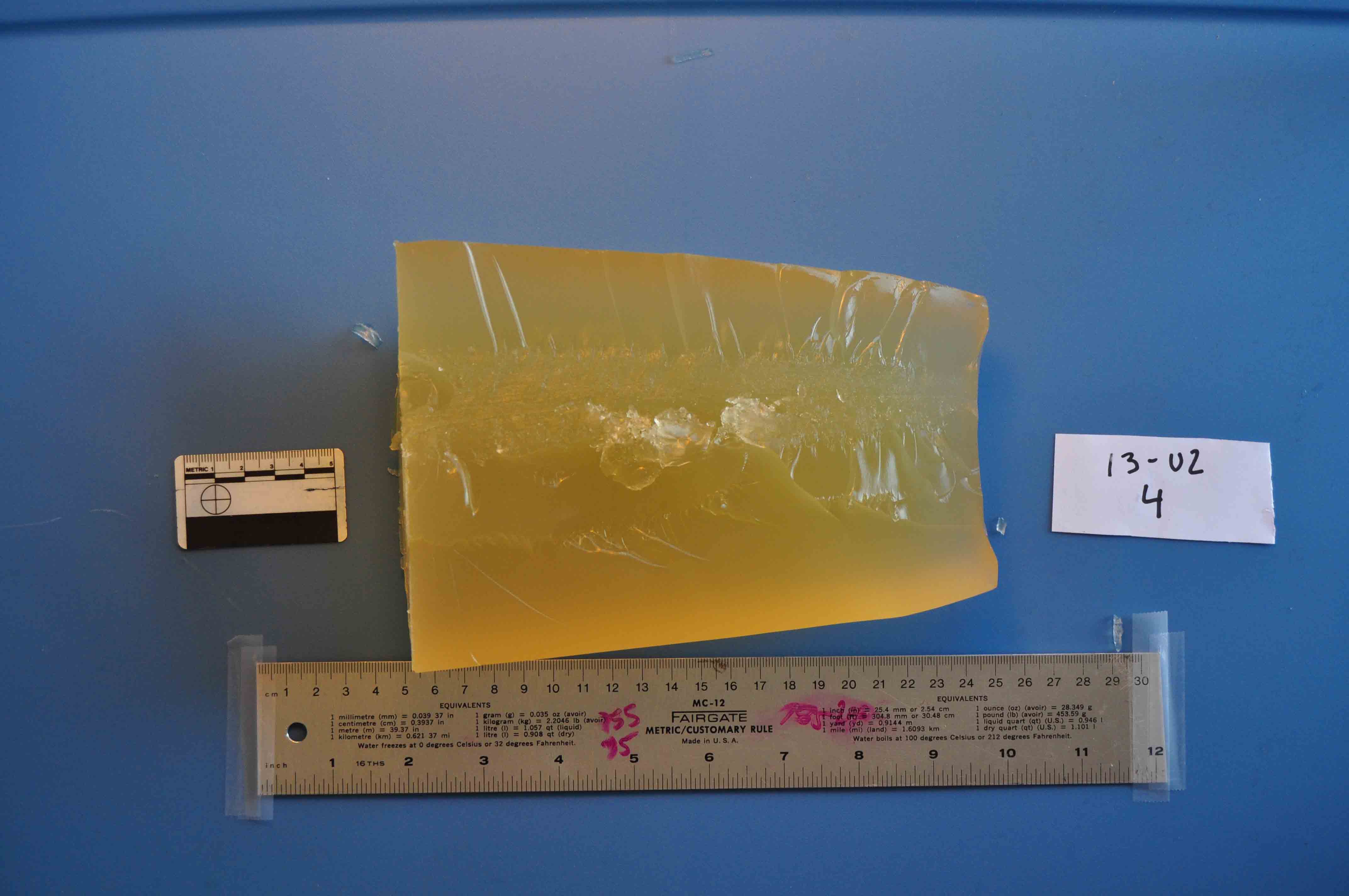

Supplement: File S2 — Wound track images, shapefiles, and tps files. (ZIP) [file pone.0104514.s002.zip › File S2/JPEGS/U2-4b.jpg]

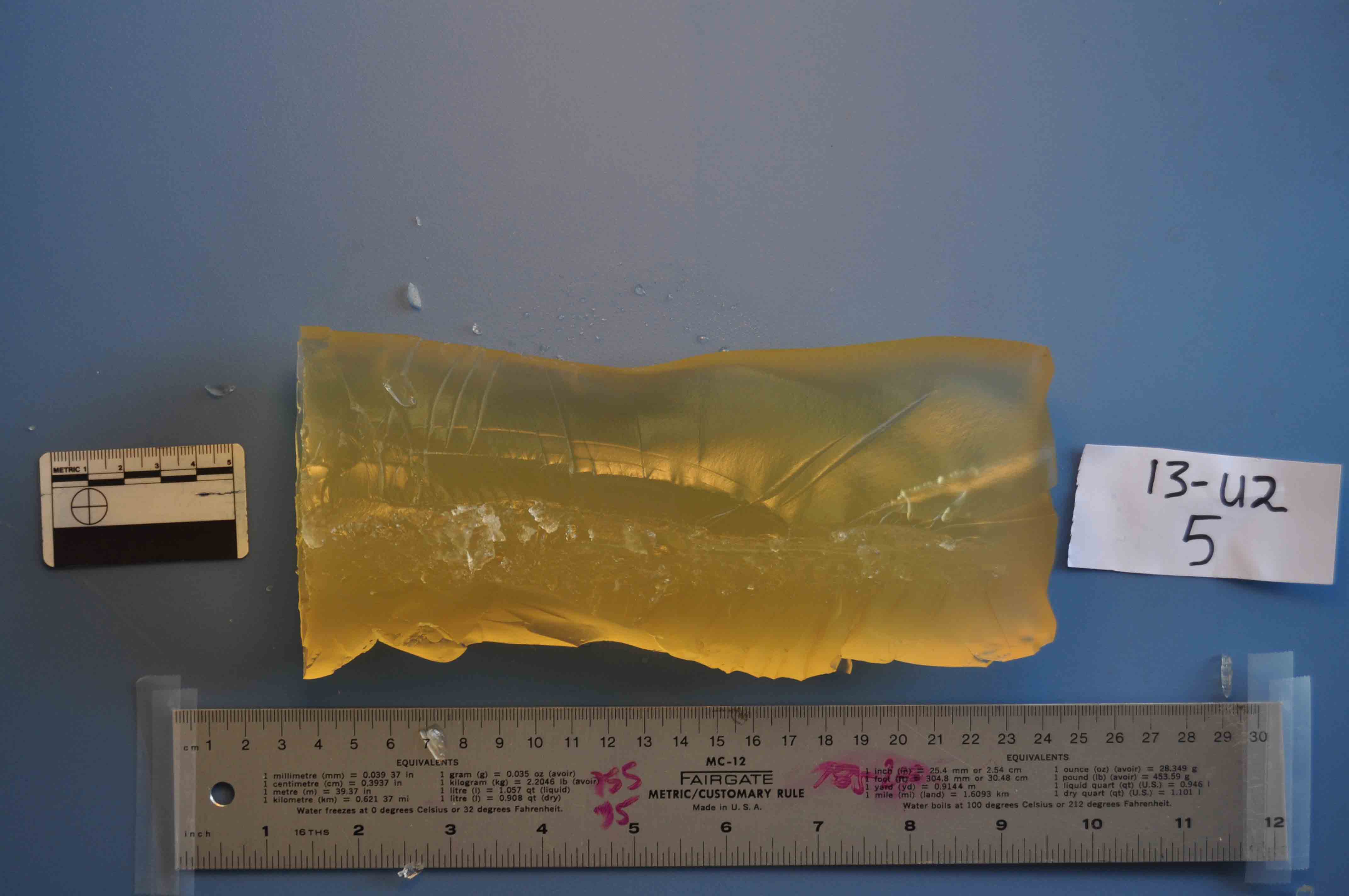

Supplement: File S2 — Wound track images, shapefiles, and tps files. (ZIP) [file pone.0104514.s002.zip › File S2/JPEGS/U2-5a.jpg]

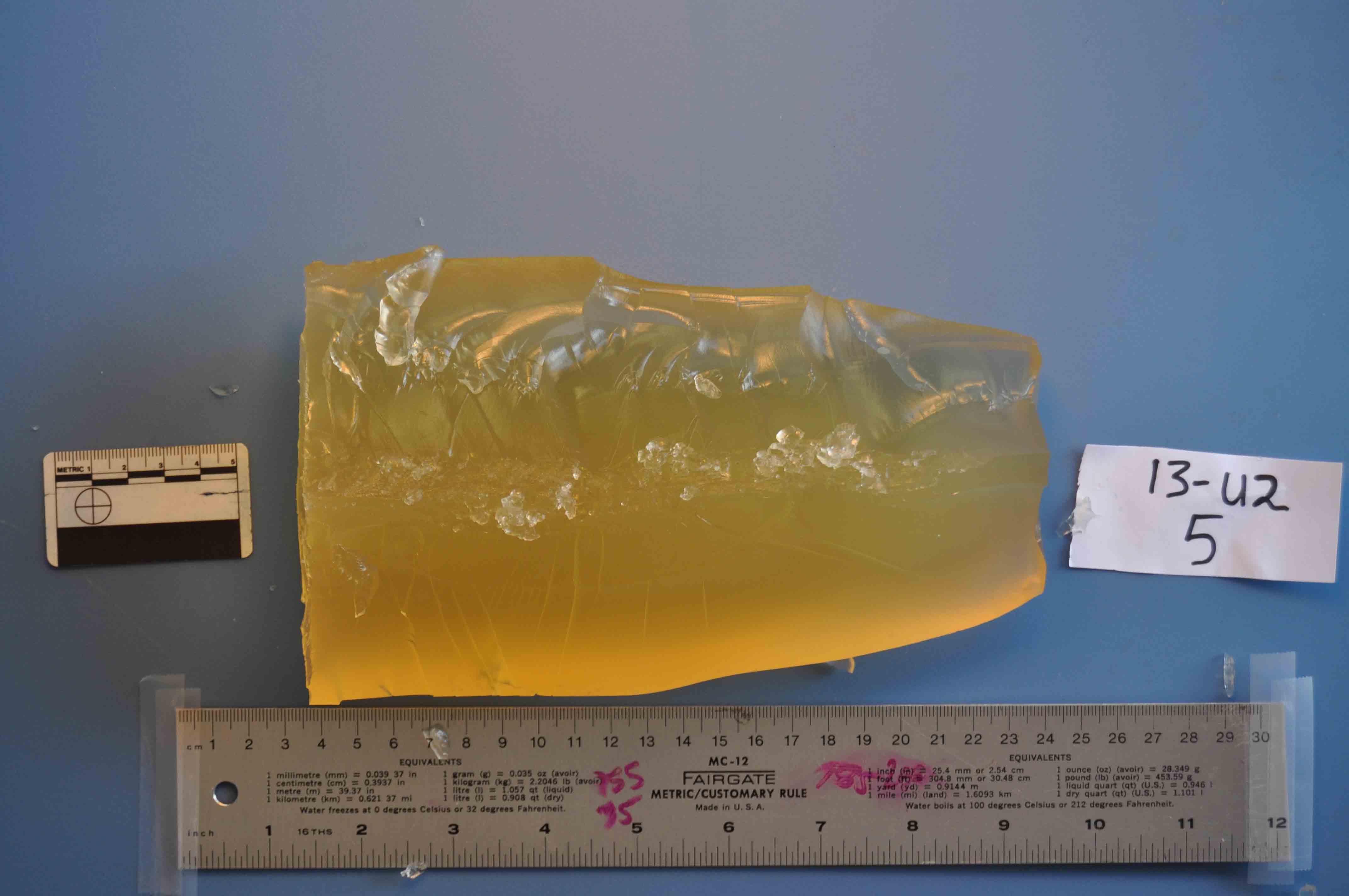

Supplement: File S2 — Wound track images, shapefiles, and tps files. (ZIP) [file pone.0104514.s002.zip › File S2/JPEGS/U2-5b.jpg]

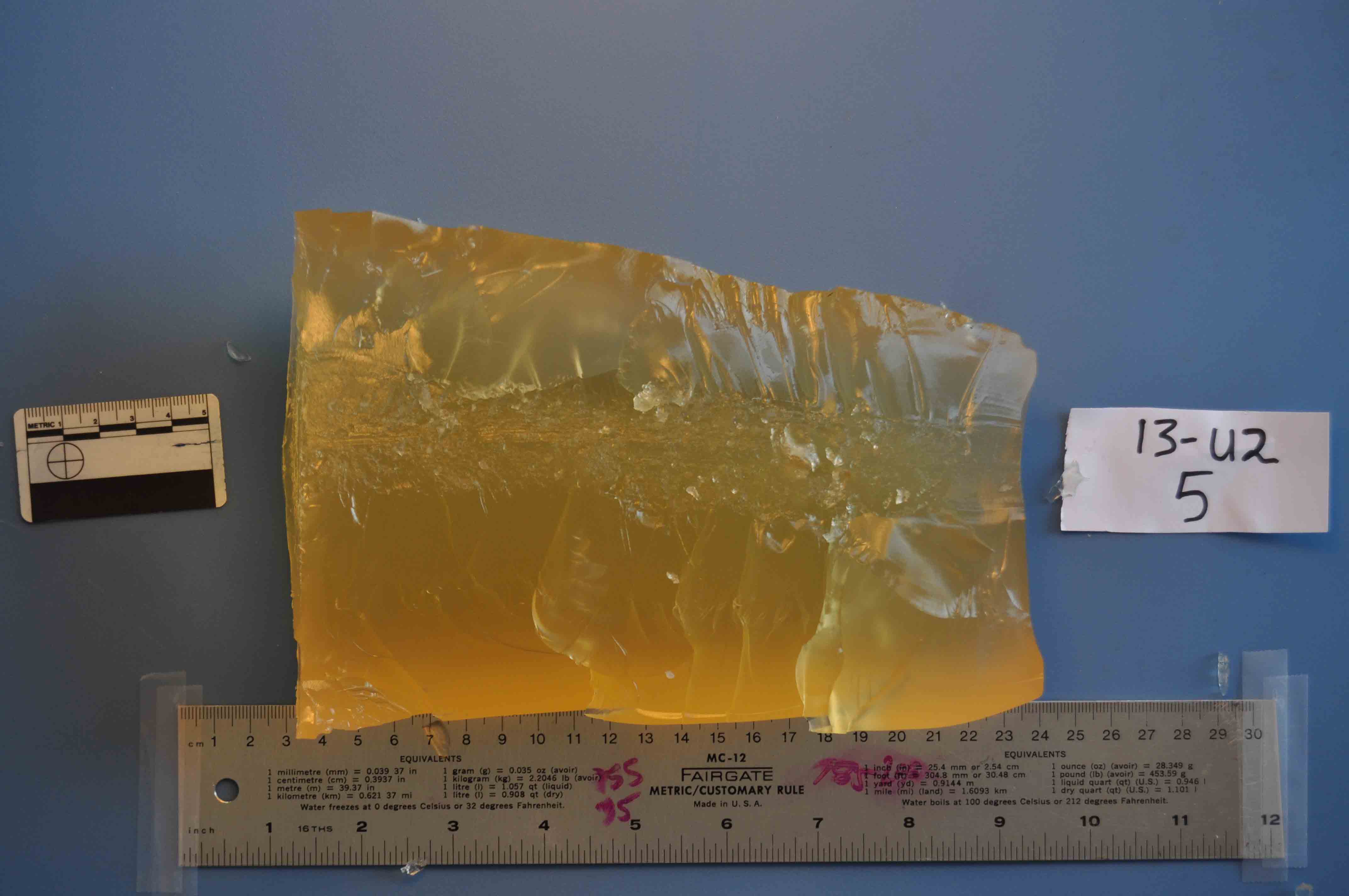

Supplement: File S2 — Wound track images, shapefiles, and tps files. (ZIP) [file pone.0104514.s002.zip › File S2/JPEGS/U2-5c.jpg]

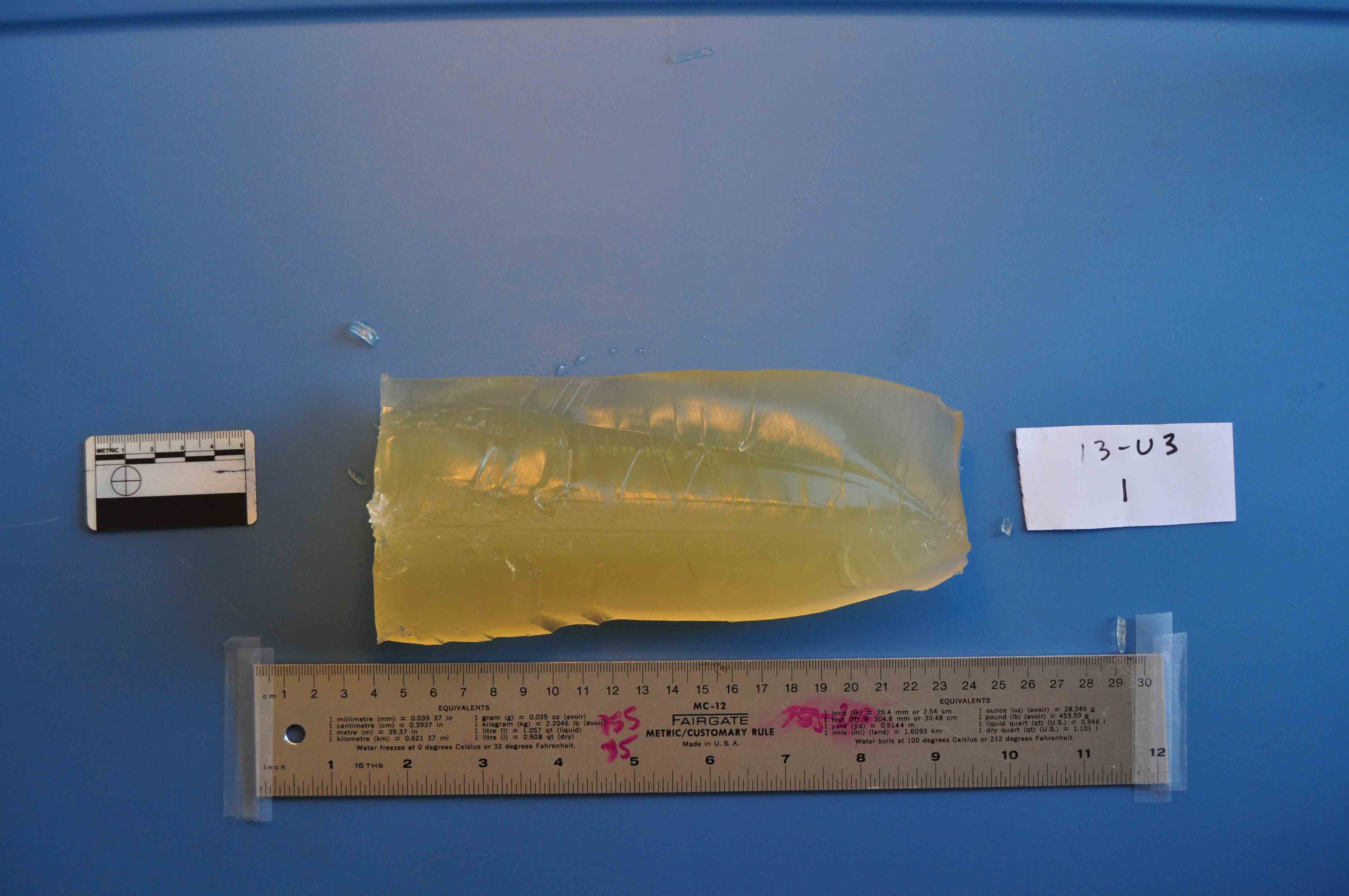

Supplement: File S2 — Wound track images, shapefiles, and tps files. (ZIP) [file pone.0104514.s002.zip › File S2/JPEGS/U3-1a.jpg]

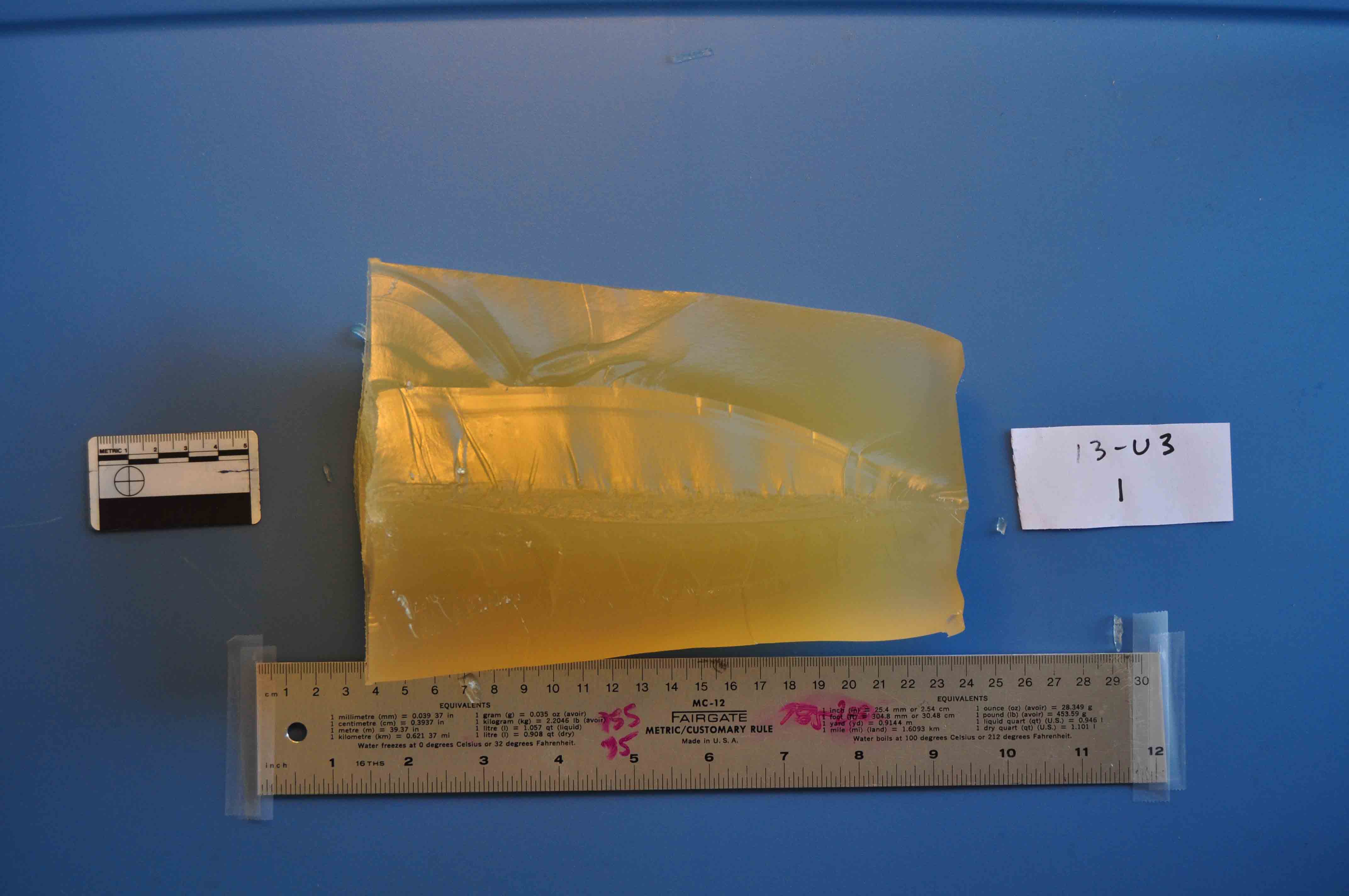

Supplement: File S2 — Wound track images, shapefiles, and tps files. (ZIP) [file pone.0104514.s002.zip › File S2/JPEGS/U3-1b.jpg]

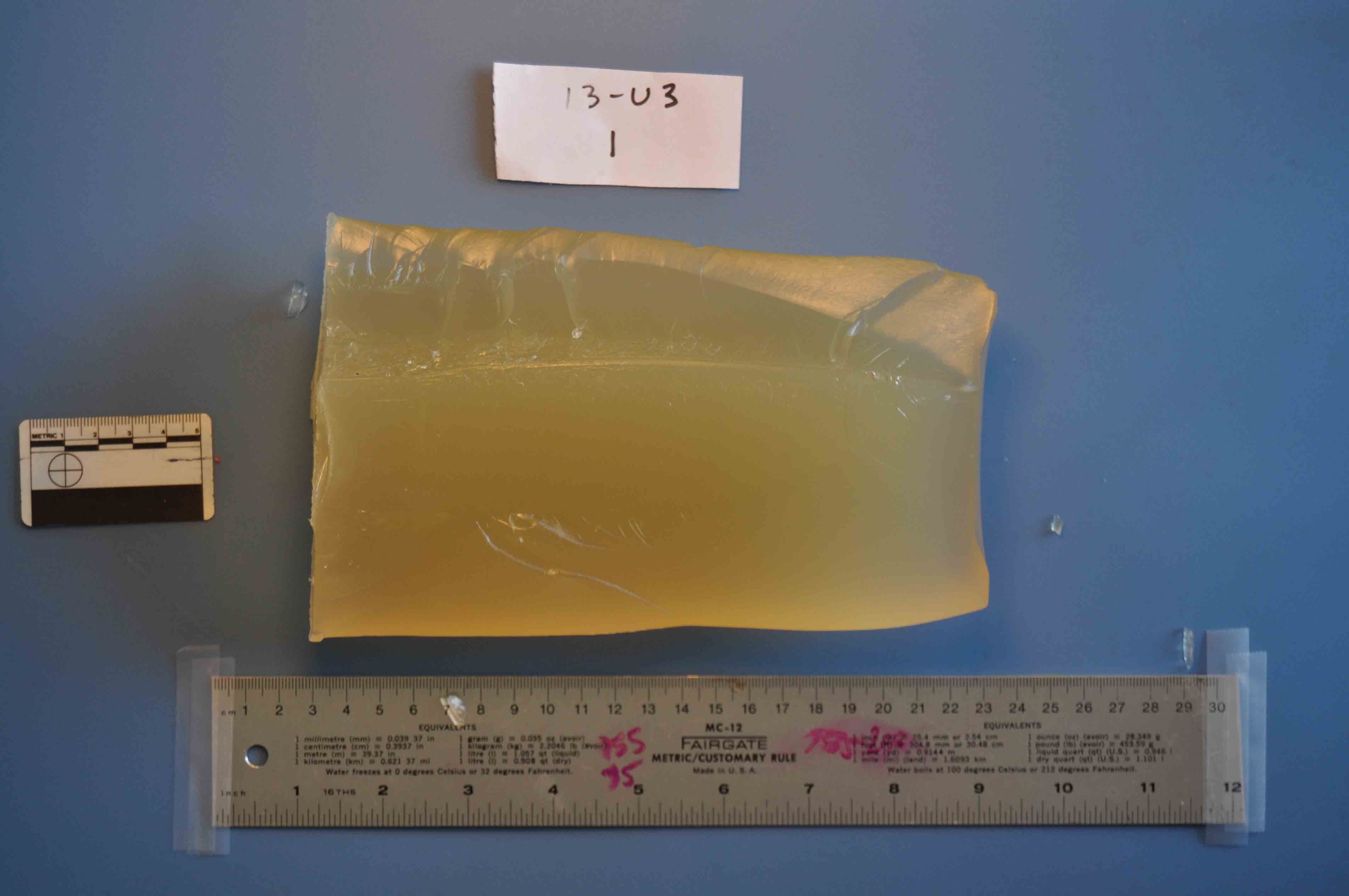

Supplement: File S2 — Wound track images, shapefiles, and tps files. (ZIP) [file pone.0104514.s002.zip › File S2/JPEGS/U3-1c.jpg]

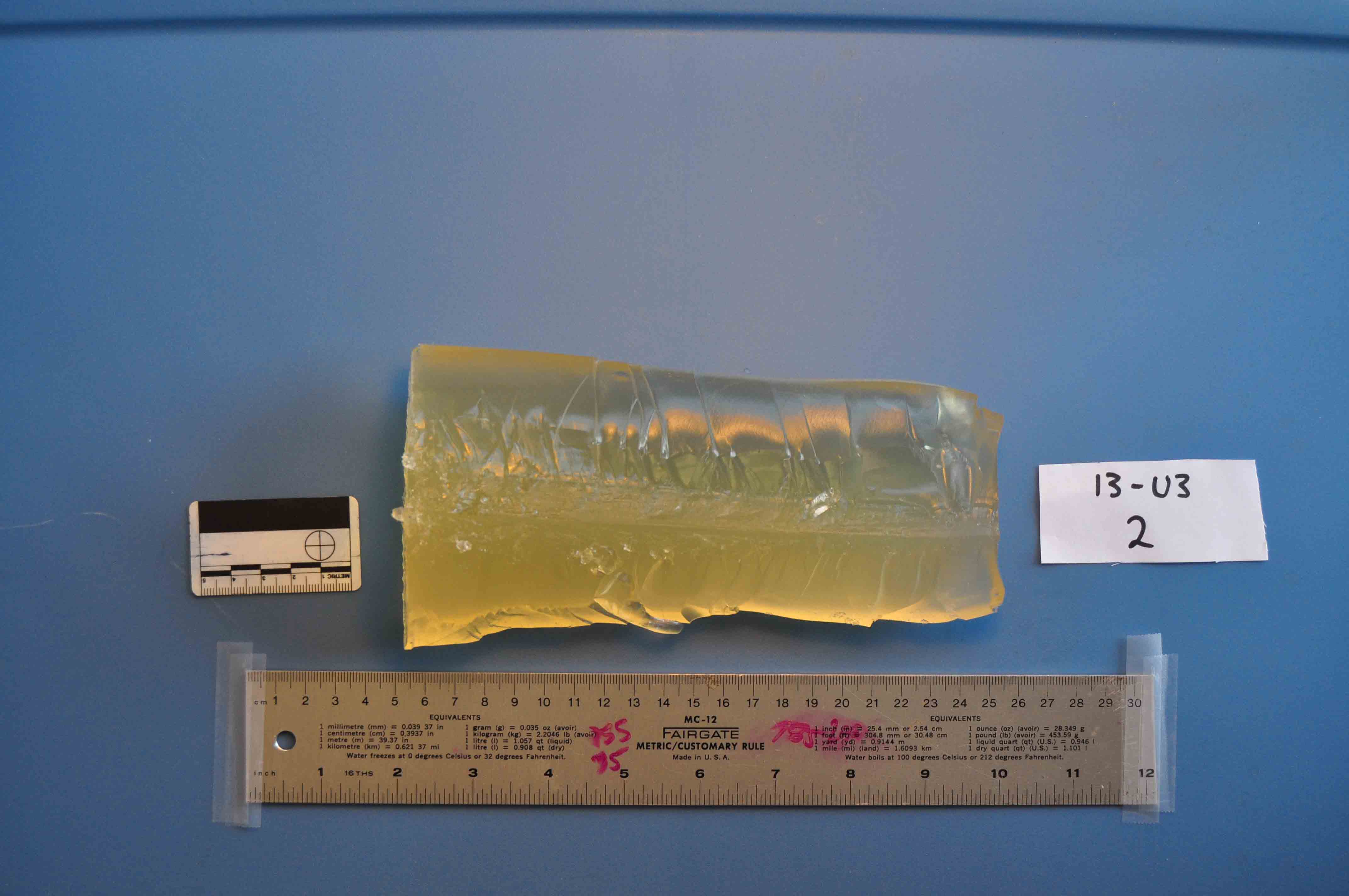

Supplement: File S2 — Wound track images, shapefiles, and tps files. (ZIP) [file pone.0104514.s002.zip › File S2/JPEGS/U3-2a.jpg]

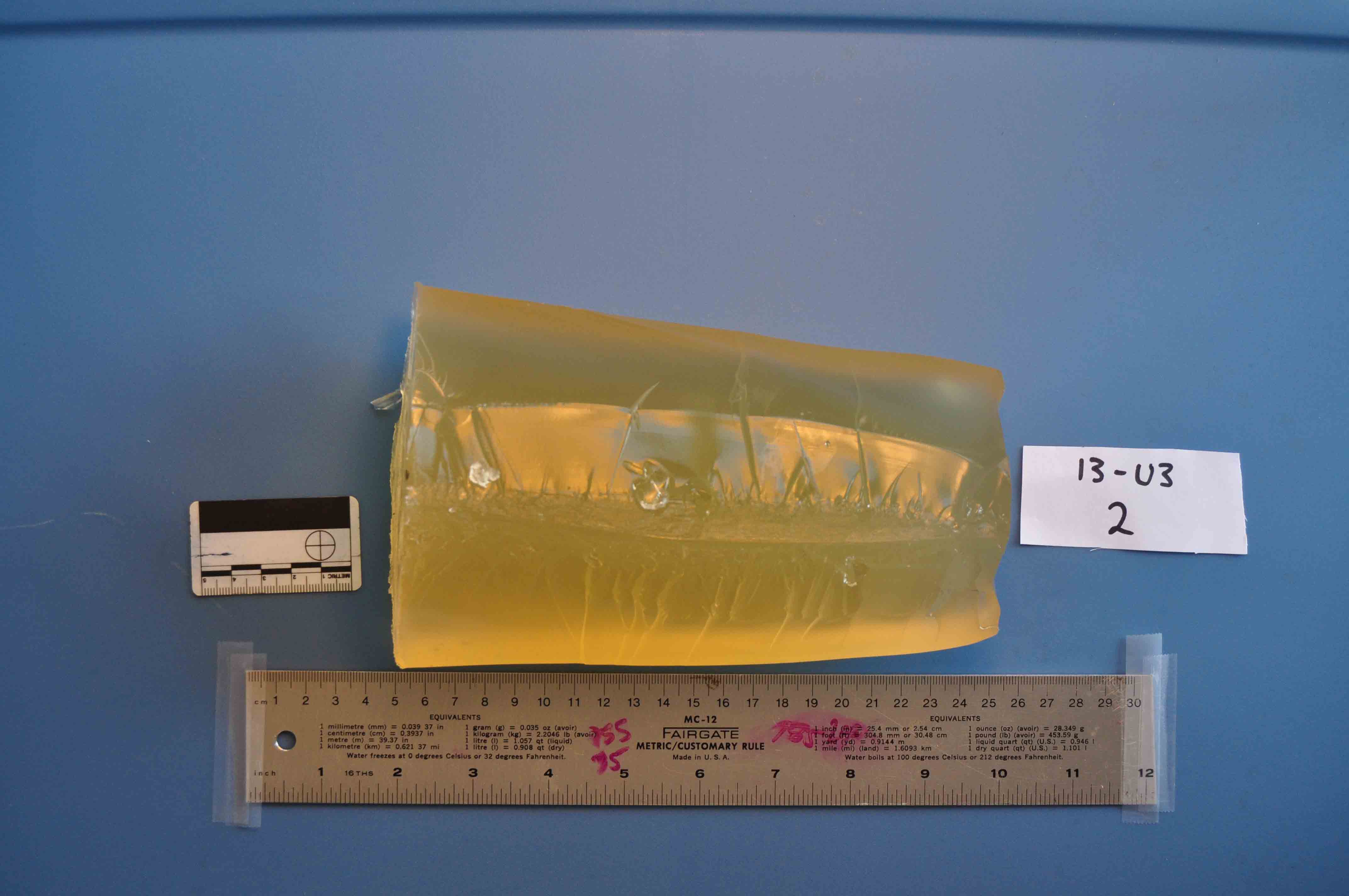

Supplement: File S2 — Wound track images, shapefiles, and tps files. (ZIP) [file pone.0104514.s002.zip › File S2/JPEGS/U3-2b.jpg]

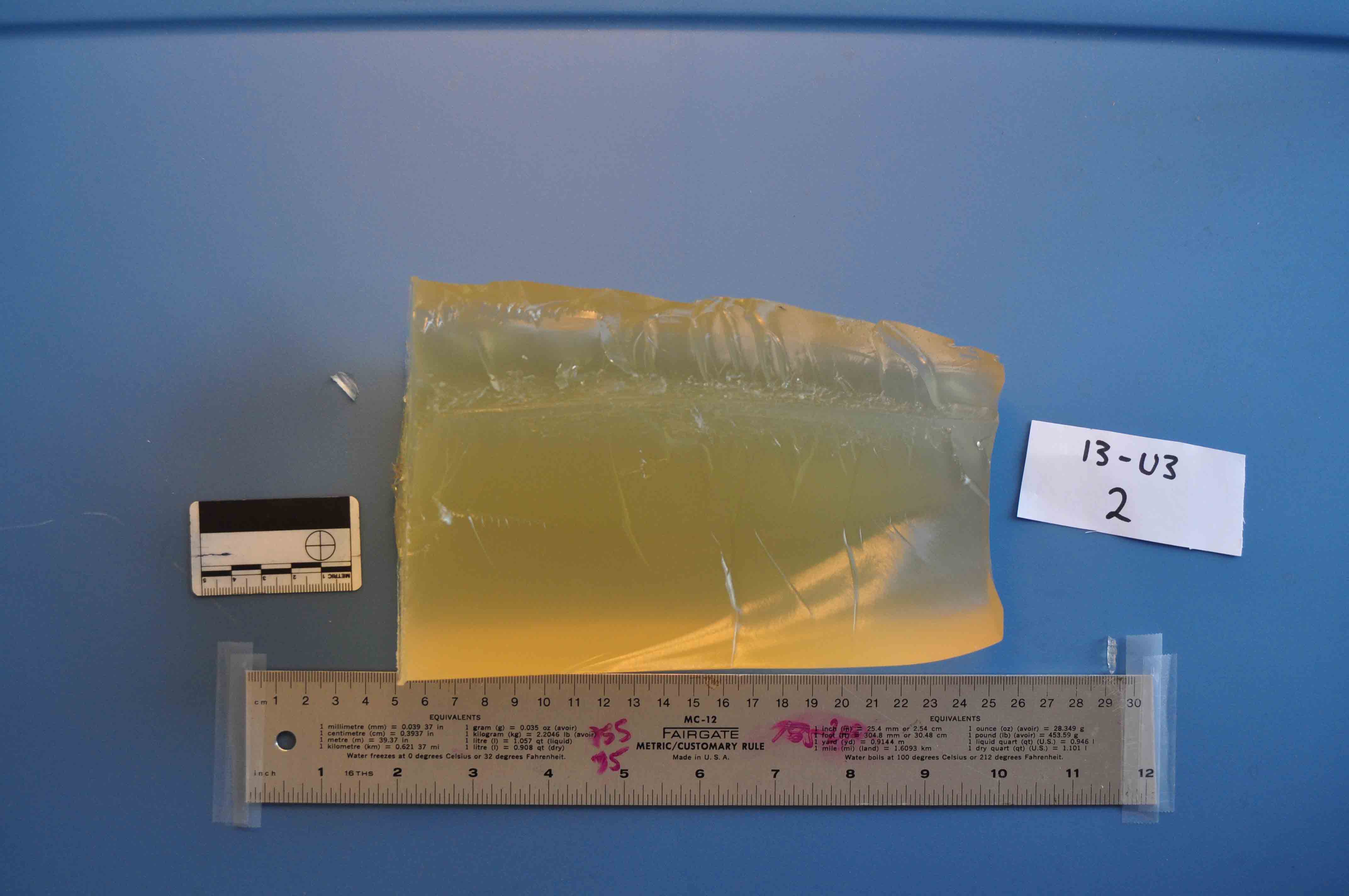

Supplement: File S2 — Wound track images, shapefiles, and tps files. (ZIP) [file pone.0104514.s002.zip › File S2/JPEGS/U3-2c.jpg]

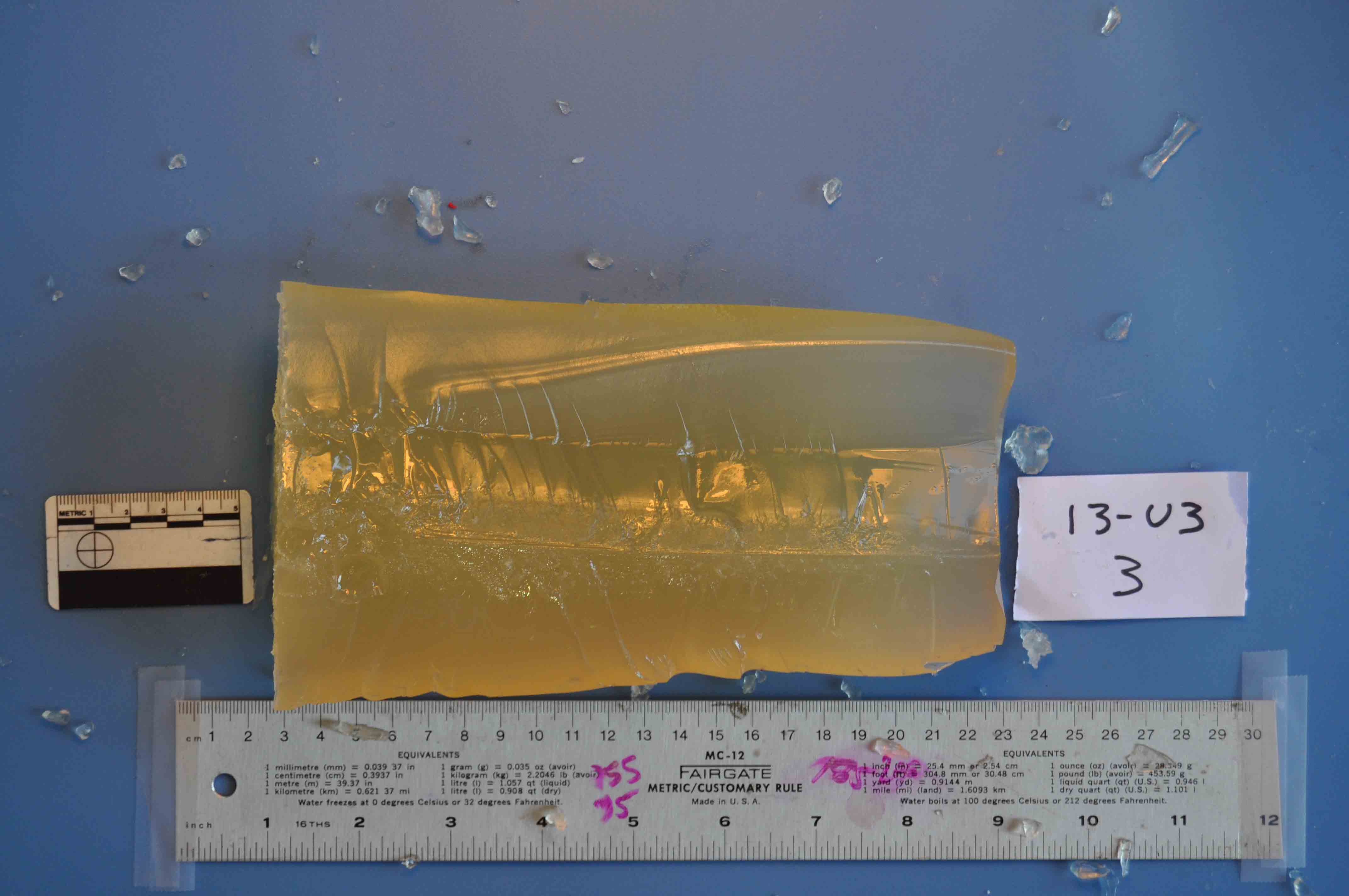

Supplement: File S2 — Wound track images, shapefiles, and tps files. (ZIP) [file pone.0104514.s002.zip › File S2/JPEGS/U3-3a.jpg]

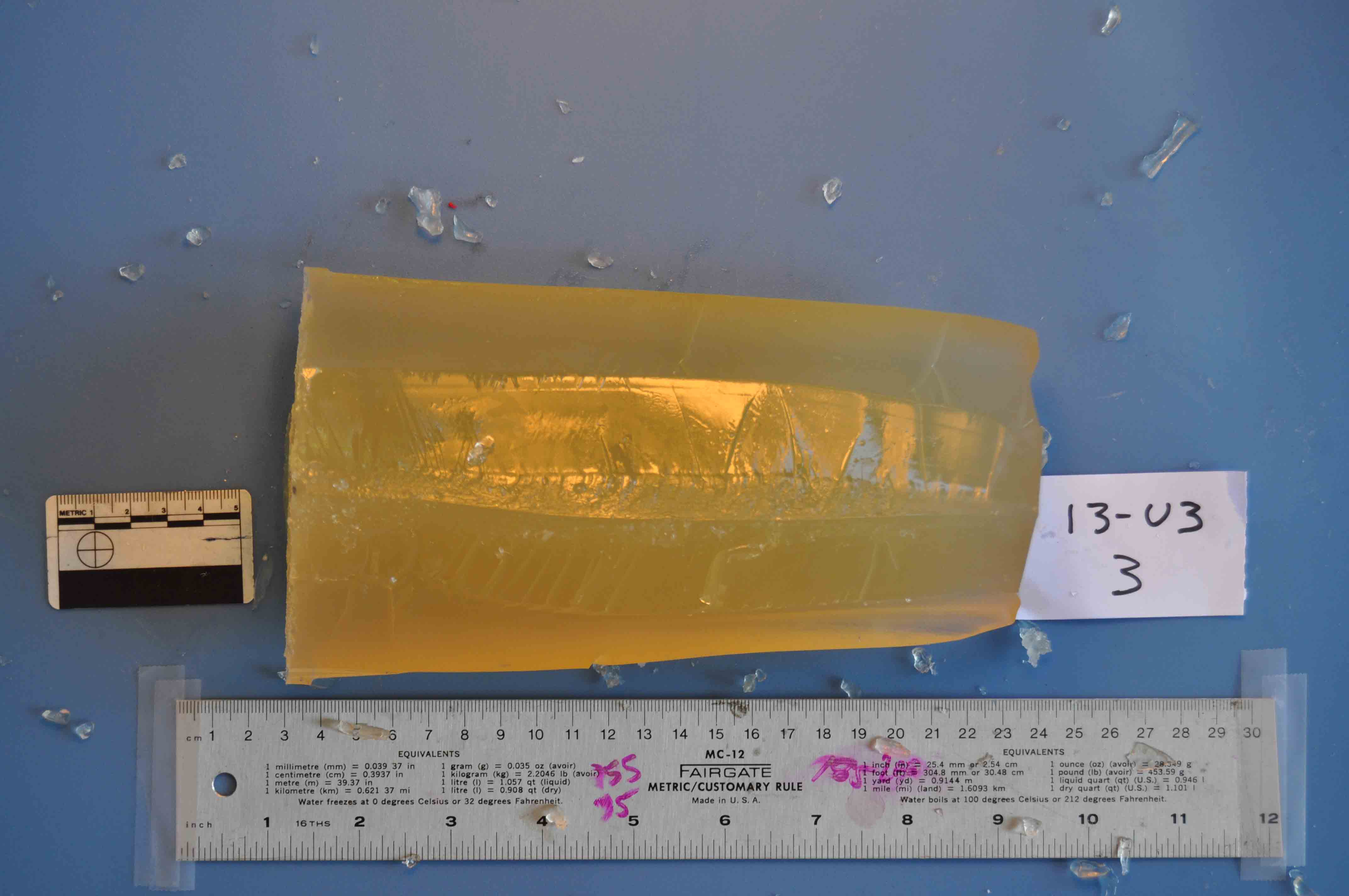

Supplement: File S2 — Wound track images, shapefiles, and tps files. (ZIP) [file pone.0104514.s002.zip › File S2/JPEGS/U3-3c.jpg]

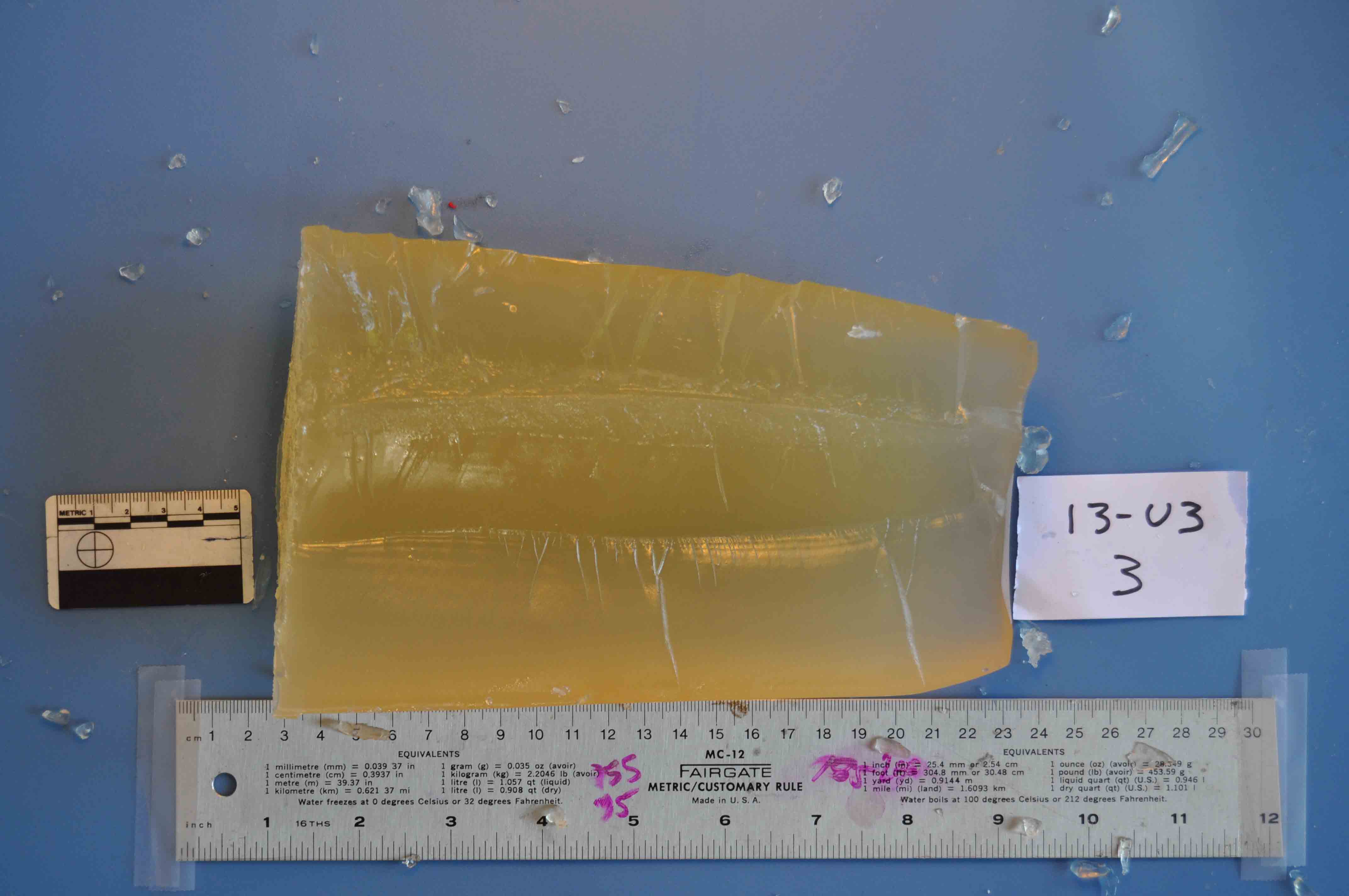

Supplement: File S2 — Wound track images, shapefiles, and tps files. (ZIP) [file pone.0104514.s002.zip › File S2/JPEGS/U3-3d.jpg]

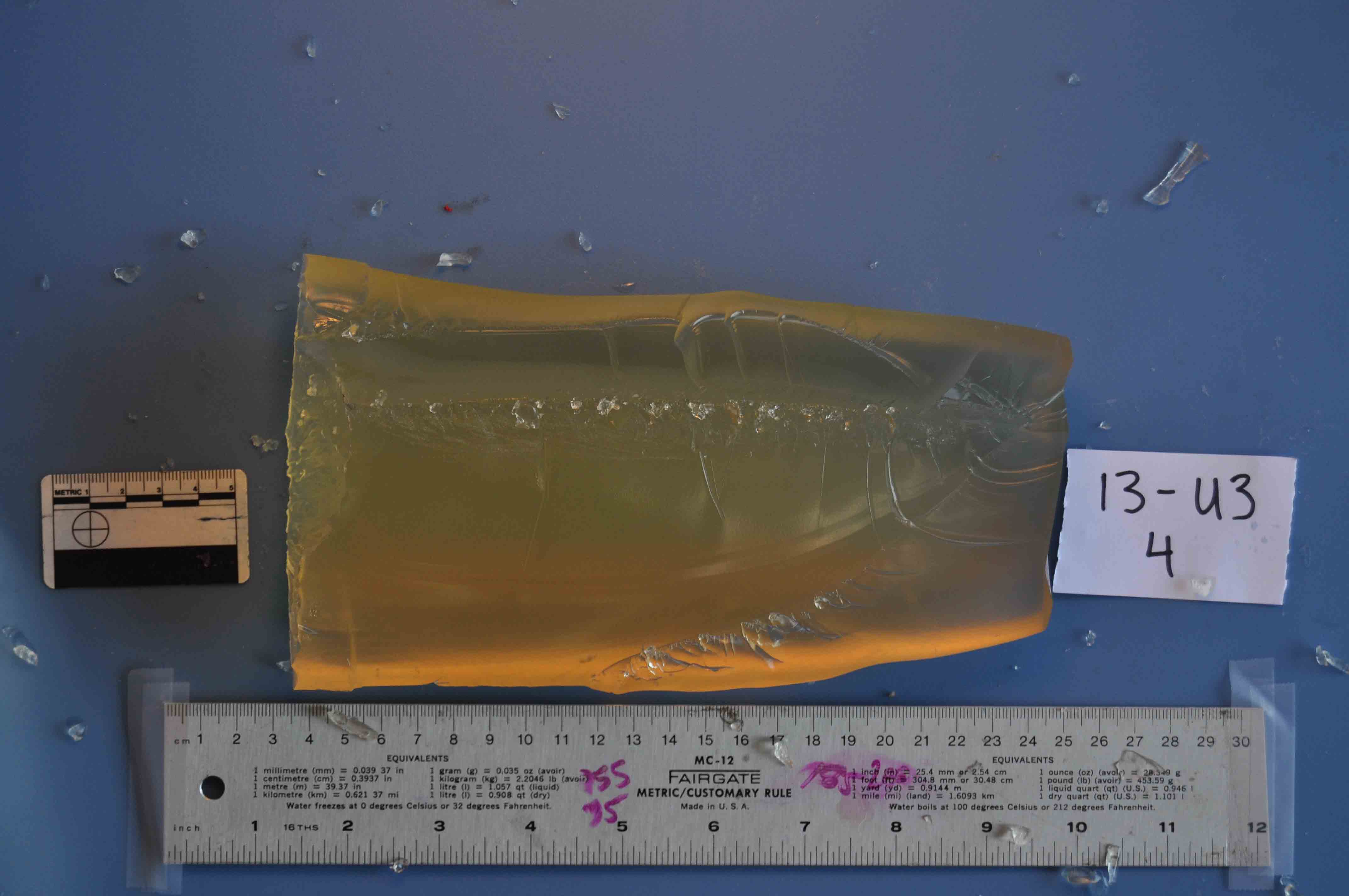

Supplement: File S2 — Wound track images, shapefiles, and tps files. (ZIP) [file pone.0104514.s002.zip › File S2/JPEGS/U3-4a.jpg]

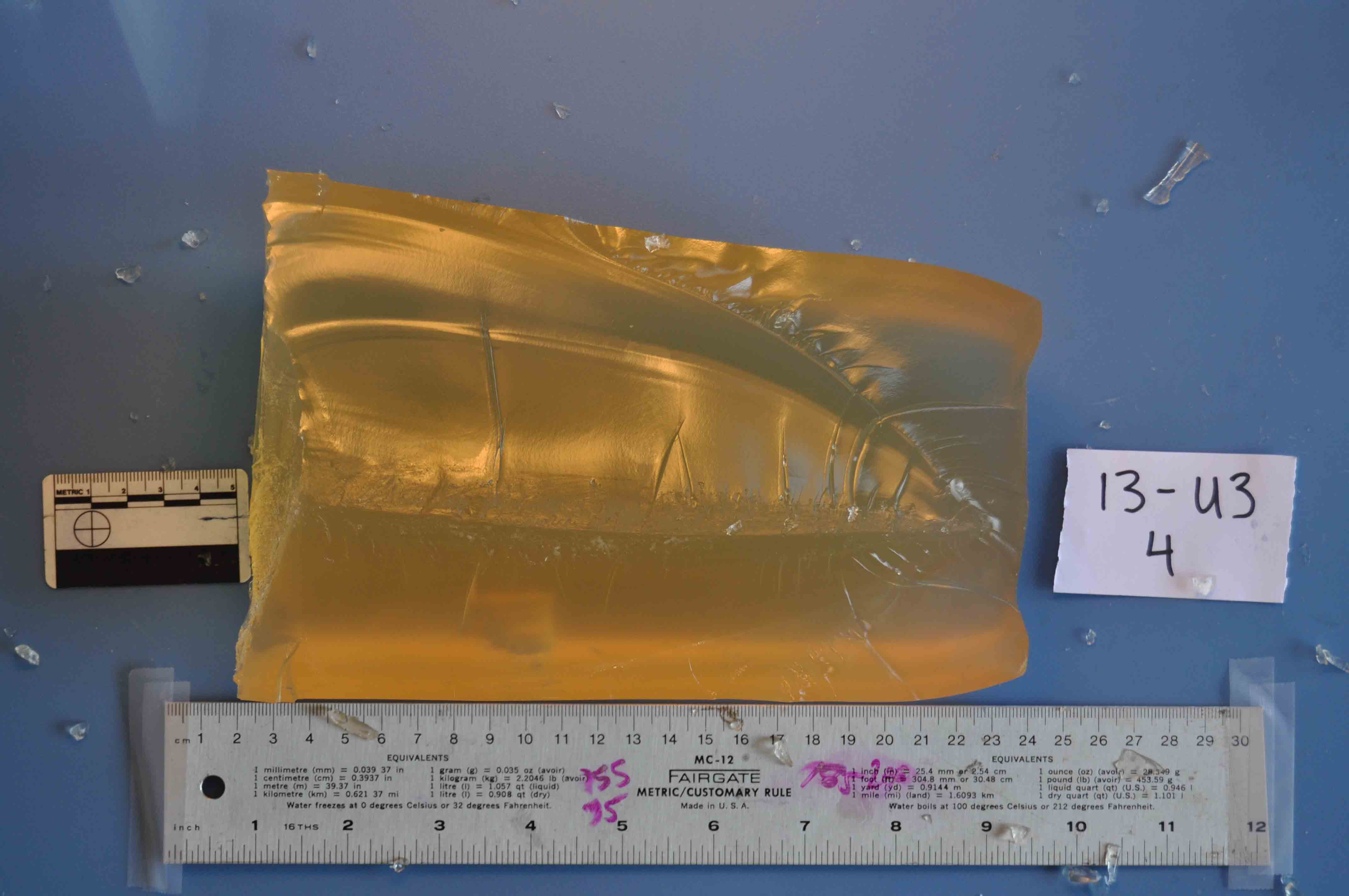

Supplement: File S2 — Wound track images, shapefiles, and tps files. (ZIP) [file pone.0104514.s002.zip › File S2/JPEGS/U3-4b.jpg]

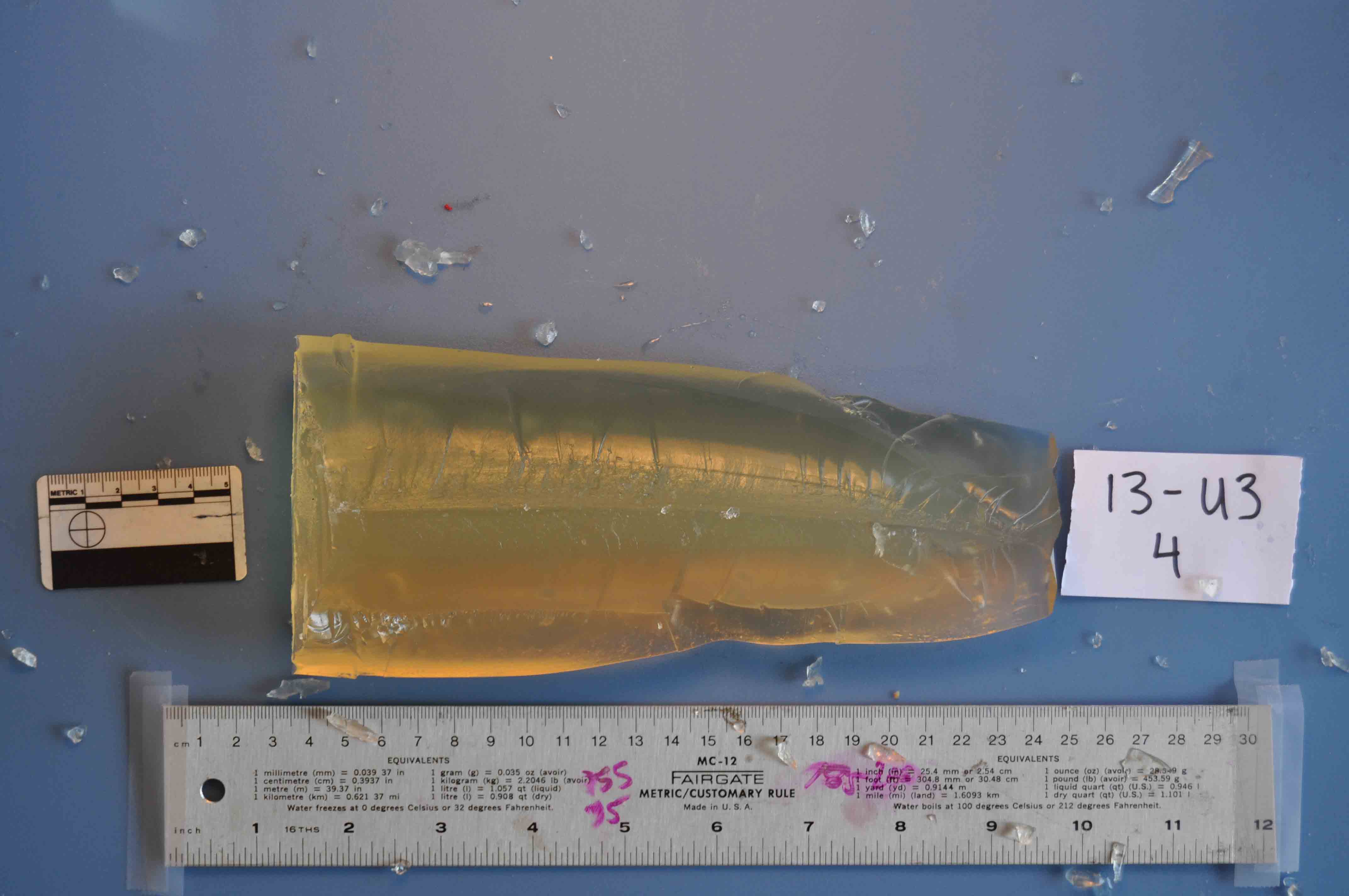

Supplement: File S2 — Wound track images, shapefiles, and tps files. (ZIP) [file pone.0104514.s002.zip › File S2/JPEGS/U3-4c.jpg]

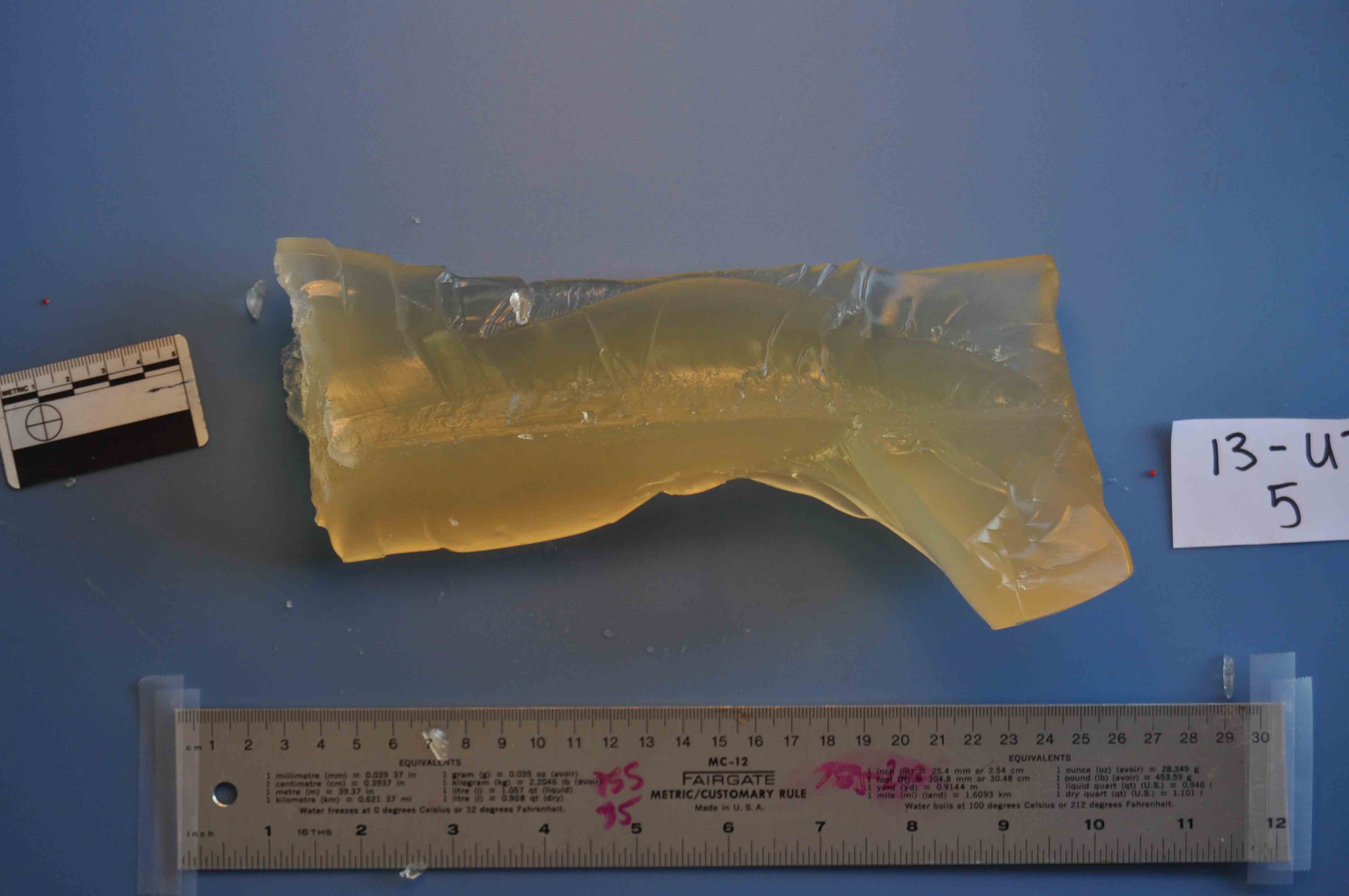

Supplement: File S2 — Wound track images, shapefiles, and tps files. (ZIP) [file pone.0104514.s002.zip › File S2/JPEGS/U3-5a.jpg]

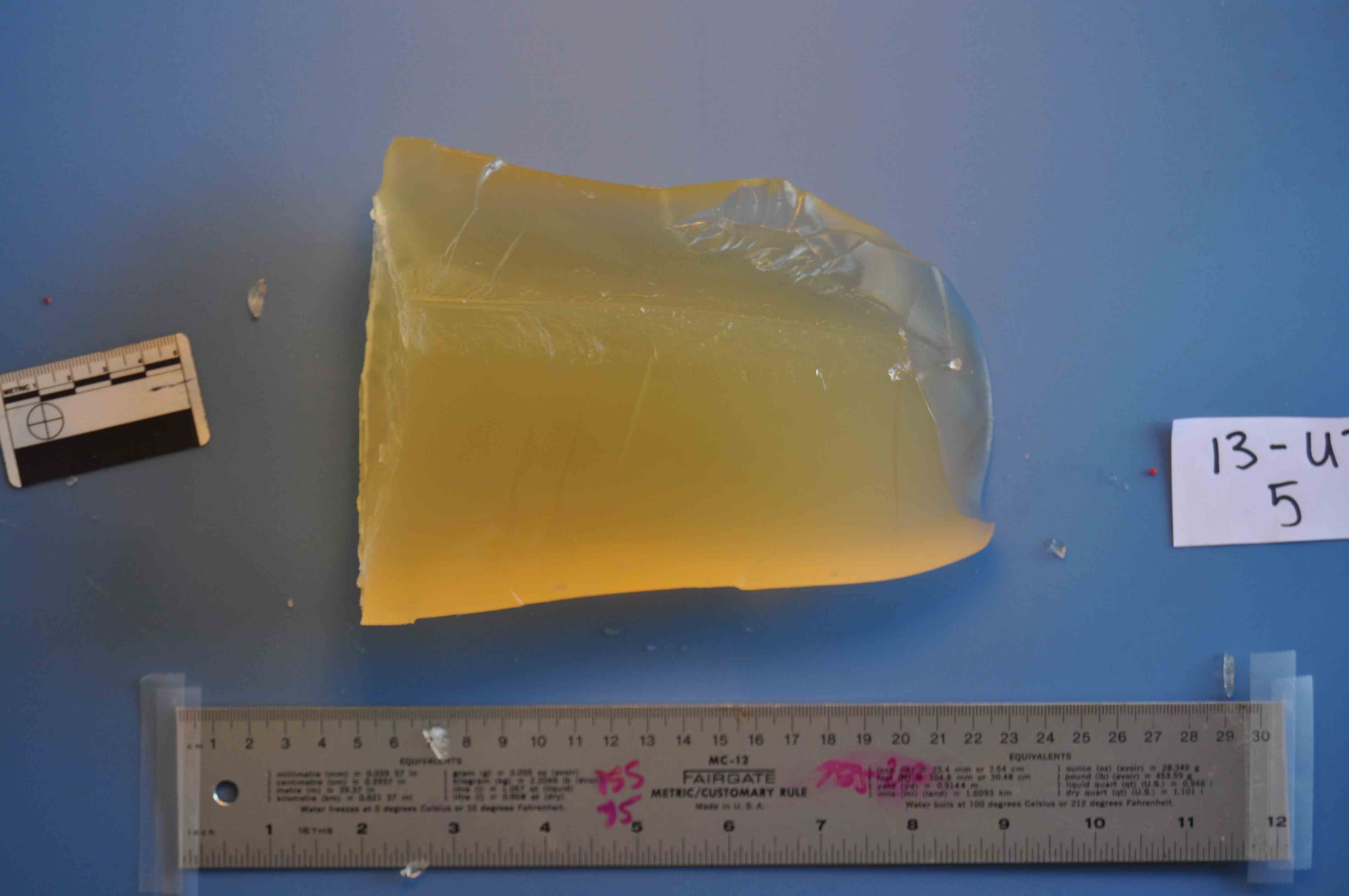

Supplement: File S2 — Wound track images, shapefiles, and tps files. (ZIP) [file pone.0104514.s002.zip › File S2/JPEGS/U3-5b.jpg]

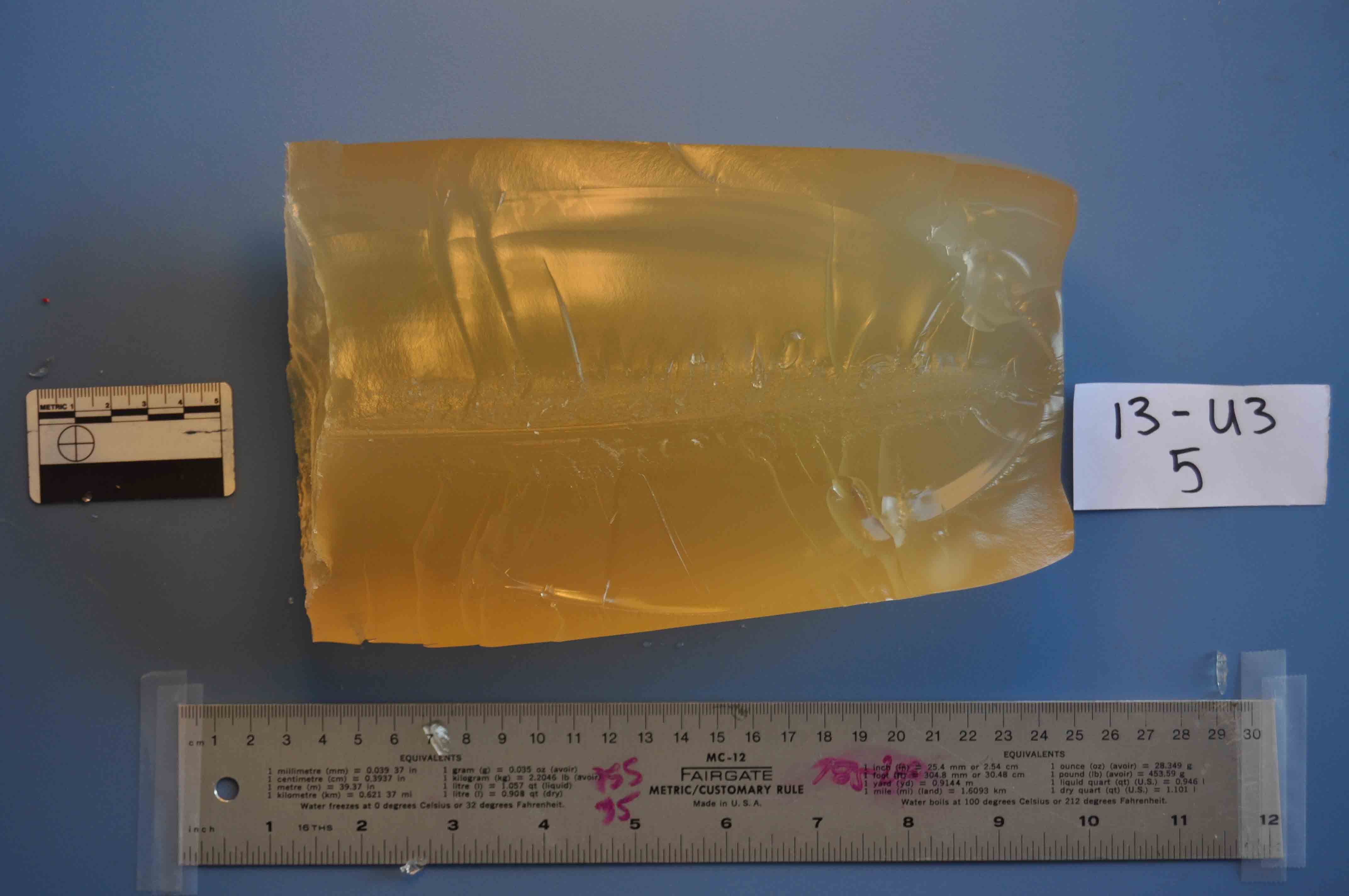

Supplement: File S2 — Wound track images, shapefiles, and tps files. (ZIP) [file pone.0104514.s002.zip › File S2/JPEGS/U3-5c.jpg]

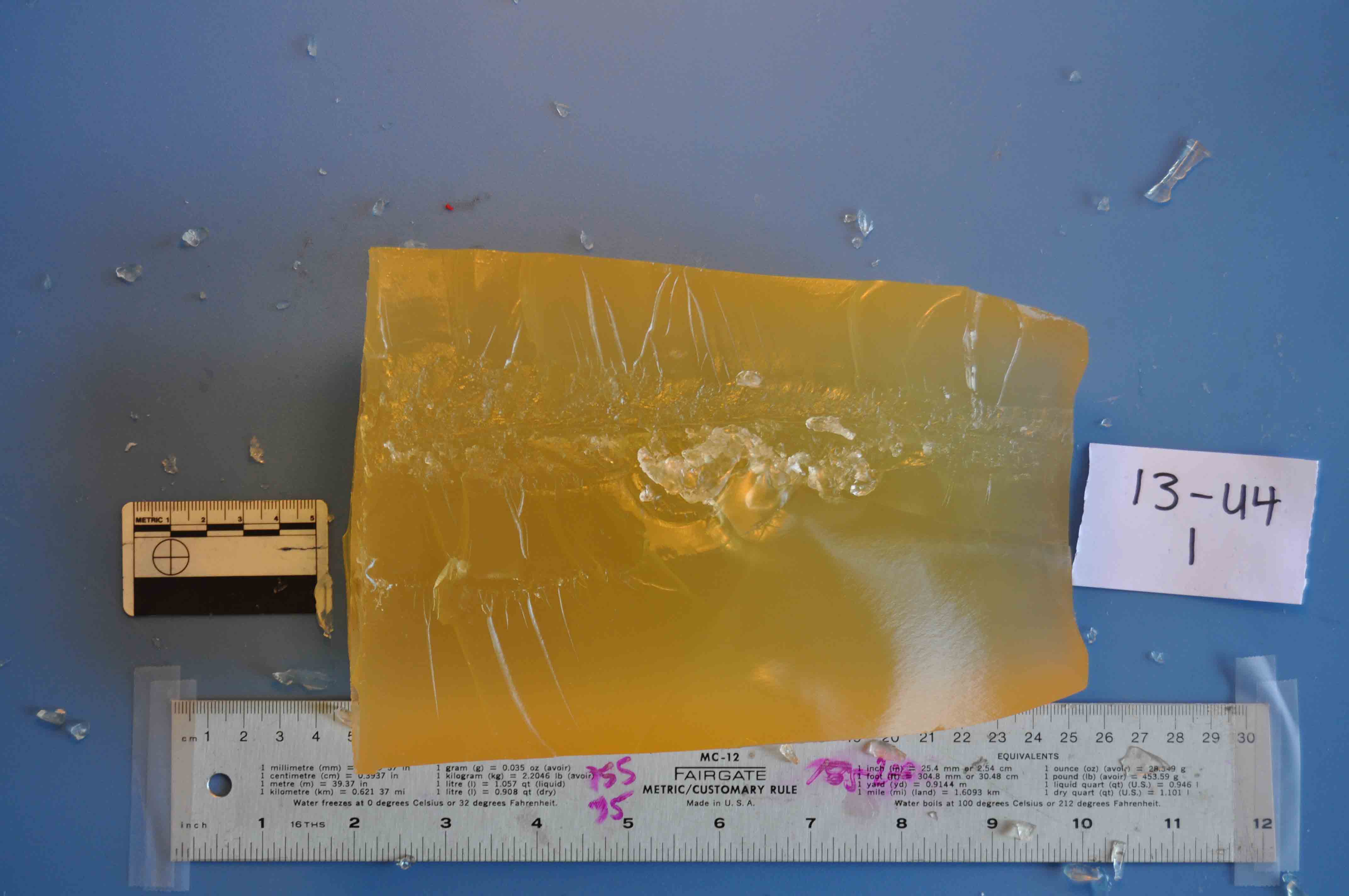

Supplement: File S2 — Wound track images, shapefiles, and tps files. (ZIP) [file pone.0104514.s002.zip › File S2/JPEGS/U4-1a.jpg]

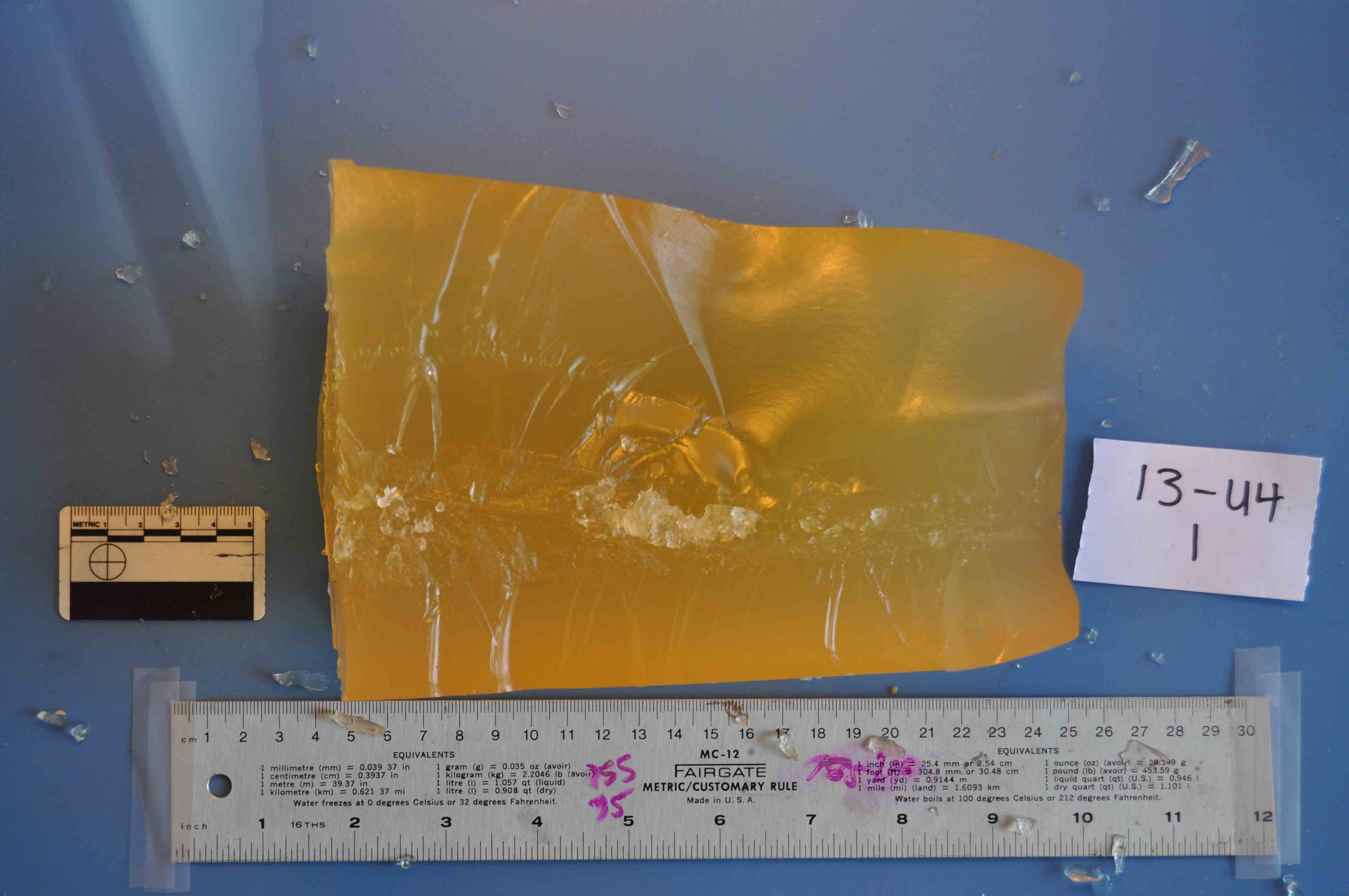

Supplement: File S2 — Wound track images, shapefiles, and tps files. (ZIP) [file pone.0104514.s002.zip › File S2/JPEGS/U4-1b.jpg]

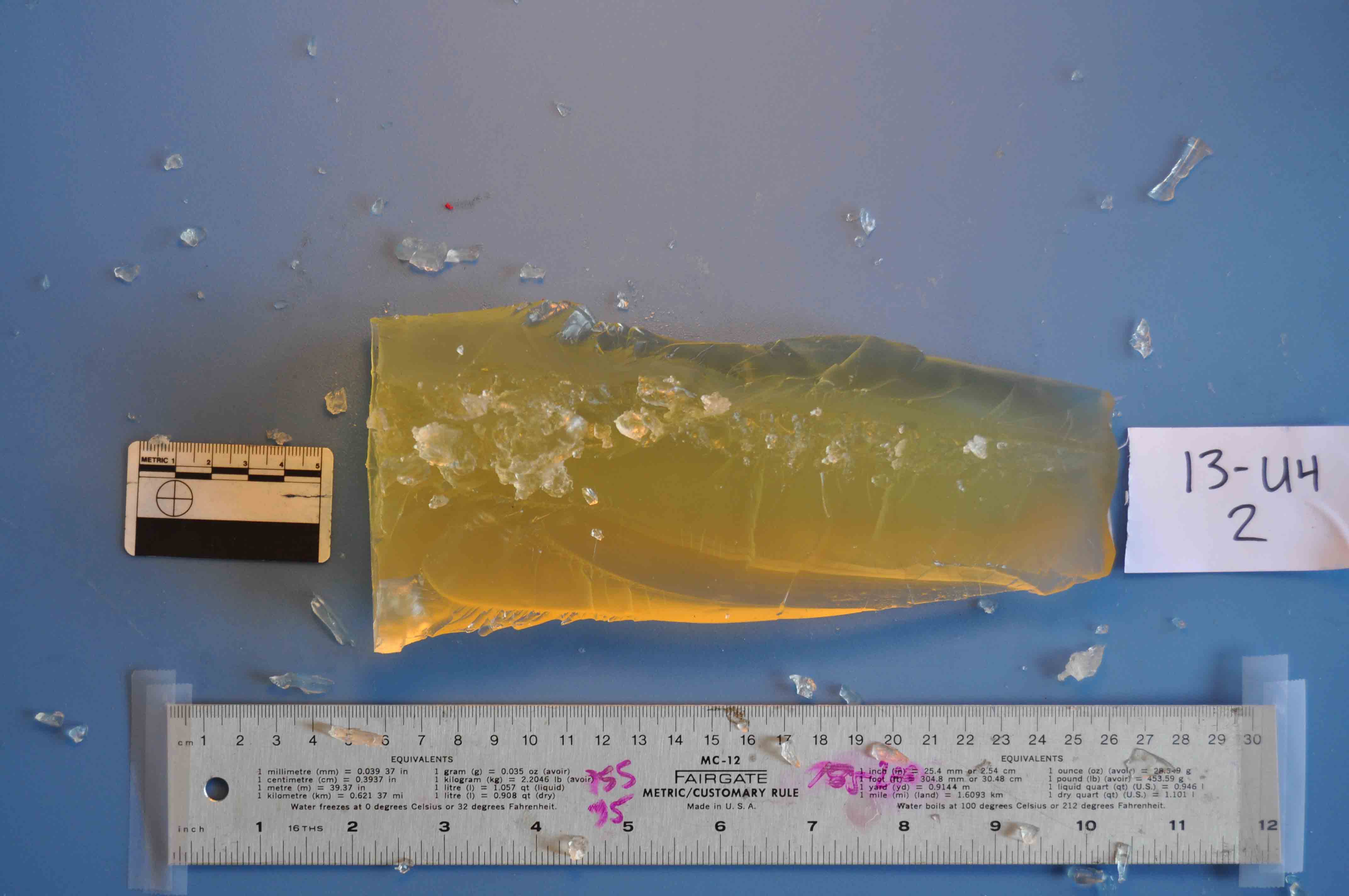

Supplement: File S2 — Wound track images, shapefiles, and tps files. (ZIP) [file pone.0104514.s002.zip › File S2/JPEGS/U4-2a.jpg]

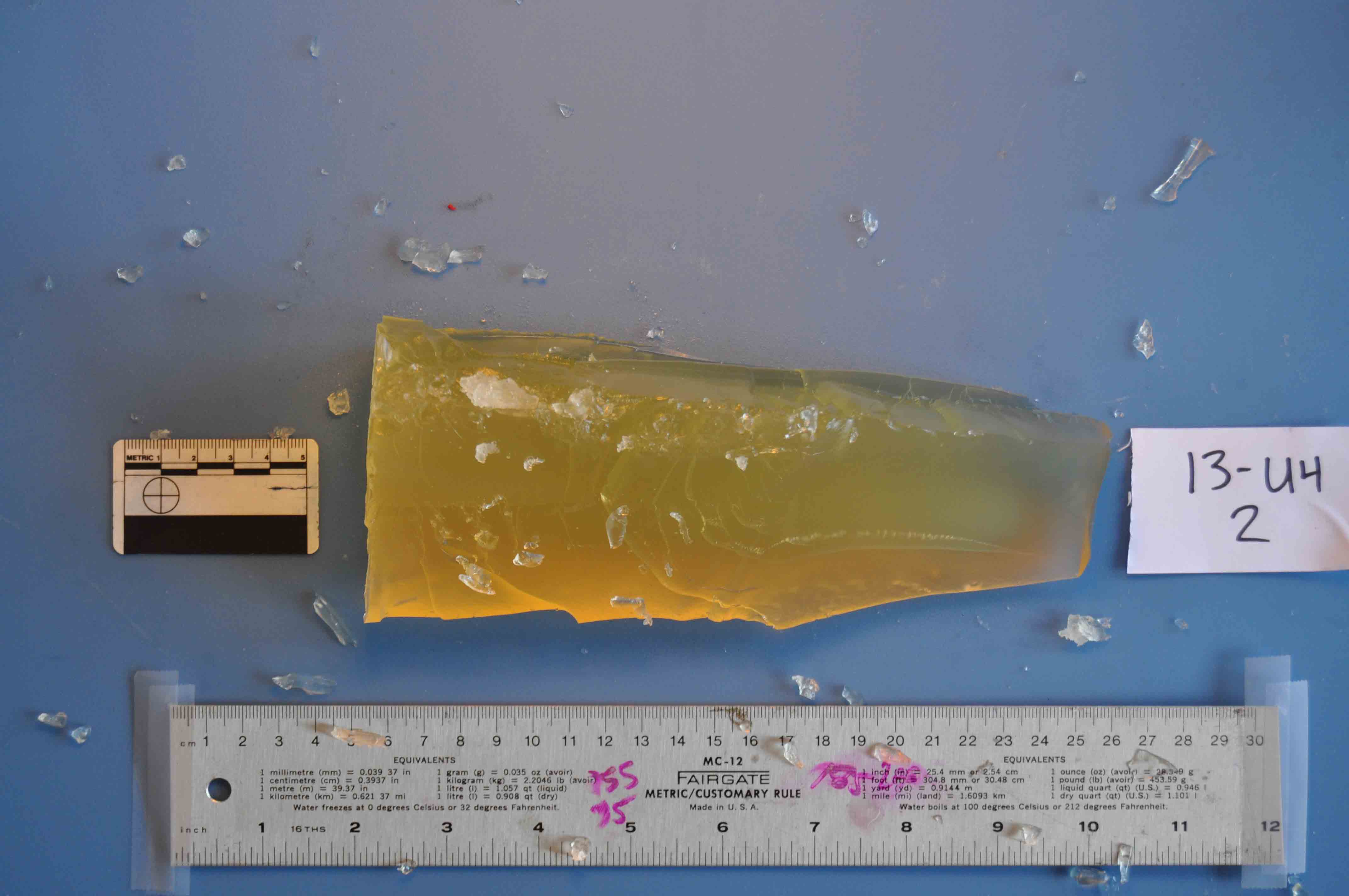

Supplement: File S2 — Wound track images, shapefiles, and tps files. (ZIP) [file pone.0104514.s002.zip › File S2/JPEGS/U4-2b.jpg]

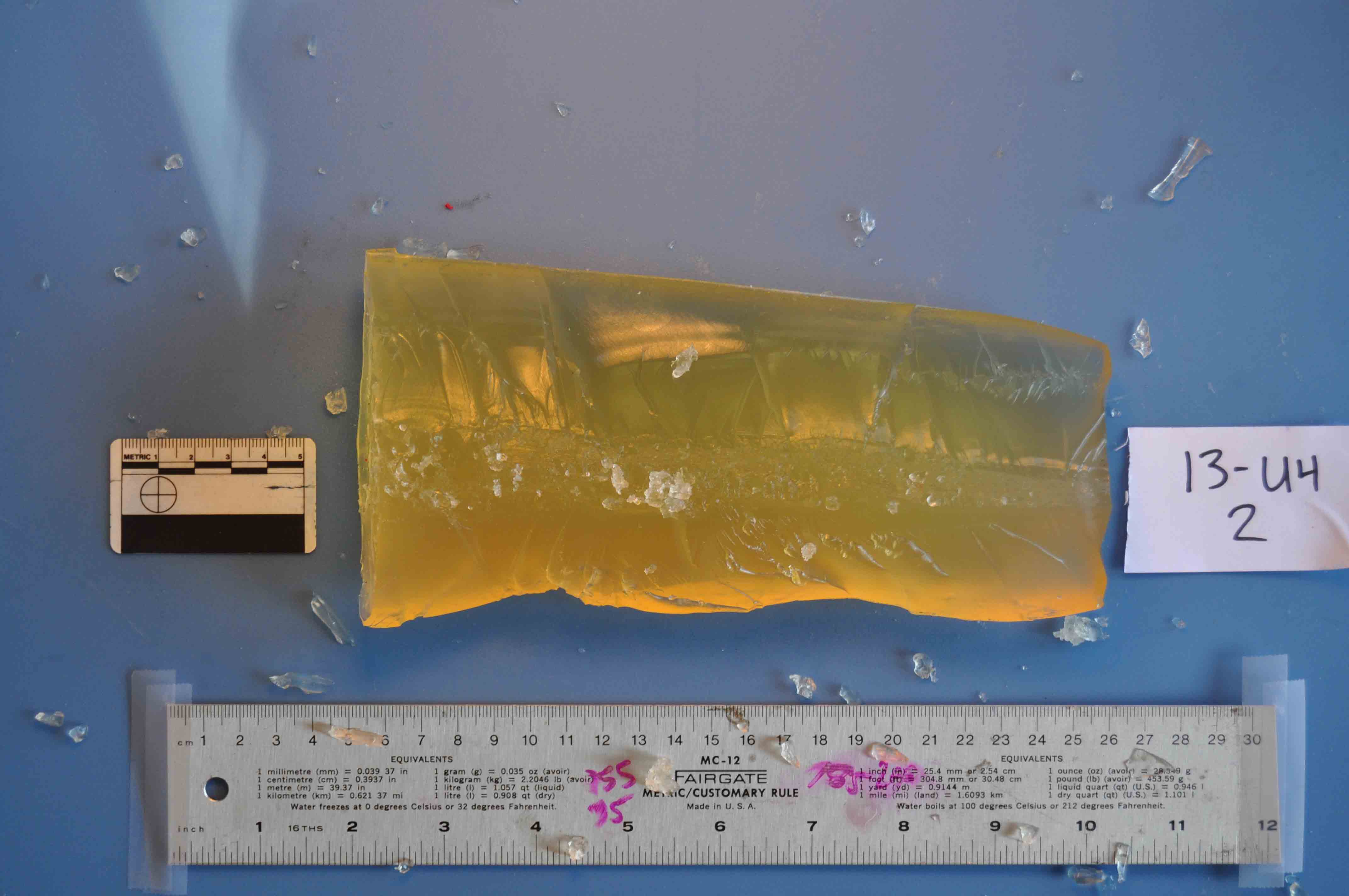

Supplement: File S2 — Wound track images, shapefiles, and tps files. (ZIP) [file pone.0104514.s002.zip › File S2/JPEGS/U4-2c.jpg]

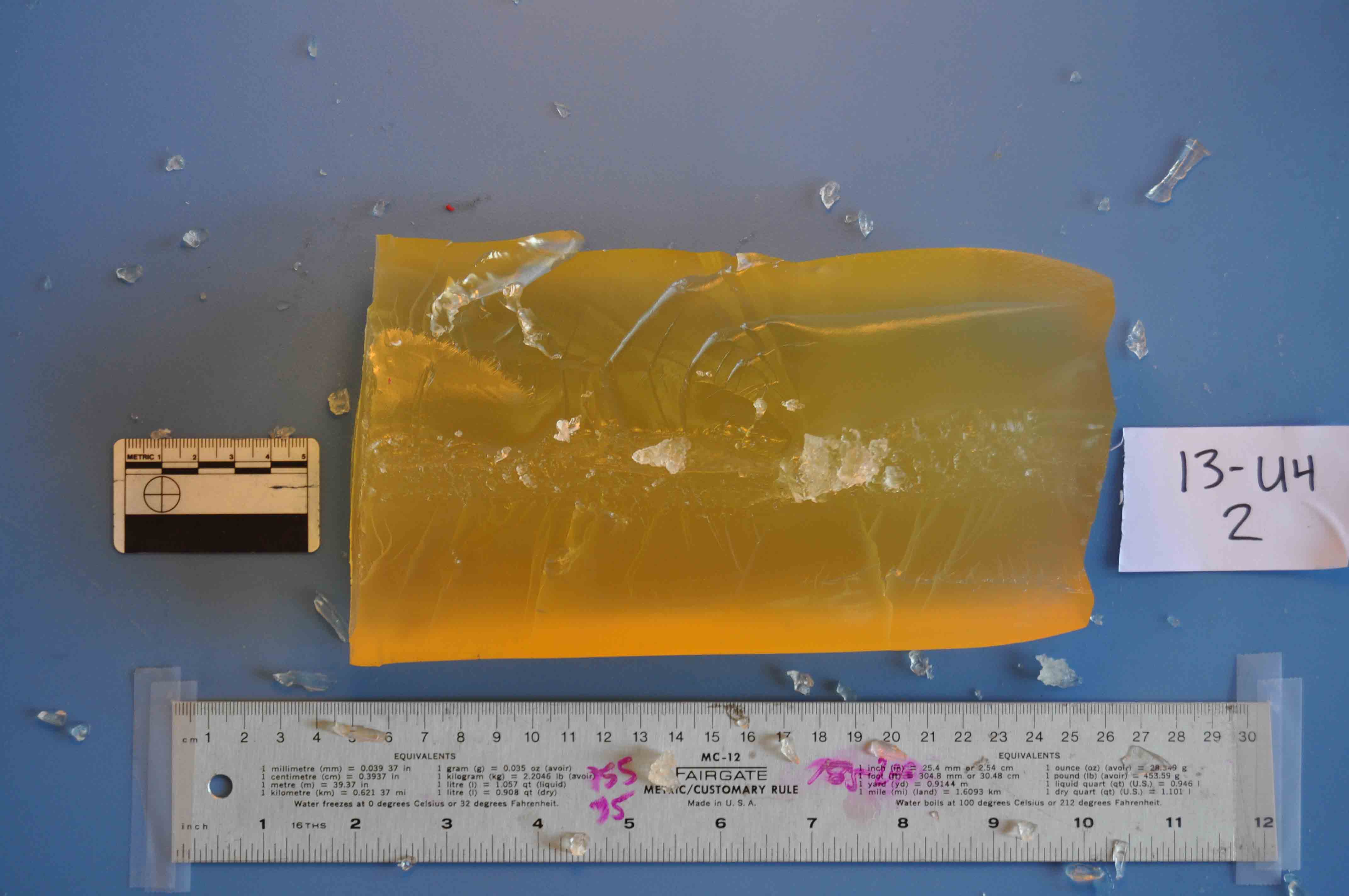

Supplement: File S2 — Wound track images, shapefiles, and tps files. (ZIP) [file pone.0104514.s002.zip › File S2/JPEGS/U4-2d.jpg]

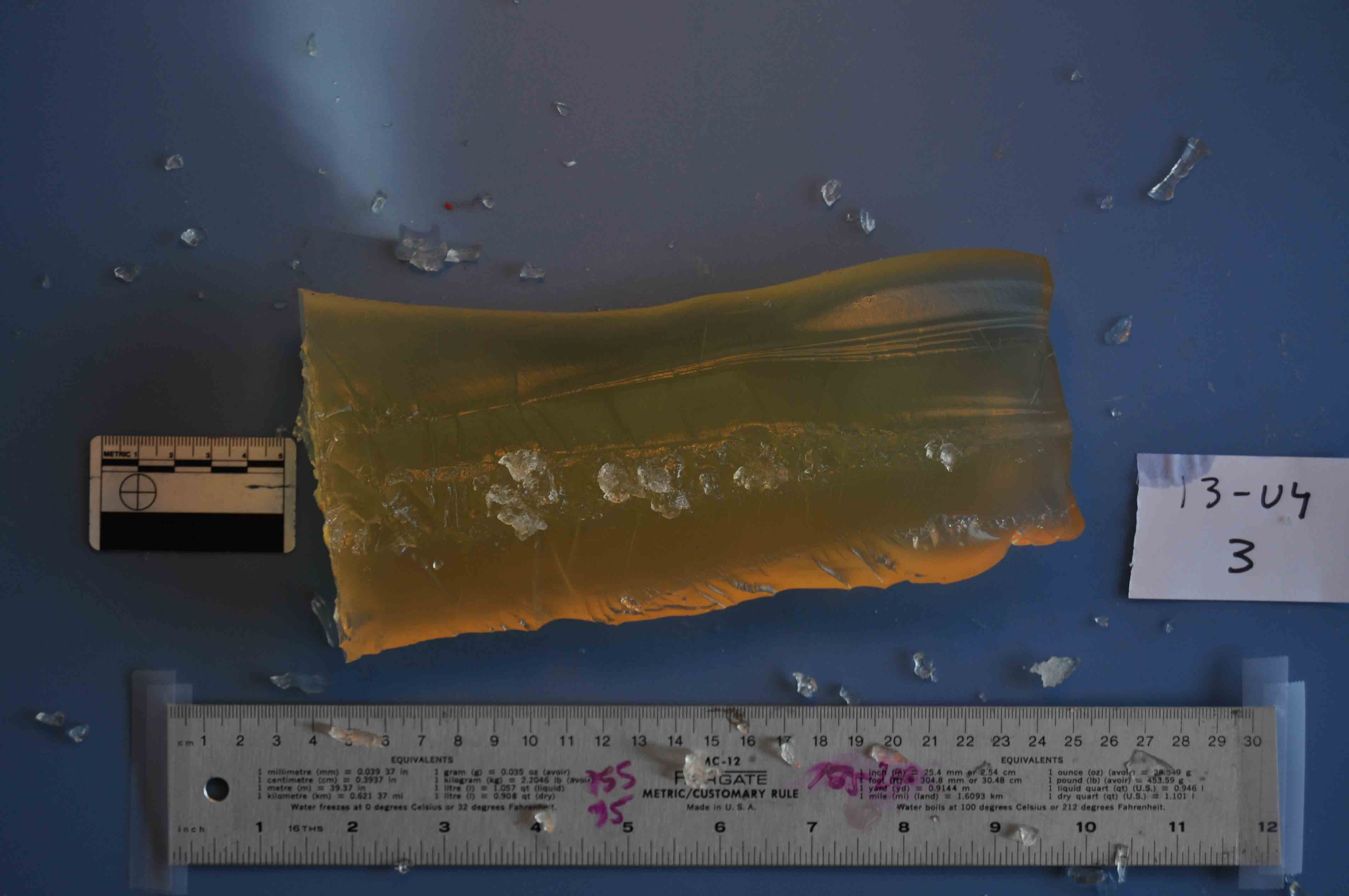

Supplement: File S2 — Wound track images, shapefiles, and tps files. (ZIP) [file pone.0104514.s002.zip › File S2/JPEGS/U4-3a.jpg]

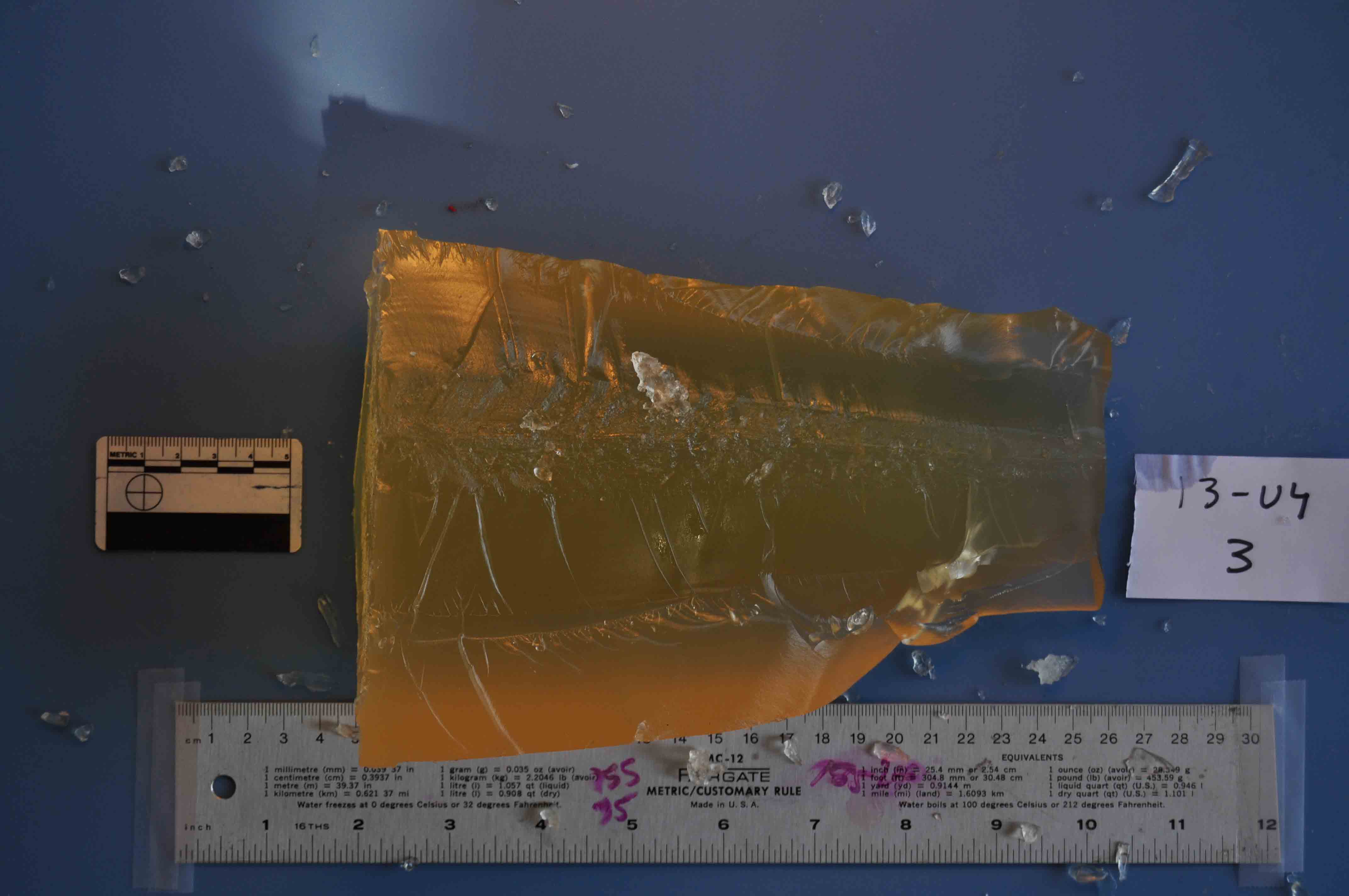

Supplement: File S2 — Wound track images, shapefiles, and tps files. (ZIP) [file pone.0104514.s002.zip › File S2/JPEGS/U4-3b.jpg]

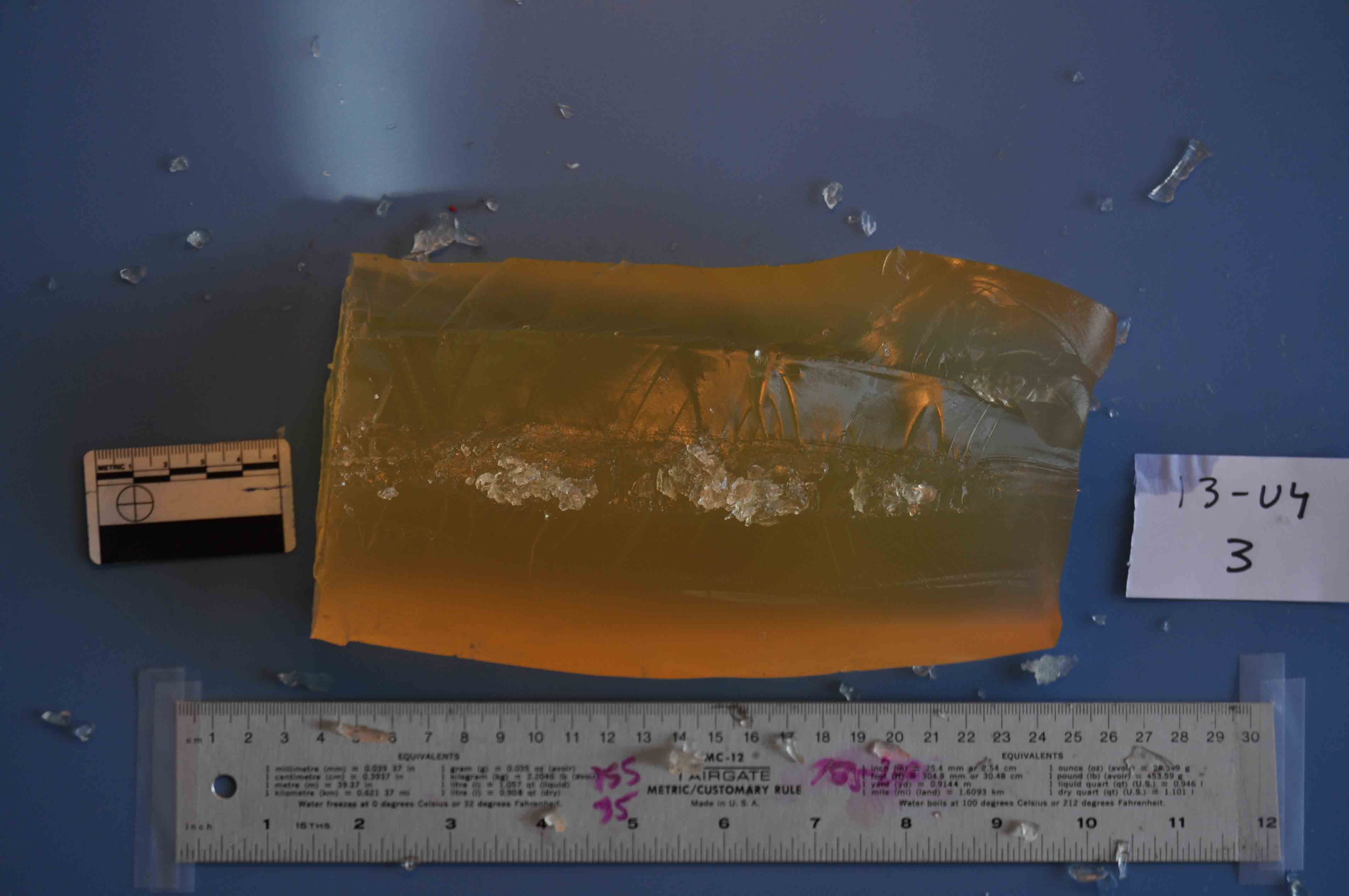

Supplement: File S2 — Wound track images, shapefiles, and tps files. (ZIP) [file pone.0104514.s002.zip › File S2/JPEGS/U4-3d.jpg]

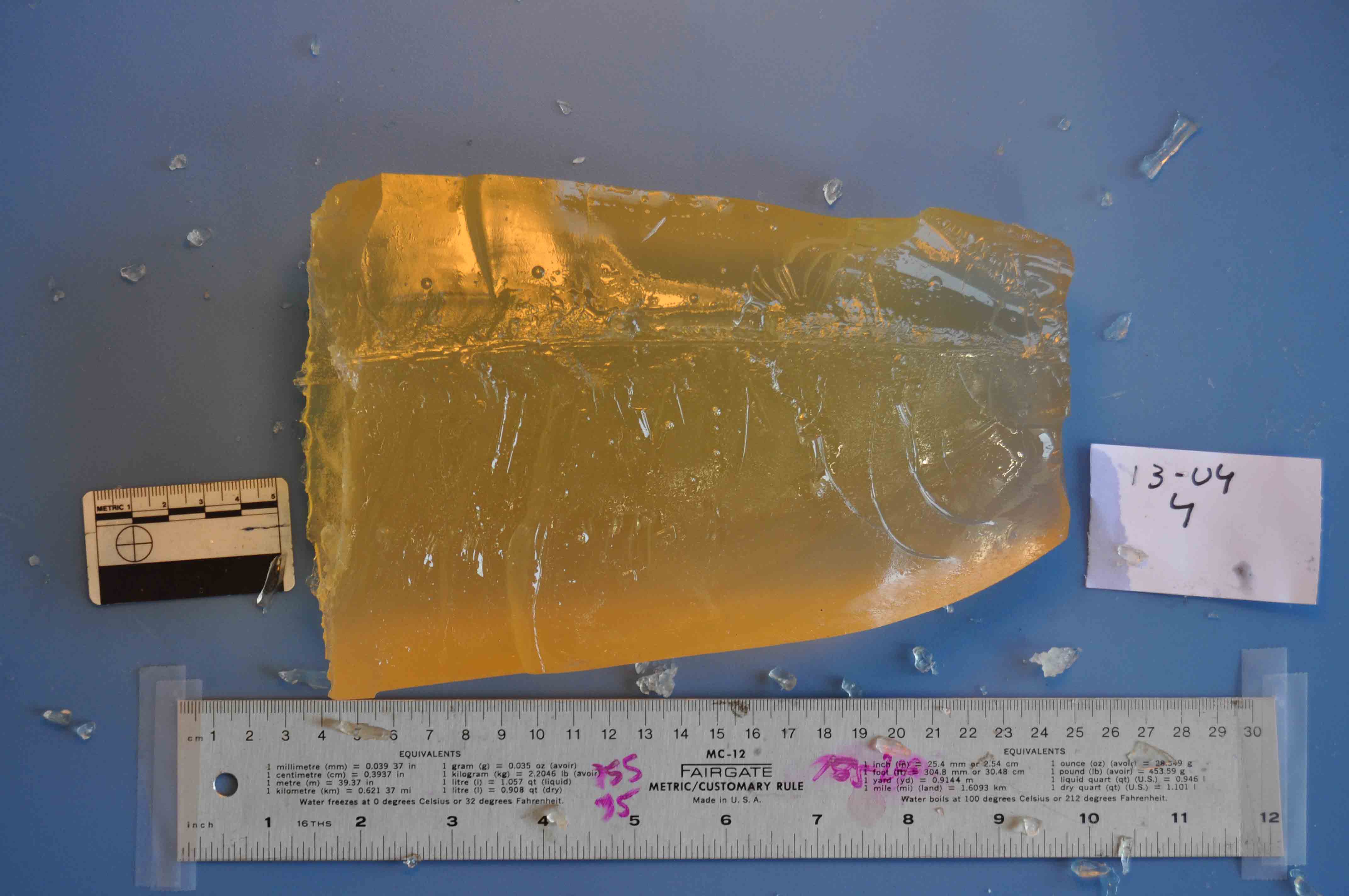

Supplement: File S2 — Wound track images, shapefiles, and tps files. (ZIP) [file pone.0104514.s002.zip › File S2/JPEGS/U4-4a.jpg]

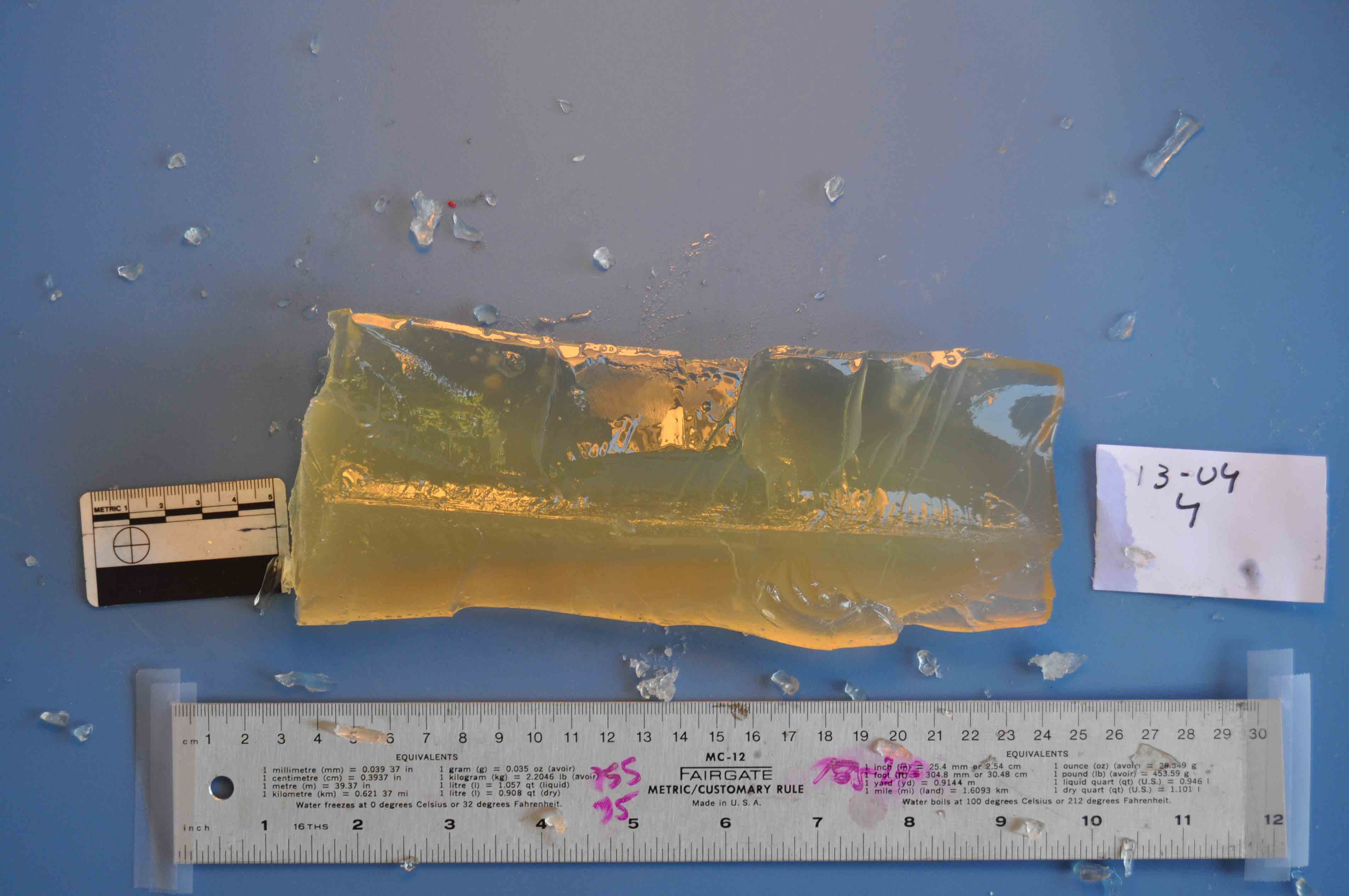

Supplement: File S2 — Wound track images, shapefiles, and tps files. (ZIP) [file pone.0104514.s002.zip › File S2/JPEGS/U4-4b.jpg]

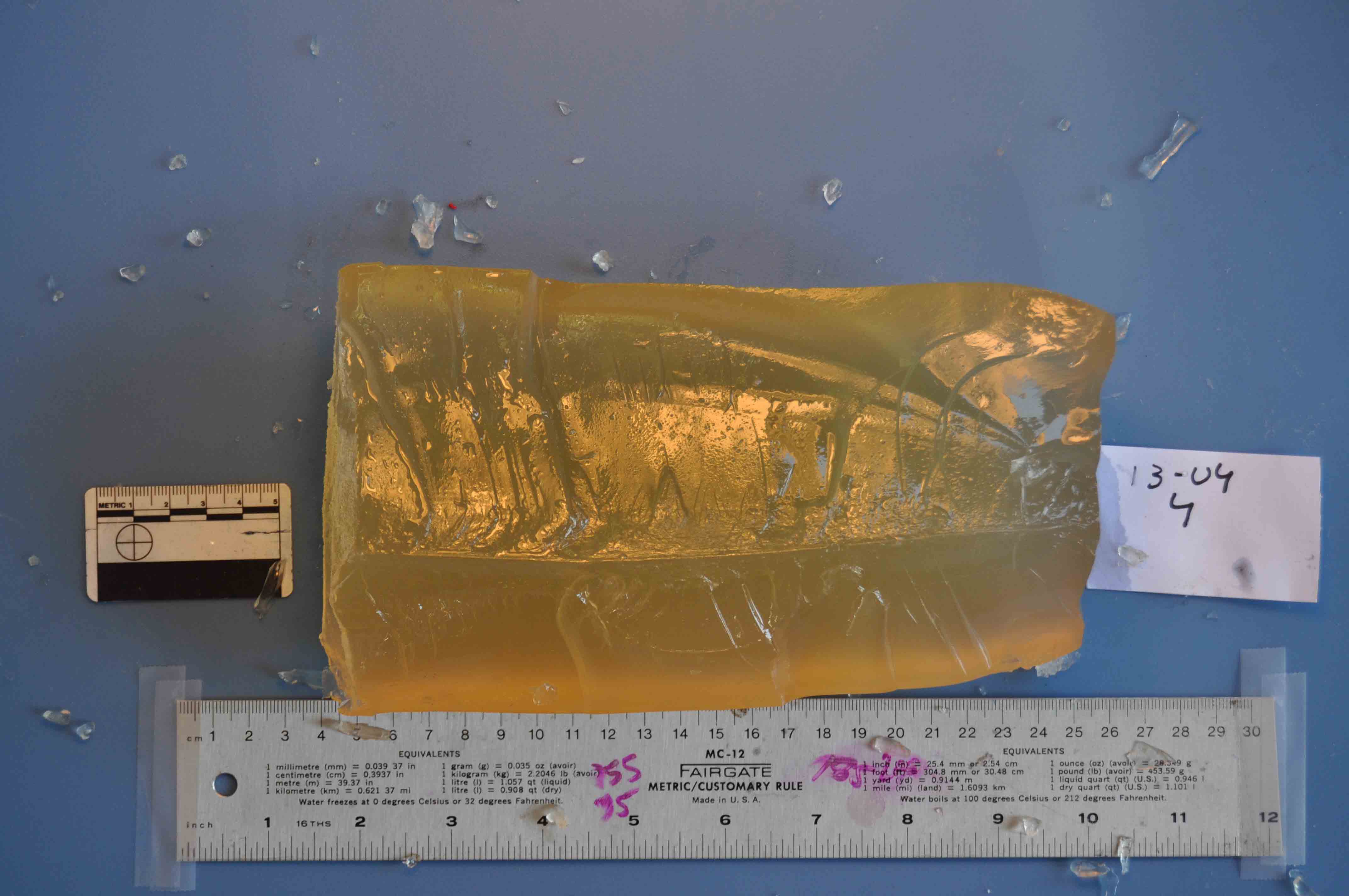

Supplement: File S2 — Wound track images, shapefiles, and tps files. (ZIP) [file pone.0104514.s002.zip › File S2/JPEGS/U4-4c.jpg]

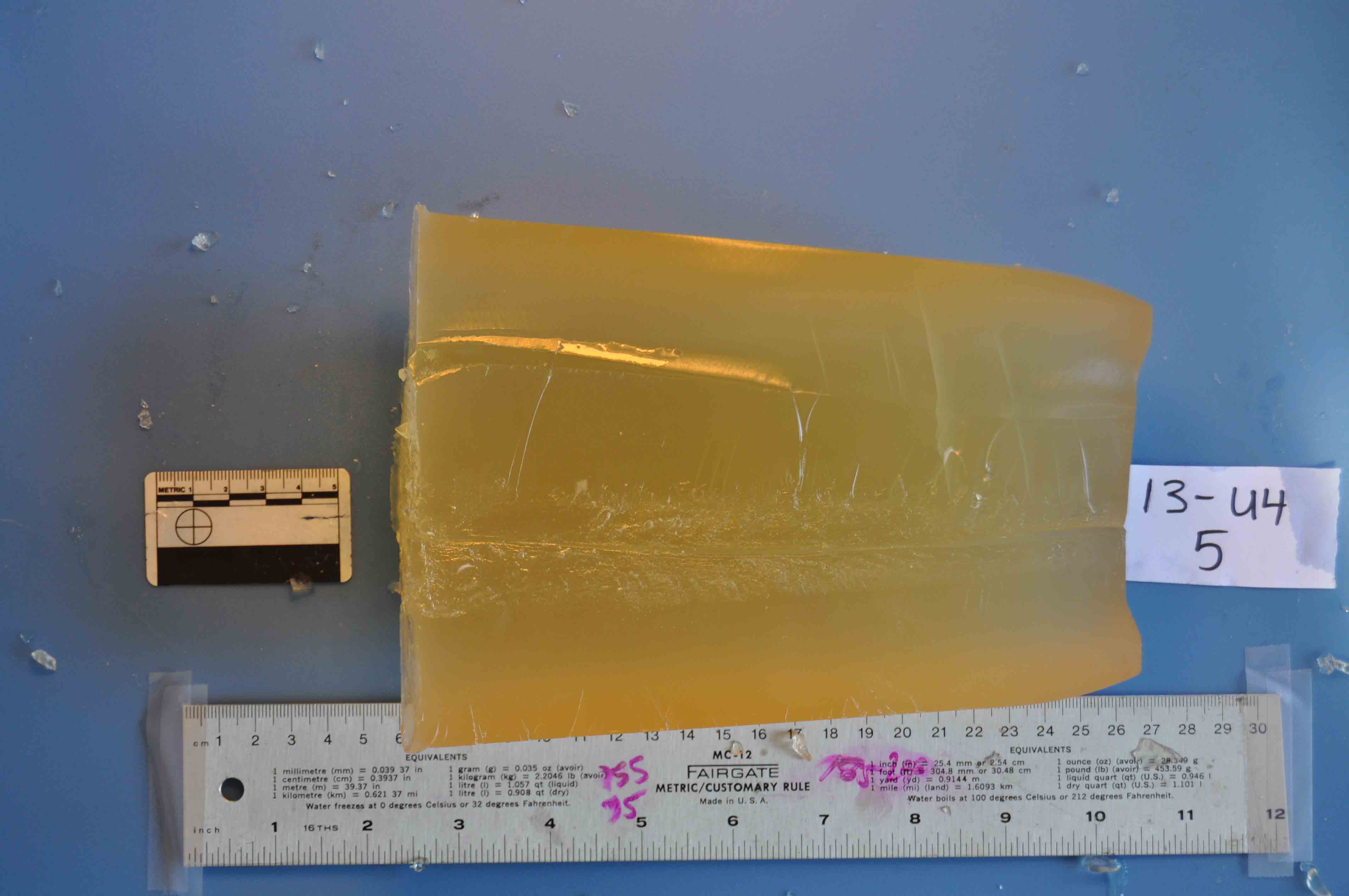

Supplement: File S2 — Wound track images, shapefiles, and tps files. (ZIP) [file pone.0104514.s002.zip › File S2/JPEGS/U4-5a.jpg]

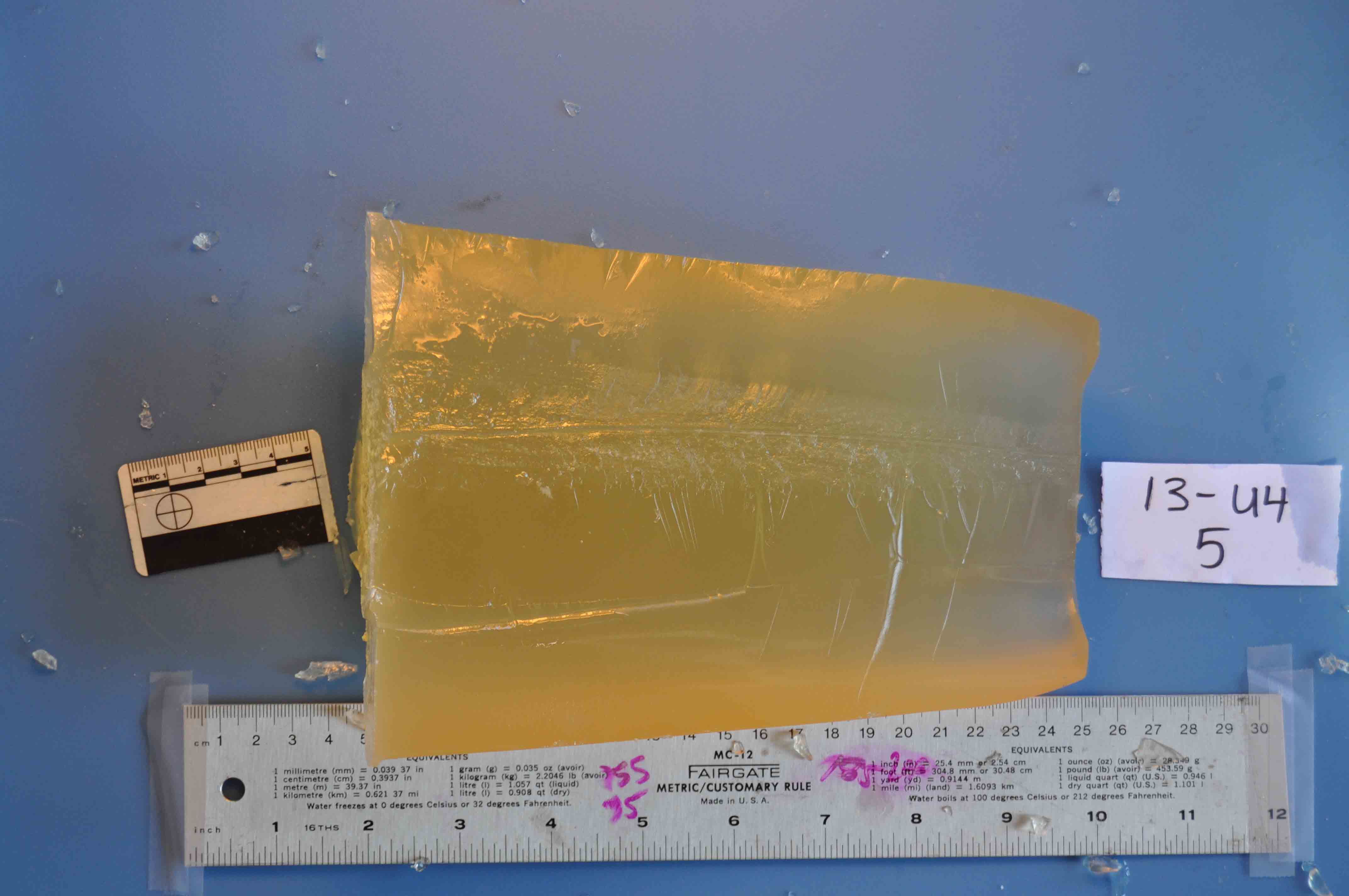

Supplement: File S2 — Wound track images, shapefiles, and tps files. (ZIP) [file pone.0104514.s002.zip › File S2/JPEGS/U4-5b.jpg]

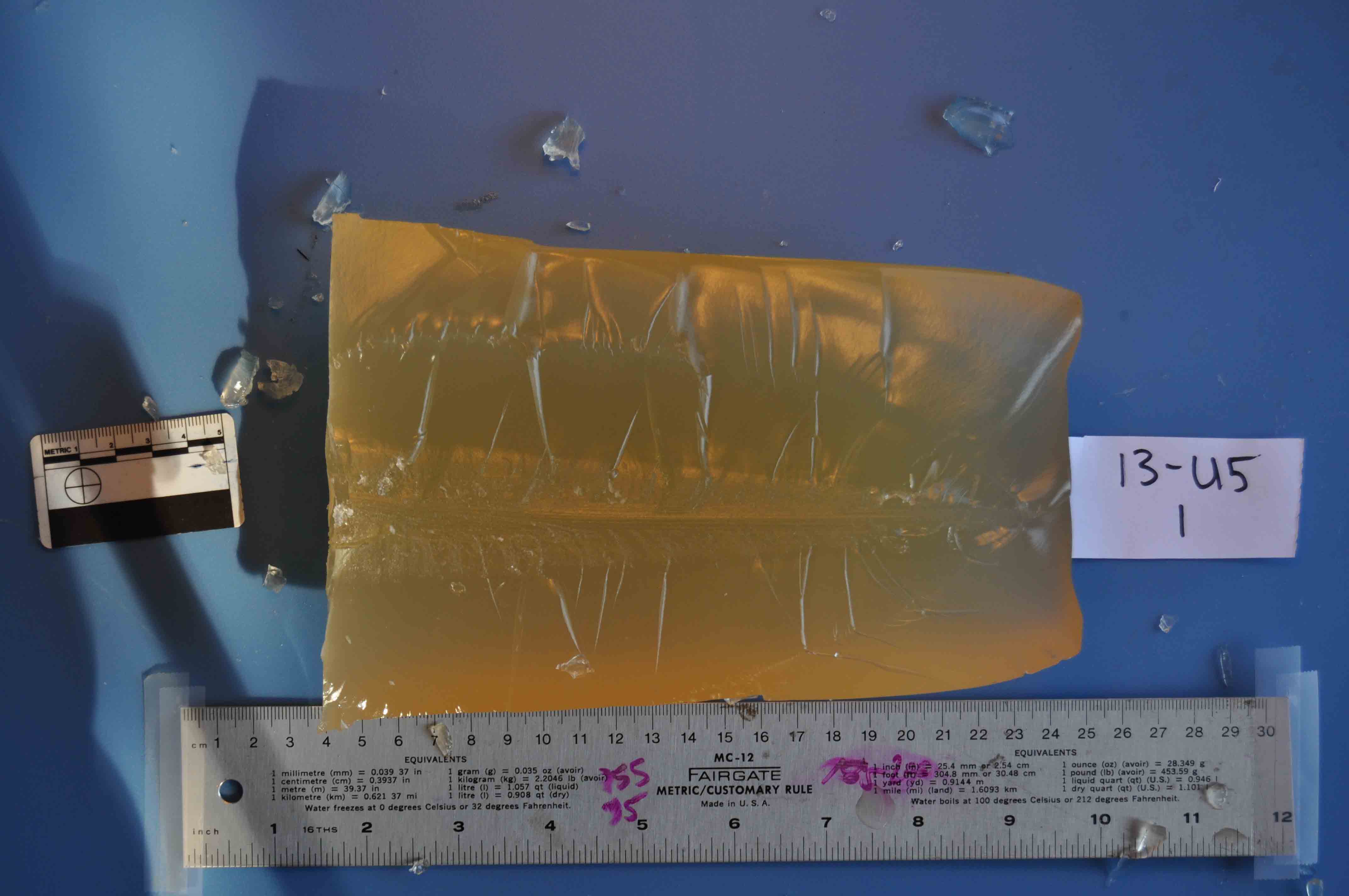

Supplement: File S2 — Wound track images, shapefiles, and tps files. (ZIP) [file pone.0104514.s002.zip › File S2/JPEGS/U5-1a.jpg]

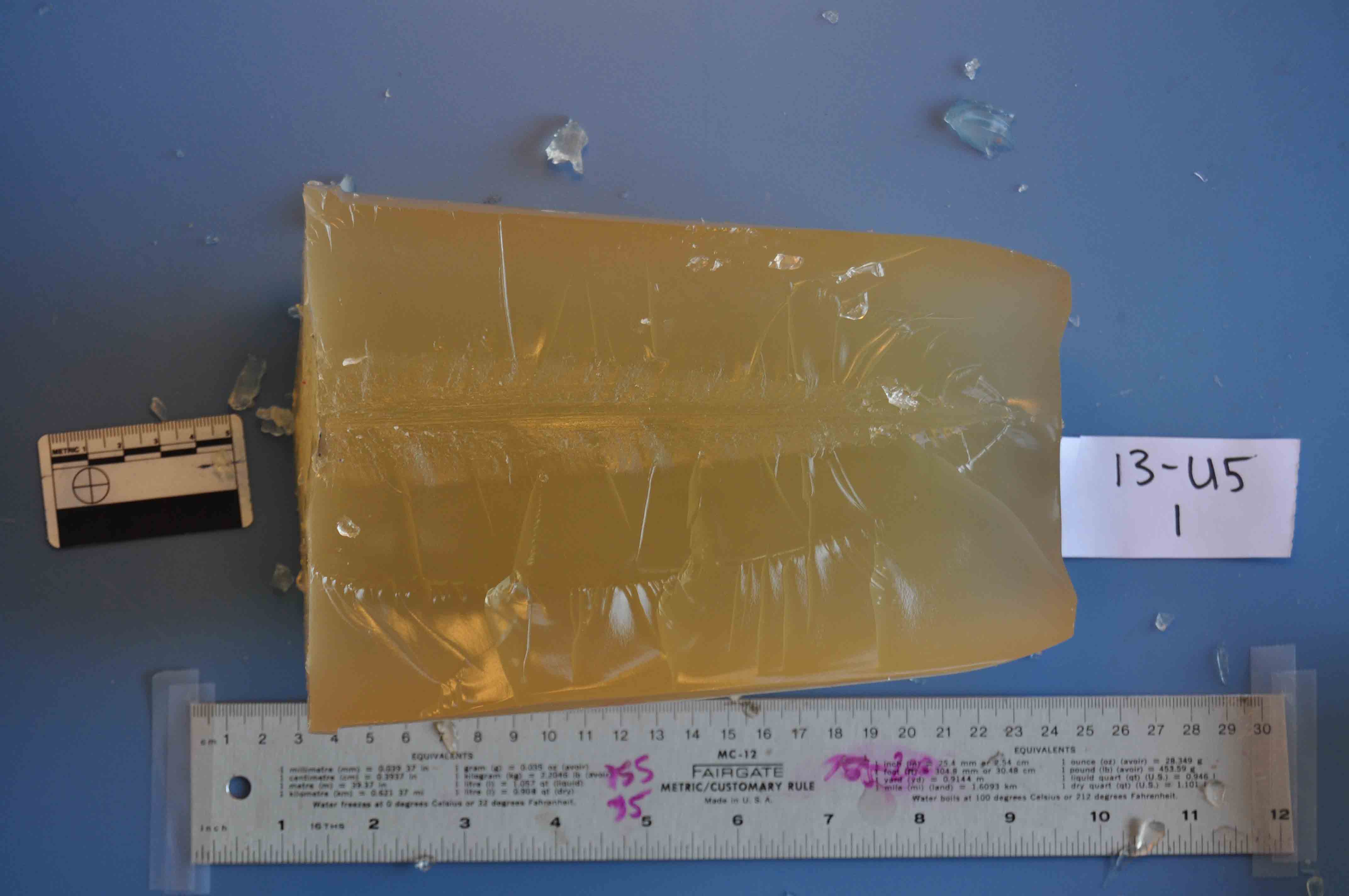

Supplement: File S2 — Wound track images, shapefiles, and tps files. (ZIP) [file pone.0104514.s002.zip › File S2/JPEGS/U5-1b.jpg]

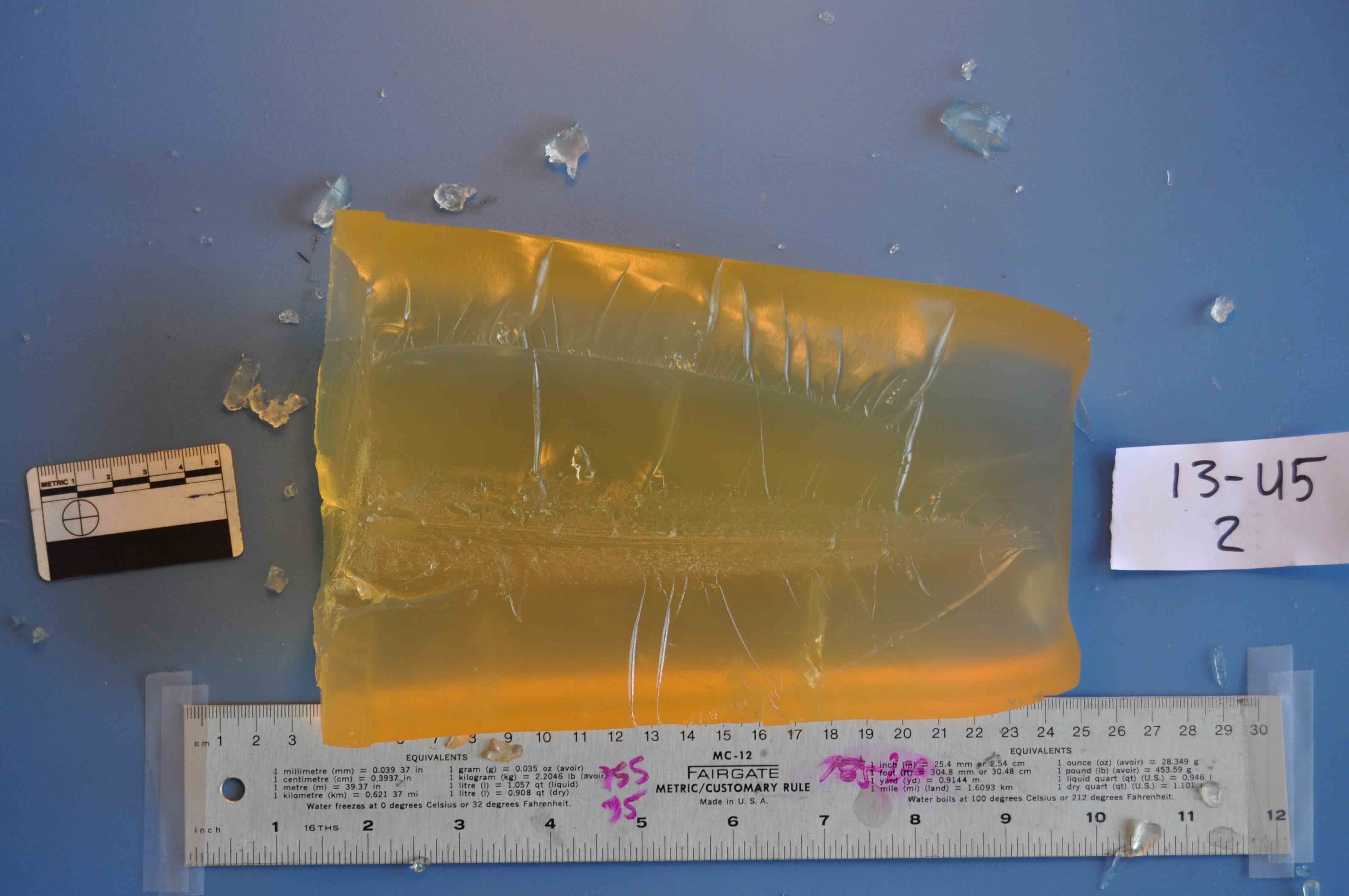

Supplement: File S2 — Wound track images, shapefiles, and tps files. (ZIP) [file pone.0104514.s002.zip › File S2/JPEGS/U5-2a.jpg]
